# Genome‐wide meta‐analysis and fine‐mapping prioritize potential causal variants and genes related to leprosy

**DOI:** 10.1002/mco2.415

**Published:** 2023-11-24

**Authors:** Zhenzhen Wang, Tingting Liu, Wenchao Li, Gongqi Yu, Zihao Mi, Chuan Wang, Xiaojie Liao, Pengcheng Huai, Tongsheng Chu, Dianchang Liu, Lele Sun, Xi'an Fu, Yonghu Sun, Honglei Wang, Na Wang, Jianjun Liu, Hong Liu, Furen Zhang

**Affiliations:** ^1^ Department of Biostatistics School of Public Health Cheeloo College of Medicine Shandong University Jinan Shandong China; ^2^ Shandong Provincial Key Lab for Dermatovenereology, Shandong Provincial Hospital for Skin Diseases & Shandong Provincial Institute of Dermatology and Venereology, Shandong First Medical University & Shandong Academy of Medical Sciences Jinan Shandong China; ^3^ Department of Human Genetics, Genome Institute of Singapore Singapore Singapore

**Keywords:** fine‐mapping, genome‐wide association study, immune response, leprosy, pathway analysis

## Abstract

To date, genome‐wide association studies (GWASs) have discovered 35 susceptible loci of leprosy; however, the cumulative effects of these loci can only partially explain the overall risk of leprosy, and the causal variants and genes within these loci remain unknown. Here, we conducted out new GWASs in two independent cohorts of 5007 cases and 4579 controls and then a meta‐analysis in these newly generated and multiple previously published (2277 cases and 3159 controls) datasets were performed. Three novel and 15 previously reported risk loci were identified from these datasets, increasing the known leprosy risk loci of explained genetic heritability from 23.0 to 38.5%. A comprehensive fine‐mapping analysis was conducted, and 19 causal variants and 14 causal genes were identified. Specifically, manual checking of epigenomic information from the Epimap database revealed that the causal variants were mainly located within the immune‐relevant or immune‐specific regulatory elements. Furthermore, by using gene‐set, tissue, and cell‐type enrichment analyses, we highlighted the key roles of immune‐related tissues and cells and implicated the PD‐1 signaling pathways in the pathogenetic mechanism of leprosy. Collectively, our study identified candidate causal variants and elucidated the potential regulatory and coding mechanisms for genes associated with leprosy.

## Graphical abstract

In the present study, three novel risk loci were identified from meta‐analysis of six leprosy GWASs (green boxes). Subsequent comprehensive fine‐mapping analysis revealed the causal variants were mainly located within the immune‐relevant or immune‐specific regulatory elements (blue boxes). Further enrichment and pheWAS analyses (brown boxes) expanded our understanding of immune‐relevant mechanisms underlying leprosy based on the genetic basis.

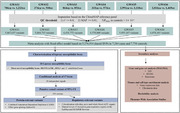

## 1 INTRODUCTION

Infectious diseases remain one of the major challenges to human health and survival for centuries, and they are among the leading causes of death and disability worldwide.^1^, ^2^ The occurrence and progression of infectious diseases depend on host exposure to pathogens and the invasiveness of pathogens. Individuals exposed to the same pathogen, however, show diverse clinical manifestations, thus indicating the critical role of host genetic factors in disease outcomes.^3^, ^4^ In the past few decades, a series of susceptibility loci of infectious diseases have been found through candidate gene analysis or genome‐wide association studies (GWASs), expanding our understanding of the genetic basis of infectious diseases.^5^

Leprosy, a chronic granulomatous infectious disease caused by *Mycobacterium leprae*, primarily affects the skin and peripheral nerves and leads to irreversible disability and deformities.^3^ Given that only a small number of individuals exposed to *M. leprae* are successfully infected and that clinical disease outcomes show marked interindividual variations, leprosy is an ideal model for genetic studies.^3^ Thus far, 35 susceptibility loci/genes of leprosy have been identified by GWASs, providing new insights into the pathogenesis of leprosy by implicating the roles of both innate and adaptive immune responses against *M. leprae*.^6^, ^7^, ^8^, ^9^, ^10^, ^11^, ^12^, ^13^, ^14^ However, all of the identified susceptibility genes/loci can explain only 23.0% of the overall risk of leprosy.^12^ Given an estimated heritability of up to 57%,^15^ more genetic susceptibility loci remain to be revealed.

GWAS loci often harbor many variants and genes, and most of the significantly associated variants are located in the nonprotein‐coding regions of the genome.^16^ For example, 65.7% (23 out of 35) of the established GWAS variants of leprosy are located in the noncoding regions of the genome.^6^, ^7^, ^8^, ^9^, ^10^, ^11^, ^12^, ^13^, ^14^, ^17^, ^18^ Thus, post‐GWASs mainly focus on fine‐mapping research, which could enable to identify causal genes or variants and establish a foundation for further functional exploration and interpretation of the pathogenesis of diseases.^19^, ^20^ Large‐scale genetic fine‐mapping analyses have been conducted for infectious and autoimmune diseases.^16^, ^19^, ^21^, ^22^ For instance, putative causal genes identified by expression quantitative trait loci (eQTL) colocalization display the importance of hair follicles in susceptibility to acne^23^; causal variants identified by multiomic epigenetic annotation are implicated in oligodendrocyte‐ and neuron‐specific functions in Alzheimer's disease and Parkinson's disease^24^; and tissue‐ or cell‐type enrichment analysis enables to choose the relevant tissue or cell type and to further elucidate the molecular mechanisms associated with significant loci.^25^, ^26^ These fine‐mapping approaches demonstrated the complexities of interpreting functional noncoding single nucleotide polymorphisms (SNPs), thus contributing to the identification of new therapeutic targets.

To further characterize the genetic architecture of leprosy, we conducted a meta‐analysis of GWASs involving 7284 cases and 7738 controls and further performed fine‐mapping and functional analyses of all the 18 identified loci, except the major histocompatibility complex (MHC) region. Our study revealed 19 putative causal genetic variants and 14 candidate disease genes within nine leprosy loci. Gene‐set analysis and tissue and cell specificity analysis showed the important role of the synergistic antimicrobial responses between phagocytes and T cells through multiple immune‐related pathways in leprosy.

## 2 RESULTS

### 2.1 Meta‐analysis identified three new leprosy‐associated loci

In the present study, two new GWASs (GWAS5 and GWAS6) were subjected to genotyping by a population‐optimized array (Illumina Asian Screening Array); GWAS5 included 3298 leprosy cases and 3272 healthy controls from northern China, while GWAS6 included 2302 leprosy cases and 1632 healthy controls from southern China. Principal component (PC) analysis confirmed that all the cases and controls in both GWAS5 and GWAS6 were well matched (Figure S1). Finally, 474,857 SNPs in 6101 samples (2991 cases and 3110 controls) of GWAS5 and 477,111 SNPs in 3485 samples (2016 cases and 1469 controls) of GWAS6 passed the SNP and sample quality control (QC) filters. Demographic information and more details of these studies are provided in the *Methods* section and Table S1.

The two new GWASs and four previously published GWASs (GWAS1−4)^6^, ^9^, ^11^ were then imputed separately by using the Chinese population‐specific reference panel (China Metabolic Analytics Project, ChinaMAP), which contains 10,155 unrelated Chinese individuals and has been reported to achieve high‐quality imputation in Chinese populations.^27^ After performing stringent QC, 5,276,914 shared SNPs in 7284 cases and 7738 controls remained to be analyzed by meta‐analysis. The quantile–quantile (Q–Q) plots and genomic inflation factors for individual studies (*λ*_GC_ = 1.02–1.07) or the meta‐analysis (*λ*_GC_ = 1.09, *λ*_GC1000_ = 1.01) did not show evidence of a substantial inflation rate (Figure S2). An overview of the analysis strategy is presented in Figure S3.

We observed genome‐wide significant (*p* < 5 × 10^−8^) associations at 18 loci (Figures S4 and S5 and Table 1), including 15 previously established loci^6^, ^7^, ^9^, ^10^, ^13^ and three novel leprosy susceptibility loci located near the *METTL1, MYL2*, and *CSK* genes. Regarding the other known susceptibility loci, 12 susceptibility loci showed consistent associations at nominal significance (*p* < 0.05, same effect direction); however, the SNP rs4833095 in *TLR1* reported in an Indian population^14^ did not show any association (*p* = 0.74). The other three SNPs located in *CARD9*, *NCKIPSD*, and *HIF1A* and five SNPs located in *SLC7A2*, *TYK2*, *GIT2*, *ALDH2*, and *IL23R* did not pass the QC because of a low minor allele frequency (MAF) and low imputation quality score, respectively (Table S2 and Figure S4).

**TABLE 1:** Association statistics for 17 significant genome‐wide loci outside the MHC region.

Locus	N signal	SNP	CHR	BP	A1	A2	F_A	F_U	OR	*p* Value	95% CS SNPs	Putative causal gene(s)
**chr12:57,665,085‐58,665,085**	**1**	**rs10877013**	**12**	**58,165,085**	**C**	**T**	**0.32**	**0.35**	**0.85**	**1.93E−09**	**35**	METTL21B^a^,^b^,^c^,^f^ CYP27B1^b^,^c^ TSFM^b^,^c^,^d^,^f^ METTL1^e^,^f^
**chr12:110,914,461‐111,914,461**	**1**	**rs12229654**	**12**	**111,414,461**	**G**	**T**	**0.17**	**0.15**	**1.20**	**4.36E−08**	**NA**	MYL2^e^
**chr15:74,587,571‐75,587,571**	**1**	**rs117618569**	**15**	**75,087,571**	**A**	**T**	**0.22**	**0.18**	**1.22**	**7.70E−11**	**13**	CSK^e^,^f^
chr1:67,097,119‐68,097,119 (IL23R)	1	rs3762318	1	67,597,119	G	A	0.07	0.10	0.69	2.15E−17	20	C1orf141^e^
chr1:151,289,647‐152,289,647 (FLG)	1	rs12144914	1	151,789,647	G	T	0.48	0.45	1.14	4.70E−08	10	*RORC* ^e^
chr2:102,591,540‐103,591,540 (IL18RAP)	1	rs17027258	2	103,091,540	G	A	0.38	0.33	1.25	1.11E−17	40	*IL18RAP* ^c^ SLC9A4^e^
chr5:158,327,769‐159,327,769 (IL12B)	1	rs56167332	5	158,827,769	A	C	0.27	0.31	0.80	9.19E−16	3	IL12B^e^
chr6:146,420,271‐147,420,271 (RAB32)	1	rs74468352	6	146,920,271	T	C	0.26	0.21	1.26	2.48E−15	14	*ADGB* ^e^,^f^
chr8:90,315,235‐91,315,235 (RIPK2)	1	rs416324	8	90,815,235	A	G	0.44	0.49	0.76	5.30E−29	20	RIPK2^e^
chr9:117,079,504‐118,140,404 (TNFSF15)	2	rs10817678	9	117,579,457	A	G	0.44	0.49	0.75	1.55E−32	5	TNFSF15^a^,^e^,^f^
		rs149097644		117,641,097	A	G	0.02	0.01	1.41	1.02E−03	NA	TNFSF15^e^
chr10:63,991,638‐64,994,157 (EGR2)	2	rs10822054	10	64,480,252	T	G	0.09	0.12	0.77	6.98E−11	22	ADO^d^,^f^ ZNF365^e^
		rs80161952		64,494,302	T	C	0.07	0.06	1.34	5.99E−09	34	ZNF365^e^
chr10:72,610,977‐73,610,977 (SLC29A3)	1	rs780666	10	73,110,977	C	A	0.47	0.44	1.15	4.95E−08	23	SLC29A3^d^,^e^
chr12:40,190,061‐41,190,061 (*LRRK2*)	1	rs17443815	12	40,690,061	G	A	0.26	0.30	0.80	3.29E−15	33	LRRK2^e^
chr13:43,959,499‐45,012,908 (LACC1)	2	rs3764147	13	44,457,925	G	A	0.42	0.31	1.63	4.12E−81	5	LACC1^e^
		rs190879280		44,502,243	G	C	0.06	0.09	0.61	1.90E−25	4	LACC1^e^
chr16:10,960,480‐11,960,480 (CIITA‐SOCS1)	1	rs4781072	16	11,460,480	C	T	0.43	0.47	0.84	1.87E−12	2	RMI2^e^
chr16:28,008,048‐29,008,048 (IL27)	1	rs180744	16	28,508,048	G	A	0.10	0.13	0.78	7.40E−11	59	*IL2*7^d^ SULT1A2^d^,^c^ APOBR^e^
chr16:50,223,371‐51,223,371 (NOD2)	1	rs8063362	16	50,723,371	T	A	0.38	0.29	1.56	1.47E−63	8	NOD2^a^,^b^,^e^ SNX20^f^

Locus, the names of previously reported locus are indicated in parentheses.

N signal, the number of independent signals at this locus.

SNP, lead SNP of each independent signal; CHR, Chromosome; BP, position based on hg19 coordinates; A1, minor allele; A2, major allele.

F_A, minor allele frequency of affected cases; F_U, minor allele frequency of unaffected controls.

95% CS SNPs, the number of SNPs within the 95% credible set.

NA, not applicable.

^a^ Implicated through eQTL colocalization evidence PP > 0.8 in blood.

^b^ Implicated through eQTL colocalization evidence PP > 0.8 in nerve.

^c^ Implicated through eQTL colocalization evidence PP > 0.8 in skin.

^d^ Protein‐altering SNP or splicing in 95% credible set.

^e^ Nearest protein‐coding gene to SNP.

^f^ SNPs located in important gene‐regulatory regions by manual review the EpiMap dataset.

Bold indicates three novel locus.

Next, stepwise conditioning was used to identify independent signals at the 17 genome‐wide significant loci except the MHC region. Only three loci at *EGR2, TNFSF15*, and *LACC1* showed a second independent signal (Table S3 and Figures S5A–C).

We also estimated the heritability of leprosy by using all 38 significant loci (35 known and three novel susceptibility loci) and all the common SNPs across the genome. Assuming a population prevalence of 0.0001 for leprosy, the risk loci explained 6.71% of the variance in leprosy liability, while common genetic variation across the genome explained 17.43% (s.e. = 0.01) of the variance in leprosy liability. These results indicate that all the known leprosy risk loci explain 38.50% (6.71%/17.43%) of the overall genetic liability for leprosy.

### 2.2 Fine‐mapping analysis identified credibly causal variants and genes

**FIGURE 1:**
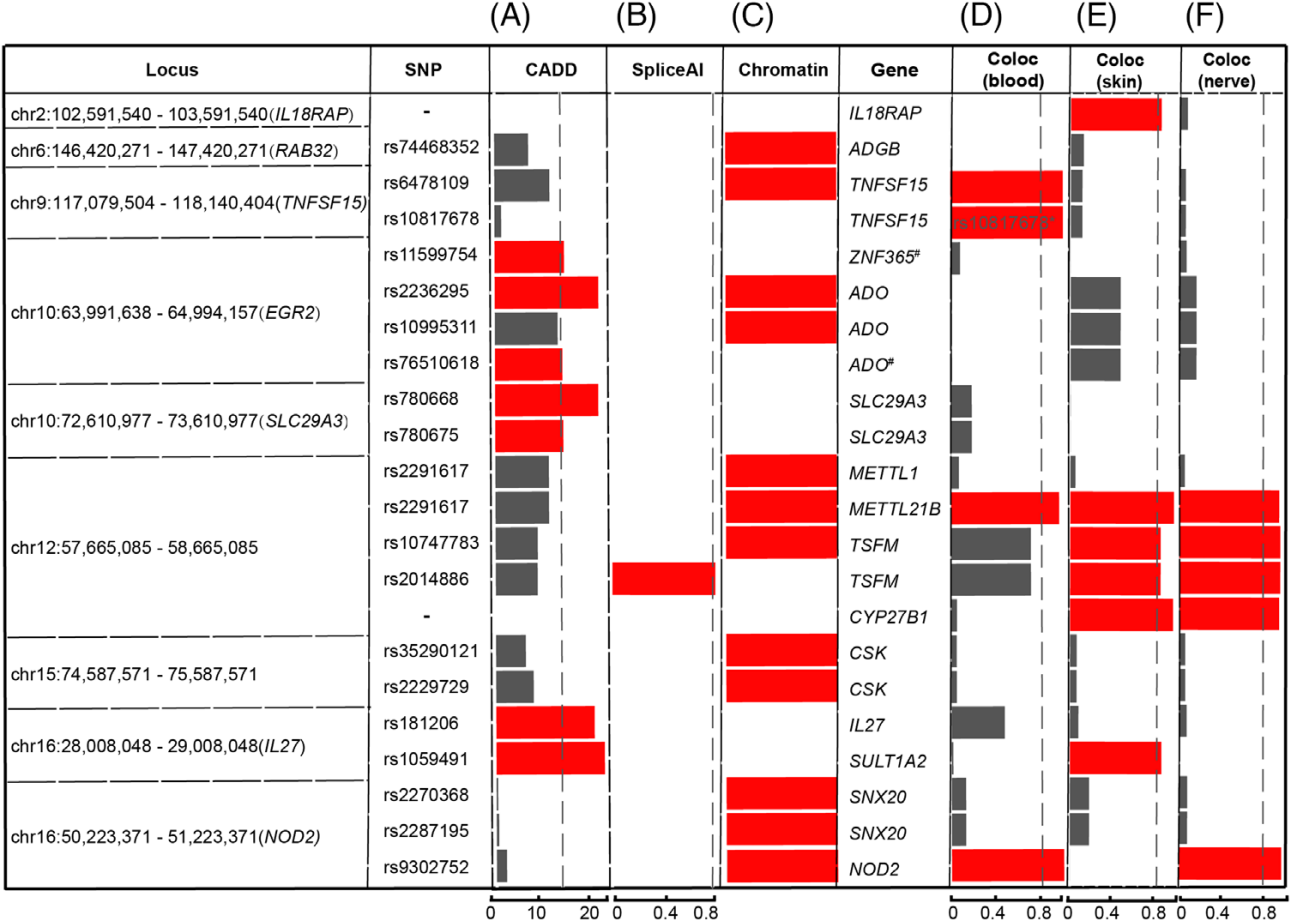
Fine‐mapping summary of all credibly causal variants and genes in nine significant genome‐wide loci. (A) CADD score of each credibly causal variant (red indicates CADD score > 15). (B) The Max SpliceAI score value of each credibly causal variant (red indicates SpliceAI score > 0.8). (C) Red indicates that credibly causal variants were located in important gene‐regulatory regions by manual review of the histone marker signals of H3K4me3 ChIP‐seq, H3K27ac ChIP‐seq, and DNase‐seq from PBMCs and skin from EpiMap. (D–F) The posterior probability (PP) of the GWAS and eQTL signals colocated in the same gene in the indicated tissue (red indicates PP.H4.abf > 0.8). ^*^In this loci, rs10817678 was identified as causal variant in *TNFSF15* gene by colocalization of GWAS and whole blood eQTL signals (SNP.PP.H4 > 0.8). ^#^Nearest protein‐coding gene to SNP. –, not applicable for three standards in (A)–(C).

To identify candidate causal variants and/or genes underlying 20 independent associations in the 17 loci outside the MHC region, we performed comprehensive fine‐mapping, colocalization, and network analyses (Figure S3). First, we limited the number of potential causal variants in all the 20 independent associations to 350 SNPs (95% credible set [CS]) by the susieR package in R software, which can implement methods for variable selection in linear regression based on the “Sum of Single Effects” (SuSiE) model. We then annotated all the 350 variants based on their potential deleterious effect on protein function or interference in gene expression regulation. We found eight protein‐damaging variants in four locus with a combined annotation‐dependent depletion (CADD) score > 15 (Figure 1A) or SpliceAI score > 0.8 (Figure 1B). We also identified another 11 regulatory variants in six locus through manual checking of chromatin interaction information from the Epigenome Browser and the HiCHIPdb database (Figure 1C) or a colocalization analysis by using eQTL datasets of skin, nerve, and whole blood (Figures 1D–F). All these 19 causal variants were located in 12 causal genes, including *ADGB*, *TNFSF15*, *ADO*, *SLC29A3*, *METTL1*, *METTL21B*, *TSFM*, *CSK*, *IL27*, *SULT1A2*, *SNX20*, and *NOD2*. In addition, *IL18RAP* and *CYP27B1* were pinpointed as causal genes through colocalized the leprosy‐associated signals with eQTL signals of skin and nerve (Figures 1E and F). Taken together, eight protein‐damaging variants and 11 regulatory variants were implicated as causal variants, and 14 genes were identified as causal genes. Figure 1 shows the results of the identified credibly causal variants and genes.

**FIGURE 2:**
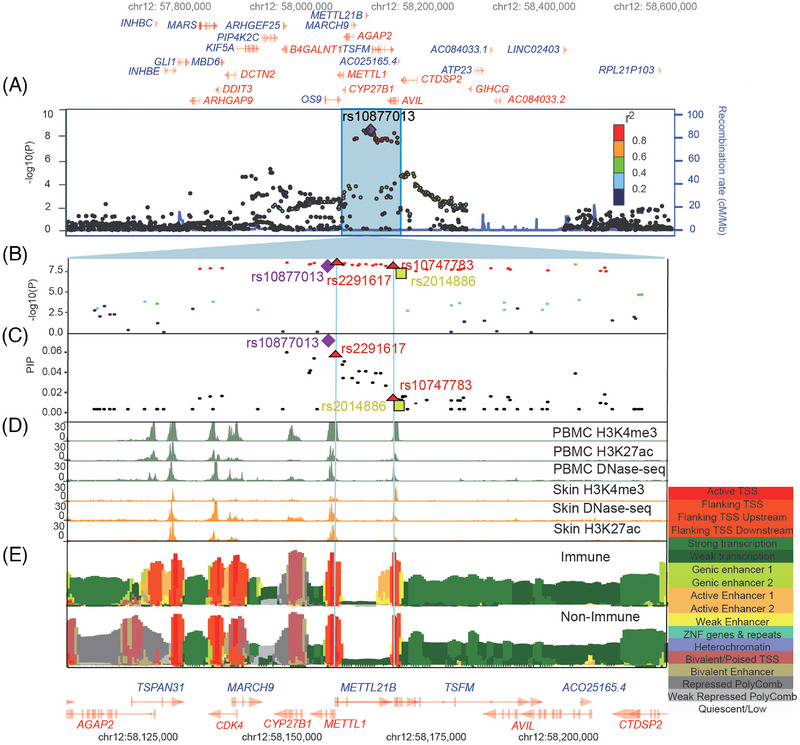
Fine‐mapping analysis of the chr12:57,665,085‐58,665,085 locus. (A) Regional association plot shows the association results (−log10 (*p* value)) using online Locuszoom tool based on ±500 kb of the leading SNP. The leading variant (rs10877013) is represented by a purple diamond. Variants are colored according to their linkage disequilibrium (LD) with the leading variant by using the 1000 genomes data from East Asian groups. (B) A zoomed‐in view of the association results (−log10 (*p* value)) for the credible set region (purple diamond = leading GWAS SNP, yellow square = consensus SNPs, red triangle = SNPs located in the epigenomic peaks). (C) Fine‐mapping analysis results obtained by the susieR package, with the posterior inclusion probability (PIP) that each SNP was causal as the *y*‐axis. (D) Histone marker signals of H3K4me3 ChIP‐seq, H3K27ac ChIP‐seq, and DNase‐seq from peripheral blood mononuclear cells (PBMCs) (green) and skin (orange) from EpiMap and visualized with the UCSC genome browser. (E) The 18‐chromatin state (ChromHMM) annotations (indicated by colors) of 833 samples from EpiMap in immune samples versus nonimmune samples and visualized with the online epilogos search tool. Gene annotations are taken from the epilogos browser.

For the novel leprosy risk locus located on chr12:57,665,085‐58,665,085, the 95% CS comprised 35 variants, including one protein‐relevant variant and two regulatory‐relevant variants (Figures 2A–C and Table S4). The leading SNP rs10877013 (*p* = 1.93 × 10^−9^, OR = 0.85) located in the intronic region of *METTL1* (Figure 2A) was not included in the list of the final credibly causal variants. The SNP rs2014886 located in the intronic region of *TSFM* was predicted by SpliceAI software to affect the splicing of *TSFM* (NM_005726.6) with a splicing probability of 0.82 for donor gain (Figure 1B). Two variants, namely rs2291617 in the 5ʹ‐UTR region of the *METTL21B* gene (also named as *EEF1AKMT3* and *FAM119B*) and rs10747783 in the first exon of *TSFM*, were located in the DNase hypersensitivity peak, H3K4me3 peak, and H3K27ac peak as regulatory‐relevant variants (Figure 2D). Moreover, according to the information from the epigenomic datasets, rs2291617 was located in the transcription start site (TSS) of *METTL21B* and *METTL1*, whereas rs10747783 was located in the TSS of *TSFM*. A comparison of the chromatin state showed almost no difference between immune and nonimmune cells (Figure 2E). Similarly, rs2291617 and rs10747783 showed significant eQTL effects in diverse tissues, including the tibial nerve, whole blood, skin, and brain, for *METTL1*, *TSFM*, *METTL21B*, and *CYP27B1* (Table S5). The GWAS association was colocalized with the eQTL effect on the expression of *TSFM*, *METTL21B*, and *CYP27B1* in both the skin and tibial nerve and *METTL21B* in the blood (PP.H4.abf > 0.8) (Figures 1D–F and Table S6). Therefore, our fine‐mapping and functional annotation analyses implicated SNPs rs2014886, rs2291617, and rs10747783; and genes *METTL1*, *TSFM*, *METTL21B*, and *CYP27B1* as candidate causal variants and genes, respectively, in the locus chr12:57,665,085‐58,665,085.

**FIGURE 3:**
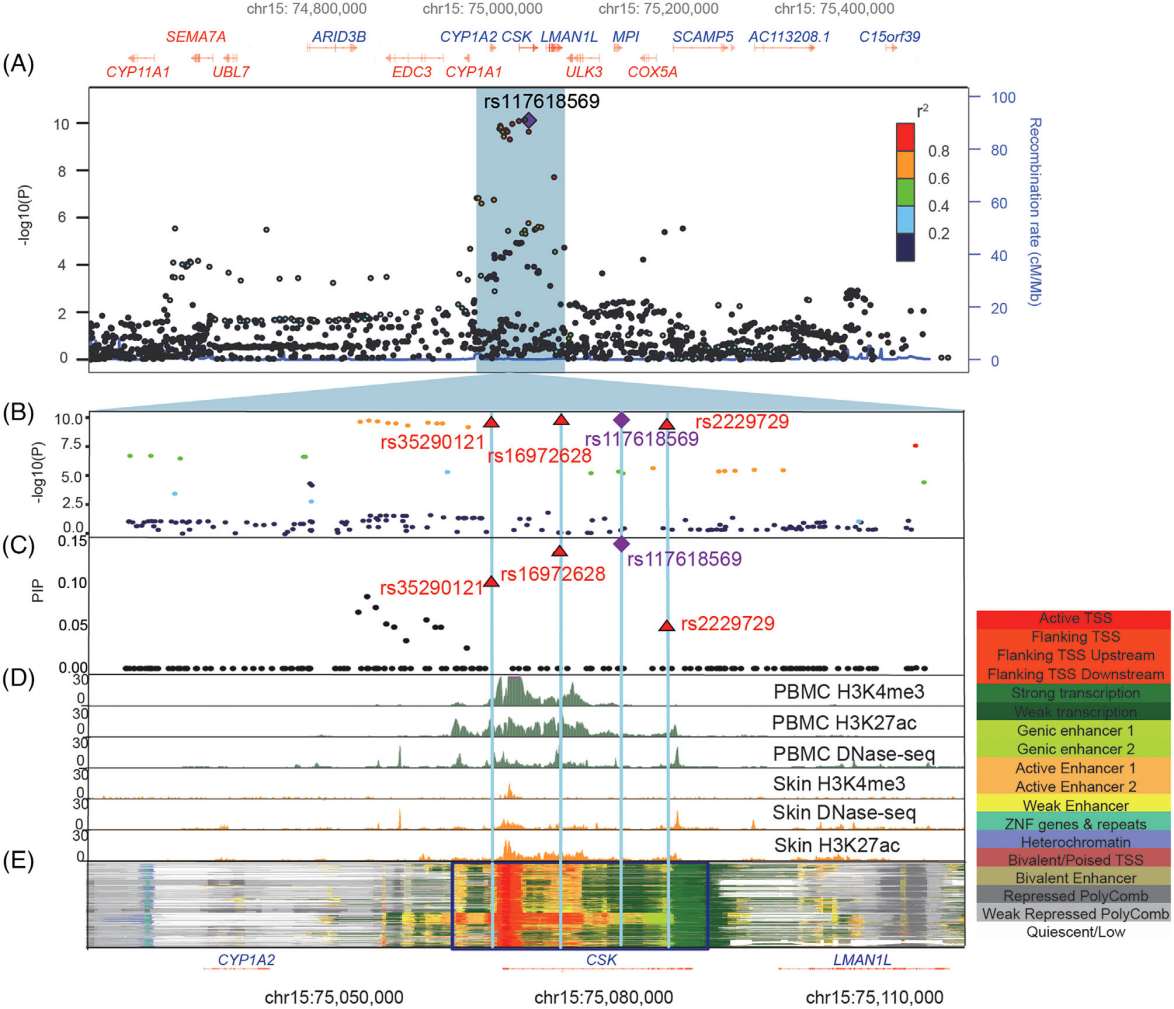
Fine‐mapping analysis of the chr15:74,587,571‐75,587,571 loci. (A) Regional association plot shows the association results (−log10 (*p* value)) using online Locuszoom tool based on ±500 kb of the leading SNP. The leading variant (rs117618569) is represented by a purple diamond. Variants are colored according to their LD with the leading variant by using the 1000 genomes data from East Asian groups. (B) A zoomed‐in view of the association results (−log10 (*p* value)) for the credible set region (purple diamond = leading GWAS SNP, red triangle = SNPs located in the epigenomic peaks). (C) Fine‐mapping analysis results obtained by the susieR package, with the PIP that each SNP was causal as the *y*‐axis. (D) Histone marker signals of H3K4me3 ChIP‐seq, H3K27ac ChIP‐seq, and DNase‐seq from PBMCs (green) and skin (orange) from EpiMap and visualized with the UCSC genome browser. (E) The 18‐chromatin state (ChromHMM) annotations (indicated by colors) of 833 samples from EpiMap and visualized with the online epilogos search tool. Gene annotations are taken from the epilogos browser.

**FIGURE 4:**
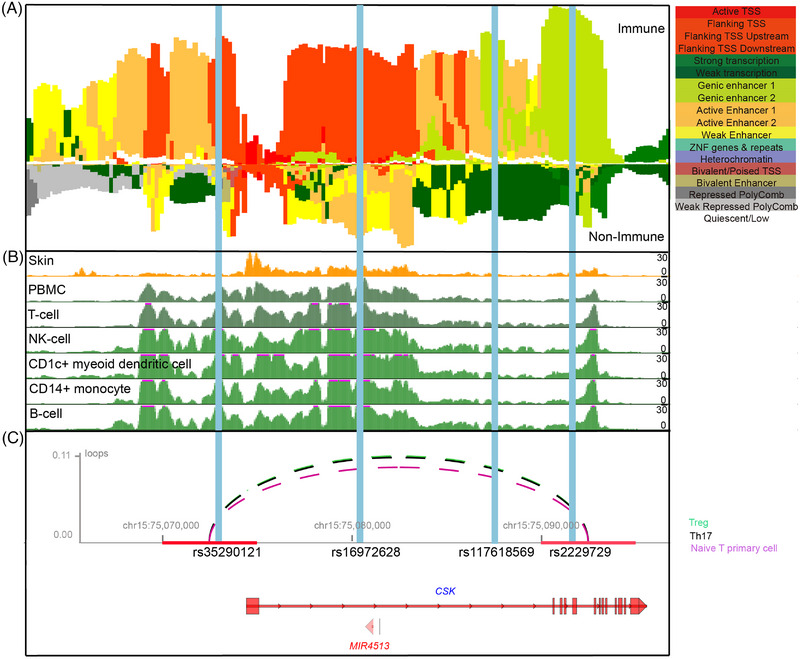
Epigenetic modification of the chr15:74,587,571‐75,587,571 loci. (A) The 18‐chromatin state (ChromHMM) annotations (indicated by colors) of 833 samples from EpiMap in immune samples versus nonimmune samples and visualized with the online epilogos search tool. (B) Histone marker signals of H3K27ac ChIP‐seq from skin, PBMCs, T cells, NK cells, CD1c^+^ myeloid dendritic cells, CD14^+^ monocyte cells, and B cells from EpiMap and visualized with the UCSC genome browser. (C) HiChIP 3D signal enrichment at the enhancer region chr15:75,090,000‐75,095,000 and the CSK TSS region chr15:75,070,000‐75,075,000 in Treg, Th17, and naïve T primary cells. Gene annotations are taken from the epilogos browser.

For the locus chr15:74,587,571‐75,587,571, there were 13 variants in the 95% CS, including four SNPs located in the DNase hypersensitivity peak or histone acetylation or methylation peaks in peripheral blood mononuclear cells (PBMCs): the leading SNP rs117618569 (*p* = 7.07 × 10^−11^, OR = 1.22) and SNP rs16972628, which located in the intronic region of *CSK*, SNP rs2229729, which located in the coding region of *CSK*, and SNP rs35290121, which located in the upstream of *CSK* (Figures 3A–D). The chromatin state of the four SNPs in 833 human samples showed strong heterogeneity across different samples (Figure 3E), particularly in immune and nonimmune samples (Figure 4A). The variants rs35290121 and rs16972628 were almost exclusively present in the flanking TSS upstream region in the immune samples, SNPs rs117618569 and rs2229729 were located in the intergenic enhancer region in immune samples, whereas in the transcription region in nonimmune samples (Figures 4A and B). Based on the information from the HiChIPdb database (a database of HiChIP regulatory interactions), only rs35290121 (SNP in the TSS region) and rs2229729 (SNP in the intergenic enhancer region) were located in the interacting sequences (looping) between chr15:75,070,000‐75,075,000 and chr15:75,090,000‐75,095,000 (Figure 4C). Given the region chr15:75,090,000‐75,095,000 that the SNP rs2229729 located in was an enhancer region, we checked its potential targeted genes and found *CSK* promoter region chr15:75,070,000‐75,075,000 harbored the strongest interaction than other genes (Figure S6). Taken together, these results suggest that SNPs rs35290121 and rs2229729 were candidate causal variants that may alter CSK expression in immune cells.

For the other new loci chr12:110,914,461‐111,914,461, rs12229654 (*p* = 4.36 × 10^−8^, OR = 1.20) were the leading SNP, and its nearest protein‐coding gene was *MYL2* (Table 1). However, we did not find the potential causal variants in this region.

In the known leprosy loci, we found seven protein‐relevant variants with a CADD score of >15 (Figure 1A and Table S4). Four variants located in coding region, including rs2236295 in the first exon of the gene *ADO* (NM_032804.5), rs780668 in the fourth exon of the gene *SLC29A3* (NM_018344.5), and rs181206 and rs1059491 in the *IL27* (NM_145659.3) and *SULT1A2* (NM_177528.2) genes, respectively (Table S7). Three other variants were located in the intronic region or near the genes *SLC29A3, ZNF365*, and *ADO* (Table S4 and S7). Moreover, signals of DNase‐seq, H3K4me3 ChIP‐seq, or H3K27ac ChIP‐seq were observed in skin or PBMCs in SNPs among the previously identified known leprosy loci, including loci that *EGR2*, *TNFSF15*, *RAB32*, and *NOD2* located in (Figures 1C and S7A–D). We also found colocalization in four known loci and eQTL data with PP.H4.abf > 0.8, including *IL18RAP* and *SULT1A2* in skin tissue, *NOD2* in whole blood and the tibial nerve, and *TNFSF15* in whole blood (Figures 1D–F and Table S6).

### 2.3 Gene‐set, tissue, and cell‐type enrichment analyses

Gene‐set analysis was conducted using MAGMA software to delineate disease‐related gene sets and pathways. Three Gene Ontology (GO) gene sets and four Kyoto Encyclopedia of Genes and Genomes (KEGG) terms and one REACTOME term were significantly over‐represented in our GWAS summary data as follows: GOMF_MHC class II receptor activity (*p* = 2.16 × 10^−8^); GOBP_Peptide antigen assembly with MHC class II protein complex (*p* = 7.77 × 10^−7^); GOCC_MHC class II protein complex (*p* = 2.21 × 10^−6^), KEGG_Leishmania infection (*p* = 2.52 × 10^−6^), KEGG_Graft versus host disease (*p* = 3.96 × 10^−6^), KEGG_Allograft rejection (*p* = 7.07 × 10^−5^), KEGG_Autoimmune thyroid disease (*p* = 2.27 × 10^−4^), and REACTOME_PD‐1 signaling (*p* = 1.35 × 10^−5^). We also found that REACTOME_interferon gamma signaling was enriched on the boundary (*p* = 4.00 × 10^−4^). The 10 most significant pathways are shown in Table S8. Tissue‐ and cell‐specific gene expression analysis showed significant results for synovial fluid (−log10 *p* = 2.87) and blood (−log10 *p* = 2.78), where *M. leprae* is detected (Table S9).^28^, ^29^, ^30^ Based on the peaks of the chromatin marker, tissue‐ and cell‐type enrichment analysis suggested that the DNase marker was significantly enriched in primary PBMCs (−log10 *p* = 3.76) and primary natural killer (NK) cells from peripheral blood (−log10 *p* = 2.91), while H3K4me1 and H3K4me3 markers were enriched in PMA‐I‐stimulated primary T helper 17 cells (−log10 *p* = 3.21 and 3.11, respectively) (Table S10). These results highlight the importance of immune‐related tissues, cells, and pathways in the pathogenesis of leprosy.

### 2.4 Phenome‐wide association study identified associations between leprosy and other diseases

Given that pleiotropic effects among leprosy and autoimmunity/inflammatory diseases have been uncovered,^9^, ^10^ to identify other diseases that are likely to be associated with leprosy at the variant level, we conducted a phenome‐wide association study (pheWAS) on each leading SNP in the GWAS Atlas database (https://atlas.ctglab.nl) that includes 4756 GWAS summary statistics. We confirmed the previous observation that the leading SNPs in the *IL12B*, *IL18RAP*, *IL23R*, and *TNFSF15* genes showed discordant effects on the risk between leprosy and inflammatory bowel disease (IBD), Crohn's disease (CD), and ulcerative colitis (UC), whereas the leading SNP in the *LACC1* gene showed concordant effects on the risk between leprosy and IBD and CD. We also found that the leading SNP in the *IL18RAP* locus showed concordant effects on the risk between leprosy and asthma, whereas the leading SNPs in the novel locus chr12:57,665,085‐58,665,085 and the *IL27* locus showed discordant effects on the risk between leprosy and rheumatoid arthritis, multiple sclerosis, IBD, CD, and UC (Table S11).

## 3 DISCUSSION

The identification of genetic variants associated with complex human diseases and traits have provided valuable insights into the complexities of their genetic architecture. Although GWAS represent a powerful tool for investigating the susceptibility loci, most variants identified so far confer relatively small increments in risk and explain only a small proportion of phenotypic variance on the liability scale.^31^, ^32^, ^33^, ^34^ The use of a large sample size to enhance the statistical power of association analysis of variants and the use of samples genotyped using population‐specific arrays and imputed based on population‐specific reference panels can help to identify novel variants.^35^, ^36^ Here, we performed a large GWAS analysis consisting of 7284 leprosy cases and 7738 controls and revealed three novel susceptible loci and 15 previously established locus, further expanding understanding of the genetic landscape underlying leprosy. To improve the fine‐mapping resolution and help prioritization of the subsequent functional studies, we further performed a comprehensive fine‐mapping analysis and 5.4% (19 out of 350) variants were identified as credibly causal variants from the 350 SNPs contained within the 95% CS. These small number of credibly causal variants provide a practical starting point for additional experiments.^37^ Among them, nine variants and 10 genes were highlighted by the software. What is more, the remaining 10 variants and four genes were detected by manual checking of the information from the chromatin interaction database. These results demonstrate the importance of utilizing multiple genomic annotation resources and manual annotation.

In the three newly identified loci, *CSK*, *METTL1*, *METTL21B*, and *CYP27B1* were identified as candidate causal genes. *CSK*, encoding C‐terminal Src kinase, inhibits T cell activation by suppressing the Src family tyrosine kinases Lck and Fyn^38^ and interacting with activated CD28 to mute TCR signaling.^39^ The inhibition of T cell‐activated IFN‐γ immunity is thought to be critical for susceptibility to leprosy and infection by other mycobacteria.^40^ *METTL1*, *METTL21B*, and *CYP27B1* involve in vitamin D‐mediated antibacterial activity, which is a key signaling associated with infection.^41^ *METTL1* and *METTL21B* introns bind to the vitamin D receptor (VDR) to regulate the transcription of *CYP27B1* in response to the parathyroid hormone.^42^ CYP27B1, the 25‐hydroxyvitamin D 1α‐hydroxylase, leads to production of active vitamin D3 at sites of infection, which is critical for the TLR2/1‐induced antimicrobial activity in macrophages infected with *Mycobacterium tuberculosis*.^43^ The causal genes pinpointed in the novel loci further expand our understanding of immune‐relevant mechanisms underlying leprosy based on the genetic basis.

Regarding the known loci, several genes like *ADGB* (*C6orf103*)^7^ and *SNX20* ^6^ located in the linkage disequilibrium region of the top susceptible sites in our previous studies, but they were not suggested as candidate genes due to the deficiency of functional annotation. In the present study, *ADGB*, *SNX20*, *ADO*, and *SULT1A2* were pinpointed as novel causal genes by comprehensive fine‐mapping methods, and some of them were reported to be involved in immune responses. *SNX20*, encoding the sorting nexin 20 protein, plays a crucial role in the immune cell infiltration and regulates various immune molecules in the tumor microenvironment.^44^ We therefore hypothesize that variants of *SNX20* may associated with the immune cell infiltration during the infection of *M. leprae*. *ADO*, encoding cysteamine (2‐aminoethanethiol) dioxygenase, regulates the accumulation of IL‐32,^45^ which is critical for NOD2‐mediated differentiation of monocytes to CD1b^+^ dendritic cells.^46^ The identification of *ADO* further established the key role of NOD2‐medaited signaling pathway in the susceptibility of *M. leprae*.^9^ However, the effects of *ADGB* and *SULT1A2* in infection and immune responses remain to be clarified. The *LRRK2* loci that showed suggestive association with leprosy in our previous study reached genome‐wide significant level in this study.^6^ *LRRK2* gene is highly expressed in immune cells and is biochemically linked with the processes of inflammation, autophagy, and phagocytosis in immune‐related disorders such as Parkinson's disease, IBD, tuberculosis, and leprosy.^47^ Moreover, the known susceptibility genes identified by their reported function of immune regulation previously, like *IL18RAP*, *IL12B*, *NOD2, IL27*, and *TNFSF15*, were also highlighted in the 15 established loci by comprehensive fine‐mapping methods here, which further confirmed the precision of our fine‐mapping strategy and the immune‐associated mechanisms in leprosy, providing a foundation for further functional exploration and interpretation of the pathogenesis.

Tissues and cell type enrichment analysis confirmed the important role of established tissue and immune cell types, including blood, primary PBMCs, NK cells, and PMA‐I‐stimulated primary T helper 17 cells.^3^, ^48^ Intriguingly, tissue‐ and cell‐specific gene expression analysis showed significant results in synovial fluid, which is consistent with that acid‐fast bacilli were found within the synovial fluid of the patients with erythema nodosum leprosum or leprosy‐related chronic arthritis.^28^, ^29^ Moreover, we first clarified the PD‐1 signaling and further confirmed the IFN‐γ signaling^10^, ^49^ by gene‐set pathway analysis. PD‐1 signaling, enriched by the susceptibility genes of *CSK* and *HLA*s, is upregulated by chronic infection to inactivate TCR signaling by recruiting tyrosine kinase CSK, leading to the exhaustion of T cells and inhibition of the T cell‐ activated IFN‐γ immunity.^50^

**FIGURE 5:**
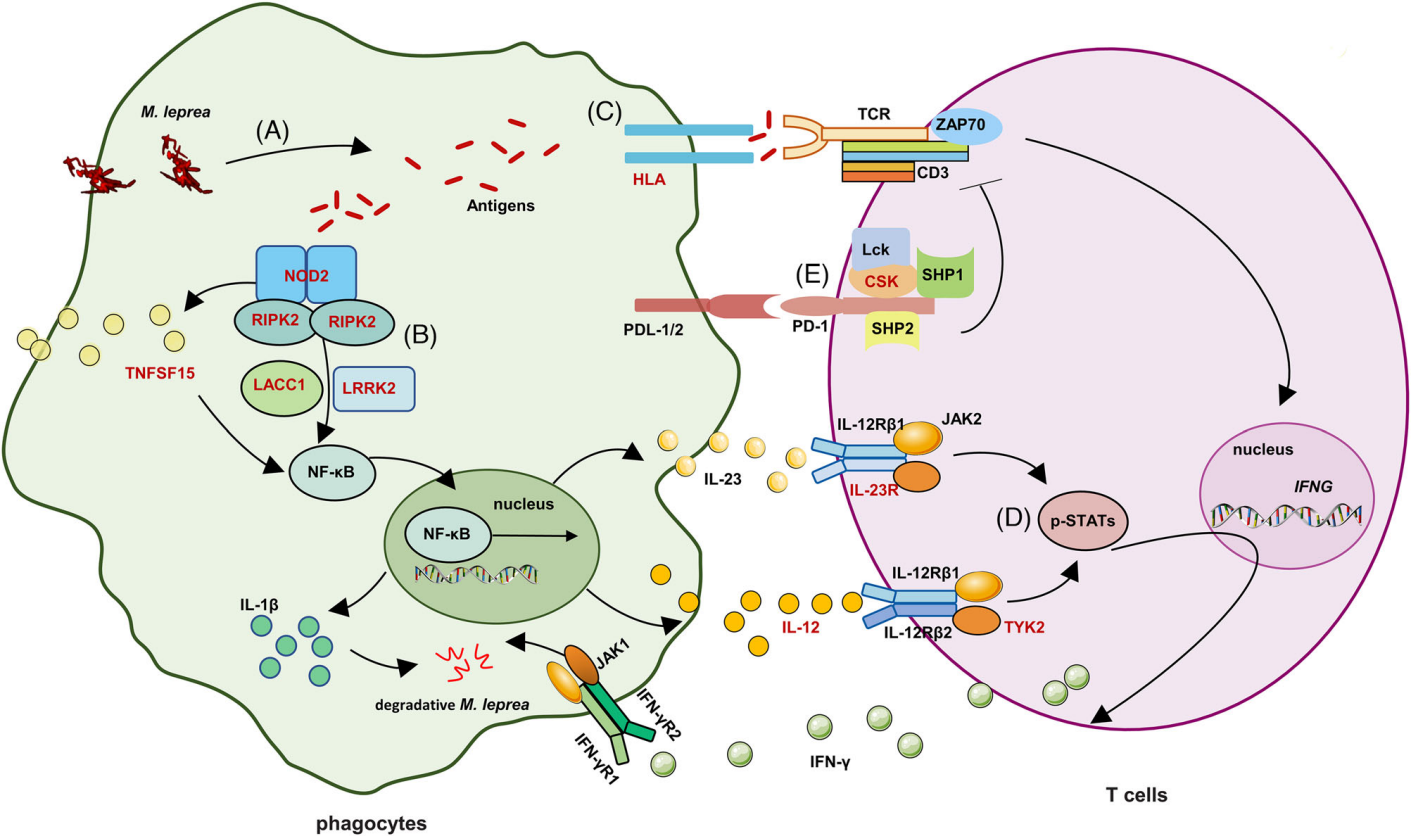
Pathways associated with known leprosy susceptibility genes. (A) *M. leprae* is recognized and digested into antigens (e.g., muramyl dipeptide) by phagocytes. (B) NOD2 senses the muramyl dipeptide and recruits RIPK2 to release antibacterial cytokines such as IL‐1β, IL‐12 and IL‐23 by NF‐κB, which is mediated by TNFSF15, LACC1, and LRRK2. (C) The antigens of *M. leprae* are presented by HLA to activate T cells. (D) Simultaneously, secretion of IL‐12 and IL‐23 activates T cells to promote the IFN‐γ‐mediated antimicrobial response through the JAK/STAT signaling pathway. (E) Chronic *M. leprae* infection upregulates the expression of the inhibitory immune receptor PD‐1, which recruits the tyrosine kinase CSK to inactivate TCR signaling and inhibit T cell‐mediated antimicrobial response. Proteins encoded by leprosy susceptibility genes are depicted in red, and the abnormality of these molecules may interferes with the synergistic antimicrobial response between phagocytes and T cells. TCR, T cell receptor; p‐STAT, phosphorylated STAT.

To systematically illustrate the molecular mechanisms underlying leprosy, on the basis of the functional annotation of previously identified causal genes including *NOD2*, *RIPK2*, *TNFSF15*, *LACC1*, *IL12B*, *HLA‐DR*, *IL23R, LRRK2*, and *TYK2* ^6^, ^10^, ^13^, ^18^ and the newly pinpointed candidate gene *CSK*, we speculated that these variants may interfere with the recognition, presentation, and clearance of *M. leprae*. Upon the recognition of *M. leprae* by phagocytes, the NOD2 recruits RIPK2 to release antibacterial cytokines such as IL‐1β, IL‐12, and IL‐23 by activating nuclear factor‐kappa B (NF‐κB),^6^, ^51^ which is mediated by TNF superfamily member 15 (TNFSF15), laccase domain‐containing 1 (LACC1), and LRRK2.^52^, ^53^, ^54^ Subsequently, antigen presentation by HLA^55^ and cytokine (IL‐12 and IL‐23) secretion activate T cells to promote the IFN‐γ‐mediated antimicrobial response through the JAK/STAT signaling pathway.^40^, ^56^ Chronic *M. leprae* infection upregulates the expression of the inhibitory immune receptor PD‐1, which recruits the tyrosine kinase CSK^50^ and inactivates TCR signaling, resulting in the exhaustion of T cells and inhibition of the T cell‐mediated antimicrobial response (Figure 5). Thus, the leprosy susceptibility loci, including *NOD2*, *RIPK2*, *TNFSF15*, *LACC1*, *LRRK2*, *IL12B*, and *HLA‐DR* in phagocytes and *IL23R, TYK2*, and *CSK* in T cells, may interfere with the synergistic antimicrobial response between phagocytes and T cells. Further functional studies are required to investigate the roles of these causal genes in leprosy.

The pleiotropic effects between leprosy and autoimmunity/inflammatory diseases have always been focused,^57^ and some shared mechanisms of pathogenic recognition and response among them have been reported.^9^ To further fully elucidate the pleiotropic effects of the causal variants, pheWAS was applied and confirmed the significant genetic overlap among leprosy, inflammatory diseases (IBD, CD and UC), and autoimmune diseases (rheumatoid arthritis, multiple sclerosis, and asthma). The concordant or discordant effects of the leading SNPs in the loci (*IL12B*, *IL18RAP*, *IL23R*, *TNFSF15*, *LACC1*, *METTL1*, and *IL27*) were identified in the present study, which further highlighted the involvement of immune responses and the shared genetic fingerprint of infectious diseases and autoimmune/inflammatory diseases. Intriguingly, we newly discovered that the leading SNP in the *IL18RAP* locus showed concordant effects on the risk between leprosy and asthma. Asthma is a Th2 cell‐mediated type 2 inflammation, lepromatous leprosy also characterized by the enrichment of Th2 cells and cytokines,^58^, ^59^ wherein IL‐18 activates Th2 cells to produce IL‐4 and IL‐13 in the absence of IL‐12 or IL‐15.^60^ The concordant effect shows that the variant rs17027258 in the *IL18RAP* locus in leprosy shares a similar mechanism of Th2‐mediated responses in asthma.

We acknowledged the limitation. Given that the eQTL datasets were not specific to the Asian/Chinese population, some east‐Asian or Chinese‐specific risk allele (e.g., rs2229729 and rs12229654) does not exist in the eQTL datasets, making it difficult to annotate the regulatory consequences of those risk locus. What is more, some causal variants may affect protein levels through their effects on translation or protein stability without any effects on mRNA levels. Use of the population‐specific eQTL dataset and protein quantitative trait loci is warranted to prioritize more causal variants and genes.

Collectively, our current study with a large sample size identified three novel loci and 15 previously established loci. The biological annotation of the fine‐mapped causal variants and genes highlight the importance of immune responses in the pathogenesis of leprosy. This work does not only expand our understanding of inherited variation and further characterizes the genetic architecture of leprosy, but also increases the power to construct a GWAS‐derived risk prediction model, which will facilitate the identification of genetically susceptible persons of leprosy patients and the implementation of post exposure prophylaxis. Subsequent functional studies should be conducted to explore the biological mechanisms underlying leprosy disease risk.

## 4 MATERIALS AND METHODS

### 4.1 Study subjects

Six GWAS datasets were included in the initial meta‐analysis: four published GWASs (GWAS1−4) and two new GWASs (GWAS5 and GWAS6), resulting in a final sample size of 7284 patients with leprosy and 7738 healthy controls (Table S1).

GWAS1 included 706 leprosy patients and 1223 healthy controls genotyped using the Illumina Human 610K‐Quad BeadChips; all the patients and controls were from northern China and of Han Chinese descent as described in our previous study.^6^

GWAS2 included 374 patients with leprosy and 510 healthy controls of Han Chinese descent in northern China. All participants were genotyped using the Illumina Human 660K‐Quad BeadChips as described in our previous study.^9^

GWAS3 and GWAS4 included 864 cases and 853 controls of Han Chinese descent from northern China and 333 cases and 573 controls of Han Chinese descent from southern China, respectively. All participants were genotyped using the Illumina Omni Zhonghua Array as described previously.^11^

GWAS5 and GWAS6 are two new GWAS datasets comprising 3298 leprosy patients and 3272 healthy controls from northern China and 2302 leprosy patients and 1632 controls from southern China, respectively, all of which were of Han Chinese descent. These new samples were genotyped using the Illumina Asian Screening Array BeadChip.

The cases and controls were recruited based on the criteria reported in our previous studies and were matched by ancestry and geographical area.^6^, ^7^, ^8^, ^9^, ^10^, ^11^ This study was approved by the institutional review board (IRB) committees at the Shandong Provincial Institute of Dermatology and Venereology, Shandong Academy of Medical Science (approval number 2013‐KYKT‐22).

### 4.2 QC procedures for GWAS

The QC procedures for the GWAS1−4 datasets have been described previously.^6^, ^9^, ^11^ The SNPs of GWAS5 and GWAS6 were separately quality controlled on the basis of the following criteria: (1) all of the SNPs on the X, Y, and mitochondrial chromosomes; intensity‐only SNPs, and copy number variations were excluded; as were (2) all the SNPs with an MAF < 1% or call rate < 95% in all cases and controls and SNPs with a *p* value of the Hardy‐Weinberg equilibrium (HWE) < 1.0 × 10^−8^ in controls.

Next, samples were removed with a call rate of <96%, one of the duplicated or related individuals (first‐, second‐, or third‐degree familial relationships) with a lower call rate, and population outliers based on the PC analysis method.

Finally, 474,857 SNPs in 6101 samples (2991 cases and 3110 controls) of GWAS5 and 477,111 SNPs in 3485 samples (2016 cases and 1469 controls) of GWAS6 passed the SNP and sample QC filters. A total of 7284 cases and 7738 controls in the six GWAS datasets that passed the QC procedure were used for imputation and genome‐wide association analysis. Table S1 provides details on the sample size and other information of each dataset.

### 4.3 Imputation using the ChinaMAP reference panel

PLINK v2.00a2LM (https://www.cog‐genomics.org/plink/2.0/) was used to convert the genotype files from plink format to vcf‐4.2 format based on the human reference genome (UCSC Genome Browser hg19). The LiftoverVcf command of software gatk‐4.0.12.0^61^ was used to lift over the vcf files from UCSC Genome Browser reference build hg19 to hg38. bcftools (Version: 1.2, http://samtools.github.io/bcftools/bcftools.html) was used to create a separate vcf file for each chromosome. The ChinaMAP reference panel that includes 10,155 unrelated Chinese individuals can achieve high‐quality imputation for genetic studies of Chinese populations, and it was used as a reference panel. The imputation was performed on the ChinaMAP imputation server by uploading all genotype vcf files to the ChinaMAP browser (www.mBiobank.com).

The imputed SNPs were included in the subsequent association analysis to confirm whether they were well imputed (*r* ^2^ > 0.8) and had an MAF ≥ 1% in all samples and SNPs without significant deviation from the HWE in the controls (*p* > 1.0 × 10^−5^). Finally, 5.88 to 6.43 million qualified variants were retained in the individual GWAS dataset, and 5,276,914 SNPs that were common across the studies were used in the genetic association analysis.

### 4.4 Statistical analysis

The single SNP association analysis was performed separately for each GWAS dataset in SNPTEST software (v2.4.1, https://www.well.ox.ac.uk/~gav/snptest/) by using the frequentist association test of an additive model. For each dataset, significant PCs based on EigenCorr2 method calculated by EigenCorr^62^ were included as covariates in the association model to account for population stratification. PC1, PC7, and PC10 were included as covariates in the association model for GWAS5. PC1, PC3, and PC13 were included as covariates in the association model for GWAS6. The PCs that were included as covariates in the association model for GWAS1–4 were as same as described previously.^6^, ^9^, ^11^

The meta‐analysis of the six GWAS datasets based on an inverse variance‐weighted fixed‐effects model was conducted using META (v1.7).^63^ Manhattan plot and Q–Q plot were generated for the meta‐analysis by using R software (v.3.4.3). Regional plots were generated using the online LocusZoom tool (http://locuszoom.sph.umich.edu/).

Conditional logistic regression was performed at the top signal at each identified locus by SNPTEST software to assess whether an additional independent association existed. Loci with a leading SNP whose *p* value was less than 5 × 10^−8^ were considered significant. The threshold of *p* < 1 × 10^−6^ was used to identify secondary signals at each significant locus. Heterogeneity across independent datasets was assessed by determining the *p* values from Cochran's *Q* statistics, and *p* < 0.01 was considered significant for heterogeneity.

### 4.5 Colocalization with eQTLs

We tested whether the 20 independent loci of leprosy coincided with the cis‐eQTLs of candidate genes in different tissues. The cis‐eQTLs of three tissues (whole blood, skin, and tibial nerve) were retrieved from GTEx v7.^64^ Coincidence of each signal was tested by pairwise colocalization analysis of the loci using the Coloc package (v5.1.0) of R software.^65^ Loci were defined by a ±500 kb window around the respective leading SNPs. For multiple independently segregating SNPs in the flanking 1 Mb regions, loci were expanded to the border of the ±500 kb window around the respective signals. PP.H4.abf value shows the posterior probability of whether a shared causal variant exists in each pair of signals. SNP.PP.H4 value shows the posterior probability of each SNP was causal variant in each pair of signals. PP.H4.abf > 0.8 and SNP.PP.H4 > 0.8 were considered as successful colocalizations in one gene and in one SNP, respectively.

### 4.6 Fine‐mapping analysis to identify credibly causal variants

The susieR package of R software (https://github.com/stephenslab/susieR) was used to identify all potential causal variants for each independent locus associated with leprosy. The imputed genotype datasets were used, and variants were extracted using a 500 kb window around the leading SNP for each locus. For the susieR method, *L*, which indicates the (maximum) number of causal SNPs, was set based on the conditional analysis results, where one or two causal SNPs can be assumed. The scaled prior variance was set as the default. Next, 95% CS of SNPs containing a potential causal variant within a locus were generated. The SNP with the posterior inclusion probability (PIP) within each CS was also identified. The CADD score was used to identify variants with high predicted deleteriousness, and a CADD PHRED score of >15 indicated potential deleteriousness.^66^ We used scores from SpliceAI (https://spliceailookup.broadinstitute.org/), a deep‐learning method that predicts the effects of variants on splicing. A variant with a SpliceAI score of >0.8 was defined as deleterious.

The ChIP‐seq peaks of H3K4me3 and H3K27ac and the DNase‐seq data of PBMCs and skin samples were acquired from the EpiMap database.^67^ We then visualized the sequencing data by using the UCSC Browser (http://genome.ucsc.edu/). The Epilogos visualization model (https://epilogos.altius.org/) based on the epigenomic datasets across 833 biosamples was used to analyze the 18‐chromatin states. To further characterize the functional relevance of looping interactions in the enhancer region, we evaluated the H3K27ac data based on the 5k bin of Treg, Th17, and naive T primary cells in HiChIPdb, a database of HiChIP regulatory interactions.^68^ All annotations were based on the human reference genome GRCh37.

### 4.7 Gene‐set, tissue, and cell‐type enrichment analyses

Gene‐set analysis was performed using MAGMA software (v1.09b, https://ctg.cncr.nl/software/magma). We downloaded gene sets from the MSigDB database v7.5.1 (http://software.broadinstitute.org/gsea/msigdb/). A total of 10,413 GO terms and 186 KEGG and 1615 Reactome pathway gene sets were detected. The following significance thresholds of *p* values were used for these three categories following Bonferroni correction: <4.80 × 10^−6^, 2.69 × 10^−4^, and 3.10 × 10^−5^, respectively.

The LDSC‐seg method in LDSC software (v1.0.1) was used to identify disease‐relevant tissues and cell types by analyzing the gene expression data and the chromatin marker data together with our GWAS summary statistics.^26^ The gene expression data contained 152 tissues or cell types from Franke's laboratory, and 489 tissue‐specific chromatin‐based annotations from peaks for six epigenetic markers were downloaded from https://alkesgroup.broadinstitute.org/LDSCORE/LDSC_SEG_ldscores. The cutoff values of –log10 (*p* value) = 2.75 for the gene expression data and–log10 (*p* value) = 2.85 for chromatin‐based annotations were considered significant.^26^

### 4.8 Heritability analysis and pheWAS

For the heritability analysis, we estimated the variance explained by the genome‐wide significant SNPs by using the GCTA software.^69^ The genetic relationship matrix from all the autosomal SNPs was determined using all genotyped SNPs with an MAF value > 0.01 in the combined GWASs with correction for the first 10 genetic PCs. We used the default option (restricted maximum likelihood) to fit the appropriate variance component model. We assumed the leprosy prevalence of 0.0001 to estimate the heritability on the liability scale.

To identify other diseases/phenotypes associated with leprosy at the variant level, we performed a pheWAS on each leading SNP in the GWAS Atlas (https://atlas.ctglab.nl). According to the GWAS Atlas that included 4756 traits, a *p* value of <1.05 × 10^−5^ was considered significant. All diseases/phenotypes from the GWAS Atlas associated with the 20 leading SNPs are shown in Table S11.

## AUTHOR CONTRIBUTION

F. R. Z. conceived of the study and obtained financial support. F. R. Z. and H. L. designed the study. P. C. H., T. S. C., D. C. L., L. L. S., X. A. F., Y. H. S., H. L. W., N. W., and C. W. collected the samples. G. Q. Y., H. L. W., and N. W. responded to sample preparation.

Z. Z. W., and W. C. L. analyzed the genotyping data and performed all the statistical analysis. Z. Z. W., T. T. L., Z. H. M., C. W., and X. J. L. performed bioinformatics. T. T. L., Z. Z. W., and W. C. L. wrote the draft and H. L., J. J. L., and F. R. Z. revised it. All authors have read and approved the final manuscript.

## CONFLICT OF INTEREST STATEMENT

The authors declare no conflict of interests.

## ETHICS STATEMENT

This study was approved by the IRB committees at the Shandong Provincial Institute of Dermatology and Venereology, Shandong Academy of Medical Science (approval number 2013‐KYKT‐22). All participants provided written informed consent.

## Supporting information

Supporting Information

## Data Availability

The genome‐wide association meta‐analysis results of each SNP have been deposited in the OMIX, China National Center for Bioinformation/Beijing Institute of Genomics, Chinese Academy of Sciences (https://ngdc.cncb.ac.cn/omix: accession no.OMIX002870). Other database required for colocalization, gene‐set, tissue, and cell‐type enrichment analyses are available in the Materials and Methods part.

## References

[1] Morens DM , Folkers GK , Fauci AS . The challenge of emerging and re‐emerging infectious diseases. Nature. 2004;430(6996):242‐249. 15241422 10.1038/nature02759 PMC7094993

[2] Chen JX , Han YS , Zhang SQ , et al. Novel therapeutic evaluation biomarkers of lipid metabolism targets in uncomplicated pulmonary tuberculosis patients. Signal Transduct Target Ther. 2021;6(1):22. 33462176 10.1038/s41392-020-00427-w PMC7814055

[3] Mi Z , Liu H , Zhang F . Advances in the immunology and genetics of leprosy. Front Immunol. 2020;11:567. 32373110 10.3389/fimmu.2020.00567 PMC7176874

[4] Mi Z , Wang Z , Xue X , et al. The immune‐suppressive landscape in lepromatous leprosy revealed by single‐cell RNA sequencing. Cell Discov. 2022;8(1):2. 35013182 10.1038/s41421-021-00353-3 PMC8748782

[5] Mozzi A , Pontremoli C , Sironi M . Genetic susceptibility to infectious diseases: current status and future perspectives from genome‐wide approaches. Infect Genet Evol. 2018;66:286‐307. 28951201 10.1016/j.meegid.2017.09.028 PMC7106304

[6] Zhang FR , Huang W , Chen SM , et al. Genomewide association study of leprosy. N Engl J Med. 2009;361(27):2609‐2618. 20018961 10.1056/NEJMoa0903753

[7] Zhang F , Liu H , Chen S , et al. Identification of two new loci at IL23R and RAB32 that influence susceptibility to leprosy. Nat Genet. 2011;43(12):1247‐1251. 22019778 10.1038/ng.973

[8] Liu H , Bao F , Irwanto A , et al. An association study of TOLL and CARD with leprosy susceptibility in Chinese population. Hum Mol Genet. 2013;22(21):4430‐4437. 23784377 10.1093/hmg/ddt286 PMC3792694

[9] Liu H , Irwanto A , Fu X , et al. Discovery of six new susceptibility loci and analysis of pleiotropic effects in leprosy. Nat Genet. 2015;47(3):267‐271. 25642632 10.1038/ng.3212

[10] Liu H , Irwanto A , Tian H , et al. Identification of IL18RAP/IL18R1 and IL12B as leprosy risk genes demonstrates shared pathogenesis between inflammation and infectious diseases. Am J Hum Genet. 2012;91(5):935‐941. 23103228 10.1016/j.ajhg.2012.09.010 PMC3487119

[11] Wang Z , Sun Y , Fu X , et al. A large‐scale genome‐wide association and meta‐analysis identified four novel susceptibility loci for leprosy. Nat Commun. 2016;7:13760. 27976721 10.1038/ncomms13760 PMC5172377

[12] Wang Z , Mi Z , Wang H , et al. Discovery of 4 exonic and 1 intergenic novel susceptibility loci for leprosy. Clin Genet. 2018;94(2):259‐263. 29722023 10.1111/cge.13376

[13] Liu H , Wang Z , Li Y , et al. Genome‐wide analysis of protein‐coding variants in leprosy. J Invest Dermatol. 2017;137(12):2544‐2551. 28842327 10.1016/j.jid.2017.08.004

[14] Wong SH , Gochhait S , Malhotra D , et al. Leprosy and the adaptation of human toll‐like receptor 1. PLoS Pathog. 2010;6:e1000979. 20617178 10.1371/journal.ppat.1000979 PMC2895660

[15] Shields ED , Russell DA , Pericak‐Vance MA . Genetic epidemiology of the susceptibility to leprosy. J Clin Invest. 1987;79(4):1139‐1143. 3549780 10.1172/JCI112930 PMC424295

[16] Farh KK , Marson A , Zhu J , et al. Genetic and epigenetic fine mapping of causal autoimmune disease variants. Nature. 2015;518(7539):337‐343. 25363779 10.1038/nature13835 PMC4336207

[17] Fava VM , Dallmann‐Sauer M , Orlova M , et al. Deep resequencing identifies candidate functional genes in leprosy GWAS loci. PLoS Negl Trop Dis. 2021;15(12):e0010029. 34879060 10.1371/journal.pntd.0010029 PMC8687567

[18] Wang D , Fan Y , Malhi M , et al. Missense variants in HIF1A and LACC1 contribute to leprosy risk in Han Chinese. Am J Hum Genet. 2018;102(5):794‐805. 29706348 10.1016/j.ajhg.2018.03.006 PMC5986702

[19] Caliskan M , Brown CD , Maranville JC . A catalog of GWAS fine‐mapping efforts in autoimmune disease. Am J Hum Genet. 2021;108(4):549‐563. 33798443 10.1016/j.ajhg.2021.03.009 PMC8059376

[20] Spain SL , Barrett JC . Strategies for fine‐mapping complex traits. Hum Mol Genet. 2015;24(R1):R111‐119. 26157023 10.1093/hmg/ddv260 PMC4572002

[21] Schwartzentruber J , Cooper S , Liu JZ , et al. Genome‐wide meta‐analysis, fine‐mapping and integrative prioritization implicate new Alzheimer's disease risk genes. Nat Genet. 2021;53(3):392‐402. 33589840 10.1038/s41588-020-00776-w PMC7610386

[22] Ahmed WU , Kleeman S , Ng M , et al. Genome‐wide association analysis and replication in 810,625 individuals with varicose veins. Nat Commun. 2022;13(1):3065. 35654884 10.1038/s41467-022-30765-y PMC9163161

[23] Mitchell BL , Saklatvala JR , Dand N , et al. Genome‐wide association meta‐analysis identifies 29 new acne susceptibility loci. Nat Commun. 2022;13(1):702. 35132056 10.1038/s41467-022-28252-5 PMC8821634

[24] Corces MR , Shcherbina A , Kundu S , et al. Single‐cell epigenomic analyses implicate candidate causal variants at inherited risk loci for Alzheimer's and Parkinson's diseases. Nat Genet. 2020;52(11):1158‐1168. 33106633 10.1038/s41588-020-00721-x PMC7606627

[25] Wei WQ , Sun H , Chen YJ , et al. Genetic identification of tissues and cell types underlying attention‐deficit/hyperactivity disorder. Front Psychiatry. 2022;13:999007. 36090352 10.3389/fpsyt.2022.999007 PMC9458853

[26] Finucane HK , Reshef YA , Anttila V , et al. Heritability enrichment of specifically expressed genes identifies disease‐relevant tissues and cell types. Nat Genet. 2018;50(4):621‐629. 29632380 10.1038/s41588-018-0081-4 PMC5896795

[27] Li L , Huang P , Sun X , et al. The ChinaMAP reference panel for the accurate genotype imputation in Chinese populations. Cell Res. 2021;31(12):1308‐1310. 34489580 10.1038/s41422-021-00564-z PMC8648815

[28] Louie JS , Kornasky JR , Cohen AH . Lepra cells in synovial fluid of a patient with erythema nodosum leprosum. N Engl J Med. 1973;289(26):1410‐1411. 4585102 10.1056/NEJM197312272892608

[29] Gunawan H , Wulansari Y , Achdiat PA , Hindritiani R . Leprosy‐related chronic arthritis with an abundance of Mycobacterium leprae in the synovial fluid: a rare case. Int Med Case Rep J. 2021;14:371‐376. 34113179 10.2147/IMCRJ.S312355 PMC8184239

[30] Reis EM , Araujo S , Lobato J , et al. Mycobacterium leprae DNA in peripheral blood may indicate a bacilli migration route and high‐risk for leprosy onset. Clin Microbiol Infect. 2014;20(5):447‐452. 24033793 10.1111/1469-0691.12349

[31] So HC , Gui AH , Cherny SS , Sham PC . Evaluating the heritability explained by known susceptibility variants: a survey of ten complex diseases. Genet Epidemiol. 2011;35(5):310‐317. 21374718 10.1002/gepi.20579

[32] Nazarian A , Kulminski AM . Evaluation of the genetic variance of Alzheimer's Disease explained by the disease‐associated chromosomal regions. J Alzheimers Dis. 2019;70(3):907‐915. 31282417 10.3233/JAD-190168 PMC7243481

[33] Zaitlen N , Kraft P . Heritability in the genome‐wide association era. Hum Genet. 2012;131(10):1655‐1664. 22821350 10.1007/s00439-012-1199-6 PMC3432754

[34] Jung S , Park D , Lee HS , et al. Identification of shared loci associated with both Crohn's disease and leprosy in east Asians. Hum Mol Genet. 2022;31(22):3934‐3944. 35512355 10.1093/hmg/ddac101

[35] Watanabe K , Jansen PR , Savage JE , et al. Genome‐wide meta‐analysis of insomnia prioritizes genes associated with metabolic and psychiatric pathways. Nat Genet. 2022;54(8):1125‐1132. 35835914 10.1038/s41588-022-01124-w

[36] Sazonovs A , Stevens CR , Venkataraman GR , et al. Large‐scale sequencing identifies multiple genes and rare variants associated with Crohn's disease susceptibility. Nat Genet. 2022;54(9):1275‐1283. 36038634 10.1038/s41588-022-01156-2 PMC9700438

[37] Stanzick KJ , Li Y , Schlosser P , et al. Discovery and prioritization of variants and genes for kidney function in >1.2 million individuals. Nat Commun. 2021;12(1):4350. 34272381 10.1038/s41467-021-24491-0 PMC8285412

[38] Sitaram P , Uyemura B , Malarkannan S , Riese MJ . Beyond the cell surface: targeting intracellular negative regulators to enhance T cell anti‐tumor activity. Int J Mol Sci. 2019;20(23):5821. 31756921 10.3390/ijms20235821 PMC6929154

[39] Skanland SS , Tasken K . Carboxyl‐terminal Src kinase binds CD28 upon activation and mutes downstream signaling. J Immunol. 2019;203(4):1055‐1063. 31292214 10.4049/jimmunol.1801660

[40] Bustamante J , Boisson‐Dupuis S , Abel L , Casanova JL . Mendelian susceptibility to mycobacterial disease: genetic, immunological, and clinical features of inborn errors of IFN‐gamma immunity. Semin Immunol. 2014;26(6):454‐470. 25453225 10.1016/j.smim.2014.09.008 PMC4357480

[41] Ismailova A , White JH . Vitamin D, infections and immunity. Rev Endocr Metab Disord. 2022;23(2):265‐277. 34322844 10.1007/s11154-021-09679-5 PMC8318777

[42] Meyer MB , Benkusky NA , Kaufmann M , et al. A kidney‐specific genetic control module in mice governs endocrine regulation of the cytochrome P450 gene Cyp27b1 essential for vitamin D3 activation. J Biol Chem. 2017;292(42):17541‐17558. 28808057 10.1074/jbc.M117.806901 PMC5655528

[43] Liu PT , Stenger S , Li H , et al. Toll‐like receptor triggering of a vitamin D‐mediated human antimicrobial response. Science. 2006;311(5768):1770‐1773. 16497887 10.1126/science.1123933

[44] Wu GJ , Ren K , He M , et al. SNX20 expression correlates with immune cell infiltration and can predict prognosis in lung adenocarcinoma. Int J Gen Med. 2021;14:7599‐7611. 34764676 10.2147/IJGM.S337198 PMC8575493

[45] Masson N , Keeley TP , Giuntoli B , et al. Conserved N‐terminal cysteine dioxygenases transduce responses to hypoxia in animals and plants. Science. 2019;365(6448):65‐69. 31273118 10.1126/science.aaw0112 PMC6715447

[46] Schenk M , Krutzik SR , Sieling PA , et al. NOD2 triggers an interleukin‐32‐dependent human dendritic cell program in leprosy. Nat Med. 2012;18(4):555‐563. 22447076 10.1038/nm.2650 PMC3348859

[47] Wallings RL , Tansey MG . LRRK2 regulation of immune‐pathways and inflammatory disease. Biochem Soc Trans. 2019;47(6):1581‐1595. 31769472 10.1042/BST20180463 PMC6925522

[48] Converse PJ , Bjune G . Natural killer (NK) cell activity and reversal reaction in leprosy. Int J Lepr Other Mycobact Dis. 1986;54(4):503‐509. 3819526

[49] Manry J , Nedelec Y , Fava VM , et al. Deciphering the genetic control of gene expression following Mycobacterium leprae antigen stimulation. PLoS Genet. 2017;13(8):e1006952. 28793313 10.1371/journal.pgen.1006952 PMC5565194

[50] Sheppard KA , Fitz LJ , Lee JM , et al. PD‐1 inhibits T‐cell receptor induced phosphorylation of the ZAP70/CD3zeta signalosome and downstream signaling to PKCtheta. FEBS Lett. 2004;574(1‐3):37‐41. 15358536 10.1016/j.febslet.2004.07.083

[51] Kobayashi KS , Chamaillard M , Ogura Y , et al. Nod2‐dependent regulation of innate and adaptive immunity in the intestinal tract. Science. 2005;307(5710):731‐734. 15692051 10.1126/science.1104911

[52] Sun R , Hedl M , Abraham C . TNFSF15 promotes antimicrobial pathways in human macrophages and these are modulated by TNFSF15 disease‐risk variants. Cell Mol Gastroenterol Hepatol. 2021;11(1):249‐272. 32827707 10.1016/j.jcmgh.2020.08.003 PMC7689184

[53] Lahiri A , Hedl M , Yan J , Abraham C . Human LACC1 increases innate receptor‐induced responses and a LACC1 disease‐risk variant modulates these outcomes. Nat Commun. 2017;8:15614. 28593945 10.1038/ncomms15614 PMC5472760

[54] Yan R , Liu Z . LRRK2 enhances Nod1/2‐mediated inflammatory cytokine production by promoting Rip2 phosphorylation. Protein Cell. 2017;8(1):55‐66. 27830463 10.1007/s13238-016-0326-x PMC5233611

[55] Ottenhoff TH , Neuteboom S , Elferink DG , de Vries RR . Molecular localization and polymorphism of HLA class II restriction determinants defined by Mycobacterium leprae‐reactive helper T cell clones from leprosy patients. J Exp Med. 1986;164(6):1923‐1939. 2431092 10.1084/jem.164.6.1923 PMC2188487

[56] Martinez‐Barricarte R , Markle JG , Ma CS , et al. Human IFN‐gamma immunity to mycobacteria is governed by both IL‐12 and IL‐23. Sci Immunol. 2018;3(30):eaau6759. 30578351 10.1126/sciimmunol.aau6759 PMC6380365

[57] Schurr E , Gros P . A common genetic fingerprint in leprosy and Crohn's disease? N Engl J Med. 2009;361(27):2666‐2668. 20018963 10.1056/NEJMe0910690

[58] Fahy JV . Type 2 inflammation in asthma–present in most, absent in many. Nat Rev Immunol. 2015;15(1):57‐65. 25534623 10.1038/nri3786 PMC4390063

[59] Modlin RL . Th1‐Th2 paradigm: insights from leprosy. J Invest Dermatol. 1994;102(6):828‐832. 8006444 10.1111/1523-1747.ep12381958

[60] Kaplanski G . Interleukin‐18: biological properties and role in disease pathogenesis. Immunol Rev. 2018;281(1):138‐153. 29247988 10.1111/imr.12616 PMC7165732

[61] DePristo MA , Banks E , Poplin R , et al. A framework for variation discovery and genotyping using next‐generation DNA sequencing data. Nat Genet. 2011;43(5):491‐498. 21478889 10.1038/ng.806 PMC3083463

[62] Lee S , Wright FA , Zou F . Control of population stratification by correlation‐selected principal components. Biometrics. 2011;67(3):967‐974. 21133882 10.1111/j.1541-0420.2010.01520.x PMC3117098

[63] Liu JZ , Tozzi F , Waterworth DM , et al. Meta‐analysis and imputation refines the association of 15q25 with smoking quantity. Nat Genet. 2010;42(5):436‐440. 20418889 10.1038/ng.572 PMC3612983

[64] Consortium GT , Laboratory DA , et al, Coordinating Center ‐Analysis Working G . Genetic effects on gene expression across human tissues. Nature. 2017;550(7675):204‐213. 29022597 10.1038/nature24277 PMC5776756

[65] Giambartolomei C , Vukcevic D , Schadt EE , et al. Bayesian test for colocalisation between pairs of genetic association studies using summary statistics. PLoS Genet. 2014;10(5):e1004383. 24830394 10.1371/journal.pgen.1004383 PMC4022491

[66] Kircher M , Witten DM , Jain P , O'Roak BJ , Cooper GM , Shendure J . A general framework for estimating the relative pathogenicity of human genetic variants. Nat Genet. 2014;46(3):310‐315. 24487276 10.1038/ng.2892 PMC3992975

[67] Boix CA , James BT , Park YP , Meuleman W , Kellis M . Regulatory genomic circuitry of human disease loci by integrative epigenomics. Nature. 2021;590(7845):300‐307. 33536621 10.1038/s41586-020-03145-z PMC7875769

[68] Zeng W , Liu Q , Yin Q , Jiang R , Wong WH . HiChIPdb: a comprehensive database of HiChIP regulatory interactions. Nucleic Acids Res. 2022. 10.1093/nar/gkac859 PMC9825415 36215037

[69] Yang J , Lee SH , Goddard ME , Visscher PM . GCTA: a tool for genome‐wide complex trait analysis. Am J Hum Genet. 2011;88(1):76‐82. 21167468 10.1016/j.ajhg.2010.11.011 PMC3014363

