# Supplementary material for: Genome‐wide meta‐analysis and fine‐mapping prioritize potential causal variants and genes related to leprosy
Source: MedComm (2020). 2023 Nov 24;4(6):e415. doi: 10.1002/mco2.415 (PMC10674079; doi:10.1002/mco2.415)
Supplement: Supplementary file 1 — Supporting Information [file MCO2-4-e415-s001.docx]

**Supplementary Information**

**Genome-wide meta-analysis and fine-mapping prioritize potential causal variants and genes related to leprosy**

**Authors:** Zhenzhen Wang^1,2#^, Tingting Liu^2,#^, Wenchao Li^2^, Gongqi Yu^2^, Zihao Mi^2^, Chuan Wang^2^, Xiaojie Liao^2^, Pengcheng Huai^2^, Tongsheng Chu^2^, Dianchang Liu^2^, Lele Sun^2^, Xi’an Fu^2^, Yonghu Sun^2^, Honglei Wang^2^, Na Wang^2^, Jianjun Liu^3^, Hong Liu^2*^, Furen Zhang^2*^

**Affiliations:**

1. Department of Biostatistics, School of Public Health, Cheeloo College of Medicine, Shandong University, Jinan 250012, Shandong, China;
2. Shandong Hospital for Skin Diseases & Shandong Provincial Institute of Dermatology and Venereology, Shandong First Medical University & Shandong Academy of Medical Sciences, Jinan 250022, Shandong, China;
3. Genome Institute of Singapore, Singapore 138632, Singapore;

^#^ These authors contributed equally.

^*^ Corresponding author.

^*^ Correspondence to**:** Furen Zhang ([zhangfuren@hotmail.com](mailto:zhangfuren@hotmail.com)) or Hong Liu [(hongyue2519@hotmail.com)](mailto:(hongyue2519@hotmail.com))

Telephone number: 86-0531-87298801.

Fax number: 86-0531-87984734.

Addresses: Shandong Hospital for Skin Diseases & Shandong Provincial Institute of Dermatology and Venereology, Shandong First Medical University & Shandong Academy of Medical Sciences.

27397 Jingshi Lu, Jinan 250022, Shandong Province, P.R. China


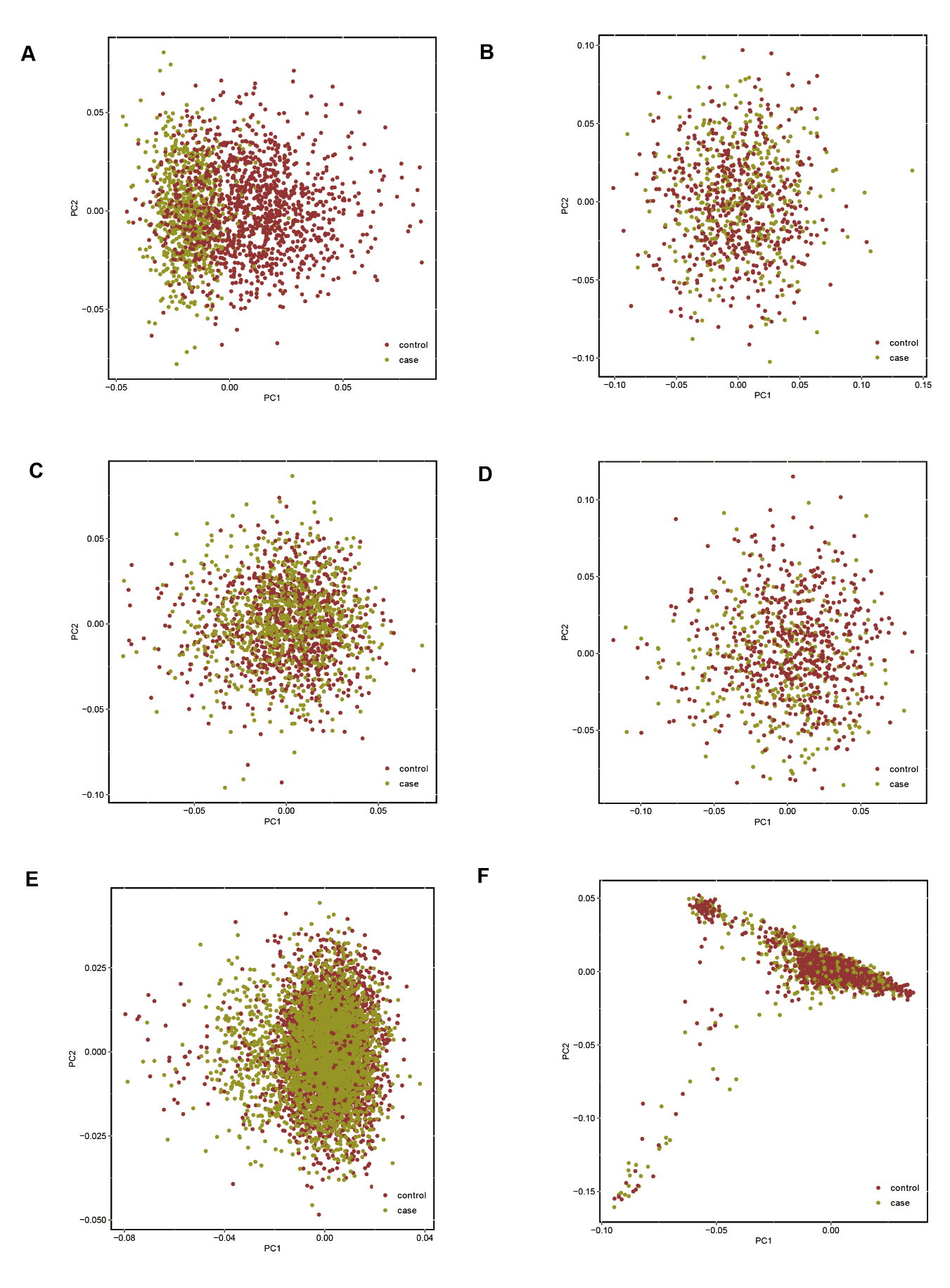


Figure S1. Principal components analysis (PCA) of GWAS1-6.

Colors represent study populations. **A)** The top two principal Components between 706 cases and 1,223 controls of GWAS1;**B)** The top two principal Components between 374 cases and 510 controls of GWAS2; **C)** The top two principal Components between 864 cases and 853 controls of GWAS3; **D)**The top two principal Components between 333 cases and 573 controls of GWAS4; **E)** The top two principal Components between 2,991 cases and 3,110 controls of GWAS5; **F)**The top two principal Components between 2,016 cases and 1,469 controls of GWAS6.


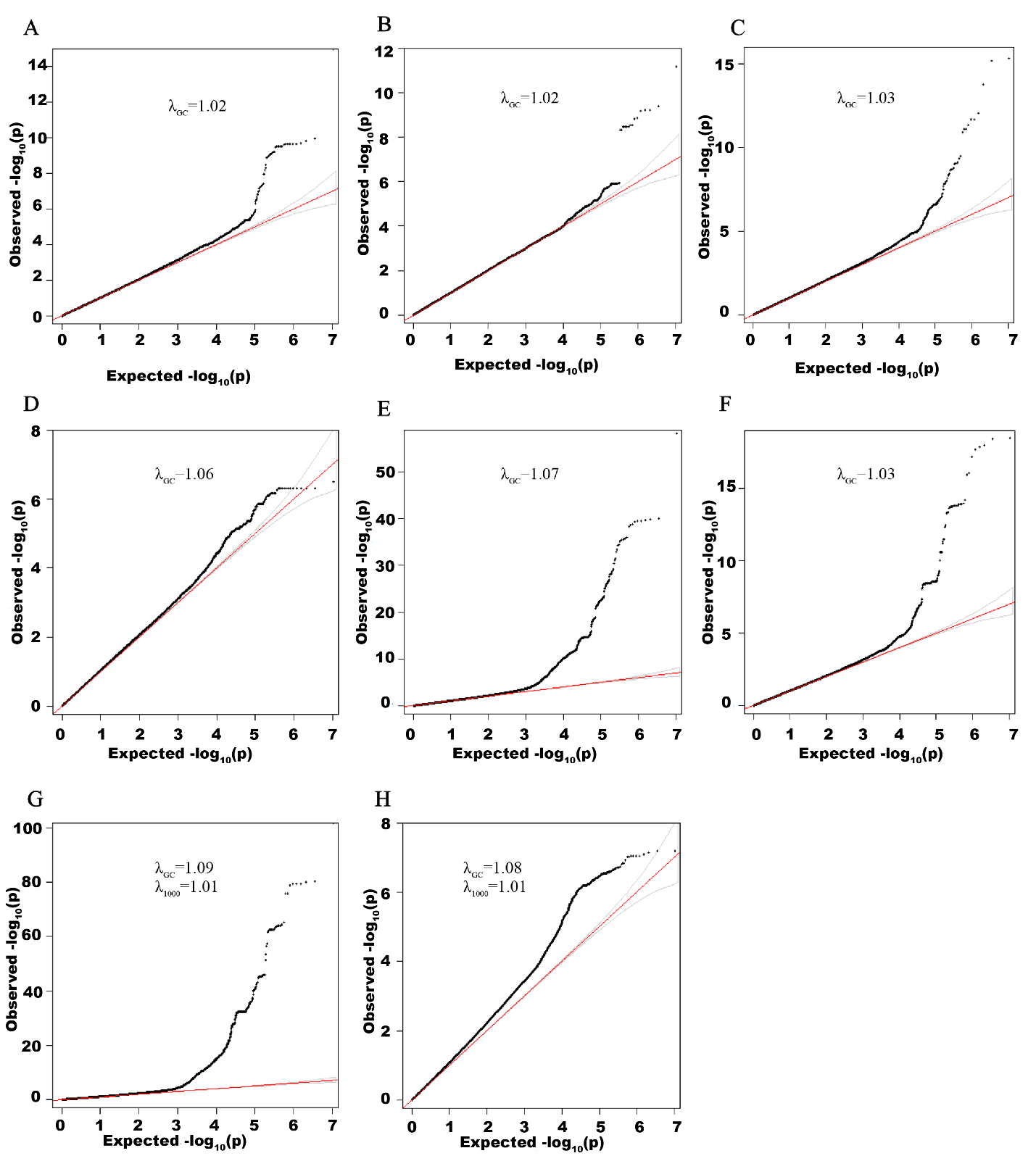


Figure S2. Quantile-quantile plot of the associations.

**A-F)** QQ plot of GWAS1 to GWAS6; **G)** Before removal of SNPs located within known leprosy susceptibility loci; **H)** After removal of SNPs located within known leprosy susceptibility loci.


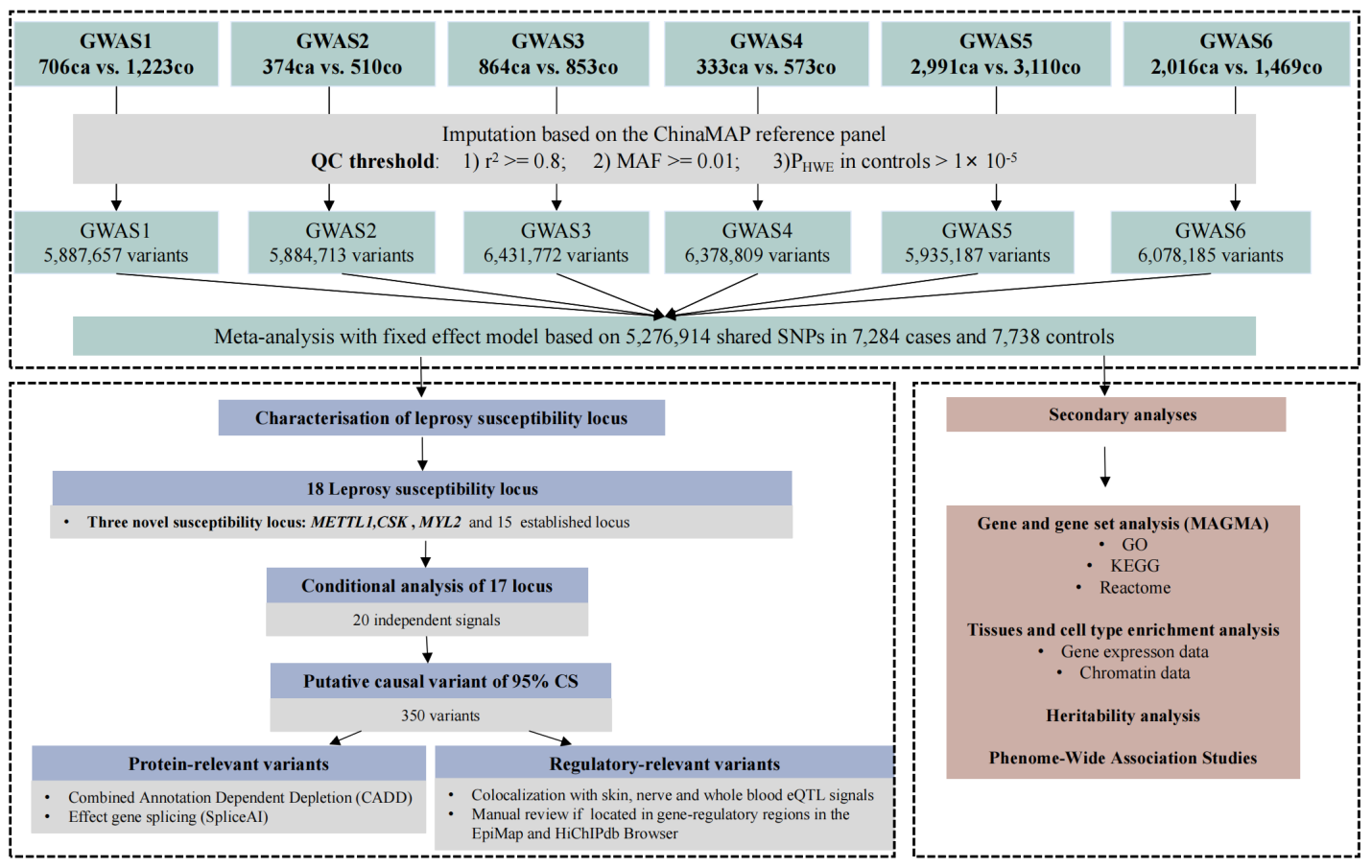


Figure S3. Summary of the Study design and analysis flowchart.

The flowchart shows the number of samples and variants included at each GWAS dataset after quality control (QC) (green boxs), the fine-mapping strategy to characterize leprosy susceptibility locus (blue boxs) and other analytical strategies employed (brown boxs).

GWAS, genome-wide association study;ca, cases; co, controls; r^2^, square correlation; MAF, minor allele frequency; P_HWE,_ P value of Hardy-Weinberg equilibrium; CS, credible set; GO, Gene Ontology; KEGG, Kyoto Encyclopedia of Genes and Genomes.


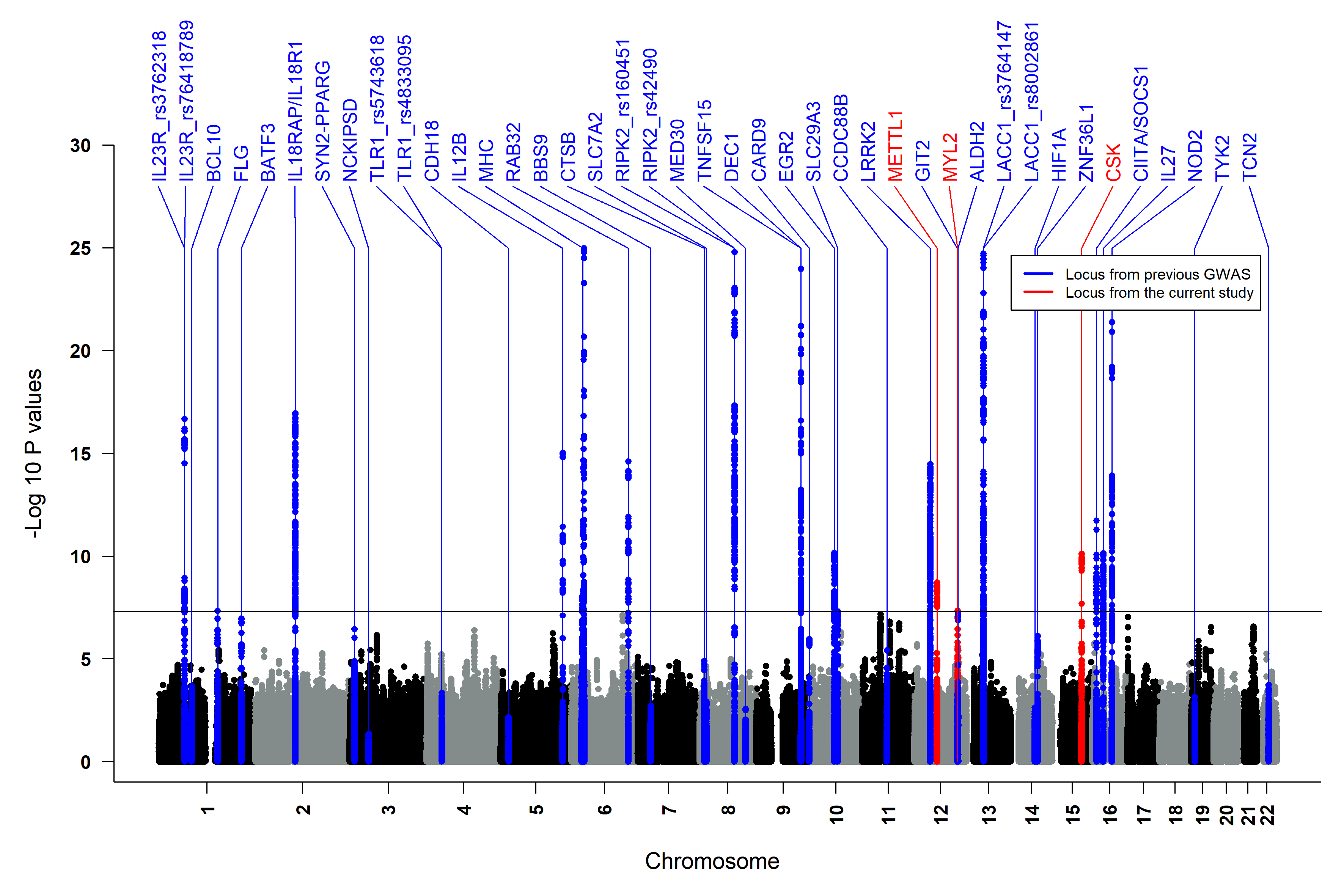


Figure S4. Chromosomal plot of the Genome-wide association analysis.

Previously published locus with genome-wide significance were highlighted in blue. Novel locus (P < 5 × 10^−8^) were highlighted in red.

A) *EGR2*:


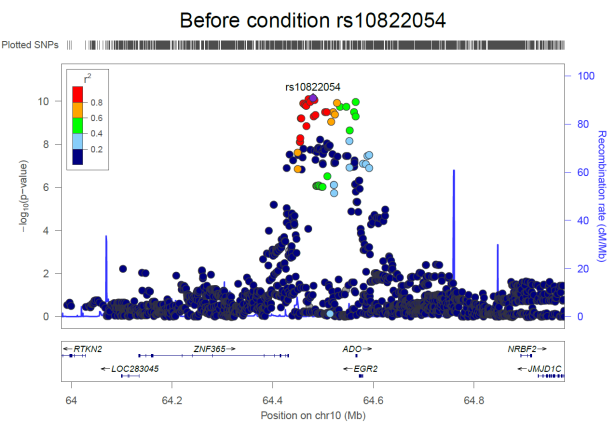

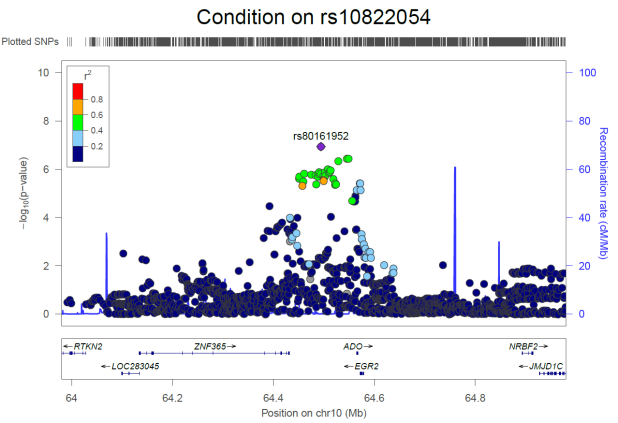

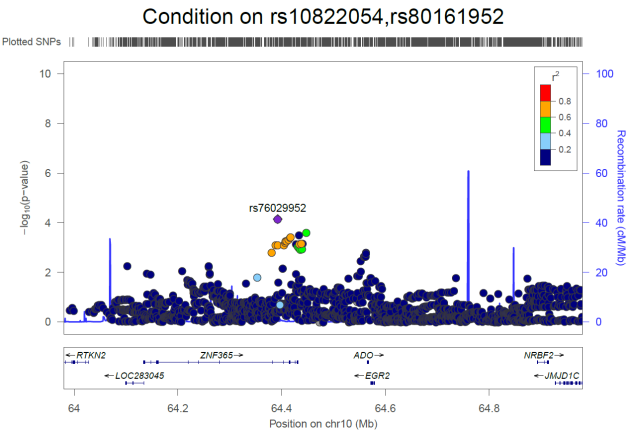


B) *TNFSF15:*


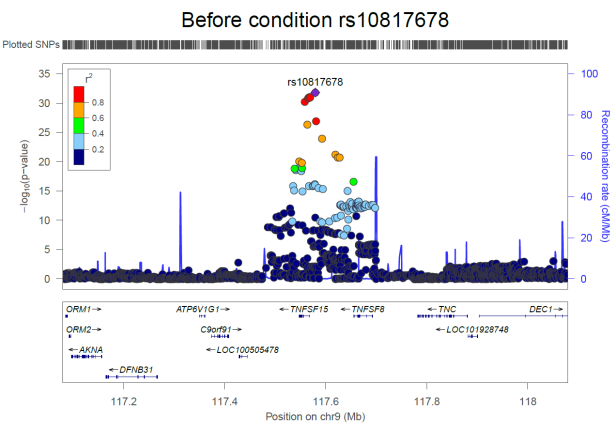

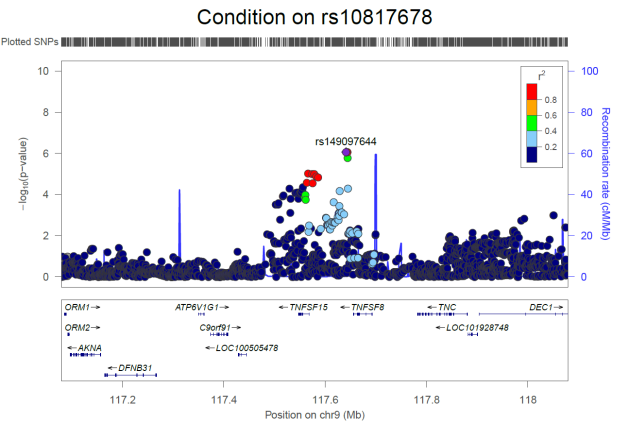

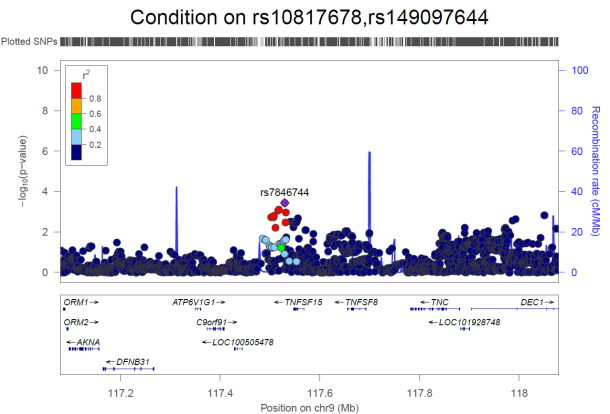


C) *LACC1*:


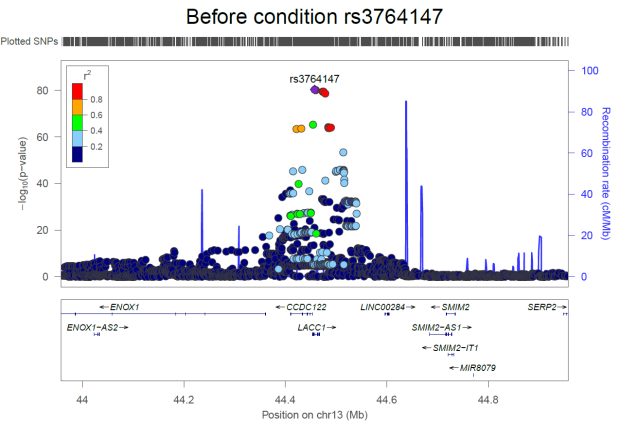

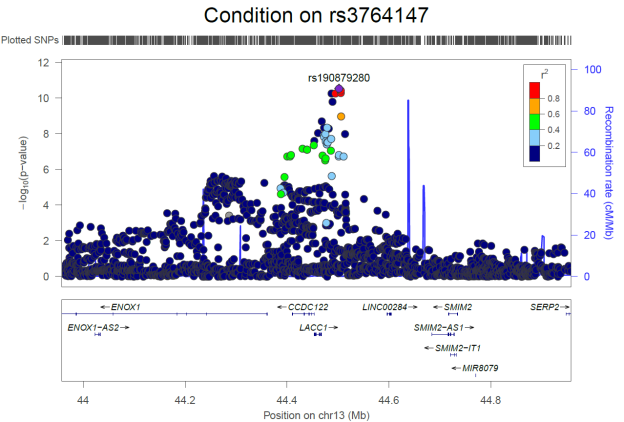


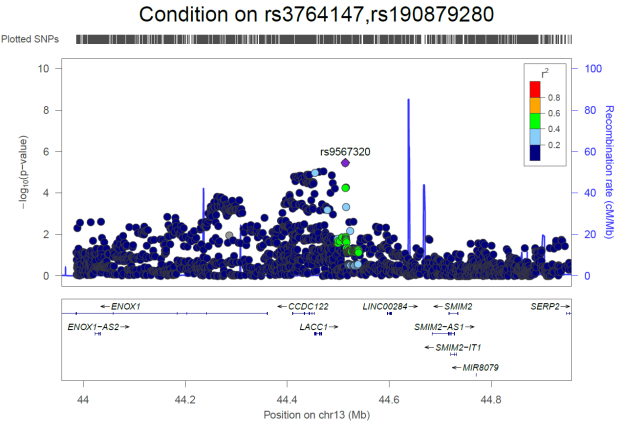


D) *IL23R*:


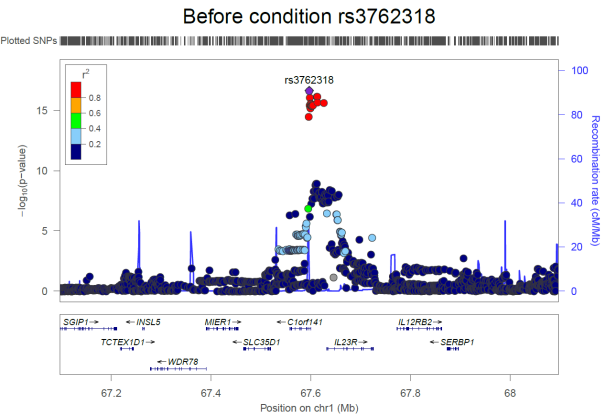

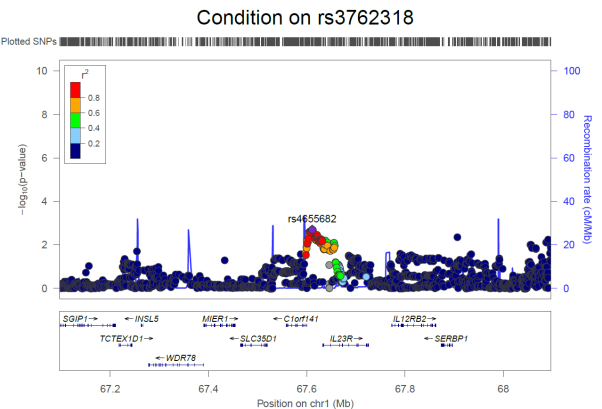


E) *FLG*:


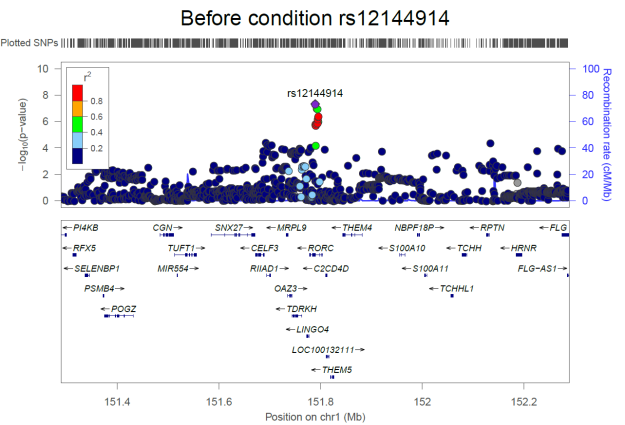

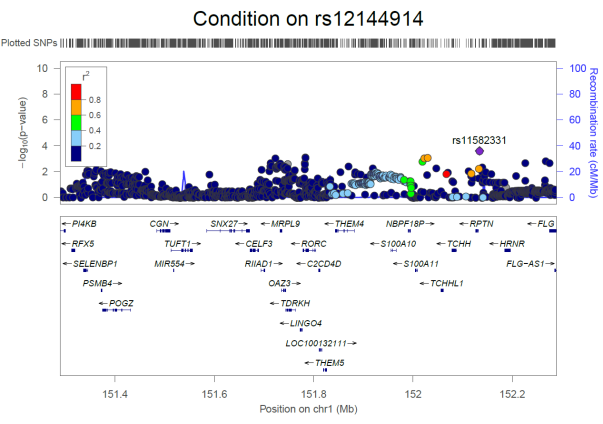


F) *IL18RAP*:


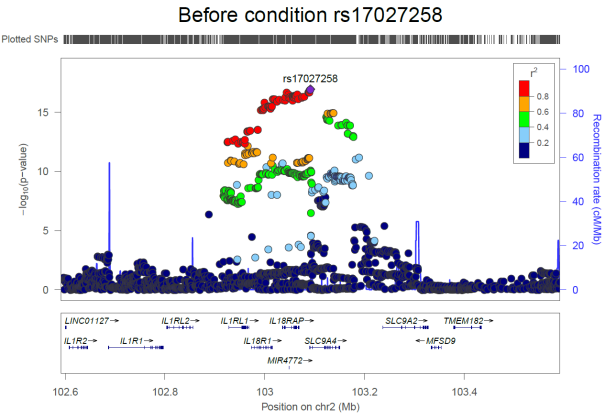

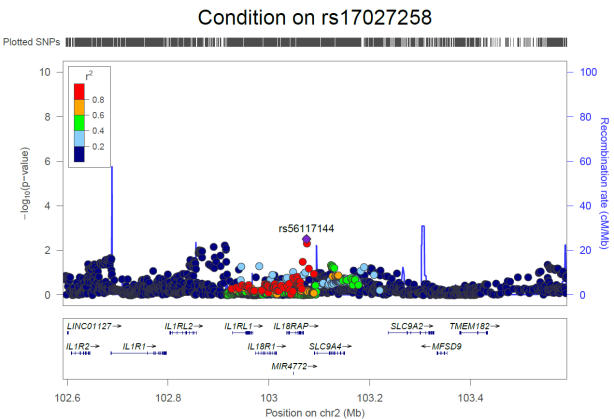


G) *IL12B*:


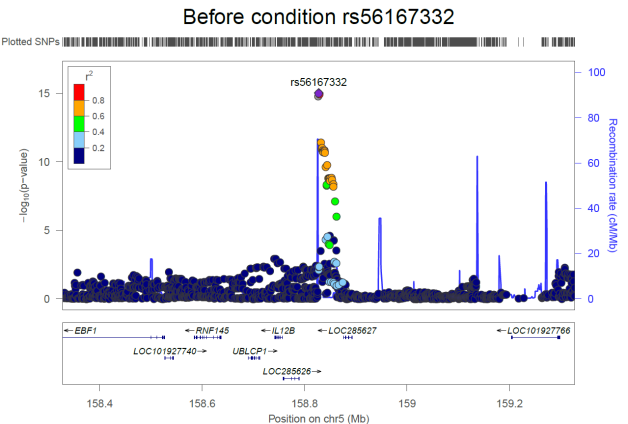

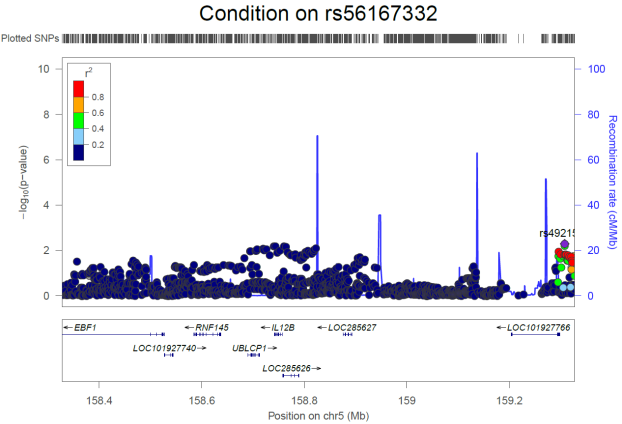


H) *RAB32*:


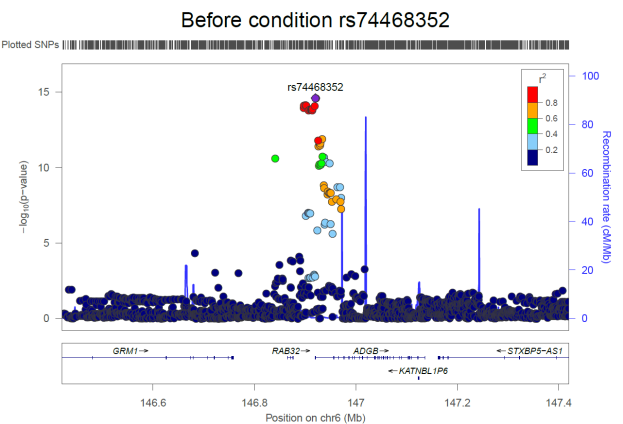

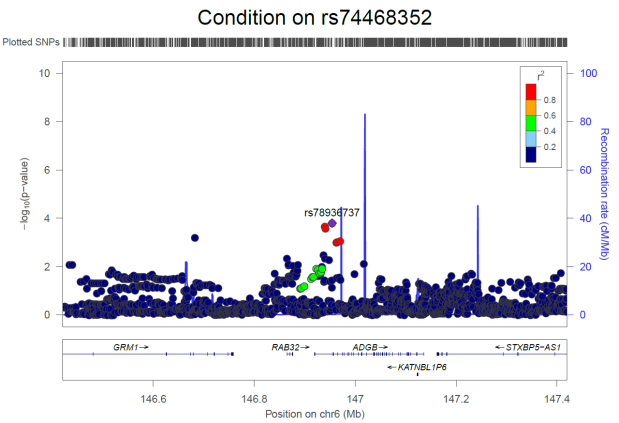


I) *RIPK2*:


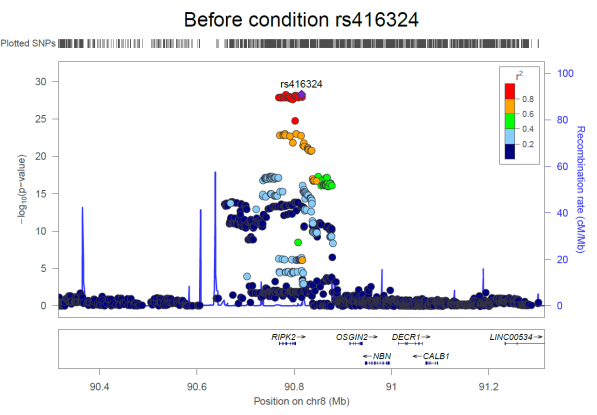

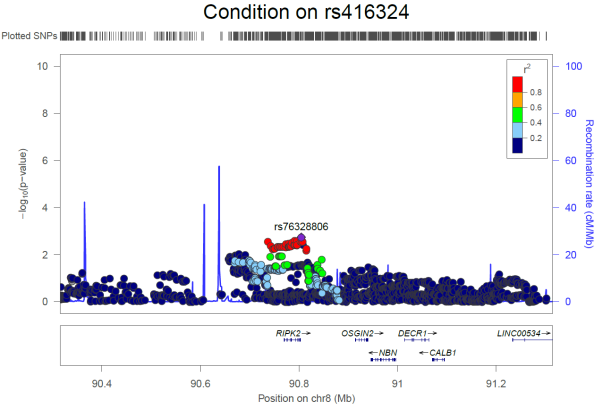


J) *SLC29A3*:


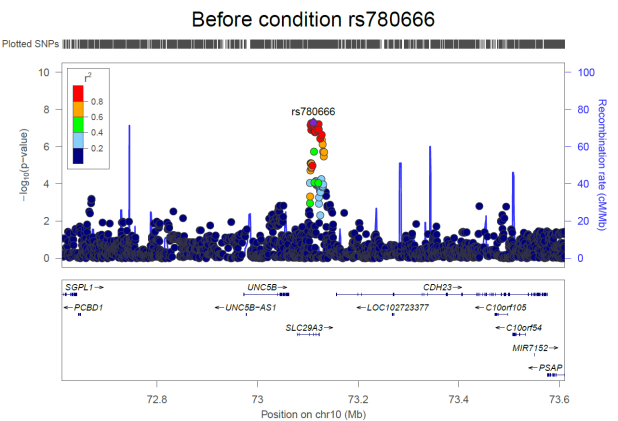

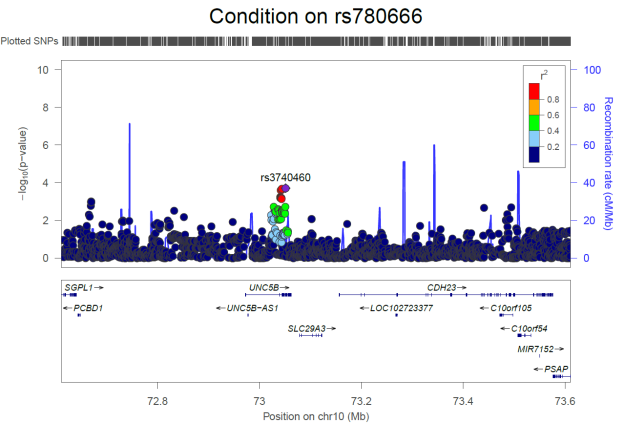


K) *LRRK2*:


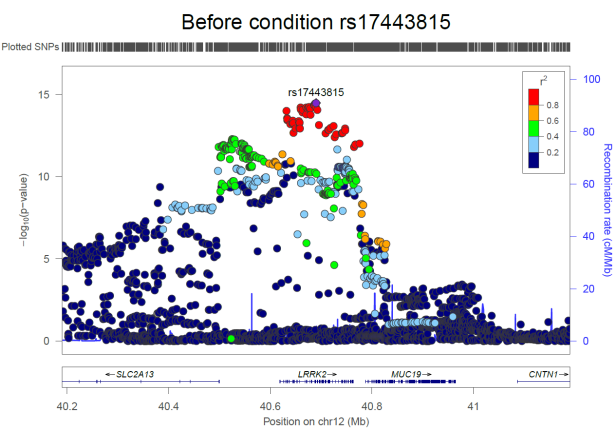

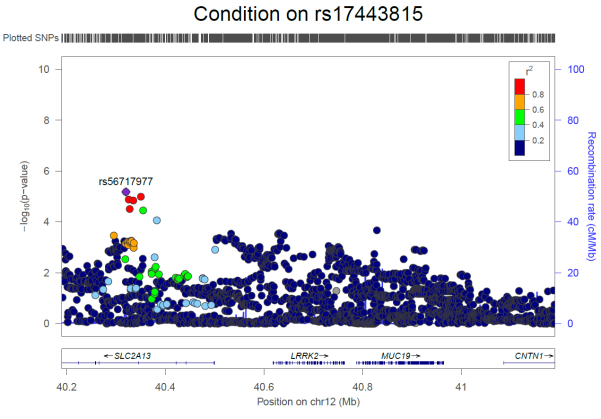


L) *CIITA*-*SOCS1*:


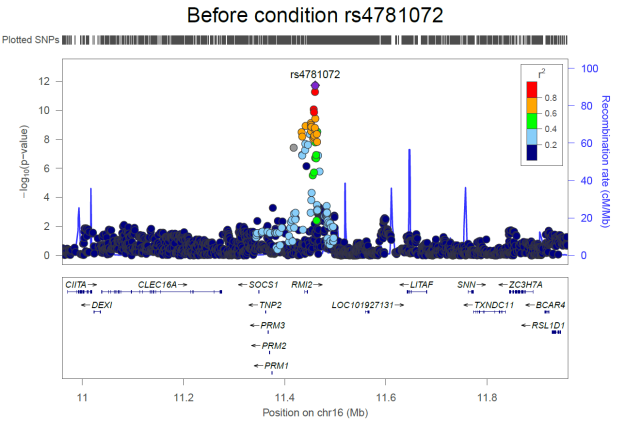

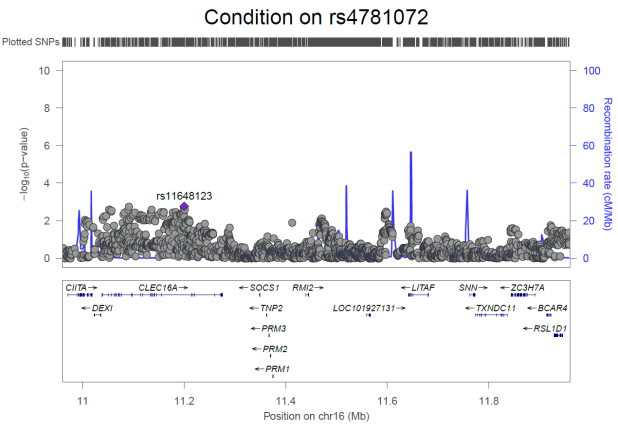


M) *IL27*:


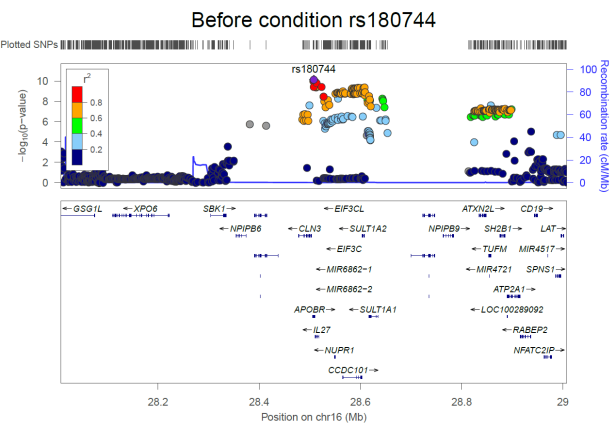

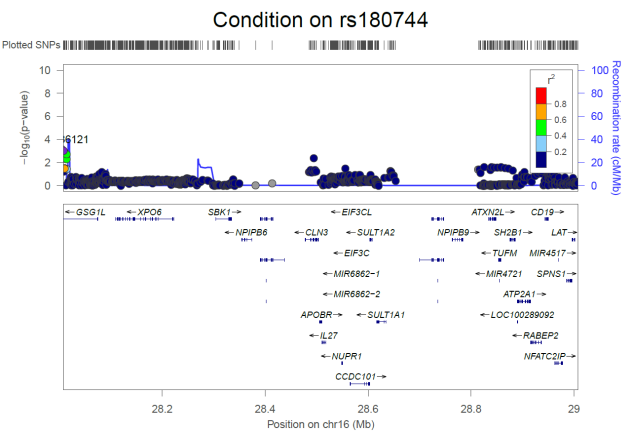


N) *NOD2*:


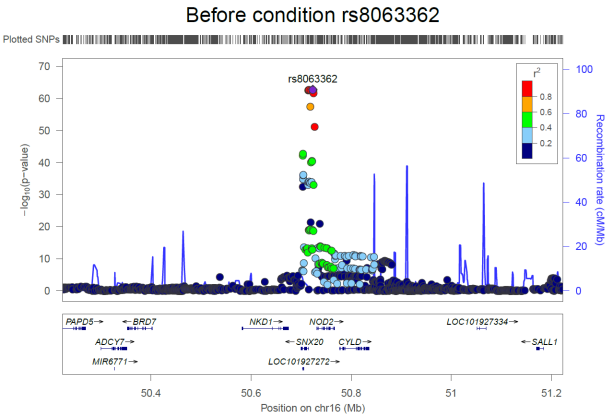

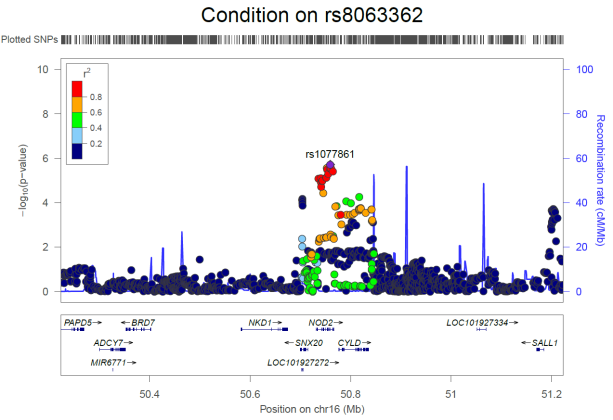


O) chr12:57,665,085 - 58,665,085


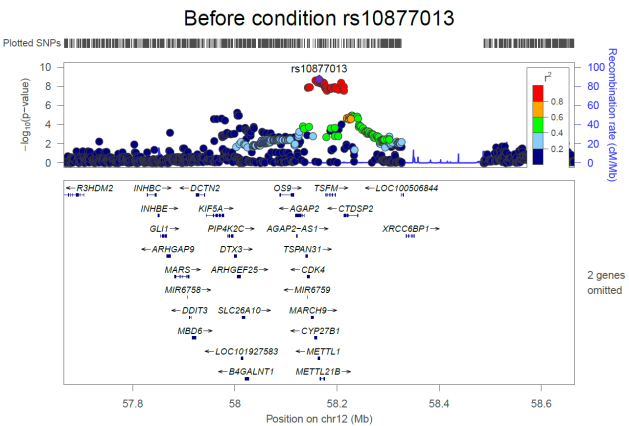

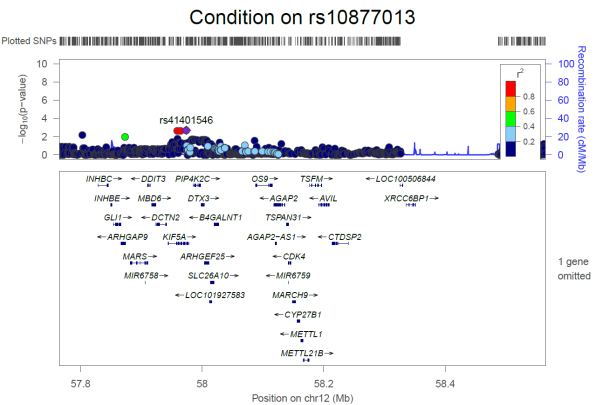


P) chr12:110,914,461 - 111,914,461


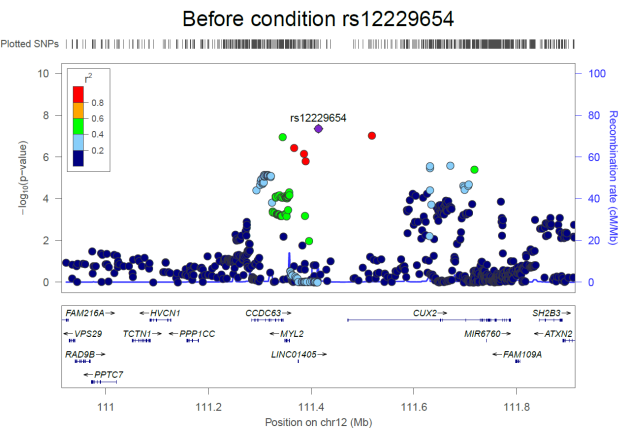

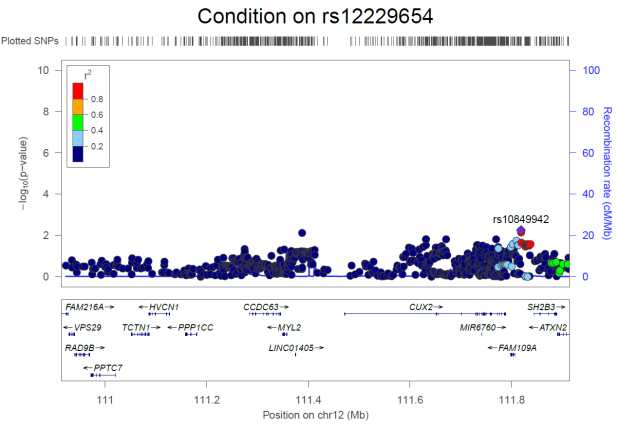


Q) chr15:74,587,571 - 75,587,571


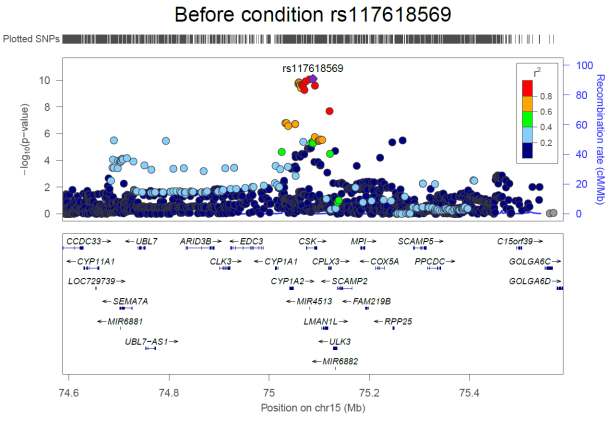

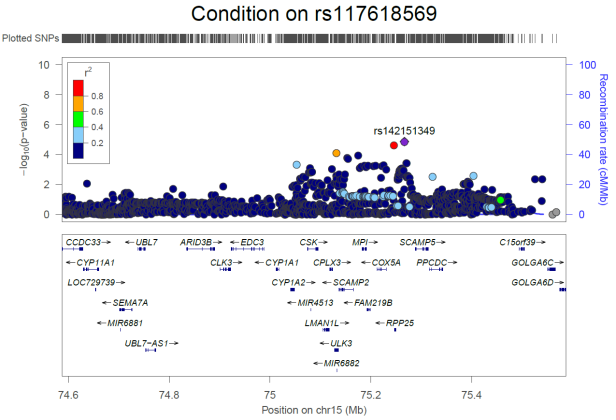


Figure S5. Regional plots of leprosy susceptibility locus.

**A-N)** Regional plots of 14 known leprosy susceptibility locus using the online LocusZoom tool. Top left: The meta-analysis results before condition top SNP. Top right: The meta-analysis results after condition top SNP. Bottom left: The meta-analysis results after condition top two independent SNPs.

**O-Q)** Regional plots of three novel leprosy susceptibility locus. Left: The meta-analysis results before condition top SNP. Right: The meta-analysis results after condition top SNP.


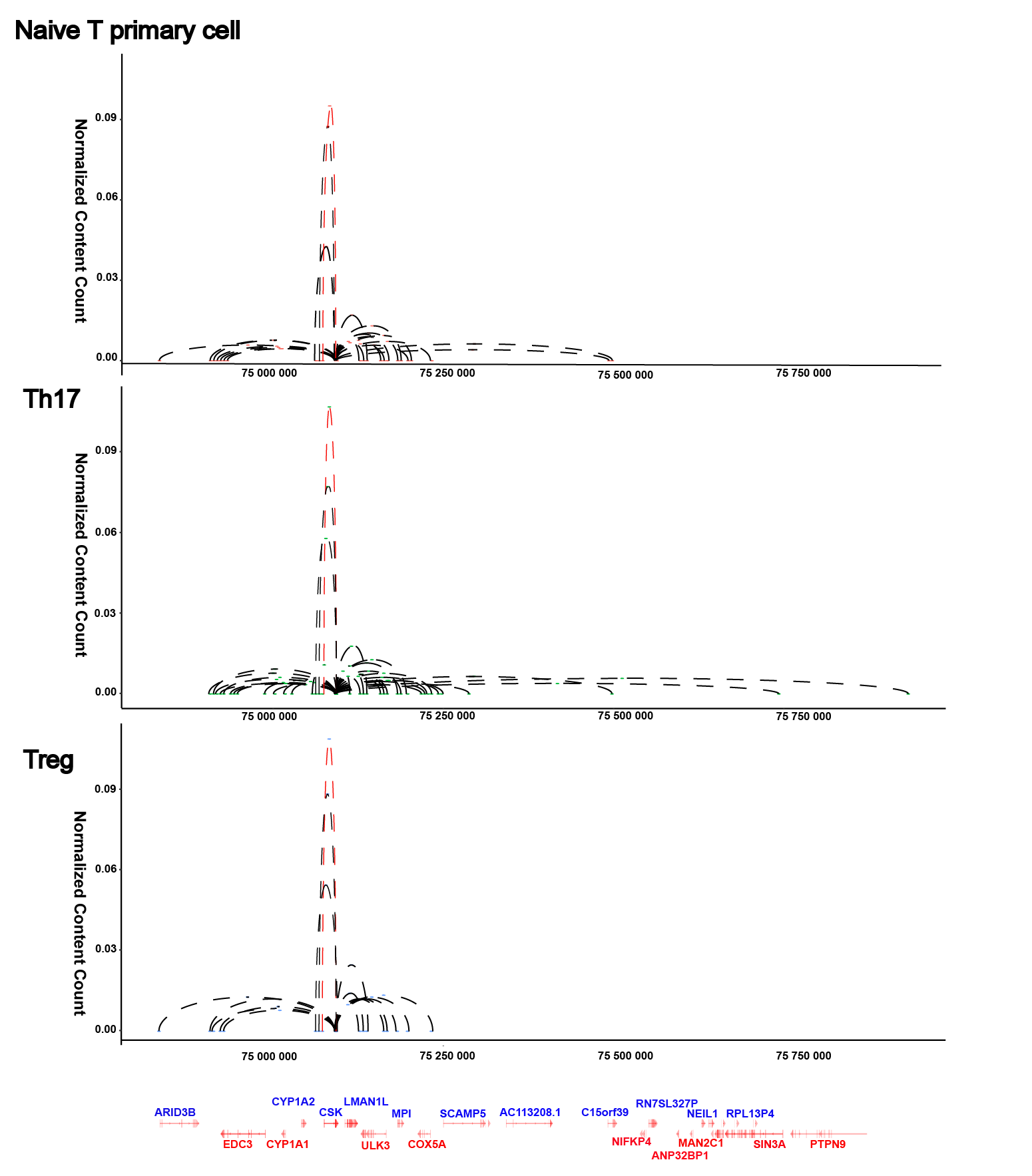


Figure S6. The interactions between enhancer region chr15:75090000-75095000 and potential target genes.

HiChIP 3D signal enrichment at the enhancer region chr15:75090000-75095000 and all promoter region in Treg, TH17 and Naive T primary cells from HiChIPdb.

A) 22 SNPs in *EGR2* loci.


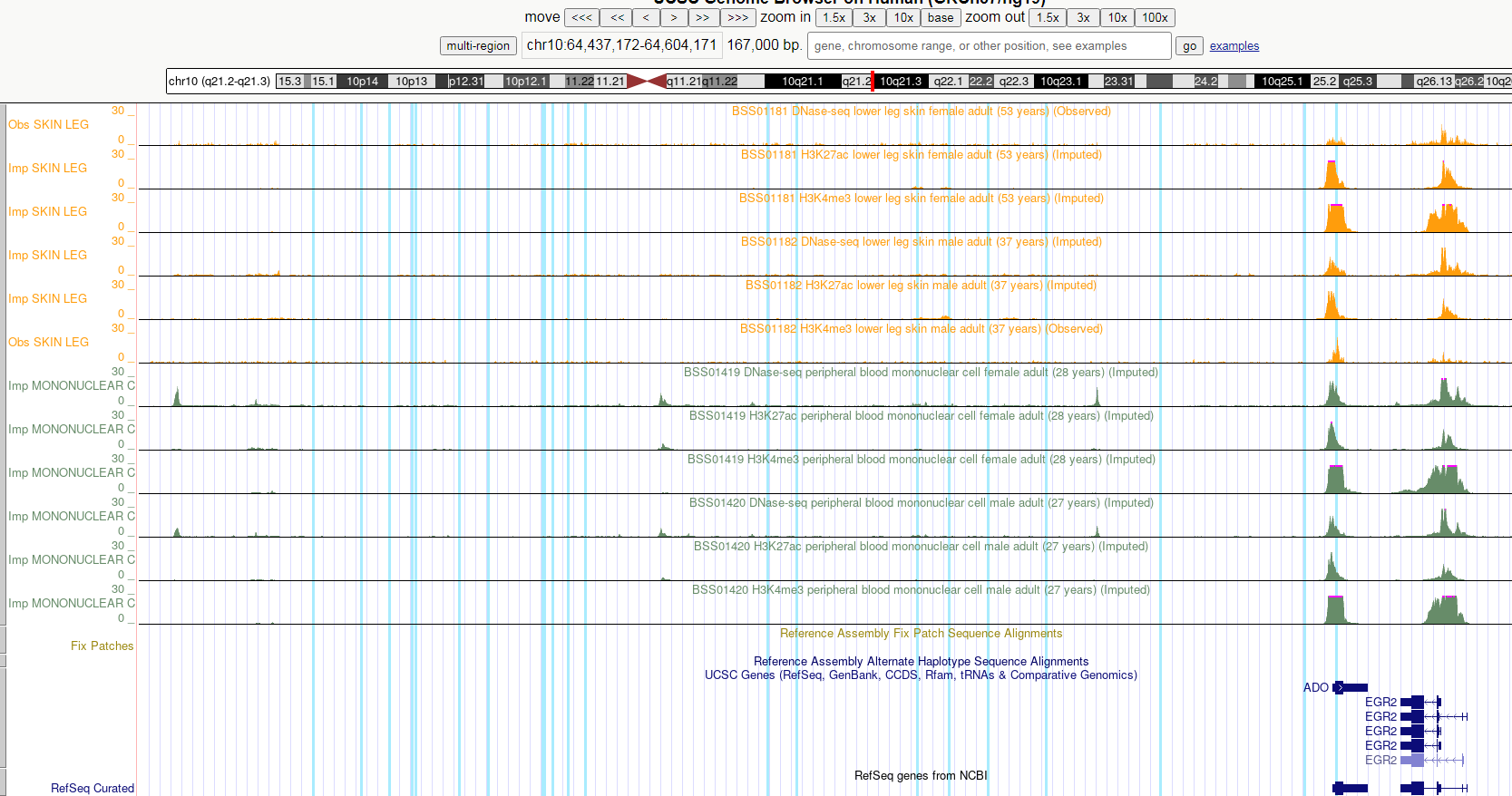


rs2236295

rs10995311


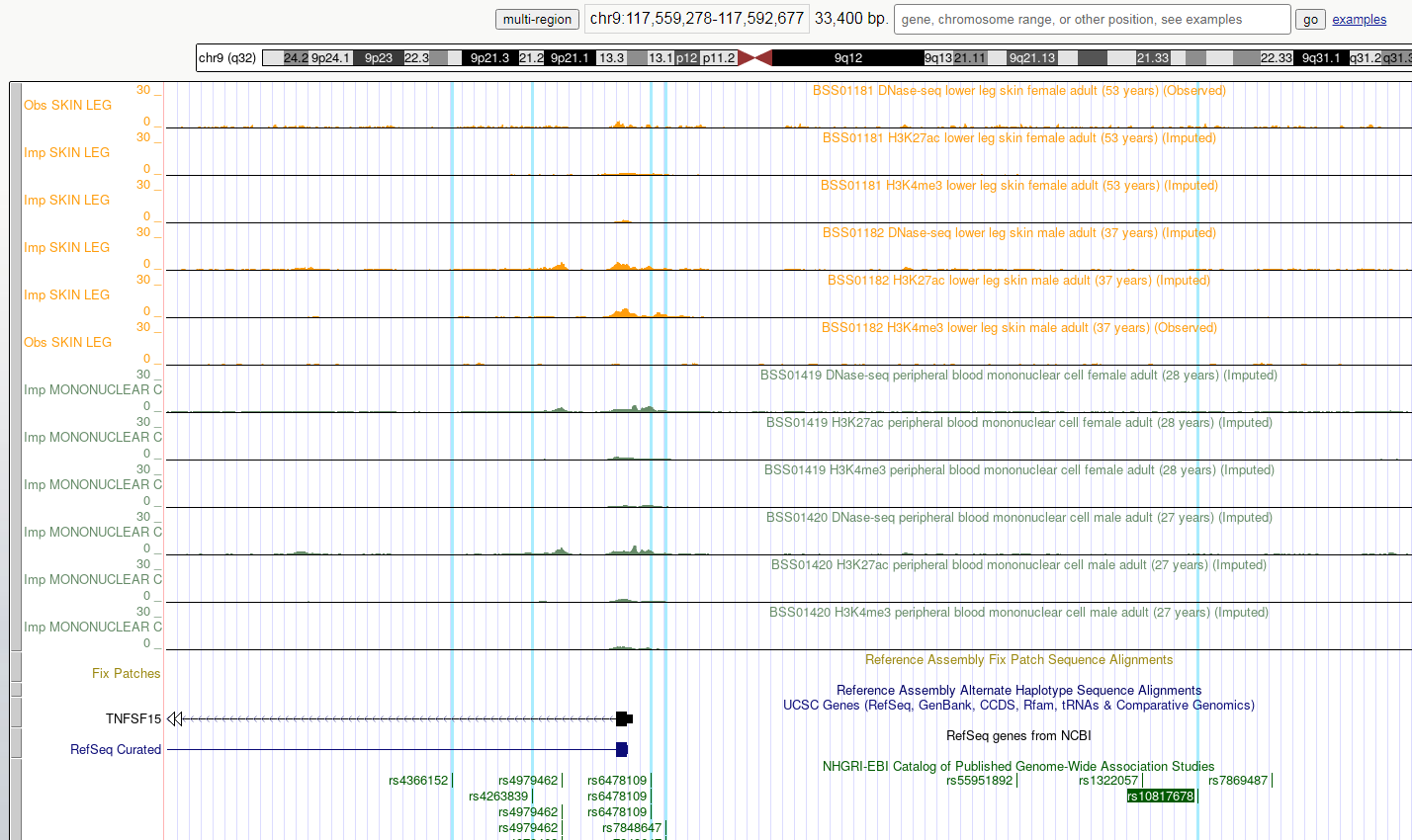


rs6478109

B) 5 SNPs in *TNFSF15* loci

C) 14 SNPs in *RAB32* loci


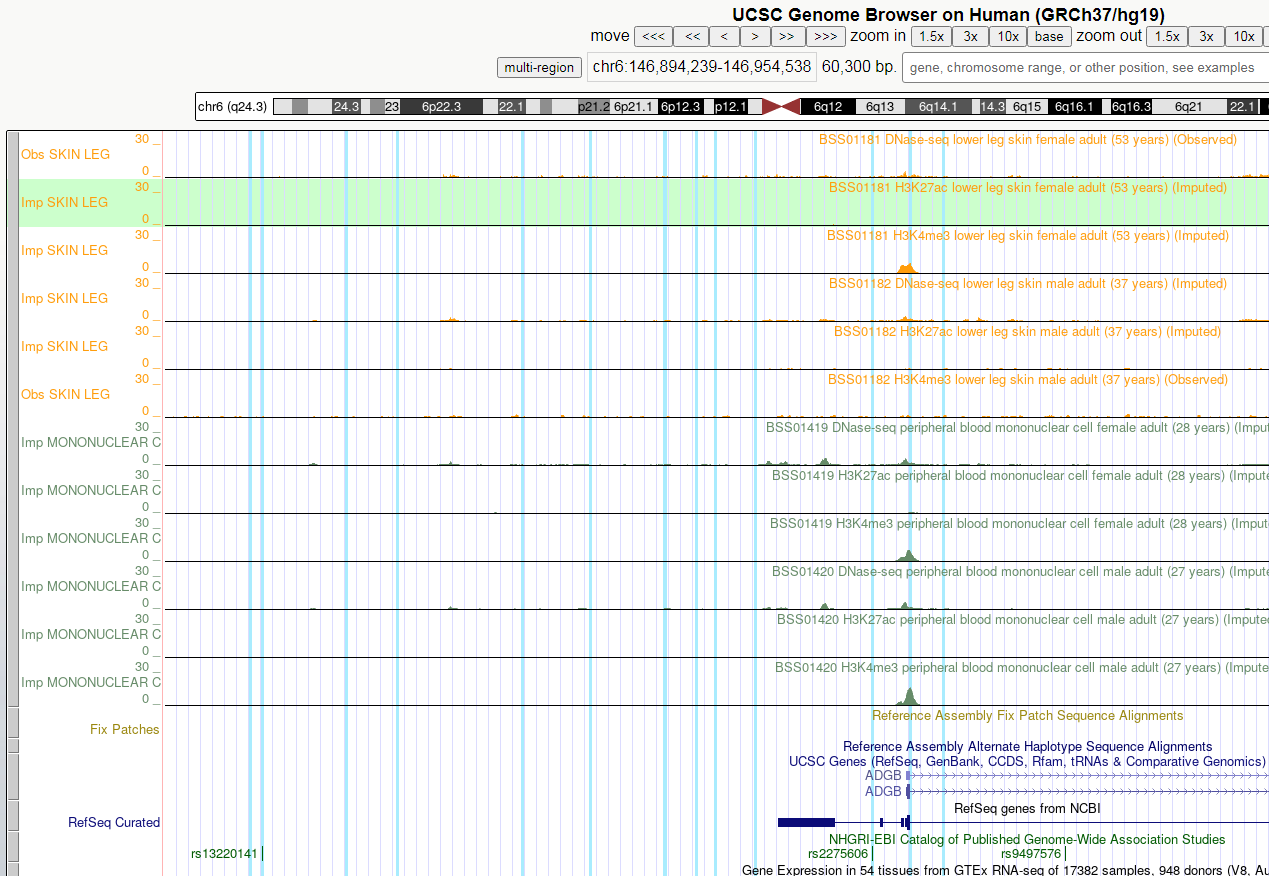


rs74468352

D) 8 SNPs in*NOD2* loci

rs2270368

rs2287195

rs9302752


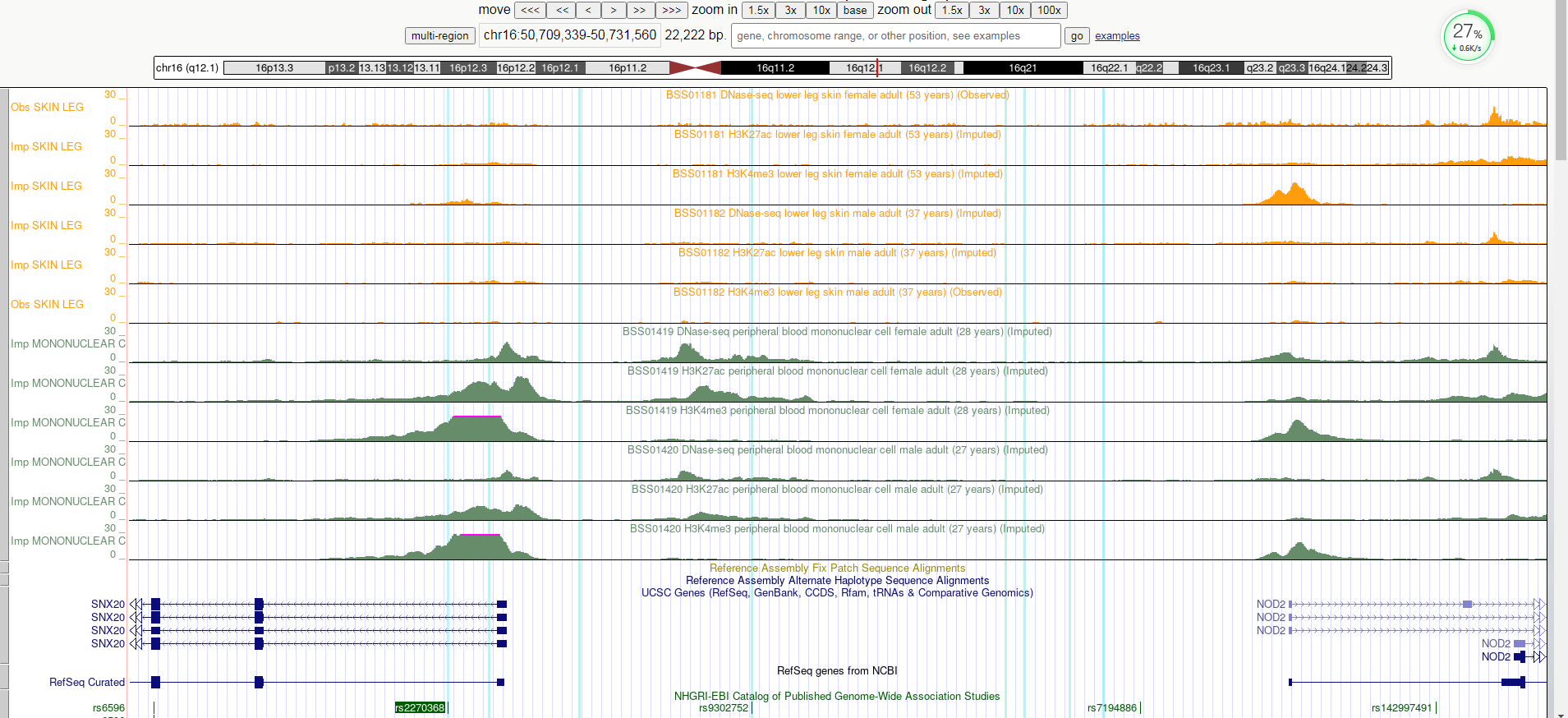


Figure S7. Epigenetic modification of the SNPs in known region.

Histone mark signals of H3K4me3 ChIP-seq, H3K27ac ChIP-seq and DNase-seq from PBMC (green) and skin (orange) from EpiMap and visualized with the UCSC genome browser.

Table S1.Baseline characteristics of cases and controls

|  | CASES | | |  |  | CONTROLS | | | Genotyping chip |
| --- | --- | --- | --- | --- | --- | --- | --- | --- | --- |
| Study | N | Male/ Female | Mean age | Mean age at onset |  | N | Male/ Female | Mean age |  |
| GWAS 1 | 706 | 555/151 | 65.5 | 21.8 |  | 1223 | 669/554 | 34.9 | Illumina Human 610K-Quad BeadChips |
| GWAS 2 | 374 | 297/77 | 65.1 | 25.6 |  | 510 | 381/129 | 43.7 | Illumina Human 660K-Quad BeadChips |
| GWAS 3 | 864 | 700/164 | 67.9 | 22.7 |  | 853 | 623/230 | 48.5 | Illumina Omni Zhonghua Array |
| GWAS 4 | 333 | 221/112 | 59.0 | 20.4 |  | 573 | 352/221 | 46.3 | Illumina Omni Zhonghua Array |
| GWAS 5 | 2991 | 2406/585 | 70.1 | 23.8 |  | 3110 | 2423/687 | 66.5 | Illumina Asian Screening Array (ASA) BeadChip |
| GWAS 6 | 2016 | 1466/550 | 56.4 | 27.9 |  | 1469 | 737/732 | 41.2 | Illumina Asian Screening Array (ASA) BeadChip |
| Total/Mean | 7284 | 5645/1639 | 64.8 | 24.6 |  | 7738 | 5185/2553 | 51.7 |  |

Table S2. Associations of known variants in known loci.

| **SNP information** | | | | | |  | **Meta-analysis results** | | | **ref** |
| --- | --- | --- | --- | --- | --- | --- | --- | --- | --- | --- |
| **Reported Gene** | **CHR** | **BP** | **SNP** | **A1** | **A2** |  | **OR(f)** | **P(f)** | **Phet** |  |
| ***IL23R*** | **1** | **67597119** | **rs3762318** | **G** | **A** |  | **0.69** | **2.15E-17** | **0.44** | **^1^** |
|  |  | **67648596** | **rs76418789** | **.** | **.** |  | **.** | **.** | **.** | **^2^** |
| *BCL10* | 1 | 85744472 | rs2735591 | A | G |  | 1.10 | 4.09E-04 | 0.07 | ^3^ |
| *BATF3* | 1 | 212873431 | rs2221593 | T | C |  | 1.17 | 1.18E-07 | 0.13 | ^4^ |
| ***FLG*** | **1** | **152275298** | **rs146466242** | **A** | **T** |  | **1.27** | **1.63E-04** | **0.21** | **^2^** |
| ***IL18RAP/IL18R1*** | **2** | **103054449** | **rs2058660** | **G** | **A** |  | **0.85** | **1.28E-10** | **0.78** | **^5^** |
| *SYN2/PPARG* | 3 | 12313846 | rs6807915 | C | T |  | 0.91 | 1.05E-04 | 0.27 | ^6^ |
| *NCKIPSD* | 3 | 48719549 | rs145562243 | . | . |  | . | . | . | ^2^ |
| *TLR1* | 4 | 38799710 | rs4833095 | T | C |  | 1.01 | 7.42E-01 | 0.69 | ^7^ |
|  |  | 38798648 | rs5743618 | . | . |  | . | . | . | ^8^ |
| *CDH18* | 5 | 20374213 | rs73058713 | A | C |  | 1.09 | 9.45E-03 | 0.67 | ^4^ |
| ***IL12B*** | **5** | **158826792** | **rs6871626** | **A** | **C** |  | **0.81** | **1.58E-15** | **0.62** | **^5^** |
| *CDSN/PSORS1C1/PSORS1C2* | 6 | 31081409 | rs1265048 | C | T |  | 0.92 | 4.95E-03 | 0.12 | ^9^ |
| ***RAB32*** | **6** | **146918950** | **rs2275606** | **A** | **G** |  | **1.25** | **8.33E-15** | **0.03** | **^1^** |
| ***HLA-DRB1*** | **6** | **32552064** | ***HLA-DRB1*15*** | **P** | **A** |  | **1.96** | **1.24E-103** | **0.03** | **^6^** |
|  | **6** | **32574060** | **rs9270986** | **A** | **C** |  | **1.91** | **4.57E-105** | **0.04** | **^4^** |
| *BBS9* | 7 | 33469241 | rs4720118 | **T** | **C** |  | **1.07** | **1.52E-02** | **0.08** | ^6^ |
| *CTSB* | 8 | 11749242 | rs55894533 | C | A |  | 1.12 | 1.24E-05 | 0.94 | ^6^ |
| *MED30* | 8 | 118626279 | rs10100465 | A | G |  | 0.93 | 1.54E-02 | 0.04 | ^6^ |
| ***RIPK2*** | **8** | **90778513** | **rs42490** | **A** | **G** |  | **0.78** | **1.51E-23** | **0.19** | **^10^** |
|  |  | **90662941** | **rs160451** | **T** | **C** |  | **0.80** | **3.51E-14** | **0.52** | **^4^** |
| *SLC7A2* | 8 | 17396415 | rs13259978 | . | . |  | . | . | . | ^11^ |
| ***TNFSF15*** | **9** | **117568766** | **rs6478109** | **G** | **A** |  | **0.75** | **8.31E-32** | **0.03** | **^10^** |
| *DEC1* | 9 | 118143933 | rs10817758 | T | C |  | 1.09 | 7.32E-04 | 0.06 | ^4^ |
| *CARD9* | 9 | 139258965 | rs149308743 | . | . |  | . | . | . | ^2^ |
| ***EGR2*** | **10** | **64507904** | **rs58600253** | **T** | **C** |  | **1.23** | **9.06E-09** | **0.95** | **^4^** |
| ***SLC29A3*** | **10** | **73111408** | **rs780668** | **T** | **C** |  | **1.14** | **7.57E-08** | **0.77** | **^2^** |
| *CCDC88B* | 11 | 64107735 | rs663743 | A | G |  | 1.13 | 2.05E-04 | 0.02 | ^4^ |
| ***LRRK2*** | **12** | **40552417** | **rs1873613** | **G** | **A** |  | **0.82** | **8.04E-12** | **0.03** | **^10^** |
| *GIT2* | 12 | 110390979 | rs925368 | . | . |  | . | . | . | ^11^ |
| *ALDH2* | 12 | 112241766 | rs671 | . | . |  | . | . | . | ^11^ |
| ***LACC1*** | **13** | **44457925** | **rs3764147** | **G** | **A** |  | **1.63** | **4.12E-81** | **0.79** | **^10^** |
|  |  | **44474517** | **rs8002861** | **G** | **A** |  | **0.88** | **3.82E-06** | **0.48** | **^4^** |
| *HIF1A* | 14 | 62203623 | rs142179458 | . | . |  | . | . | . | ^12^ |
| *ZNF36L1* | 14 | 69263599 | rs1465788 | C | T |  | 0.87 | 1.48E-06 | 0.54 | ^11^ |
| ***NOD2*** | **16** | **50719103** | **rs9302752** | **C** | **T** |  | **1.55** | **2.52E-63** | **0.03** | **^10^** |
| ***CIITA/SOCS1*** | **16** | **11452542** | **rs77061563** | **T** | **C** |  | **0.86** | **2.76E-09** | **0.84** | **^4^** |
| ***IL27*** | **16** | **28513403** | **rs181206** | **G** | **A** |  | **0.78** | **3.57E-10** | **0.59** | ^2^ |
| *TYK2* | 19 | 10469919 | rs55882956 | . | . |  | . | . | . | ^2^ |
| *TCN2* | 22 | 31007023 | rs75680863 | T | A |  | 0.88 | 3.11E-04 | 0.02 | ^11^ |

CHR, Chromosome; BP, position based on hg19 coordinates;

A1, minor allele; A2, major allele;

., not applicable;

OR, odds ratio based on minor allele; P, p-value of association based on log-additive model;(f) indicates results from fixed-effects meta analysis;

Phet, Pvalue of heterogeneity;

Bold indicates that this SNP or other SNPs within the known loci that have reached genome-wide significance level (P < 5×10^-8^);

ref, reference.

Table S3. Independent associations in Significant locus.

A) chr9:117,079,504 - 118,140,404

| SNP (effect allele) | | **rs10817678 (A)** | **rs149097644 (A)** |
| --- | --- | --- | --- |
| Unconditional | OR | 0.75 | 1.41 |
|  | P | 1.55E-32 | 1.02E-03 |
| Condition on rs10817678 | OR | **NA** | 1.69 |
|  | P | **NA** | 8.64E-07 |
| Condition on rs149097644 | OR | 0.73 | **NA** |
|  | P | 2.45E-35 | **NA** |
| r^2^ | **rs10817678** | 1 | 0.015192042 |
|  | **rs149097644** | 0.015192042 | 1 |

B) chr10:63,991,638 - 64,994,157

| SNP (effect allele) | | **rs10822054 (T)** | **rs80161952 (T)** |
| --- | --- | --- | --- |
| Unconditional | OR | 0.77 | 1.34 |
|  | P | 6.98E-11 | 5.99E-09 |
| Condition on rs10822054 | OR | **NA** | 1.31 |
|  | P | **NA** | 1.15E-07 |
| Condition on rs80161952 | OR | 0.78 | **NA** |
|  | P | 1.64E-09 | **NA** |
| r^2^ | **rs10822054** | 1 | 0.006123016 |
|  | **rs80161952** | 0.006123016 | 1 |

C) chr13:43,959,499 - 45,012,908

| SNP (effect allele) | | **rs3764147 (G)** | **rs190879280 (G)** |
| --- | --- | --- | --- |
| Unconditional | OR | 1.63 | 0.61 |
|  | P | 4.12E-81 | 1.90E-25 |
| Condition on rs3764147 | OR | **NA** | 0.72 |
|  | P | **NA** | 2.90E-11 |
| Condition on rs190879280 | OR | 1.58 | **NA** |
|  | P | 3.74E-67 | **NA** |
| r^2^ | **rs3764147** | 1 | 0.037874609 |
|  | **rs190879280** | 0.037874609 | 1 |

Table S4. The fine-mapping summary results of all putative causal variant in 20 independent loci.

| **GWAS_lead** | **Locus** | **SNP** | **CHR:BP_A1_A2** | **P_value** | **OR** | **Phet** | **r^2^** | **Gene** | **Function** | **PIP** | **CADD** |
| --- | --- | --- | --- | --- | --- | --- | --- | --- | --- | --- | --- |
| rs17443815 | chr12:40,190,061-41,190,061 | rs10878245 | 12:40631791_C_T | 9.67E-15 | 0.81 | 0.08 | 0.88 | LRRK2 | synonymous | 0.03 | 10.3 |
| rs17443815 | chr12:40,190,061-41,190,061 | rs954884 | 12:40632958_T_G | 2.79E-14 | 0.81 | 0.08 | 0.89 | LRRK2 | intronic | 0.01 | 3.039 |
| rs17443815 | chr12:40,190,061-41,190,061 | rs10878249 | 12:40633314_C_T | 3.51E-14 | 0.81 | 0.08 | 0.88 | LRRK2 | intronic | 0.01 | 0.542 |
| rs17443815 | chr12:40,190,061-41,190,061 | rs10784444 | 12:40633488_A_G | 3.13E-14 | 0.81 | 0.09 | 0.89 | LRRK2 | intronic | 0.01 | 0.829 |
| rs17443815 | chr12:40,190,061-41,190,061 | rs2404580 | 12:40639561_G_A | 6.12E-14 | 0.81 | 0.09 | 0.89 | LRRK2 | intronic | 0.00 | 3.384 |
| rs17443815 | chr12:40,190,061-41,190,061 | rs732374 | 12:40643468_T_C | 5.34E-14 | 0.81 | 0.07 | 0.88 | LRRK2 | intronic | 0.01 | 12.27 |
| rs17443815 | chr12:40,190,061-41,190,061 | rs10878262 | 12:40644279_C_T | 5.66E-14 | 0.81 | 0.07 | 0.88 | LRRK2 | intronic | 0.01 | 3.373 |
| rs17443815 | chr12:40,190,061-41,190,061 | rs12371502 | 12:40644305_C_T | 3.87E-14 | 0.81 | 0.08 | 0.89 | LRRK2 | intronic | 0.01 | 0.219 |
| rs17443815 | chr12:40,190,061-41,190,061 | rs7955902 | 12:40645257_A_C | 5.46E-14 | 0.81 | 0.07 | 0.88 | LRRK2 | intronic | 0.01 | 11.14 |
| rs17443815 | chr12:40,190,061-41,190,061 | rs11175769 | 12:40656179_G_A | 3.96E-14 | 0.81 | 0.09 | 0.96 | LRRK2 | intronic | 0.01 | 3.625 |
| rs17443815 | chr12:40,190,061-41,190,061 | rs10784461 | 12:40657537_G_A | 5.48E-14 | 0.81 | 0.09 | 0.96 | LRRK2 | intronic | 0.01 | 9.438 |
| rs17443815 | chr12:40,190,061-41,190,061 | rs10784462 | 12:40657771_G_C | 4.95E-14 | 0.81 | 0.09 | 0.96 | LRRK2 | intronic | 0.01 | 1.456 |
| rs17443815 | chr12:40,190,061-41,190,061 | rs11175784 | 12:40659534_T_C | 6.07E-15 | 0.80 | 0.11 | 0.99 | LRRK2 | intronic | 0.04 | 5.528 |
| rs17443815 | chr12:40,190,061-41,190,061 | rs11175787 | 12:40660094_A_C | 7.67E-15 | 0.81 | 0.11 | 0.98 | LRRK2 | intronic | 0.03 | 4.789 |
| rs17443815 | chr12:40,190,061-41,190,061 | rs10784470 | 12:40663596_T_G | 1.15E-14 | 0.81 | 0.10 | 0.98 | LRRK2 | intronic | 0.02 | 0.199 |
| rs17443815 | chr12:40,190,061-41,190,061 | rs12146857 | 12:40663918_G_C | 8.66E-15 | 0.81 | 0.10 | 0.98 | LRRK2 | intronic | 0.03 | 0.719 |
| rs17443815 | chr12:40,190,061-41,190,061 | rs11175836 | 12:40670676_T_C | 1.60E-14 | 0.81 | 0.11 | 0.99 | LRRK2 | intronic | 0.02 | 1.846 |
| rs17443815 | chr12:40,190,061-41,190,061 | rs4272849 | 12:40670801_T_C | 6.12E-15 | 0.80 | 0.12 | 0.99 | LRRK2 | intronic | 0.04 | 0.299 |
| rs17443815 | chr12:40,190,061-41,190,061 | rs7309197 | 12:40673260_A_T | 6.87E-15 | 0.81 | 0.12 | 1.00 | LRRK2 | intronic | 0.04 | 0.828 |
| rs17443815 | chr12:40,190,061-41,190,061 | rs11175845 | 12:40675052_G_A | 6.19E-15 | 0.80 | 0.12 | 1.00 | LRRK2 | intronic | 0.04 | 6.862 |
| rs17443815 | chr12:40,190,061-41,190,061 | rs11175847 | 12:40676200_T_G | 5.78E-15 | 0.80 | 0.12 | 1.00 | LRRK2 | intronic | 0.05 | 0.373 |
| rs17443815 | chr12:40,190,061-41,190,061 | rs17519846 | 12:40678341_T_C | 6.93E-15 | 0.81 | 0.12 | 1.00 | LRRK2 | intronic | 0.04 | 1.311 |
| rs17443815 | chr12:40,190,061-41,190,061 | rs11175862 | 12:40679269_A_T | 1.65E-14 | 0.81 | 0.11 | 0.99 | LRRK2 | intronic | 0.02 | 0.919 |
| rs17443815 | chr12:40,190,061-41,190,061 | rs7957754 | 12:40686038_T_G | 4.41E-15 | 0.80 | 0.13 | 1.00 | LRRK2 | intronic | 0.06 | 2.055 |
| rs17443815 | chr12:40,190,061-41,190,061 | rs7973479 | 12:40686323_C_A | 4.41E-15 | 0.80 | 0.13 | 1.00 | LRRK2 | intronic | 0.06 | 0.709 |
| rs17443815 | chr12:40,190,061-41,190,061 | rs11175911 | 12:40686731_G_A | 4.41E-15 | 0.80 | 0.13 | 1.00 | LRRK2 | intronic | 0.06 | 2.897 |
| rs17443815 | chr12:40,190,061-41,190,061 | rs12369882 | 12:40686845_G_A | 4.41E-15 | 0.80 | 0.13 | 1.00 | LRRK2 | intronic | 0.06 | 10.42 |
| rs17443815 | chr12:40,190,061-41,190,061 | rs12820920 | 12:40688081_G_A | 3.55E-15 | 0.80 | 0.14 | 1.00 | LRRK2 | intronic | 0.07 | 2.036 |
| rs17443815 | chr12:40,190,061-41,190,061 | rs11175922 | 12:40689588_T_C | 5.45E-15 | 0.80 | 0.12 | 1.00 | LRRK2 | intronic | 0.05 | 1.845 |
| rs17443815 | chr12:40,190,061-41,190,061 | rs17443815 | 12:40690061_G_A | 3.29E-15 | 0.80 | 0.13 | 1.00 | LRRK2 | intronic | 0.08 | 5.899 |
| rs17443815 | chr12:40,190,061-41,190,061 | rs7962011 | 12:40692543_T_C | 8.62E-15 | 0.81 | 0.14 | 0.99 | LRRK2 | intronic | 0.03 | 5.104 |
| rs17443815 | chr12:40,190,061-41,190,061 | rs35124455 | 12:40695791_G_T | 7.15E-14 | 0.81 | 0.09 | 0.97 | LRRK2 | intronic | 0.00 | 6.37 |
| rs17443815 | chr12:40,190,061-41,190,061 | rs17444124 | 12:40724494_C_A | 8.27E-14 | 0.81 | 0.06 | 0.97 | LRRK2 | intronic | 0.00 | 5.268 |
| rs10877013 | chr12:57,665,085-58,665,085 | rs2069506 | 12:58142854_C_A | 1.42E-08 | 0.86 | 0.83 | 0.85 | CDK4 | intronic | 0.01 | 6.844 |
| rs10877013 | chr12:57,665,085-58,665,085 | rs2069502 | 12:58144665_C_T | 1.19E-08 | 0.86 | 0.81 | 0.87 | CDK4 | intronic | 0.01 | 11.87 |
| rs10877013 | chr12:57,665,085-58,665,085 | rs2072052 | 12:58146719_A_C | 1.14E-08 | 0.86 | 0.82 | 0.87 | 554bp 5' of CDK4 | . | 0.01 | 12.66 |
| rs10877013 | chr12:57,665,085-58,665,085 | rs4646536 | 12:58157988_A_G | 2.43E-09 | 0.86 | 0.59 | 0.92 | CYP27B1 | intronic | 0.06 | 9.687 |
| rs10877013 | chr12:57,665,085-58,665,085 | rs3782130 | 12:58161898_G_C | 3.90E-09 | 0.86 | 0.64 | 0.92 | 451bp 3' of METTL1 | . | 0.04 | 1.597 |
| rs10877013 | chr12:57,665,085-58,665,085 | rs10877012 | 12:58162085_G_T | 3.65E-09 | 0.86 | 0.64 | 0.92 | 264bp 3' of METTL1 | . | 0.04 | 2.607 |
| rs10877013 | chr12:57,665,085-58,665,085 | rs703842 | 12:58162739_A_G | 2.74E-09 | 0.86 | 0.66 | 0.92 | METTL1 | 3'-UTR | 0.05 | 7.373 |
| rs10877013 | chr12:57,665,085-58,665,085 | rs10877013 | 12:58165085_C_T | 1.93E-09 | 0.85 | 0.63 | 1.00 | METTL1 | intronic | 0.07 | 3.3 |
| rs10877013 | chr12:57,665,085-58,665,085 | rs2291617 | 12:58166403_T_G | 2.49E-09 | 0.86 | 0.64 | 0.93 | METTL21B | 5'-UTR | 0.06 | 12.19 |
| rs10877013 | chr12:57,665,085-58,665,085 | rs10877014 | 12:58167661_A_G | 3.14E-09 | 0.86 | 0.63 | 0.93 | METTL21B | intronic | 0.05 | 7.547 |
| rs10877013 | chr12:57,665,085-58,665,085 | rs10877015 | 12:58167788_A_G | 4.60E-09 | 0.86 | 0.63 | 0.92 | METTL21B | intronic | 0.03 | 12.51 |
| rs10877013 | chr12:57,665,085-58,665,085 | rs10877016 | 12:58167992_A_G | 2.92E-09 | 0.86 | 0.62 | 0.93 | METTL21B | intronic | 0.05 | 1.094 |
| rs10877013 | chr12:57,665,085-58,665,085 | rs10877017 | 12:58169361_T_C | 4.61E-09 | 0.86 | 0.65 | 0.92 | METTL21B | intronic | 0.03 | 2.73 |
| rs10877013 | chr12:57,665,085-58,665,085 | rs11172333 | 12:58170335_A_G | 5.45E-09 | 0.86 | 0.63 | 0.92 | METTL21B | intronic | 0.03 | 11.16 |
| rs10877013 | chr12:57,665,085-58,665,085 | rs9652011 | 12:58170556_T_C | 3.73E-09 | 0.86 | 0.64 | 0.92 | METTL21B | intronic | 0.04 | 5.332 |
| rs10877013 | chr12:57,665,085-58,665,085 | rs10877018 | 12:58171726_T_C | 3.73E-09 | 0.86 | 0.64 | 0.92 | METTL21B | intronic | 0.04 | 0.36 |
| rs10877013 | chr12:57,665,085-58,665,085 | rs10877019 | 12:58172929_C_T | 5.45E-09 | 0.86 | 0.63 | 0.92 | METTL21B | intronic | 0.03 | 3.908 |
| rs10877013 | chr12:57,665,085-58,665,085 | rs8181644 | 12:58173466_T_C | 3.95E-09 | 0.86 | 0.63 | 0.92 | METTL21B | intronic | 0.04 | 7.162 |
| rs10877013 | chr12:57,665,085-58,665,085 | rs923829 | 12:58174306_T_C | 3.95E-09 | 0.86 | 0.63 | 0.92 | METTL21B | synonymous | 0.04 | 6.375 |
| rs10877013 | chr12:57,665,085-58,665,085 | rs11172335 | 12:58175201_C_T | 6.19E-09 | 0.86 | 0.64 | 0.92 | METTL21B | 3'-UTR | 0.02 | 4.256 |
| rs10877013 | chr12:57,665,085-58,665,085 | rs10747783 | 12:58176614_T_C | 1.24E-08 | 0.86 | 0.56 | 0.92 | TSFM | synonymous | 0.01 | 9.58 |
| rs10877013 | chr12:57,665,085-58,665,085 | rs2014886 | 12:58177437_C_T | 1.59E-08 | 0.86 | 0.57 | 0.91 | TSFM | intronic | 0.01 | 9.534 |
| rs10877013 | chr12:57,665,085-58,665,085 | rs1599932 | 12:58177943_G_A | 1.68E-08 | 0.86 | 0.58 | 0.91 | TSFM | intronic | 0.01 | 5.455 |
| rs10877013 | chr12:57,665,085-58,665,085 | rs10431552 | 12:58178148_G_A | 1.25E-08 | 0.86 | 0.57 | 0.92 | TSFM | intronic | 0.01 | 0.915 |
| rs10877013 | chr12:57,665,085-58,665,085 | rs6581155 | 12:58178162_A_G | 1.17E-08 | 0.86 | 0.57 | 0.92 | TSFM | intronic | 0.01 | 0.142 |
| rs10877013 | chr12:57,665,085-58,665,085 | rs10431505 | 12:58178589_A_G | 1.17E-08 | 0.86 | 0.57 | 0.92 | TSFM | intronic | 0.01 | 1.904 |
| rs10877013 | chr12:57,665,085-58,665,085 | rs56177740 | 12:58182312_C_T | 1.68E-08 | 0.86 | 0.58 | 0.91 | TSFM | intronic | 0.01 | 0.233 |
| rs10877013 | chr12:57,665,085-58,665,085 | rs4301823 | 12:58186594_C_T | 1.76E-08 | 0.86 | 0.58 | 0.91 | . | . | 0.01 | 0.444 |
| rs10877013 | chr12:57,665,085-58,665,085 | rs7965287 | 12:58188696_G_C | 1.33E-08 | 0.86 | 0.58 | 0.92 | TSFM | intronic | 0.01 | 0.673 |
| rs10877013 | chr12:57,665,085-58,665,085 | rs11172344 | 12:58193448_T_C | 1.18E-08 | 0.86 | 0.56 | 0.91 | AVIL | intronic | 0.01 | 7.857 |
| rs10877013 | chr12:57,665,085-58,665,085 | rs10783847 | 12:58196447_G_A | 1.26E-08 | 0.86 | 0.57 | 0.91 | AVIL | 3'-UTR | 0.01 | 2.781 |
| rs10877013 | chr12:57,665,085-58,665,085 | rs10783848 | 12:58196528_G_A | 1.26E-08 | 0.86 | 0.57 | 0.91 | AVIL | 3'-UTR | 0.01 | 9.535 |
| rs10877013 | chr12:57,665,085-58,665,085 | rs1875124 | 12:58196802_A_G | 1.68E-08 | 0.86 | 0.56 | 0.91 | AVIL | intronic | 0.01 | 6.567 |
| rs10877013 | chr12:57,665,085-58,665,085 | rs1021469 | 12:58208164_G_A | 3.98E-09 | 0.86 | 0.87 | 0.75 | AVIL | intronic | 0.04 | 3.241 |
| rs10877013 | chr12:57,665,085-58,665,085 | rs11172350 | 12:58213272_A_G | 1.02E-08 | 0.86 | 0.49 | 0.91 | 436bp 3' of CTDSP2 | . | 0.02 | 0.155 |
| rs117618569 | chr15:74,587,571-75,587,571 | rs117429208 | 15:75058402_T_C | 1.79E-10 | 1.20 | 0.36 | 0.73 | 9.5kb 3' of CYP1A2 | . | 0.07 | 6.457 |
| rs117618569 | chr15:74,587,571-75,587,571 | rs4488423 | 15:75059387_G_T | 1.38E-10 | 1.20 | 0.37 | 0.73 | 10kb 3' of CYP1A2 | . | 0.09 | 2.197 |
| rs117618569 | chr15:74,587,571-75,587,571 | rs12437562 | 15:75060346_G_C | 1.65E-10 | 1.20 | 0.34 | 0.73 | 11kb 3' of CYP1A2 | . | 0.07 | 0.741 |
| rs117618569 | chr15:74,587,571-75,587,571 | rs16972381 | 15:75061524_A_G | 2.29E-10 | 1.20 | 0.35 | 0.73 | 13kb 3' of CYP1A2 | . | 0.05 | 2.55 |
| rs117618569 | chr15:74,587,571-75,587,571 | rs59567621 | 15:75062405_C_T | 2.47E-10 | 1.20 | 0.35 | 0.73 | 12kb 5' of CSK | . | 0.05 | 0.761 |
| rs117618569 | chr15:74,587,571-75,587,571 | rs58322828 | 15:75063742_G_A | 3.75E-10 | 1.20 | 0.36 | 0.73 | 11kb 5' of CSK | . | 0.03 | 11.39 |
| rs117618569 | chr15:74,587,571-75,587,571 | rs16972486 | 15:75066008_T_C | 2.09E-10 | 1.20 | 0.42 | 0.77 | 8.4kb 5' of CSK | . | 0.06 | 3.769 |
| rs117618569 | chr15:74,587,571-75,587,571 | rs56992651 | 15:75067012_T_C | 2.48E-10 | 1.20 | 0.42 | 0.77 | 7.4kb 5' of CSK | . | 0.05 | 0.01 |
| rs117618569 | chr15:74,587,571-75,587,571 | rs58050869 | 15:75067625_T_C | 2.48E-10 | 1.20 | 0.42 | 0.77 | 6.8kb 5' of CSK | . | 0.05 | 2.228 |
| rs117618569 | chr15:74,587,571-75,587,571 | rs35290121 | 15:75073121_A_T | 1.16E-10 | 1.22 | 0.41 | 0.97 | 1.3kb 5' of CSK | . | 0.10 | 6.761 |
| rs117618569 | chr15:74,587,571-75,587,571 | rs16972628 | 15:75080456_G_A | 8.48E-11 | 1.22 | 0.33 | 1.00 | CSK | intronic | 0.14 | 2.495 |
| rs117618569 | chr15:74,587,571-75,587,571 | rs117618569 | 15:75087571_A_T | 7.70E-11 | 1.22 | 0.33 | 1.00 | CSK | intronic | 0.15 | 2.408 |
| rs117618569 | chr15:74,587,571-75,587,571 | rs2229729 | 15:75091664_A_G | 2.42E-10 | 1.22 | 0.34 | 0.99 | CSK | synonymous | 0.05 | 8.533 |
| rs3762318 | chr1:67,097,119-68,097,119 | rs3762318 | 1:67597119_G_A | 2.15E-17 | 0.69 | 0.44 | 1.00 | 2.9kb 5' of C1orf141 | . | 0.31 | 0.496 |
| rs3762318 | chr1:67,097,119-68,097,119 | rs2024825 | 1:67597977_T_C | 3.50E-16 | 0.69 | 0.35 | 0.95 | 3.8kb 5' of C1orf141 | . | 0.02 | 3.378 |
| rs3762318 | chr1:67,097,119-68,097,119 | rs12563505 | 1:67598170_T_C | 8.35E-17 | 0.69 | 0.52 | 0.94 | 3.9kb 5' of C1orf141 | . | 0.08 | 2.188 |
| rs3762318 | chr1:67,097,119-68,097,119 | rs114661385 | 1:67599425_A_G | 5.17E-16 | 0.69 | 0.33 | 0.89 | 5.2kb 5' of C1orf141 | . | 0.01 | 2.497 |
| rs3762318 | chr1:67,097,119-68,097,119 | rs12069782 | 1:67600101_C_T | 2.45E-16 | 0.69 | 0.27 | 0.94 | 5.9kb 5' of C1orf141 | . | 0.03 | 1.44 |
| rs3762318 | chr1:67,097,119-68,097,119 | rs12095536 | 1:67600142_T_C | 2.62E-16 | 0.69 | 0.24 | 0.94 | 5.9kb 5' of C1orf141 | . | 0.03 | 4.932 |
| rs3762318 | chr1:67,097,119-68,097,119 | rs12090164 | 1:67600163_G_A | 2.62E-16 | 0.69 | 0.24 | 0.94 | 5.9kb 5' of C1orf141 | . | 0.03 | 1.729 |
| rs3762318 | chr1:67,097,119-68,097,119 | rs78865162 | 1:67600929_T_C | 4.00E-16 | 0.69 | 0.30 | 0.89 | 6.7kb 5' of C1orf141 | . | 0.02 | 0.864 |
| rs3762318 | chr1:67,097,119-68,097,119 | rs115776568 | 1:67601074_G_A | 4.00E-16 | 0.69 | 0.30 | 0.89 | 6.9kb 5' of C1orf141 | . | 0.02 | 3.996 |
| rs3762318 | chr1:67,097,119-68,097,119 | rs114370420 | 1:67601084_A_G | 4.00E-16 | 0.69 | 0.30 | 0.89 | 6.9kb 5' of C1orf141 | . | 0.02 | 1.34 |
| rs3762318 | chr1:67,097,119-68,097,119 | rs78917656 | 1:67601328_G_T | 4.00E-16 | 0.69 | 0.30 | 0.89 | 7.1kb 5' of C1orf141 | . | 0.02 | 0.832 |
| rs3762318 | chr1:67,097,119-68,097,119 | rs78597810 | 1:67601412_C_T | 3.15E-16 | 0.68 | 0.29 | 0.88 | 7.2kb 5' of C1orf141 | . | 0.02 | 1.959 |
| rs3762318 | chr1:67,097,119-68,097,119 | rs12568393 | 1:67602139_A_G | 4.00E-16 | 0.69 | 0.30 | 0.89 | 7.9kb 5' of C1orf141 | . | 0.02 | 1.152 |
| rs3762318 | chr1:67,097,119-68,097,119 | rs12561798 | 1:67602164_C_T | 4.00E-16 | 0.69 | 0.30 | 0.89 | 7.9kb 5' of C1orf141 | . | 0.02 | 8.197 |
| rs3762318 | chr1:67,097,119-68,097,119 | rs76436269 | 1:67602996_A_G | 4.00E-16 | 0.69 | 0.30 | 0.89 | 8.8kb 5' of C1orf141 | . | 0.02 | 0.895 |
| rs3762318 | chr1:67,097,119-68,097,119 | rs6693659 | 1:67604615_A_C | 3.53E-16 | 0.68 | 0.20 | 0.87 | 10kb 5' of C1orf141 | . | 0.02 | 1.688 |
| rs3762318 | chr1:67,097,119-68,097,119 | rs78377598 | 1:67612502_T_C | 6.55E-17 | 0.68 | 0.21 | 0.91 | 18kb 5' of C1orf141 | . | 0.10 | 2.253 |
| rs3762318 | chr1:67,097,119-68,097,119 | rs12564219 | 1:67613252_G_A | 6.55E-17 | 0.68 | 0.21 | 0.91 | 19kb 5' of IL23R | . | 0.10 | 0.235 |
| rs3762318 | chr1:67,097,119-68,097,119 | rs12566159 | 1:67613730_T_C | 1.99E-16 | 0.69 | 0.17 | 0.91 | 18kb 5' of IL23R | . | 0.04 | 0.165 |
| rs3762318 | chr1:67,097,119-68,097,119 | rs117282985 | 1:67626416_C_G | 2.19E-16 | 0.69 | 0.22 | 0.90 | 5.8kb 5' of IL23R | . | 0.03 | 0.402 |
| rs12144914 | chr1:151,289,647-152,289,647 | rs12144914 | 1:151789647_G_T | 4.70E-08 | 1.14 | 0.72 | 1.00 | RORC | intronic | 0.39 | 1.47 |
| rs12144914 | chr1:151,289,647-152,289,647 | rs7540034 | 1:151790339_C_G | 1.61E-06 | 1.13 | 0.63 | 0.89 | RORC | intronic | 0.01 | 0.148 |
| rs12144914 | chr1:151,289,647-152,289,647 | rs6693413 | 1:151792310_A_G | 1.57E-06 | 1.13 | 0.62 | 0.89 | RORC | intronic | 0.02 | 4.89 |
| rs12144914 | chr1:151,289,647-152,289,647 | rs949969 | 1:151792814_A_G | 1.08E-07 | 0.87 | 0.66 | 0.57 | RORC | intronic | 0.18 | 11.81 |
| rs12144914 | chr1:151,289,647-152,289,647 | rs61815119 | 1:151794100_G_A | 1.12E-07 | 0.88 | 0.76 | 0.63 | RORC | intronic | 0.17 | 4.164 |
| rs12144914 | chr1:151,289,647-152,289,647 | rs7531041 | 1:151794412_A_C | 1.28E-06 | 1.13 | 0.62 | 0.89 | RORC | intronic | 0.02 | 2.066 |
| rs12144914 | chr1:151,289,647-152,289,647 | rs6587620 | 1:151794794_A_G | 6.76E-07 | 1.13 | 0.66 | 0.88 | RORC | intronic | 0.03 | 7.248 |
| rs12144914 | chr1:151,289,647-152,289,647 | rs11204895 | 1:151795497_C_G | 4.36E-07 | 1.13 | 0.71 | 0.88 | RORC | intronic | 0.05 | 0.908 |
| rs12144914 | chr1:151,289,647-152,289,647 | rs76990585 | 1:151795777_T_C | 9.85E-07 | 0.88 | 0.81 | 0.53 | RORC | intronic | 0.02 | 8.204 |
| rs12144914 | chr1:151,289,647-152,289,647 | rs12036145 | 1:151795866_T_C | 3.93E-07 | 1.13 | 0.74 | 0.88 | RORC | intronic | 0.05 | 4.782 |
| rs17027258 | chr2:102,591,540-103,591,540 | rs56258475 | 2:102999312_G_A | 1.48E-16 | 1.24 | 0.24 | 0.95 | IL18R1 | intronic | 0.01 | 5.16 |
| rs17027258 | chr2:102,591,540-103,591,540 | rs4851570 | 2:103006387_G_A | 3.06E-16 | 1.23 | 0.22 | 0.95 | IL18R1 | intronic | 0.00 | 1.259 |
| rs17027258 | chr2:102,591,540-103,591,540 | rs17027060 | 2:103007567_C_T | 3.06E-16 | 1.23 | 0.22 | 0.95 | IL18R1 | intronic | 0.00 | 6.515 |
| rs17027258 | chr2:102,591,540-103,591,540 | rs17027087 | 2:103015918_T_C | 7.17E-17 | 1.24 | 0.23 | 0.97 | 700bp 3' of IL18R1 | . | 0.01 | 0.388 |
| rs17027258 | chr2:102,591,540-103,591,540 | rs3732123 | 2:103018077_G_C | 7.79E-17 | 1.24 | 0.24 | 0.97 | 2.9kb 3' of IL18R1 | . | 0.01 | 1.393 |
| rs17027258 | chr2:102,591,540-103,591,540 | rs55742125 | 2:103019782_A_G | 7.17E-17 | 1.24 | 0.23 | 0.97 | 4.6kb 3' of IL18R1 | . | 0.01 | 1.571 |
| rs17027258 | chr2:102,591,540-103,591,540 | rs55883125 | 2:103024331_A_G | 1.15E-16 | 1.24 | 0.22 | 0.98 | 9.1kb 3' of IL18R1 | . | 0.01 | 0.395 |
| rs17027258 | chr2:102,591,540-103,591,540 | rs4851006 | 2:103024738_A_T | 1.14E-16 | 1.24 | 0.22 | 0.98 | 9.5kb 3' of IL18R1 | . | 0.01 | 0.048 |
| rs17027258 | chr2:102,591,540-103,591,540 | rs11693955 | 2:103029165_T_A | 8.60E-17 | 1.24 | 0.24 | 0.98 | 6.1kb 5' of IL18RAP | . | 0.01 | 8.796 |
| rs17027258 | chr2:102,591,540-103,591,540 | rs3771156 | 2:103036677_T_C | 8.91E-17 | 1.24 | 0.26 | 0.98 | IL18RAP | intronic | 0.01 | 6.375 |
| rs17027258 | chr2:102,591,540-103,591,540 | rs887972 | 2:103040945_A_G | 9.65E-17 | 1.24 | 0.29 | 0.98 | IL18RAP | intronic | 0.01 | 2.728 |
| rs17027258 | chr2:102,591,540-103,591,540 | rs887971 | 2:103041167_C_T | 4.98E-17 | 1.24 | 0.30 | 0.98 | IL18RAP | intronic | 0.02 | 2.952 |
| rs17027258 | chr2:102,591,540-103,591,540 | rs11678975 | 2:103043739_A_T | 2.02E-17 | 1.24 | 0.30 | 0.99 | IL18RAP | intronic | 0.05 | 3.018 |
| rs17027258 | chr2:102,591,540-103,591,540 | rs66566526 | 2:103047802_T_C | 3.05E-17 | 1.24 | 0.22 | 0.99 | IL18RAP | intronic | 0.03 | 3.08 |
| rs17027258 | chr2:102,591,540-103,591,540 | rs56331791 | 2:103048157_A_G | 5.42E-17 | 1.24 | 0.23 | 0.99 | IL18RAP | intronic | 0.02 | 0.856 |
| rs17027258 | chr2:102,591,540-103,591,540 | rs11681718 | 2:103051144_G_A | 4.64E-17 | 1.24 | 0.23 | 0.99 | IL18RAP | intronic | 0.02 | 3.666 |
| rs17027258 | chr2:102,591,540-103,591,540 | rs4851582 | 2:103051558_C_T | 6.64E-17 | 1.24 | 0.19 | 0.99 | IL18RAP | intronic | 0.02 | 0.893 |
| rs17027258 | chr2:102,591,540-103,591,540 | rs17027166 | 2:103055420_A_G | 4.47E-17 | 1.24 | 0.21 | 0.99 | IL18RAP | intronic | 0.02 | 1.454 |
| rs17027258 | chr2:102,591,540-103,591,540 | rs55645612 | 2:103055661_T_C | 4.47E-17 | 1.24 | 0.21 | 0.99 | IL18RAP | intronic | 0.02 | 1.272 |
| rs17027258 | chr2:102,591,540-103,591,540 | rs56166614 | 2:103056096_G_A | 1.00E-16 | 1.24 | 0.19 | 0.98 | IL18RAP | intronic | 0.01 | 4.505 |
| rs17027258 | chr2:102,591,540-103,591,540 | rs10490204 | 2:103056534_C_A | 5.18E-17 | 1.24 | 0.24 | 0.99 | IL18RAP | intronic | 0.02 | 1.637 |
| rs17027258 | chr2:102,591,540-103,591,540 | rs17027179 | 2:103057159_T_C | 4.47E-17 | 1.24 | 0.21 | 0.99 | IL18RAP | intronic | 0.02 | 3.952 |
| rs17027258 | chr2:102,591,540-103,591,540 | rs11123927 | 2:103058803_T_C | 4.05E-17 | 1.24 | 0.21 | 0.99 | IL18RAP | intronic | 0.02 | 1.9 |
| rs17027258 | chr2:102,591,540-103,591,540 | rs10490203 | 2:103059237_G_T | 5.76E-17 | 1.24 | 0.21 | 0.99 | IL18RAP | intronic | 0.02 | 0.396 |
| rs17027258 | chr2:102,591,540-103,591,540 | rs4851583 | 2:103060300_C_T | 4.05E-17 | 1.24 | 0.21 | 0.99 | IL18RAP | intronic | 0.02 | 6.369 |
| rs17027258 | chr2:102,591,540-103,591,540 | rs3771150 | 2:103060851_A_G | 4.20E-17 | 1.24 | 0.24 | 0.99 | IL18RAP | intronic | 0.02 | 4.751 |
| rs17027258 | chr2:102,591,540-103,591,540 | rs11694360 | 2:103061147_A_G | 4.05E-17 | 1.24 | 0.21 | 0.99 | IL18RAP | intronic | 0.02 | 5.232 |
| rs17027258 | chr2:102,591,540-103,591,540 | rs11123928 | 2:103061286_A_G | 4.20E-17 | 1.24 | 0.24 | 0.99 | IL18RAP | intronic | 0.02 | 0.103 |
| rs17027258 | chr2:102,591,540-103,591,540 | rs7597017 | 2:103062116_G_A | 5.76E-17 | 1.24 | 0.21 | 0.99 | IL18RAP | intronic | 0.02 | 5.234 |
| rs17027258 | chr2:102,591,540-103,591,540 | rs4851585 | 2:103062754_A_T | 4.05E-17 | 1.24 | 0.21 | 0.99 | IL18RAP | intronic | 0.02 | 0.007 |
| rs17027258 | chr2:102,591,540-103,591,540 | rs56044378 | 2:103065367_A_G | 2.85E-17 | 1.24 | 0.21 | 0.99 | IL18RAP | intronic | 0.03 | 0.417 |
| rs17027258 | chr2:102,591,540-103,591,540 | rs11123929 | 2:103067143_A_G | 7.34E-17 | 1.24 | 0.23 | 0.99 | IL18RAP | intronic | 0.01 | 0.989 |
| rs17027258 | chr2:102,591,540-103,591,540 | rs56117144 | 2:103074651_C_T | 5.42E-17 | 1.24 | 0.23 | 1.00 | 5.6kb 3' of IL18RAP | . | 0.02 | 3.904 |
| rs17027258 | chr2:102,591,540-103,591,540 | rs57081652 | 2:103075381_C_G | 4.89E-17 | 1.24 | 0.23 | 1.00 | 6.4kb 3' of IL18RAP | . | 0.02 | 1.403 |
| rs17027258 | chr2:102,591,540-103,591,540 | rs17027230 | 2:103079330_T_C | 4.73E-17 | 1.24 | 0.20 | 0.99 | 10kb 3' of IL18RAP | . | 0.02 | 0.527 |
| rs17027258 | chr2:102,591,540-103,591,540 | rs56043441 | 2:103087383_T_C | 1.95E-17 | 1.25 | 0.24 | 1.00 | 2.4kb 5' of SLC9A4 | . | 0.05 | 0.24 |
| rs17027258 | chr2:102,591,540-103,591,540 | rs4140785 | 2:103088517_C_A | 1.96E-17 | 1.25 | 0.24 | 1.00 | 1.2kb 5' of SLC9A4 | . | 0.05 | 3.247 |
| rs17027258 | chr2:102,591,540-103,591,540 | rs4851011 | 2:103089678_T_C | 2.10E-17 | 1.25 | 0.25 | 1.00 | 82bp 5' of SLC9A4 | . | 0.05 | 13.9 |
| rs17027258 | chr2:102,591,540-103,591,540 | rs17027255 | 2:103090127_T_C | 1.33E-17 | 1.25 | 0.21 | 1.00 | SLC9A4 | 5'-UTR | 0.07 | 6.864 |
| rs17027258 | chr2:102,591,540-103,591,540 | rs17027258 | 2:103091540_G_A | 1.11E-17 | 1.25 | 0.24 | 1.00 | SLC9A4 | intronic | 0.09 | 13.19 |
| rs56167332 | chr5:158,327,769-159,327,769 | rs6871626 | 5:158826792_A_C | 1.58E-15 | 0.81 | 0.62 | 0.99 | 37kb 3' of LOC285626 | . | 0.24 | 1.191 |
| rs56167332 | chr5:158,327,769-159,327,769 | rs56167332 | 5:158827769_A_C | 9.19E-16 | 0.80 | 0.67 | 1.00 | 38kb 3' of LOC285626 | . | 0.41 | 4.742 |
| rs56167332 | chr5:158,327,769-159,327,769 | rs755374 | 5:158829294_T_C | 1.11E-15 | 0.80 | 0.64 | 0.98 | 39kb 3' of LOC285626 | . | 0.34 | 3.265 |
| rs74468352 | chr6:146,420,271-147,420,271 | rs71566446 | 6:146897206_G_A | 1.13E-14 | 1.25 | 0.02 | 0.97 | 21kb 3' of RAB32 | . | 0.05 | 9.12 |
| rs74468352 | chr6:146,420,271-147,420,271 | rs13220141 | 6:146897633_T_A | 7.50E-15 | 1.25 | 0.03 | 0.97 | 22kb 3' of RAB32 | . | 0.07 | 6.865 |
| rs74468352 | chr6:146,420,271-147,420,271 | rs13215778 | 6:146900563_C_T | 7.26E-15 | 1.25 | 0.03 | 0.98 | 20kb 5' of C6orf103 | . | 0.07 | 4.081 |
| rs74468352 | chr6:146,420,271-147,420,271 | rs6906530 | 6:146902340_C_G | 7.85E-15 | 1.25 | 0.03 | 0.98 | 18kb 5' of C6orf103 | . | 0.06 | 0.668 |
| rs74468352 | chr6:146,420,271-147,420,271 | rs76462351 | 6:146906700_T_G | 1.56E-14 | 1.25 | 0.02 | 0.99 | 13kb 5' of C6orf103 | . | 0.03 | 12.02 |
| rs74468352 | chr6:146,420,271-147,420,271 | rs77964037 | 6:146909094_A_C | 1.40E-14 | 1.25 | 0.03 | 0.99 | 11kb 5' of C6orf103 | . | 0.04 | 3.628 |
| rs74468352 | chr6:146,420,271-147,420,271 | rs35192162 | 6:146911668_C_T | 1.42E-14 | 1.25 | 0.03 | 0.99 | 8.5kb 5' of C6orf103 | . | 0.04 | 0.379 |
| rs74468352 | chr6:146,420,271-147,420,271 | rs34267274 | 6:146911692_C_T | 1.42E-14 | 1.25 | 0.03 | 0.99 | 8.4kb 5' of C6orf103 | . | 0.04 | 0.552 |
| rs74468352 | chr6:146,420,271-147,420,271 | rs17076142 | 6:146912807_T_C | 1.42E-14 | 1.25 | 0.03 | 0.99 | 7.3kb 5' of C6orf103 | . | 0.04 | 4.967 |
| rs74468352 | chr6:146,420,271-147,420,271 | rs71566450 | 6:146913437_A_G | 1.26E-14 | 1.25 | 0.03 | 0.99 | 6.7kb 5' of C6orf103 | . | 0.04 | 0.478 |
| rs74468352 | chr6:146,420,271-147,420,271 | rs34321971 | 6:146914837_T_C | 1.26E-14 | 1.25 | 0.03 | 0.99 | 5.3kb 5' of C6orf103 | . | 0.04 | 4.311 |
| rs74468352 | chr6:146,420,271-147,420,271 | rs2275606 | 6:146918950_A_G | 8.33E-15 | 1.25 | 0.03 | 0.99 | 1.2kb 5' of C6orf103 | . | 0.06 | 0.446 |
| rs74468352 | chr6:146,420,271-147,420,271 | rs74468352 | 6:146920271_T_C | 2.48E-15 | 1.26 | 0.07 | 1.00 | C6orf103 | intronic | 0.20 | 7.716 |
| rs74468352 | chr6:146,420,271-147,420,271 | rs75395649 | 6:146921421_A_C | 2.48E-15 | 1.26 | 0.07 | 1.00 | C6orf103 | intronic | 0.20 | 4.643 |
| rs416324 | chr8:90,315,235-91,315,235 | rs405734 | 8:90768439_G_A | 1.21E-28 | 0.76 | 0.26 | 0.96 | 1.5kb 5' of RIPK2 | . | 0.04 | 6.145 |
| rs416324 | chr8:90,315,235-91,315,235 | rs402886 | 8:90772500_T_C | 1.25E-28 | 0.76 | 0.27 | 0.97 | RIPK2 | intronic | 0.04 | 5.658 |
| rs416324 | chr8:90,315,235-91,315,235 | rs39500 | 8:90772665_A_G | 1.25E-28 | 0.76 | 0.27 | 0.97 | RIPK2 | intronic | 0.04 | 4.686 |
| rs416324 | chr8:90,315,235-91,315,235 | rs39761 | 8:90772920_C_T | 1.25E-28 | 0.76 | 0.27 | 0.97 | RIPK2 | intronic | 0.04 | 4.45 |
| rs416324 | chr8:90,315,235-91,315,235 | rs43225 | 8:90773004_G_A | 1.25E-28 | 0.76 | 0.27 | 0.97 | RIPK2 | intronic | 0.04 | 4.156 |
| rs416324 | chr8:90,315,235-91,315,235 | rs40452 | 8:90778159_A_G | 1.22E-28 | 0.76 | 0.27 | 0.97 | RIPK2 | intronic | 0.04 | 6.116 |
| rs416324 | chr8:90,315,235-91,315,235 | rs39503 | 8:90779807_G_A | 1.22E-28 | 0.76 | 0.27 | 0.97 | RIPK2 | intronic | 0.04 | 3.855 |
| rs416324 | chr8:90,315,235-91,315,235 | rs39504 | 8:90782909_C_T | 5.79E-29 | 0.76 | 0.27 | 0.97 | . | . | 0.08 | 2.319 |
| rs416324 | chr8:90,315,235-91,315,235 | rs447618 | 8:90785685_T_C | 1.28E-28 | 0.76 | 0.26 | 0.97 | RIPK2 | intronic | 0.04 | 0.729 |
| rs416324 | chr8:90,315,235-91,315,235 | rs43134 | 8:90787960_G_A | 9.76E-29 | 0.76 | 0.27 | 0.97 | RIPK2 | intronic | 0.05 | 6.692 |
| rs416324 | chr8:90,315,235-91,315,235 | rs40545 | 8:90789711_G_A | 9.76E-29 | 0.76 | 0.27 | 0.97 | RIPK2 | intronic | 0.05 | 2.66 |
| rs416324 | chr8:90,315,235-91,315,235 | rs2735882 | 8:90791611_C_T | 1.79E-28 | 0.76 | 0.27 | 0.97 | RIPK2 | intronic | 0.03 | 0.698 |
| rs416324 | chr8:90,315,235-91,315,235 | rs39505 | 8:90792066_G_A | 1.29E-28 | 0.76 | 0.27 | 0.97 | RIPK2 | intronic | 0.04 | 3.728 |
| rs416324 | chr8:90,315,235-91,315,235 | rs39506 | 8:90792127_C_A | 1.29E-28 | 0.76 | 0.27 | 0.97 | RIPK2 | intronic | 0.04 | 6.089 |
| rs416324 | chr8:90,315,235-91,315,235 | rs40453 | 8:90802045_G_A | 6.93E-29 | 0.76 | 0.30 | 0.98 | RIPK2 | intronic | 0.07 | 2.228 |
| rs416324 | chr8:90,315,235-91,315,235 | rs400411 | 8:90802099_G_A | 6.93E-29 | 0.76 | 0.30 | 0.98 | RIPK2 | intronic | 0.07 | 1.43 |
| rs416324 | chr8:90,315,235-91,315,235 | rs39509 | 8:90804165_A_G | 6.93E-29 | 0.76 | 0.30 | 0.98 | 872bp 3' of RIPK2 | . | 0.07 | 1.294 |
| rs416324 | chr8:90,315,235-91,315,235 | rs411279 | 8:90808157_C_A | 1.22E-28 | 0.76 | 0.30 | 0.97 | 4.9kb 3' of RIPK2 | . | 0.04 | 4.466 |
| rs416324 | chr8:90,315,235-91,315,235 | rs416324 | 8:90815235_A_G | 5.30E-29 | 0.76 | 0.38 | 1.00 | 12kb 3' of RIPK2 | . | 0.09 | 1.121 |
| rs416324 | chr8:90,315,235-91,315,235 | rs465 | 8:90816058_C_G | 1.11E-28 | 0.76 | 0.34 | 1.00 | 13kb 3' of RIPK2 | . | 0.04 | 1.22 |
| rs10817678 | chr9:117,079,504-118,140,404 | rs4366152 | 9:117564875_C_T | 1.38E-31 | 0.75 | 0.02 | 0.98 | TNFSF15 | intronic | 0.06 | 2.756 |
| rs10817678 | chr9:117,079,504-118,140,404 | rs4263839 | 9:117566440_G_A | 9.70E-32 | 0.75 | 0.02 | 0.98 | TNFSF15 | intronic | 0.08 | 1.026 |
| rs10817678 | chr9:117,079,504-118,140,404 | rs6478109 | 9:117568766_G_A | 8.31E-32 | 0.75 | 0.03 | 0.99 | 357bp 5' of TNFSF15 | . | 0.11 | 12.54 |
| rs10817678 | chr9:117,079,504-118,140,404 | rs7848647 | 9:117569046_C_T | 1.02E-31 | 0.75 | 0.03 | 0.99 | 637bp 5' of TNFSF15 | . | 0.09 | 6.363 |
| rs10817678 | chr9:117,079,504-118,140,404 | rs10817678 | 9:117579457_A_G | 1.55E-32 | 0.75 | 0.02 | 1.00 | 11kb 5' of TNFSF15 | . | 0.65 | 1.509 |
| rs10822054 | chr10:63,991,638-64,994,157 | rs10822052 | 10:64455770_A_G | 6.26E-10 | 0.78 | 0.23 | 0.97 | 24kb 3' of ZNF365 | . | 0.01 | 4.699 |
| rs10822054 | chr10:63,991,638-64,994,157 | rs10822053 | 10:64460835_C_T | 1.22E-10 | 0.77 | 0.17 | 0.98 | 29kb 3' of ZNF365 | . | 0.06 | 2.054 |
| rs10822054 | chr10:63,991,638-64,994,157 | rs149339832 | 10:64463836_T_A | 1.41E-10 | 0.77 | 0.18 | 0.98 | 32kb 3' of ZNF365 | . | 0.05 | 0.893 |
| rs10822054 | chr10:63,991,638-64,994,157 | rs11597299 | 10:64466275_A_G | 1.57E-10 | 0.77 | 0.18 | 0.99 | 35kb 3' of ZNF365 | . | 0.04 | 1.76 |
| rs10822054 | chr10:63,991,638-64,994,157 | rs11599754 | 10:64466651_C_T | 1.57E-10 | 0.77 | 0.18 | 0.99 | 35kb 3' of ZNF365 | . | 0.04 | 15.8 |
| rs10822054 | chr10:63,991,638-64,994,157 | rs10995282 | 10:64471291_C_G | 7.68E-11 | 0.77 | 0.26 | 1.00 | 40kb 3' of ZNF365 | . | 0.09 | 2.638 |
| rs10822054 | chr10:63,991,638-64,994,157 | rs10995283 | 10:64474388_A_G | 1.03E-10 | 0.76 | 0.30 | 0.90 | 43kb 3' of ZNF365 | . | 0.06 | 6.565 |
| rs10822054 | chr10:63,991,638-64,994,157 | rs10822054 | 10:64480252_T_G | 6.98E-11 | 0.77 | 0.27 | 1.00 | 48kb 3' of ZNF365 | . | 0.09 | 0.079 |
| rs10822054 | chr10:63,991,638-64,994,157 | rs7084016 | 10:64480441_T_C | 6.98E-11 | 0.77 | 0.27 | 1.00 | 49kb 3' of ZNF365 | . | 0.09 | 2.219 |
| rs10822054 | chr10:63,991,638-64,994,157 | rs7088592 | 10:64481290_G_A | 5.08E-10 | 0.78 | 0.21 | 0.97 | 50kb 3' of ZNF365 | . | 0.02 | 3.382 |
| rs10822054 | chr10:63,991,638-64,994,157 | rs10995294 | 10:64482917_A_C | 8.68E-11 | 0.77 | 0.24 | 1.00 | 51kb 3' of ZNF365 | . | 0.08 | 0.744 |
| rs10822054 | chr10:63,991,638-64,994,157 | rs7895364 | 10:64484813_C_G | 4.37E-10 | 0.78 | 0.19 | 0.96 | 53kb 3' of ZNF365 | . | 0.02 | 1.043 |
| rs10822054 | chr10:63,991,638-64,994,157 | rs10995299 | 10:64504203_T_C | 3.13E-10 | 0.77 | 0.26 | 0.96 | 60kb 5' of ADO | . | 0.03 | 11.17 |
| rs10822054 | chr10:63,991,638-64,994,157 | rs10995301 | 10:64507295_G_C | 3.15E-10 | 0.77 | 0.24 | 0.96 | 57kb 5' of ADO | . | 0.03 | 2.8 |
| rs10822054 | chr10:63,991,638-64,994,157 | rs10822057 | 10:64520151_A_G | 3.20E-10 | 0.79 | 0.14 | 0.77 | 44kb 5' of ADO | . | 0.02 | 8.679 |
| rs10822054 | chr10:63,991,638-64,994,157 | rs10822058 | 10:64523582_T_A | 4.03E-10 | 0.80 | 0.16 | 0.77 | 41kb 5' of ADO | . | 0.02 | 7.898 |
| rs10822054 | chr10:63,991,638-64,994,157 | rs224046 | 10:64527757_G_C | 1.17E-10 | 0.78 | 0.15 | 0.65 | 37kb 5' of ADO | . | 0.04 | 0.502 |
| rs10822054 | chr10:63,991,638-64,994,157 | rs11592442 | 10:64533904_A_T | 1.76E-10 | 0.78 | 0.20 | 0.64 | 31kb 5' of ADO | . | 0.03 | 9.921 |
| rs10822054 | chr10:63,991,638-64,994,157 | rs11527181 | 10:64546164_G_A | 1.77E-10 | 0.78 | 0.19 | 0.63 | 18kb 5' of ADO | . | 0.03 | 2.548 |
| rs10822054 | chr10:63,991,638-64,994,157 | rs12220700 | 10:64561506_G_A | 3.07E-10 | 0.78 | 0.11 | 0.56 | 3kb 5' of ADO | . | 0.02 | 3.827 |
| rs10822054 | chr10:63,991,638-64,994,157 | rs2236295 | 10:64564892_T_G | 4.93E-10 | 0.78 | 0.11 | 0.53 | ADO | missense | 0.02 | 23.6 |
| rs10822054 | chr10:63,991,638-64,994,157 | rs10995311 | 10:64564934_G_C | 1.07E-10 | 0.78 | 0.14 | 0.56 | ADO | missense | 0.06 | 14.3 |
| rs80161952 | chr10:63,991,638-64,994,157 | rs12248559 | 10:64451153_A_T | 3.14E-08 | 1.22 | 0.68 | 0.36 | 19kb 3' of ZNF365 | . | 0.02 | 7.135 |
| rs80161952 | chr10:63,991,638-64,994,157 | rs12248561 | 10:64451168_C_T | 3.11E-08 | 1.22 | 0.69 | 0.36 | 19kb 3' of ZNF365 | . | 0.02 | 7.861 |
| rs80161952 | chr10:63,991,638-64,994,157 | rs59043953 | 10:64451419_G_A | 2.52E-08 | 1.23 | 0.69 | 0.36 | 20kb 3' of ZNF365 | . | 0.02 | 14.02 |
| rs80161952 | chr10:63,991,638-64,994,157 | rs61592318 | 10:64459365_A_G | 3.99E-08 | 1.22 | 0.75 | 0.38 | 28kb 3' of ZNF365 | . | 0.01 | 5.664 |
| rs80161952 | chr10:63,991,638-64,994,157 | rs74624683 | 10:64461062_C_T | 1.61E-08 | 1.23 | 0.80 | 0.39 | 29kb 3' of ZNF365 | . | 0.02 | 2.17 |
| rs80161952 | chr10:63,991,638-64,994,157 | rs59841587 | 10:64463838_T_C | 2.52E-08 | 1.23 | 0.89 | 0.39 | . | . | 0.02 | 0.57 |
| rs80161952 | chr10:63,991,638-64,994,157 | rs57352524 | 10:64474915_C_T | 1.77E-08 | 1.23 | 0.88 | 0.39 | 43kb 3' of ZNF365 | . | 0.02 | 8.963 |
| rs80161952 | chr10:63,991,638-64,994,157 | rs60850962 | 10:64483634_C_G | 1.89E-08 | 1.23 | 0.88 | 0.39 | 52kb 3' of ZNF365 | . | 0.02 | 2.63 |
| rs80161952 | chr10:63,991,638-64,994,157 | rs79520290 | 10:64484853_G_C | 5.04E-08 | 1.22 | 0.92 | 0.40 | 53kb 3' of ZNF365 | . | 0.01 | 4.51 |
| rs80161952 | chr10:63,991,638-64,994,157 | rs79979619 | 10:64487844_G_A | 2.27E-08 | 1.23 | 0.87 | 0.39 | 56kb 3' of ZNF365 | . | 0.02 | 1.502 |
| rs80161952 | chr10:63,991,638-64,994,157 | rs77920050 | 10:64489257_C_T | 1.61E-08 | 1.23 | 0.86 | 0.39 | 57kb 3' of ZNF365 | . | 0.02 | 2.244 |
| rs80161952 | chr10:63,991,638-64,994,157 | rs73291184 | 10:64490674_T_C | 1.39E-08 | 1.23 | 0.85 | 0.39 | 59kb 3' of ZNF365 | . | 0.03 | 0.285 |
| rs80161952 | chr10:63,991,638-64,994,157 | rs77943290 | 10:64490766_A_T | 1.39E-08 | 1.23 | 0.85 | 0.39 | 59kb 3' of ZNF365 | . | 0.03 | 4.736 |
| rs80161952 | chr10:63,991,638-64,994,157 | rs76438762 | 10:64493592_T_C | 1.61E-08 | 1.23 | 0.86 | 0.39 | 62kb 3' of ZNF365 | . | 0.02 | 5.962 |
| rs80161952 | chr10:63,991,638-64,994,157 | rs80161952 | 10:64494302_T_C | 5.99E-09 | 1.34 | 0.95 | 1.00 | 63kb 3' of ZNF365 | . | 0.21 | 0.141 |
| rs80161952 | chr10:63,991,638-64,994,157 | rs76595948 | 10:64498722_A_C | 2.18E-08 | 1.23 | 0.87 | 0.39 | 66kb 5' of ADO | . | 0.02 | 4.46 |
| rs80161952 | chr10:63,991,638-64,994,157 | rs58507210 | 10:64499193_A_T | 7.40E-08 | 1.25 | 0.96 | 0.56 | 65kb 5' of ADO | . | 0.01 | 5.672 |
| rs80161952 | chr10:63,991,638-64,994,157 | rs58900042 | 10:64499518_G_A | 8.17E-08 | 1.24 | 0.96 | 0.56 | 65kb 5' of ADO | . | 0.01 | 6.736 |
| rs80161952 | chr10:63,991,638-64,994,157 | rs79561070 | 10:64499927_A_G | 2.80E-08 | 1.22 | 0.90 | 0.39 | 65kb 5' of ADO | . | 0.02 | 0.188 |
| rs80161952 | chr10:63,991,638-64,994,157 | rs78026788 | 10:64502243_A_G | 1.47E-08 | 1.23 | 0.87 | 0.39 | 62kb 5' of ADO | . | 0.03 | 4.063 |
| rs80161952 | chr10:63,991,638-64,994,157 | rs79606067 | 10:64502304_A_G | 1.47E-08 | 1.23 | 0.87 | 0.39 | 62kb 5' of ADO | . | 0.03 | 0.129 |
| rs80161952 | chr10:63,991,638-64,994,157 | rs78368330 | 10:64503041_C_T | 1.87E-08 | 1.22 | 0.94 | 0.36 | 61kb 5' of ADO | . | 0.02 | 4.627 |
| rs80161952 | chr10:63,991,638-64,994,157 | rs58600253 | 10:64507904_T_C | 9.06E-09 | 1.23 | 0.95 | 0.36 | 57kb 5' of ADO | . | 0.03 | 7.644 |
| rs80161952 | chr10:63,991,638-64,994,157 | rs57565226 | 10:64508010_T_C | 9.06E-09 | 1.23 | 0.95 | 0.36 | 57kb 5' of ADO | . | 0.03 | 3.353 |
| rs80161952 | chr10:63,991,638-64,994,157 | rs58418222 | 10:64509968_G_A | 1.38E-08 | 1.22 | 0.94 | 0.36 | 55kb 5' of ADO | . | 0.02 | 4.038 |
| rs80161952 | chr10:63,991,638-64,994,157 | rs113168114 | 10:64511676_C_T | 1.21E-08 | 1.22 | 0.95 | 0.36 | 53kb 5' of ADO | . | 0.03 | 5.529 |
| rs80161952 | chr10:63,991,638-64,994,157 | rs73292872 | 10:64512124_C_T | 1.21E-08 | 1.22 | 0.95 | 0.36 | 52kb 5' of ADO | . | 0.03 | 6.55 |
| rs80161952 | chr10:63,991,638-64,994,157 | rs79754022 | 10:64513538_T_C | 1.07E-08 | 1.22 | 0.96 | 0.36 | 51kb 5' of ADO | . | 0.03 | 4.353 |
| rs80161952 | chr10:63,991,638-64,994,157 | rs78783416 | 10:64518687_A_C | 2.71E-08 | 1.22 | 0.97 | 0.35 | 46kb 5' of ADO | . | 0.01 | 12.46 |
| rs80161952 | chr10:63,991,638-64,994,157 | rs117436764 | 10:64522738_G_A | 6.30E-08 | 1.21 | 0.95 | 0.35 | 42kb 5' of ADO | . | 0.01 | 9.255 |
| rs80161952 | chr10:63,991,638-64,994,157 | rs58018964 | 10:64524267_A_G | 7.15E-08 | 1.21 | 0.95 | 0.35 | 40kb 5' of ADO | . | 0.01 | 0.635 |
| rs80161952 | chr10:63,991,638-64,994,157 | rs76510618 | 10:64528725_T_C | 3.67E-08 | 1.21 | 0.97 | 0.33 | 36kb 5' of ADO | . | 0.04 | 15.35 |
| rs80161952 | chr10:63,991,638-64,994,157 | rs7074237 | 10:64545572_T_G | 3.39E-08 | 1.21 | 0.87 | 0.32 | 19kb 5' of ADO | . | 0.05 | 0.126 |
| rs80161952 | chr10:63,991,638-64,994,157 | rs11818600 | 10:64548854_A_G | 3.39E-08 | 1.21 | 0.87 | 0.32 | 16kb 5' of ADO | . | 0.05 | 4.824 |
| rs780666 | chr10:72,610,977-73,610,977 | rs2249989 | 10:73105421_G_C | 6.79E-08 | 1.14 | 0.76 | 0.99 | SLC29A3 | intronic | 0.06 | 6.191 |
| rs780666 | chr10:72,610,977-73,610,977 | rs2243485 | 10:73106690_G_A | 5.53E-08 | 1.14 | 0.77 | 0.99 | SLC29A3 | intronic | 0.07 | 1.652 |
| rs780666 | chr10:72,610,977-73,610,977 | rs1609651 | 10:73106800_A_G | 1.26E-07 | 1.14 | 0.81 | 0.98 | SLC29A3 | intronic | 0.03 | 3.433 |
| rs780666 | chr10:72,610,977-73,610,977 | rs2249752 | 10:73107786_G_A | 8.43E-08 | 1.14 | 0.70 | 0.99 | SLC29A3 | intronic | 0.05 | 7.554 |
| rs780666 | chr10:72,610,977-73,610,977 | rs780665 | 10:73110657_T_C | 1.10E-07 | 1.14 | 0.82 | 0.93 | SLC29A3 | intronic | 0.04 | 4.085 |
| rs780666 | chr10:72,610,977-73,610,977 | rs780666 | 10:73110977_C_A | 4.95E-08 | 1.15 | 0.71 | 1.00 | SLC29A3 | intronic | 0.08 | 7.372 |
| rs780666 | chr10:72,610,977-73,610,977 | rs780667 | 10:73111258_T_G | 7.57E-08 | 1.14 | 0.77 | 1.00 | SLC29A3 | intronic | 0.05 | 3.498 |
| rs780666 | chr10:72,610,977-73,610,977 | rs780668 | 10:73111408_T_C | 7.57E-08 | 1.14 | 0.77 | 1.00 | SLC29A3 | missense | 0.05 | 23.5 |
| rs780666 | chr10:72,610,977-73,610,977 | rs780670 | 10:73112257_G_C | 1.37E-07 | 1.14 | 0.84 | 0.98 | SLC29A3 | intronic | 0.03 | 3.88 |
| rs780666 | chr10:72,610,977-73,610,977 | rs780672 | 10:73113246_T_C | 1.46E-07 | 1.14 | 0.80 | 0.98 | SLC29A3 | intronic | 0.03 | 3.207 |
| rs780666 | chr10:72,610,977-73,610,977 | rs780674 | 10:73113497_A_G | 1.46E-07 | 1.14 | 0.80 | 0.98 | SLC29A3 | intronic | 0.03 | 8.412 |
| rs780666 | chr10:72,610,977-73,610,977 | rs780675 | 10:73113790_T_C | 1.58E-07 | 1.14 | 0.81 | 0.98 | SLC29A3 | intronic | 0.03 | 15.51 |
| rs780666 | chr10:72,610,977-73,610,977 | rs780676 | 10:73113931_T_C | 1.58E-07 | 1.14 | 0.81 | 0.98 | SLC29A3 | intronic | 0.03 | 0.716 |
| rs780666 | chr10:72,610,977-73,610,977 | rs780677 | 10:73114402_G_C | 1.68E-07 | 1.14 | 0.81 | 0.98 | SLC29A3 | intronic | 0.02 | 6.199 |
| rs780666 | chr10:72,610,977-73,610,977 | rs780678 | 10:73114486_T_C | 1.51E-07 | 1.14 | 0.80 | 0.98 | SLC29A3 | intronic | 0.03 | 0.192 |
| rs780666 | chr10:72,610,977-73,610,977 | rs780679 | 10:73114509_C_T | 8.12E-08 | 1.14 | 0.77 | 0.99 | SLC29A3 | intronic | 0.05 | 4.996 |
| rs780666 | chr10:72,610,977-73,610,977 | rs809691 | 10:73118099_T_A | 7.29E-08 | 1.14 | 0.81 | 0.92 | SLC29A3 | intronic | 0.05 | 4.244 |
| rs780666 | chr10:72,610,977-73,610,977 | rs780653 | 10:73118285_G_C | 7.74E-08 | 1.14 | 0.82 | 0.92 | SLC29A3 | intronic | 0.05 | 0.772 |
| rs780666 | chr10:72,610,977-73,610,977 | rs703256 | 10:73119023_T_C | 7.29E-08 | 1.14 | 0.81 | 0.92 | SLC29A3 | intronic | 0.05 | 0.51 |
| rs780666 | chr10:72,610,977-73,610,977 | rs780654 | 10:73119233_A_G | 1.20E-07 | 1.14 | 0.81 | 0.98 | SLC29A3 | intronic | 0.03 | 0.385 |
| rs780666 | chr10:72,610,977-73,610,977 | rs2487066 | 10:73121591_C_A | 5.89E-08 | 1.14 | 0.78 | 0.96 | SLC29A3 | intronic | 0.06 | 1.303 |
| rs780666 | chr10:72,610,977-73,610,977 | rs2487067 | 10:73121645_A_C | 1.14E-07 | 1.14 | 0.73 | 0.96 | SLC29A3 | intronic | 0.03 | 0.213 |
| rs780666 | chr10:72,610,977-73,610,977 | rs780690 | 10:73127384_C_A | 2.31E-07 | 1.14 | 0.74 | 0.73 | 4.2kb 3' of SLC29A3 | . | 0.02 | 13.4 |
| rs3764147 | chr13:43,959,499-45,012,908 | rs3764147 | 13:44457925_G_A | 4.12E-81 | 1.63 | 0.79 | 1.00 | LACC1 | missense | 0.49 | 12.52 |
| rs3764147 | chr13:43,959,499-45,012,908 | rs1932990 | 13:44460242_T_C | 6.33E-81 | 1.63 | 0.78 | 1.00 | LACC1 | intronic | 0.33 | 5.69 |
| rs3764147 | chr13:43,959,499-45,012,908 | rs2121034 | 13:44473866_T_G | 2.93E-80 | 1.63 | 0.73 | 0.99 | 5.8kb 3' of LACC1 | . | 0.06 | 5.56 |
| rs3764147 | chr13:43,959,499-45,012,908 | rs2121033 | 13:44475052_G_C | 3.93E-80 | 1.63 | 0.74 | 0.99 | 7kb 3' of LACC1 | . | 0.05 | 0.744 |
| rs3764147 | chr13:43,959,499-45,012,908 | rs1373904 | 13:44475398_G_A | 3.20E-80 | 1.63 | 0.75 | 0.99 | 7.3kb 3' of LACC1 | . | 0.06 | 2.241 |
| rs190879280 | chr13:43,959,499-45,012,908 | rs117869348 | 13:44495076_C_A | 2.37E-25 | 0.61 | 0.93 | 0.98 | 27kb 3' of LACC1 | . | 0.16 | 3.49 |
| rs190879280 | chr13:43,959,499-45,012,908 | rs190879280 | 13:44502243_G_C | 1.90E-25 | 0.61 | 0.90 | 1.00 | 34kb 3' of LACC1 | . | 0.30 | 2.148 |
| rs190879280 | chr13:43,959,499-45,012,908 | rs80216552 | 13:44505692_G_A | 5.20E-25 | 0.61 | 0.89 | 0.98 | 38kb 3' of LACC1 | . | 0.23 | 1.328 |
| rs190879280 | chr13:43,959,499-45,012,908 | rs77972794 | 13:44505716_G_C | 3.67E-25 | 0.61 | 0.86 | 0.98 | 38kb 3' of LACC1 | . | 0.29 | 0.765 |
| rs4781072 | chr16:10,960,480-11,960,480 | rs11646972 | 16:11459939_C_T | 5.21E-12 | 0.84 | 0.57 | 0.85 | 14kb 3' of RMI2 | intronic | 0.26 | 2.883 |
| rs4781072 | chr16:10,960,480-11,960,480 | rs4781072 | 16:11460480_C_T | 1.87E-12 | 0.84 | 0.58 | 1.00 | 15kb 3' of RMI2 | intronic | 0.69 | 2.468 |
| rs180744 | chr16:28,008,048-29,008,048 | rs149271 | 16:28506872_G_A | 9.90E-11 | 0.78 | 0.35 | 0.99 | APOBR | synonymous | 0.08 | 10.23 |
| rs180744 | chr16:28,008,048-29,008,048 | rs180743 | 16:28507644_G_C | 8.24E-11 | 0.78 | 0.35 | 1.00 | APOBR | missense | 0.10 | 9.161 |
| rs180744 | chr16:28,008,048-29,008,048 | rs180744 | 16:28508048_G_A | 7.40E-11 | 0.78 | 0.35 | 1.00 | APOBR | synonymous | 0.11 | 0.632 |
| rs180744 | chr16:28,008,048-29,008,048 | rs151174 | 16:28508069_T_C | 3.62E-10 | 0.78 | 0.29 | 0.98 | APOBR | synonymous | 0.02 | 0.056 |
| rs180744 | chr16:28,008,048-29,008,048 | rs181203 | 16:28512371_C_A | 4.10E-10 | 0.78 | 0.58 | 0.94 | IL27 | intronic | 0.02 | 1.997 |
| rs180744 | chr16:28,008,048-29,008,048 | rs181205 | 16:28513129_C_T | 2.82E-10 | 0.78 | 0.60 | 0.94 | . | . | 0.03 | 4.035 |
| rs180744 | chr16:28,008,048-29,008,048 | rs181206 | 16:28513403_G_A | 3.57E-10 | 0.78 | 0.59 | 0.94 | IL27 | missense | 0.02 | 22.4 |
| rs180744 | chr16:28,008,048-29,008,048 | rs181207 | 16:28513530_T_C | 1.53E-10 | 0.78 | 0.51 | 0.93 | IL27 | intronic | 0.05 | 1.11 |
| rs180744 | chr16:28,008,048-29,008,048 | rs181209 | 16:28514854_T_G | 3.21E-10 | 0.78 | 0.62 | 0.93 | IL27 | intronic | 0.03 | 0.57 |
| rs180744 | chr16:28,008,048-29,008,048 | rs56354901 | 16:28523144_C_T | 3.80E-10 | 0.78 | 0.60 | 0.93 | 5kb 5' of IL27 | . | 0.02 | 0.927 |
| rs180744 | chr16:28,008,048-29,008,048 | rs3785354 | 16:28550667_T_C | 1.68E-09 | 0.78 | 0.84 | 0.77 | 171bp 5' of NUPR1 | . | 0.01 | 0.331 |
| rs180744 | chr16:28,008,048-29,008,048 | rs1074631 | 16:28554108_A_G | 1.43E-09 | 0.78 | 0.85 | 0.77 | 3.6kb 5' of NUPR1 | . | 0.01 | 4.606 |
| rs180744 | chr16:28,008,048-29,008,048 | rs4787457 | 16:28555400_G_A | 1.43E-09 | 0.78 | 0.85 | 0.77 | 4.9kb 5' of NUPR1 | . | 0.01 | 3.131 |
| rs180744 | chr16:28,008,048-29,008,048 | rs4788081 | 16:28556147_C_T | 1.43E-09 | 0.78 | 0.85 | 0.77 | 5.7kb 5' of NUPR1 | . | 0.01 | 1.898 |
| rs180744 | chr16:28,008,048-29,008,048 | rs4788080 | 16:28558081_T_C | 1.60E-09 | 0.78 | 0.86 | 0.77 | 7.2kb 5' of CCDC101 | . | 0.01 | 2.736 |
| rs180744 | chr16:28,008,048-29,008,048 | rs4787456 | 16:28559573_T_C | 1.60E-09 | 0.78 | 0.86 | 0.77 | 5.7kb 5' of CCDC101 | . | 0.01 | 0.901 |
| rs180744 | chr16:28,008,048-29,008,048 | rs4788078 | 16:28559981_A_G | 1.74E-09 | 0.78 | 0.87 | 0.77 | 5.3kb 5' of CCDC101 | . | 0.01 | 0.948 |
| rs180744 | chr16:28,008,048-29,008,048 | rs112366001 | 16:28561785_A_T | 1.55E-09 | 0.78 | 0.87 | 0.77 | 3.5kb 5' of CCDC101 | . | 0.01 | 1.227 |
| rs180744 | chr16:28,008,048-29,008,048 | rs62034350 | 16:28562256_G_A | 1.74E-09 | 0.78 | 0.87 | 0.77 | 3kb 5' of CCDC101 | . | 0.01 | 7.739 |
| rs180744 | chr16:28,008,048-29,008,048 | rs62034351 | 16:28565489_A_G | 1.00E-09 | 0.78 | 0.91 | 0.76 | CCDC101 | intronic | 0.01 | 9.461 |
| rs180744 | chr16:28,008,048-29,008,048 | rs7187604 | 16:28565953_G_A | 1.50E-09 | 0.78 | 0.87 | 0.77 | CCDC101 | intronic | 0.01 | 6.668 |
| rs180744 | chr16:28,008,048-29,008,048 | rs4788077 | 16:28569764_C_G | 1.69E-09 | 0.78 | 0.87 | 0.77 | CCDC101 | intronic | 0.01 | 1.521 |
| rs180744 | chr16:28,008,048-29,008,048 | rs4788076 | 16:28570005_T_C | 1.50E-09 | 0.78 | 0.87 | 0.77 | CCDC101 | intronic | 0.01 | 0.231 |
| rs180744 | chr16:28,008,048-29,008,048 | rs56272201 | 16:28580685_G_A | 1.76E-09 | 0.78 | 0.86 | 0.77 | CCDC101 | intronic | 0.01 | 7.628 |
| rs180744 | chr16:28,008,048-29,008,048 | rs56396270 | 16:28580829_T_C | 1.76E-09 | 0.78 | 0.86 | 0.77 | CCDC101 | intronic | 0.01 | 0.216 |
| rs180744 | chr16:28,008,048-29,008,048 | rs62034355 | 16:28581038_C_G | 1.76E-09 | 0.78 | 0.86 | 0.77 | CCDC101 | intronic | 0.01 | 6.593 |
| rs180744 | chr16:28,008,048-29,008,048 | rs12447461 | 16:28582941_A_C | 5.81E-10 | 0.78 | 0.70 | 0.77 | CCDC101 | intronic | 0.01 | 11.23 |
| rs180744 | chr16:28,008,048-29,008,048 | rs112191041 | 16:28583362_G_A | 5.81E-10 | 0.78 | 0.70 | 0.77 | CCDC101 | intronic | 0.01 | 3.079 |
| rs180744 | chr16:28,008,048-29,008,048 | rs111693583 | 16:28583610_T_C | 5.81E-10 | 0.78 | 0.70 | 0.77 | CCDC101 | intronic | 0.01 | 3.668 |
| rs180744 | chr16:28,008,048-29,008,048 | rs11074911 | 16:28583865_T_C | 5.14E-10 | 0.78 | 0.70 | 0.77 | CCDC101 | intronic | 0.02 | 1.723 |
| rs180744 | chr16:28,008,048-29,008,048 | rs56209193 | 16:28585636_T_C | 6.46E-10 | 0.78 | 0.70 | 0.77 | CCDC101 | intronic | 0.01 | 0.572 |
| rs180744 | chr16:28,008,048-29,008,048 | rs7186573 | 16:28587389_C_T | 5.32E-10 | 0.78 | 0.70 | 0.77 | CCDC101 | intronic | 0.02 | 3.373 |
| rs180744 | chr16:28,008,048-29,008,048 | rs62034358 | 16:28587597_T_G | 1.74E-09 | 0.78 | 0.85 | 0.77 | CCDC101 | intronic | 0.01 | 0.058 |
| rs180744 | chr16:28,008,048-29,008,048 | rs12445823 | 16:28588049_C_A | 1.74E-09 | 0.78 | 0.85 | 0.77 | CCDC101 | intronic | 0.01 | 4.345 |
| rs180744 | chr16:28,008,048-29,008,048 | rs12445744 | 16:28588395_C_A | 1.74E-09 | 0.78 | 0.85 | 0.77 | CCDC101 | intronic | 0.01 | 3.331 |
| rs180744 | chr16:28,008,048-29,008,048 | rs12448429 | 16:28588443_T_C | 5.32E-10 | 0.78 | 0.70 | 0.77 | CCDC101 | intronic | 0.02 | 1.928 |
| rs180744 | chr16:28,008,048-29,008,048 | rs4787455 | 16:28589455_A_G | 1.74E-09 | 0.78 | 0.85 | 0.77 | CCDC101 | intronic | 0.01 | 7.045 |
| rs180744 | chr16:28,008,048-29,008,048 | rs7190771 | 16:28590030_A_G | 5.32E-10 | 0.78 | 0.70 | 0.77 | CCDC101 | intronic | 0.02 | 1.389 |
| rs180744 | chr16:28,008,048-29,008,048 | rs112047757 | 16:28590862_T_C | 5.32E-10 | 0.78 | 0.70 | 0.77 | CCDC101 | intronic | 0.02 | 4.975 |
| rs180744 | chr16:28,008,048-29,008,048 | rs112831778 | 16:28590989_T_C | 1.74E-09 | 0.78 | 0.85 | 0.77 | CCDC101 | intronic | 0.01 | 1.945 |
| rs180744 | chr16:28,008,048-29,008,048 | rs17707300 | 16:28593347_C_T | 5.32E-10 | 0.78 | 0.70 | 0.77 | CCDC101 | intronic | 0.02 | 1.497 |
| rs180744 | chr16:28,008,048-29,008,048 | rs4788074 | 16:28593597_A_G | 1.74E-09 | 0.78 | 0.85 | 0.77 | CCDC101 | intronic | 0.01 | 2.243 |
| rs180744 | chr16:28,008,048-29,008,048 | rs4788073 | 16:28594549_G_A | 4.47E-10 | 0.78 | 0.84 | 0.77 | CCDC101 | intronic | 0.02 | 7.056 |
| rs180744 | chr16:28,008,048-29,008,048 | rs17640009 | 16:28595700_G_A | 4.77E-10 | 0.78 | 0.72 | 0.77 | CCDC101 | intronic | 0.02 | 3.549 |
| rs180744 | chr16:28,008,048-29,008,048 | rs75539558 | 16:28595810_G_C | 4.77E-10 | 0.78 | 0.72 | 0.77 | CCDC101 | intronic | 0.02 | 2.317 |
| rs180744 | chr16:28,008,048-29,008,048 | rs55792032 | 16:28599411_G_A | 4.77E-10 | 0.78 | 0.72 | 0.77 | CCDC101 | intronic | 0.02 | 0.734 |
| rs180744 | chr16:28,008,048-29,008,048 | rs62034359 | 16:28600091_T_C | 1.56E-09 | 0.78 | 0.86 | 0.77 | CCDC101 | intronic | 0.01 | 0.237 |
| rs180744 | chr16:28,008,048-29,008,048 | rs2077031 | 16:28603168_A_T | 1.56E-09 | 0.78 | 0.86 | 0.77 | 56bp 3' of CCDC101 | . | 0.01 | 0.842 |
| rs180744 | chr16:28,008,048-29,008,048 | rs762634 | 16:28603335_C_T | 1.56E-09 | 0.78 | 0.86 | 0.77 | SULT1A2 | 3'-UTR | 0.01 | 4.506 |
| rs180744 | chr16:28,008,048-29,008,048 | rs710410 | 16:28603342_G_A | 1.56E-09 | 0.78 | 0.86 | 0.77 | SULT1A2 | 3'-UTR | 0.01 | 0.202 |
| rs180744 | chr16:28,008,048-29,008,048 | rs1059491 | 16:28603655_G_T | 1.56E-09 | 0.78 | 0.86 | 0.77 | SULT1A2 | missense | 0.01 | 24.7 |
| rs180744 | chr16:28,008,048-29,008,048 | rs62031560 | 16:28605915_A_T | 4.77E-10 | 0.78 | 0.72 | 0.77 | SULT1A2 | intronic | 0.02 | 2.036 |
| rs180744 | chr16:28,008,048-29,008,048 | rs113208333 | 16:28606160_T_G | 8.11E-10 | 0.78 | 0.70 | 0.77 | SULT1A2 | intronic | 0.01 | 1.262 |
| rs180744 | chr16:28,008,048-29,008,048 | rs4149406 | 16:28607070_G_A | 4.66E-10 | 0.78 | 0.72 | 0.77 | SULT1A2 | intronic | 0.02 | 7.408 |
| rs180744 | chr16:28,008,048-29,008,048 | rs4115668 | 16:28607532_A_G | 1.66E-09 | 0.78 | 0.86 | 0.76 | SULT1A2 | 5'-UTR | 0.01 | 5.822 |
| rs180744 | chr16:28,008,048-29,008,048 | rs743590 | 16:28608230_A_G | 4.19E-10 | 0.78 | 0.72 | 0.77 | SULT1A2 | intronic | 0.02 | 5.357 |
| rs180744 | chr16:28,008,048-29,008,048 | rs762633 | 16:28608341_C_T | 1.57E-09 | 0.78 | 0.86 | 0.76 | SULT1A2 | 5'-UTR | 0.01 | 5.274 |
| rs180744 | chr16:28,008,048-29,008,048 | rs4149398 | 16:28608938_G_C | 1.23E-09 | 0.78 | 0.90 | 0.76 | 546bp 5' of SULT1A2 | . | 0.01 | 0.795 |
| rs180744 | chr16:28,008,048-29,008,048 | rs62031562 | 16:28609329_T_A | 1.27E-09 | 0.78 | 0.83 | 0.77 | 937bp 5' of SULT1A2 | . | 0.01 | 9.481 |
| rs8063362 | chr16:50,223,371-51,223,371 | rs2270368 | 16:50714335_A_G | 2.30E-63 | 1.56 | 0.07 | 0.98 | SNX20 | intronic | 0.13 | 0.338 |
| rs8063362 | chr16:50,223,371-51,223,371 | rs2287195 | 16:50714979_C_T | 2.03E-63 | 1.55 | 0.07 | 0.98 | SNX20 | intronic | 0.14 | 0.533 |
| rs8063362 | chr16:50,223,371-51,223,371 | rs9939349 | 16:50716398_G_T | 2.05E-63 | 1.55 | 0.04 | 1.00 | 1.1kb 5' of SNX20 | . | 0.14 | 0.094 |
| rs8063362 | chr16:50,223,371-51,223,371 | rs9302752 | 16:50719103_C_T | 2.52E-63 | 1.55 | 0.03 | 0.99 | 3.8kb 5' of SNX20 | . | 0.12 | 2.284 |
| rs8063362 | chr16:50,223,371-51,223,371 | rs1981760 | 16:50723074_T_C | 4.11E-63 | 1.55 | 0.03 | 1.00 | 7.8kb 5' of SNX20 | . | 0.07 | 0.633 |
| rs8063362 | chr16:50,223,371-51,223,371 | rs8063362 | 16:50723371_T_A | 1.47E-63 | 1.56 | 0.03 | 1.00 | 7.7kb 5' of NOD2 | . | 0.20 | 10.53 |
| rs8063362 | chr16:50,223,371-51,223,371 | rs9933594 | 16:50724087_C_G | 3.31E-63 | 1.55 | 0.04 | 0.99 | 7kb 5' of NOD2 | . | 0.09 | 0.053 |
| rs8063362 | chr16:50,223,371-51,223,371 | rs12926429 | 16:50724614_A_G | 3.32E-63 | 1.55 | 0.04 | 0.99 | 6.4kb 5' of NOD2 | . | 0.09 | 8.233 |

GWAS_lead, lead SNP of each independent signal;

CHR:BP_A1_A2, CHR, Chromosome; BP, position based on hg19 coordinates; A1, minor allele; A2, major allele;

Phet, P value of heterogeneity; r2, r2 with the lead SNP in all GWAS samples;

Function, SNP funcition annotation from HaploReg v4.1 (<https://pubs.broadinstitute.org/mammals/haploreg/haploreg.php>);

PIP, the posterior inclusion probability (PIP) that each SNP was causal by SusieR R package;

CADD, the score value from CADD tool (CADD score >15 was defined as deleterious;

Table S5. The eQTL results of rs2291617 and rs10747783.

A) rs2291617

| **Study ID** | **PMID** | **Tissue** | **Correlated gene** | **p-value** |
| --- | --- | --- | --- | --- |
| GTEx2015_v6 | [25954001](http://pubmed.gov/25954001) | Adipose_Subcutaneous | METTL21B | 7.79E-30 |
| GTEx2015_v6 | [25954001](http://pubmed.gov/25954001) | Adipose_Subcutaneous | XRCC6BP1 | 1.31E-10 |
| GTEx2015_v6 | [25954001](http://pubmed.gov/25954001) | Adipose_Visceral_Omentum | METTL21B | 5.98E-17 |
| GTEx2015_v6 | [25954001](http://pubmed.gov/25954001) | Adipose_Visceral_Omentum | TSFM | 3.05E-06 |
| GTEx2015_v6 | [25954001](http://pubmed.gov/25954001) | Adrenal_Gland | METTL21B | 3.37E-13 |
| GTEx2015_v6 | [25954001](http://pubmed.gov/25954001) | Adrenal_Gland | TSFM | 6.06E-11 |
| GTEx2015_v6 | [25954001](http://pubmed.gov/25954001) | Artery_Aorta | METTL21B | 2.54E-18 |
| GTEx2015_v6 | [25954001](http://pubmed.gov/25954001) | Artery_Aorta | TSFM | 2.20E-06 |
| GTEx2015_v6 | [25954001](http://pubmed.gov/25954001) | Artery_Aorta | XRCC6BP1 | 1.13E-07 |
| GTEx2015_v6 | [25954001](http://pubmed.gov/25954001) | Artery_Coronary | METTL21B | 8.14E-07 |
| GTEx2015_v6 | [25954001](http://pubmed.gov/25954001) | Artery_Tibial | METTL21B | 3.30E-19 |
| GTEx2015_v6 | [25954001](http://pubmed.gov/25954001) | Artery_Tibial | TSFM | 7.37E-08 |
| GTEx2015_v6 | [25954001](http://pubmed.gov/25954001) | Artery_Tibial | XRCC6BP1 | 4.06E-11 |
| GTEx2015_v6 | [25954001](http://pubmed.gov/25954001) | Brain_Anterior_cingulate_cortex_BA24 | METTL21B | 3.01E-06 |
| GTEx2015_v6 | [25954001](http://pubmed.gov/25954001) | Brain_Caudate_basal_ganglia | METTL21B | 1.80E-14 |
| GTEx2015_v6 | [25954001](http://pubmed.gov/25954001) | Brain_Cerebellar_Hemisphere | METTL21B | 1.71E-19 |
| GTEx2015_v6 | [25954001](http://pubmed.gov/25954001) | Brain_Cerebellum | METTL21B | 3.01E-19 |
| GTEx2015_v6 | [25954001](http://pubmed.gov/25954001) | Brain_Cortex | METTL21B | 1.98E-14 |
| GTEx2015_v6 | [25954001](http://pubmed.gov/25954001) | Brain_Frontal_Cortex_BA9 | METTL21B | 1.17E-09 |
| GTEx2015_v6 | [25954001](http://pubmed.gov/25954001) | Brain_Hippocampus | METTL21B | 2.63E-08 |
| GTEx2015_v6 | [25954001](http://pubmed.gov/25954001) | Brain_Hypothalamus | METTL21B | 1.92E-09 |
| GTEx2015_v6 | [25954001](http://pubmed.gov/25954001) | Brain_Nucleus_accumbens_basal_ganglia | METTL21B | 1.98E-15 |
| GTEx2015_v6 | [25954001](http://pubmed.gov/25954001) | Brain_Putamen_basal_ganglia | METTL21B | 8.54E-13 |
| GTEx2015_v6 | [25954001](http://pubmed.gov/25954001) | Brain_Putamen_basal_ganglia | RP11-571M6.17 | 7.03E-07 |
| GTEx2015_v6 | [25954001](http://pubmed.gov/25954001) | Breast_Mammary_Tissue | METTL21B | 5.20E-08 |
| GTEx2015_v6 | [25954001](http://pubmed.gov/25954001) | Cells_EBV-transformed_lymphocytes | METTL21B | 1.20E-11 |
| GTEx2015_v6 | [25954001](http://pubmed.gov/25954001) | Cells_Transformed_fibroblasts | METTL21B | 2.19E-47 |
| GTEx2015_v6 | [25954001](http://pubmed.gov/25954001) | Cells_Transformed_fibroblasts | TSFM | 8.48E-17 |
| GTEx2015_v6 | [25954001](http://pubmed.gov/25954001) | Cells_Transformed_fibroblasts | XRCC6BP1 | 1.17E-06 |
| GTEx2015_v6 | [25954001](http://pubmed.gov/25954001) | Colon_Sigmoid | METTL21B | 1.85E-11 |
| GTEx2015_v6 | [25954001](http://pubmed.gov/25954001) | Colon_Sigmoid | XRCC6BP1 | 7.20E-07 |
| GTEx2015_v6 | [25954001](http://pubmed.gov/25954001) | Colon_Transverse | METTL21B | 1.95E-15 |
| GTEx2015_v6 | [25954001](http://pubmed.gov/25954001) | Esophagus_Gastroesophageal_Junction | METTL21B | 9.67E-13 |
| GTEx2015_v6 | [25954001](http://pubmed.gov/25954001) | Esophagus_Mucosa | METTL21B | 8.14E-18 |
| GTEx2015_v6 | [25954001](http://pubmed.gov/25954001) | Esophagus_Mucosa | XRCC6BP1 | 1.18E-05 |
| GTEx2015_v6 | [25954001](http://pubmed.gov/25954001) | Esophagus_Muscularis | METTL21B | 1.16E-22 |
| GTEx2015_v6 | [25954001](http://pubmed.gov/25954001) | Esophagus_Muscularis | RP11-571M6.7 | 1.49E-05 |
| GTEx2015_v6 | [25954001](http://pubmed.gov/25954001) | Esophagus_Muscularis | TSFM | 2.97E-09 |
| GTEx2015_v6 | [25954001](http://pubmed.gov/25954001) | Esophagus_Muscularis | XRCC6BP1 | 3.51E-07 |
| GTEx2015_v6 | [25954001](http://pubmed.gov/25954001) | Heart_Atrial_Appendage | METTL21B | 1.09E-09 |
| GTEx2015_v6 | [25954001](http://pubmed.gov/25954001) | Heart_Atrial_Appendage | TSFM | 2.78E-08 |
| GTEx2015_v6 | [25954001](http://pubmed.gov/25954001) | Heart_Atrial_Appendage | XRCC6BP1 | 6.63E-08 |
| GTEx2015_v6 | [25954001](http://pubmed.gov/25954001) | Heart_Left_Ventricle | METTL21B | 8.10E-06 |
| GTEx2015_v6 | [25954001](http://pubmed.gov/25954001) | Heart_Left_Ventricle | SLC26A10 | 1.50E-07 |
| GTEx2015_v6 | [25954001](http://pubmed.gov/25954001) | Heart_Left_Ventricle | TSFM | 2.97E-07 |
| GTEx2015_v6 | [25954001](http://pubmed.gov/25954001) | Heart_Left_Ventricle | XRCC6BP1 | 2.48E-07 |
| GTEx2015_v6 | [25954001](http://pubmed.gov/25954001) | Lung | METTL21B | 4.68E-20 |
| GTEx2015_v6 | [25954001](http://pubmed.gov/25954001) | Lung | XRCC6BP1 | 1.33E-07 |
| GTEx2015_v6 | [25954001](http://pubmed.gov/25954001) | Muscle_Skeletal | METTL21B | 4.25E-14 |
| GTEx2015_v6 | [25954001](http://pubmed.gov/25954001) | Muscle_Skeletal | RP11-571M6.17 | 3.28E-06 |
| GTEx2015_v6 | [25954001](http://pubmed.gov/25954001) | Muscle_Skeletal | TSFM | 2.05E-13 |
| GTEx2015_v6 | [25954001](http://pubmed.gov/25954001) | Muscle_Skeletal | XRCC6BP1 | 1.01E-12 |
| GTEx2015_v6 | [25954001](http://pubmed.gov/25954001) | Nerve_Tibial | METTL21B | 9.99E-23 |
| GTEx2015_v6 | [25954001](http://pubmed.gov/25954001) | Nerve_Tibial | XRCC6BP1 | 1.39E-11 |
| GTEx2015_v6 | [25954001](http://pubmed.gov/25954001) | Pancreas | METTL21B | 1.91E-13 |
| GTEx2015_v6 | [25954001](http://pubmed.gov/25954001) | Pituitary | METTL21B | 4.48E-06 |
| GTEx2015_v6 | [25954001](http://pubmed.gov/25954001) | Skin_Not_Sun_Exposed_Suprapubic | METTL21B | 3.26E-09 |
| GTEx2015_v6 | [25954001](http://pubmed.gov/25954001) | Skin_Sun_Exposed_Lower_leg | METTL21B | 7.21E-24 |
| GTEx2015_v6 | [25954001](http://pubmed.gov/25954001) | Skin_Sun_Exposed_Lower_leg | XRCC6BP1 | 7.78E-08 |
| GTEx2015_v6 | [25954001](http://pubmed.gov/25954001) | Spleen | METTL21B | 2.11E-08 |
| GTEx2015_v6 | [25954001](http://pubmed.gov/25954001) | Stomach | METTL21B | 7.14E-14 |
| GTEx2015_v6 | [25954001](http://pubmed.gov/25954001) | Thyroid | CYP27B1 | 8.05E-38 |
| GTEx2015_v6 | [25954001](http://pubmed.gov/25954001) | Thyroid | METTL1 | 8.33E-15 |
| GTEx2015_v6 | [25954001](http://pubmed.gov/25954001) | Thyroid | TSFM | 8.99E-07 |
| GTEx2015_v6 | [25954001](http://pubmed.gov/25954001) | Thyroid | XRCC6BP1 | 8.01E-10 |
| GTEx2015_v6 | [25954001](http://pubmed.gov/25954001) | Vagina | METTL21B | 1.81E-06 |
| GTEx2015_v6 | [25954001](http://pubmed.gov/25954001) | Whole_Blood | METTL21B | 2.57E-25 |
| GTEx2015_v6 | [25954001](http://pubmed.gov/25954001) | Whole_Blood | XRCC6BP1 | 2.88E-11 |
| Gibbs2010 | [20485568](http://pubmed.gov/20485568) | Cerbellum | FAM119B | 1.84E-11 |
| Gibbs2010 | [20485568](http://pubmed.gov/20485568) | FrontalCortex | FAM119B | 1.72E-12 |
| Gibbs2010 | [20485568](http://pubmed.gov/20485568) | Pons | FAM119B | 2.50E-08 |
| Lappalainen2013 | [24037378](http://pubmed.gov/24037378) | Lymphoblastoid_EUR_exonlevel | ENSG00000123297.11_58176372_58176690 | 8.52E-14 |
| Lappalainen2013 | [24037378](http://pubmed.gov/24037378) | Lymphoblastoid_EUR_exonlevel | ENSG00000123297.11_58176893_58177066 | 1.80E-24 |
| Lappalainen2013 | [24037378](http://pubmed.gov/24037378) | Lymphoblastoid_EUR_exonlevel | ENSG00000123297.11_58179946_58180074 | 2.60E-16 |
| Lappalainen2013 | [24037378](http://pubmed.gov/24037378) | Lymphoblastoid_EUR_exonlevel | ENSG00000123297.11_58180823_58180945 | 7.40E-21 |
| Lappalainen2013 | [24037378](http://pubmed.gov/24037378) | Lymphoblastoid_EUR_exonlevel | ENSG00000123297.11_58186769_58186856 | 3.45E-19 |
| Lappalainen2013 | [24037378](http://pubmed.gov/24037378) | Lymphoblastoid_EUR_exonlevel | ENSG00000123297.11_58189960_58191370 | 8.38E-30 |
| Lappalainen2013 | [24037378](http://pubmed.gov/24037378) | Lymphoblastoid_EUR_exonlevel | ENSG00000123427.11_58174038_58176324 | 1.05E-61 |
| Lappalainen2013 | [24037378](http://pubmed.gov/24037378) | Lymphoblastoid_EUR_exonlevel | ENSG00000135452.5_58139959_58140039 | 1.24E-05 |
| Lappalainen2013 | [24037378](http://pubmed.gov/24037378) | Lymphoblastoid_EUR_exonlevel | ENSG00000135452.5_58140372_58140503 | 3.36E-06 |
| Lappalainen2013 | [24037378](http://pubmed.gov/24037378) | Lymphoblastoid_EUR_exonlevel | ENSG00000135452.5_58140801_58140914 | 6.78E-08 |
| Lappalainen2013 | [24037378](http://pubmed.gov/24037378) | Lymphoblastoid_EUR_exonlevel | ENSG00000135506.11_58111929_58112204 | 9.47E-06 |
| Lappalainen2013 | [24037378](http://pubmed.gov/24037378) | Lymphoblastoid_EUR_exonlevel | ENSG00000166896.3_58340778_58340859 | 1.06E-05 |
| Lappalainen2013 | [24037378](http://pubmed.gov/24037378) | Lymphoblastoid_EUR_exonlevel | ENSG00000166896.3_58345541_58345678 | 3.64E-07 |
| Lappalainen2013 | [24037378](http://pubmed.gov/24037378) | Lymphoblastoid_EUR_exonlevel | ENSG00000257921.1_58176893_58177066 | 1.10E-20 |
| Lappalainen2013 | [24037378](http://pubmed.gov/24037378) | Lymphoblastoid_EUR_exonlevel | ENSG00000257921.1_58179946_58180030 | 1.89E-09 |
| Lappalainen2013 | [24037378](http://pubmed.gov/24037378) | Lymphoblastoid_EUR_genelevel | TSFM | 3.89E-08 |
| Stranger2008 | [17873874](http://pubmed.gov/17873874) | Lymphoblastoid | FAM119B | 1.05E-21 |
| Westra2013 | [24013639](http://pubmed.gov/24013639) | Whole_Blood | FAM119B | 9.81E-198 |
| Westra2013 | [24013639](http://pubmed.gov/24013639) | Whole_Blood | TSFM | 8.84E-22 |
| Westra2013 | [24013639](http://pubmed.gov/24013639) | Whole_Blood | TSPAN31 | 1.39E-38 |

B) rs10747783

| **Study ID** | **PMID** | **Tissue** | **Correlated gene** | **p-value** |
| --- | --- | --- | --- | --- |
| GTEx2015_v6 | [25954001](http://pubmed.gov/25954001) | Adipose_Subcutaneous | METTL21B | 1.49E-28 |
| GTEx2015_v6 | [25954001](http://pubmed.gov/25954001) | Adipose_Subcutaneous | XRCC6BP1 | 8.15E-12 |
| GTEx2015_v6 | [25954001](http://pubmed.gov/25954001) | Adipose_Visceral_Omentum | METTL21B | 4.83E-18 |
| GTEx2015_v6 | [25954001](http://pubmed.gov/25954001) | Adipose_Visceral_Omentum | TSFM | 8.37E-06 |
| GTEx2015_v6 | [25954001](http://pubmed.gov/25954001) | Adrenal_Gland | METTL21B | 5.52E-15 |
| GTEx2015_v6 | [25954001](http://pubmed.gov/25954001) | Adrenal_Gland | TSFM | 1.92E-10 |
| GTEx2015_v6 | [25954001](http://pubmed.gov/25954001) | Artery_Aorta | METTL21B | 4.52E-17 |
| GTEx2015_v6 | [25954001](http://pubmed.gov/25954001) | Artery_Aorta | TSFM | 7.50E-07 |
| GTEx2015_v6 | [25954001](http://pubmed.gov/25954001) | Artery_Aorta | XRCC6BP1 | 1.16E-07 |
| GTEx2015_v6 | [25954001](http://pubmed.gov/25954001) | Artery_Coronary | METTL21B | 4.41E-07 |
| GTEx2015_v6 | [25954001](http://pubmed.gov/25954001) | Artery_Tibial | METTL21B | 1.08E-19 |
| GTEx2015_v6 | [25954001](http://pubmed.gov/25954001) | Artery_Tibial | RP11-571M6.7 | 2.16E-05 |
| GTEx2015_v6 | [25954001](http://pubmed.gov/25954001) | Artery_Tibial | TSFM | 6.15E-08 |
| GTEx2015_v6 | [25954001](http://pubmed.gov/25954001) | Artery_Tibial | XRCC6BP1 | 2.20E-10 |
| GTEx2015_v6 | [25954001](http://pubmed.gov/25954001) | Brain_Anterior_cingulate_cortex_BA24 | METTL21B | 3.95E-07 |
| GTEx2015_v6 | [25954001](http://pubmed.gov/25954001) | Brain_Caudate_basal_ganglia | METTL21B | 2.66E-14 |
| GTEx2015_v6 | [25954001](http://pubmed.gov/25954001) | Brain_Cerebellar_Hemisphere | METTL21B | 1.77E-18 |
| GTEx2015_v6 | [25954001](http://pubmed.gov/25954001) | Brain_Cerebellum | METTL21B | 7.85E-21 |
| GTEx2015_v6 | [25954001](http://pubmed.gov/25954001) | Brain_Cortex | METTL21B | 2.05E-15 |
| GTEx2015_v6 | [25954001](http://pubmed.gov/25954001) | Brain_Frontal_Cortex_BA9 | METTL21B | 2.42E-10 |
| GTEx2015_v6 | [25954001](http://pubmed.gov/25954001) | Brain_Hippocampus | METTL21B | 2.29E-08 |
| GTEx2015_v6 | [25954001](http://pubmed.gov/25954001) | Brain_Hypothalamus | METTL21B | 7.37E-10 |
| GTEx2015_v6 | [25954001](http://pubmed.gov/25954001) | Brain_Nucleus_accumbens_basal_ganglia | METTL21B | 3.51E-17 |
| GTEx2015_v6 | [25954001](http://pubmed.gov/25954001) | Brain_Putamen_basal_ganglia | METTL21B | 2.23E-13 |
| GTEx2015_v6 | [25954001](http://pubmed.gov/25954001) | Brain_Putamen_basal_ganglia | RP11-571M6.17 | 3.22E-07 |
| GTEx2015_v6 | [25954001](http://pubmed.gov/25954001) | Breast_Mammary_Tissue | METTL21B | 9.99E-09 |
| GTEx2015_v6 | [25954001](http://pubmed.gov/25954001) | Cells_EBV-transformed_lymphocytes | METTL21B | 1.53E-14 |
| GTEx2015_v6 | [25954001](http://pubmed.gov/25954001) | Cells_Transformed_fibroblasts | METTL21B | 4.28E-48 |
| GTEx2015_v6 | [25954001](http://pubmed.gov/25954001) | Cells_Transformed_fibroblasts | TSFM | 2.63E-18 |
| GTEx2015_v6 | [25954001](http://pubmed.gov/25954001) | Cells_Transformed_fibroblasts | XRCC6BP1 | 4.83E-08 |
| GTEx2015_v6 | [25954001](http://pubmed.gov/25954001) | Colon_Sigmoid | METTL21B | 4.26E-12 |
| GTEx2015_v6 | [25954001](http://pubmed.gov/25954001) | Colon_Sigmoid | XRCC6BP1 | 8.35E-07 |
| GTEx2015_v6 | [25954001](http://pubmed.gov/25954001) | Colon_Transverse | METTL21B | 6.97E-15 |
| GTEx2015_v6 | [25954001](http://pubmed.gov/25954001) | Esophagus_Gastroesophageal_Junction | METTL21B | 1.32E-12 |
| GTEx2015_v6 | [25954001](http://pubmed.gov/25954001) | Esophagus_Mucosa | METTL21B | 6.06E-18 |
| GTEx2015_v6 | [25954001](http://pubmed.gov/25954001) | Esophagus_Mucosa | OS9 | 1.85E-05 |
| GTEx2015_v6 | [25954001](http://pubmed.gov/25954001) | Esophagus_Mucosa | XRCC6BP1 | 1.20E-06 |
| GTEx2015_v6 | [25954001](http://pubmed.gov/25954001) | Esophagus_Muscularis | METTL21B | 1.35E-23 |
| GTEx2015_v6 | [25954001](http://pubmed.gov/25954001) | Esophagus_Muscularis | RP11-571M6.7 | 1.82E-05 |
| GTEx2015_v6 | [25954001](http://pubmed.gov/25954001) | Esophagus_Muscularis | TSFM | 9.10E-09 |
| GTEx2015_v6 | [25954001](http://pubmed.gov/25954001) | Esophagus_Muscularis | XRCC6BP1 | 2.81E-07 |
| GTEx2015_v6 | [25954001](http://pubmed.gov/25954001) | Heart_Atrial_Appendage | METTL21B | 2.81E-10 |
| GTEx2015_v6 | [25954001](http://pubmed.gov/25954001) | Heart_Atrial_Appendage | TSFM | 1.30E-08 |
| GTEx2015_v6 | [25954001](http://pubmed.gov/25954001) | Heart_Atrial_Appendage | XRCC6BP1 | 4.59E-08 |
| GTEx2015_v6 | [25954001](http://pubmed.gov/25954001) | Heart_Left_Ventricle | METTL21B | 5.20E-07 |
| GTEx2015_v6 | [25954001](http://pubmed.gov/25954001) | Heart_Left_Ventricle | SLC26A10 | 2.85E-08 |
| GTEx2015_v6 | [25954001](http://pubmed.gov/25954001) | Heart_Left_Ventricle | TSFM | 5.74E-07 |
| GTEx2015_v6 | [25954001](http://pubmed.gov/25954001) | Heart_Left_Ventricle | XRCC6BP1 | 1.78E-08 |
| GTEx2015_v6 | [25954001](http://pubmed.gov/25954001) | Lung | METTL21B | 1.35E-19 |
| GTEx2015_v6 | [25954001](http://pubmed.gov/25954001) | Lung | XRCC6BP1 | 2.43E-08 |
| GTEx2015_v6 | [25954001](http://pubmed.gov/25954001) | Muscle_Skeletal | METTL21B | 2.77E-15 |
| GTEx2015_v6 | [25954001](http://pubmed.gov/25954001) | Muscle_Skeletal | RP11-571M6.17 | 3.03E-06 |
| GTEx2015_v6 | [25954001](http://pubmed.gov/25954001) | Muscle_Skeletal | TSFM | 1.82E-13 |
| GTEx2015_v6 | [25954001](http://pubmed.gov/25954001) | Muscle_Skeletal | XRCC6BP1 | 5.81E-14 |
| GTEx2015_v6 | [25954001](http://pubmed.gov/25954001) | Nerve_Tibial | METTL21B | 7.29E-25 |
| GTEx2015_v6 | [25954001](http://pubmed.gov/25954001) | Nerve_Tibial | XRCC6BP1 | 2.36E-11 |
| GTEx2015_v6 | [25954001](http://pubmed.gov/25954001) | Pancreas | METTL21B | 1.58E-13 |
| GTEx2015_v6 | [25954001](http://pubmed.gov/25954001) | Pituitary | METTL21B | 4.74E-08 |
| GTEx2015_v6 | [25954001](http://pubmed.gov/25954001) | Skin_Not_Sun_Exposed_Suprapubic | METTL21B | 4.24E-10 |
| GTEx2015_v6 | [25954001](http://pubmed.gov/25954001) | Skin_Sun_Exposed_Lower_leg | METTL21B | 6.65E-26 |
| GTEx2015_v6 | [25954001](http://pubmed.gov/25954001) | Skin_Sun_Exposed_Lower_leg | XRCC6BP1 | 1.11E-07 |
| GTEx2015_v6 | [25954001](http://pubmed.gov/25954001) | Spleen | METTL21B | 8.35E-08 |
| GTEx2015_v6 | [25954001](http://pubmed.gov/25954001) | Stomach | METTL21B | 9.51E-14 |
| GTEx2015_v6 | [25954001](http://pubmed.gov/25954001) | Testis | TSFM | 2.26E-05 |
| GTEx2015_v6 | [25954001](http://pubmed.gov/25954001) | Thyroid | CYP27B1 | 3.91E-37 |
| GTEx2015_v6 | [25954001](http://pubmed.gov/25954001) | Thyroid | METTL1 | 1.20E-13 |
| GTEx2015_v6 | [25954001](http://pubmed.gov/25954001) | Thyroid | TSFM | 2.26E-06 |
| GTEx2015_v6 | [25954001](http://pubmed.gov/25954001) | Thyroid | XRCC6BP1 | 1.95E-10 |
| GTEx2015_v6 | [25954001](http://pubmed.gov/25954001) | Vagina | METTL21B | 2.62E-06 |
| GTEx2015_v6 | [25954001](http://pubmed.gov/25954001) | Whole_Blood | METTL21B | 2.22E-26 |
| GTEx2015_v6 | [25954001](http://pubmed.gov/25954001) | Whole_Blood | XRCC6BP1 | 5.89E-11 |
| Gibbs2010 | [20485568](http://pubmed.gov/20485568) | Cerbellum | FAM119B | 1.01E-11 |
| Gibbs2010 | [20485568](http://pubmed.gov/20485568) | FrontalCortex | FAM119B | 1.55E-12 |
| Gibbs2010 | [20485568](http://pubmed.gov/20485568) | Pons | FAM119B | 2.83E-08 |
| Lappalainen2013 | [24037378](http://pubmed.gov/24037378) | Lymphoblastoid_EUR_exonlevel | ENSG00000123297.11_58176372_58176690 | 3.02E-13 |
| Lappalainen2013 | [24037378](http://pubmed.gov/24037378) | Lymphoblastoid_EUR_exonlevel | ENSG00000123297.11_58176893_58177066 | 1.22E-23 |
| Lappalainen2013 | [24037378](http://pubmed.gov/24037378) | Lymphoblastoid_EUR_exonlevel | ENSG00000123297.11_58179946_58180074 | 5.21E-16 |
| Lappalainen2013 | [24037378](http://pubmed.gov/24037378) | Lymphoblastoid_EUR_exonlevel | ENSG00000123297.11_58180823_58180945 | 3.50E-21 |
| Lappalainen2013 | [24037378](http://pubmed.gov/24037378) | Lymphoblastoid_EUR_exonlevel | ENSG00000123297.11_58186769_58186856 | 1.23E-18 |
| Lappalainen2013 | [24037378](http://pubmed.gov/24037378) | Lymphoblastoid_EUR_exonlevel | ENSG00000123297.11_58189960_58191370 | 2.69E-29 |
| Lappalainen2013 | [24037378](http://pubmed.gov/24037378) | Lymphoblastoid_EUR_exonlevel | ENSG00000123427.11_58174038_58176324 | 9.60E-63 |
| Lappalainen2013 | [24037378](http://pubmed.gov/24037378) | Lymphoblastoid_EUR_exonlevel | ENSG00000135452.5_58140372_58140503 | 7.76E-06 |
| Lappalainen2013 | [24037378](http://pubmed.gov/24037378) | Lymphoblastoid_EUR_exonlevel | ENSG00000135452.5_58140801_58140914 | 1.86E-07 |
| Lappalainen2013 | [24037378](http://pubmed.gov/24037378) | Lymphoblastoid_EUR_exonlevel | ENSG00000135506.11_58111929_58112204 | 7.30E-06 |
| Lappalainen2013 | [24037378](http://pubmed.gov/24037378) | Lymphoblastoid_EUR_exonlevel | ENSG00000166896.3_58345541_58345678 | 1.15E-06 |
| Lappalainen2013 | [24037378](http://pubmed.gov/24037378) | Lymphoblastoid_EUR_exonlevel | ENSG00000257921.1_58176893_58177066 | 5.04E-20 |
| Lappalainen2013 | [24037378](http://pubmed.gov/24037378) | Lymphoblastoid_EUR_exonlevel | ENSG00000257921.1_58179946_58180030 | 4.34E-09 |
| Lappalainen2013 | [24037378](http://pubmed.gov/24037378) | Lymphoblastoid_EUR_genelevel | TSFM | 5.93E-08 |
| Stranger2008 | [17873874](http://pubmed.gov/17873874) | Lymphoblastoid | FAM119B | 1.58E-19 |
| Westra2013 | [24013639](http://pubmed.gov/24013639) | Whole_Blood | FAM119B | 9.81E-198 |
| Westra2013 | [24013639](http://pubmed.gov/24013639) | Whole_Blood | TSFM | 4.61E-22 |
| Westra2013 | [24013639](http://pubmed.gov/24013639) | Whole_Blood | TSPAN31 | 1.54E-38 |

This results come form HaploReg v4.1 (<https://pubs.broadinstitute.org/mammals/haploreg/haploreg.php>)

Table S6. All colocalization tests results across leprosy loci and eQTL datasets.

| Locus | Signal | Dataset | Gene Symbol | Num snps | PP.H0.abf | PP.H1.abf | PP.H2.abf | PP.H3.abf | PP.H4.abf | Top Coloc SNP | PP of SNP |
| --- | --- | --- | --- | --- | --- | --- | --- | --- | --- | --- | --- |
| chr12:57,665,085-58,665,085 | All | gtex.Whole_Blood | *METTL1* | 964 | 0.000 | 0.865 | 0.000 | 0.066 | 0.069 | rs10877013 | 0.073 |
| chr12:57,665,085-58,665,085 | All | gtex.Whole_Blood | *CYP27B1* | 964 | 0.000 | 0.893 | 0.000 | 0.058 | 0.049 | rs10877013 | 0.072 |
| chr12:57,665,085-58,665,085 | All | gtex.Whole_Blood | *GLI1* | 964 | 0.000 | 0.861 | 0.000 | 0.081 | 0.057 | rs10877013 | 0.073 |
| chr12:57,665,085-58,665,085 | All | gtex.Whole_Blood | *TSFM* | 964 | 0.000 | 0.214 | 0.000 | 0.082 | 0.704 | rs10877013 | 0.097 |
| chr12:57,665,085-58,665,085 | All | gtex.Whole_Blood | *ARHGAP9* | 964 | 0.000 | 0.000 | 0.000 | 1.000 | 0.000 | rs10877013 | 0.000 |
| chr12:57,665,085-58,665,085 | All | gtex.Whole_Blood | *METTL21B* | 964 | 0.000 | 0.000 | 0.000 | 0.045 | **0.955** | rs923829 | 0.160 |
| chr12:57,665,085-58,665,085 | All | gtex.Whole_Blood | *AGAP2* | 964 | 0.000 | 0.842 | 0.000 | 0.069 | 0.089 | rs10877013 | 0.077 |
| chr12:57,665,085-58,665,085 | All | gtex.Whole_Blood | *CDK4* | 964 | 0.000 | 0.854 | 0.000 | 0.093 | 0.053 | rs10877013 | 0.078 |
| chr12:57,665,085-58,665,085 | All | gtex.Whole_Blood | *TSPAN31* | 964 | 0.000 | 0.365 | 0.000 | 0.080 | 0.555 | rs2291617 | 0.067 |
| chr12:57,665,085-58,665,085 | All | gtex.Whole_Blood | *OS9* | 964 | 0.000 | 0.227 | 0.000 | 0.730 | 0.043 | rs10877013 | 0.068 |
| chr12:57,665,085-58,665,085 | All | gtex.Whole_Blood | *INHBE* | 964 | 0.000 | 0.747 | 0.000 | 0.188 | 0.065 | rs10877013 | 0.061 |
| chr12:57,665,085-58,665,085 | All | gtex.Whole_Blood | *KIF5A* | 964 | 0.000 | 0.845 | 0.000 | 0.091 | 0.064 | rs10877013 | 0.069 |
| chr12:57,665,085-58,665,085 | All | gtex.Whole_Blood | *ATP23* | 964 | 0.000 | 0.000 | 0.000 | 1.000 | 0.000 | rs703842 | 0.000 |
| chr12:57,665,085-58,665,085 | All | gtex.Whole_Blood | *PIP4K2C* | 964 | 0.000 | 0.824 | 0.000 | 0.110 | 0.067 | rs10877013 | 0.079 |
| chr12:57,665,085-58,665,085 | All | gtex.Whole_Blood | *MBD6* | 964 | 0.000 | 0.892 | 0.000 | 0.062 | 0.046 | rs10877013 | 0.074 |
| chr12:57,665,085-58,665,085 | All | gtex.Whole_Blood | *DDIT3* | 964 | 0.000 | 0.841 | 0.000 | 0.072 | 0.087 | rs10877013 | 0.071 |
| chr12:57,665,085-58,665,085 | All | gtex.Whole_Blood | *DCTN2* | 964 | 0.000 | 0.878 | 0.000 | 0.072 | 0.050 | rs10877013 | 0.079 |
| chr12:57,665,085-58,665,085 | All | gtex.Whole_Blood | *CTDSP2* | 964 | 0.000 | 0.846 | 0.000 | 0.067 | 0.087 | rs4646536 | 0.092 |
| chr12:57,665,085-58,665,085 | All | gtex.Whole_Blood | *DTX3* | 964 | 0.000 | 0.873 | 0.000 | 0.082 | 0.045 | rs10877013 | 0.075 |
| chr12:57,665,085-58,665,085 | All | gtex.Whole_Blood | *R3HDM2* | 964 | 0.000 | 0.869 | 0.000 | 0.079 | 0.052 | rs10877013 | 0.083 |
| chr12:57,665,085-58,665,085 | All | gtex.Whole_Blood | *ARHGEF25* | 964 | 0.000 | 0.867 | 0.000 | 0.079 | 0.054 | rs10877013 | 0.076 |
| chr12:57,665,085-58,665,085 | All | gtex.Whole_Blood | *AGAP2-AS1* | 964 | 0.000 | 0.873 | 0.000 | 0.065 | 0.062 | rs10877013 | 0.070 |
| chr12:57,665,085-58,665,085 | All | gtex.Whole_Blood | *GIHCG* | 964 | 0.000 | 0.005 | 0.000 | 0.995 | 0.000 | rs10877013 | 0.069 |
| chr12:57,665,085-58,665,085 | All | gtex.Nerve_Tibial | *METTL1* | 964 | 0.000 | 0.873 | 0.000 | 0.077 | 0.050 | rs10877013 | 0.075 |
| chr12:57,665,085-58,665,085 | All | gtex.Nerve_Tibial | *CYP27B1* | 964 | 0.000 | 0.009 | 0.000 | 0.038 | **0.953** | rs2291617 | 0.090 |
| chr12:57,665,085-58,665,085 | All | gtex.Nerve_Tibial | *GLI1* | 964 | 0.000 | 0.845 | 0.000 | 0.095 | 0.061 | rs10877013 | 0.068 |
| chr12:57,665,085-58,665,085 | All | gtex.Nerve_Tibial | *TSFM* | 964 | 0.000 | 0.000 | 0.000 | 0.037 | **0.963** | rs2291617 | 0.121 |
| chr12:57,665,085-58,665,085 | All | gtex.Nerve_Tibial | *ARHGAP9* | 964 | 0.000 | 0.167 | 0.000 | 0.812 | 0.021 | rs10877013 | 0.074 |
| chr12:57,665,085-58,665,085 | All | gtex.Nerve_Tibial | *METTL21B* | 964 | 0.000 | 0.000 | 0.000 | 0.048 | **0.952** | rs8181644 | 0.304 |
| chr12:57,665,085-58,665,085 | All | gtex.Nerve_Tibial | *AGAP2* | 964 | 0.000 | 0.874 | 0.000 | 0.078 | 0.047 | rs10877013 | 0.071 |
| chr12:57,665,085-58,665,085 | All | gtex.Nerve_Tibial | *CDK4* | 964 | 0.000 | 0.395 | 0.000 | 0.279 | 0.326 | rs2291617 | 0.073 |
| chr12:57,665,085-58,665,085 | All | gtex.Nerve_Tibial | *TSPAN31* | 964 | 0.000 | 0.678 | 0.000 | 0.112 | 0.210 | rs8181644 | 0.064 |
| chr12:57,665,085-58,665,085 | All | gtex.Nerve_Tibial | *B4GALNT1* | 964 | 0.000 | 0.000 | 0.000 | 0.999 | 0.001 | rs10877013 | 0.000 |
| chr12:57,665,085-58,665,085 | All | gtex.Nerve_Tibial | *SLC26A10* | 964 | 0.000 | 0.001 | 0.000 | 0.996 | 0.002 | rs10877013 | 0.004 |
| chr12:57,665,085-58,665,085 | All | gtex.Nerve_Tibial | *OS9* | 964 | 0.000 | 0.589 | 0.000 | 0.254 | 0.156 | rs2291617 | 0.068 |
| chr12:57,665,085-58,665,085 | All | gtex.Nerve_Tibial | *INHBE* | 964 | 0.000 | 0.831 | 0.000 | 0.087 | 0.083 | rs10877013 | 0.060 |
| chr12:57,665,085-58,665,085 | All | gtex.Nerve_Tibial | *KIF5A* | 964 | 0.000 | 0.609 | 0.000 | 0.143 | 0.249 | rs2291617 | 0.075 |
| chr12:57,665,085-58,665,085 | All | gtex.Nerve_Tibial | *ATP23* | 964 | 0.000 | 0.000 | 0.000 | 1.000 | 0.000 | rs10877018 | 0.000 |
| chr12:57,665,085-58,665,085 | All | gtex.Nerve_Tibial | *PIP4K2C* | 964 | 0.000 | 0.291 | 0.000 | 0.402 | 0.307 | rs2291617 | 0.073 |
| chr12:57,665,085-58,665,085 | All | gtex.Nerve_Tibial | *MBD6* | 964 | 0.000 | 0.106 | 0.000 | 0.888 | 0.005 | rs10877013 | 0.076 |
| chr12:57,665,085-58,665,085 | All | gtex.Nerve_Tibial | *INHBC* | 964 | 0.000 | 0.859 | 0.000 | 0.091 | 0.050 | rs10877013 | 0.072 |
| chr12:57,665,085-58,665,085 | All | gtex.Nerve_Tibial | *DDIT3* | 964 | 0.000 | 0.885 | 0.000 | 0.060 | 0.056 | rs10877013 | 0.074 |
| chr12:57,665,085-58,665,085 | All | gtex.Nerve_Tibial | *DCTN2* | 964 | 0.000 | 0.855 | 0.000 | 0.101 | 0.043 | rs10877013 | 0.076 |
| chr12:57,665,085-58,665,085 | All | gtex.Nerve_Tibial | *CTDSP2* | 964 | 0.000 | 0.887 | 0.000 | 0.066 | 0.046 | rs10877013 | 0.076 |
| chr12:57,665,085-58,665,085 | All | gtex.Nerve_Tibial | *DTX3* | 964 | 0.000 | 0.861 | 0.000 | 0.088 | 0.051 | rs10877013 | 0.077 |
| chr12:57,665,085-58,665,085 | All | gtex.Nerve_Tibial | *R3HDM2* | 964 | 0.000 | 0.839 | 0.000 | 0.098 | 0.063 | rs10877013 | 0.064 |
| chr12:57,665,085-58,665,085 | All | gtex.Nerve_Tibial | *ARHGEF25* | 964 | 0.000 | 0.000 | 0.000 | 0.999 | 0.001 | rs10877013 | 0.000 |
| chr12:57,665,085-58,665,085 | All | gtex.Nerve_Tibial | *AGAP2-AS1* | 964 | 0.000 | 0.820 | 0.000 | 0.098 | 0.082 | rs10877013 | 0.062 |
| chr12:57,665,085-58,665,085 | All | gtex.Nerve_Tibial | *GIHCG* | 964 | 0.000 | 0.097 | 0.000 | 0.889 | 0.014 | rs703842 | 0.096 |
| chr12:57,665,085-58,665,085 | All | gtex.Skin_Sun_Exposed_Lower_leg | *METTL1* | 964 | 0.000 | 0.892 | 0.000 | 0.063 | 0.045 | rs10877013 | 0.074 |
| chr12:57,665,085-58,665,085 | All | gtex.Skin_Sun_Exposed_Lower_leg | *CYP27B1* | 964 | 0.000 | 0.006 | 0.000 | 0.047 | **0.947** | rs10877012 | 0.104 |
| chr12:57,665,085-58,665,085 | All | gtex.Skin_Sun_Exposed_Lower_leg | *GLI1* | 964 | 0.000 | 0.875 | 0.000 | 0.069 | 0.055 | rs10877013 | 0.073 |
| chr12:57,665,085-58,665,085 | All | gtex.Skin_Sun_Exposed_Lower_leg | *TSFM* | 964 | 0.000 | 0.110 | 0.000 | 0.059 | **0.832** | rs2291617 | 0.057 |
| chr12:57,665,085-58,665,085 | All | gtex.Skin_Sun_Exposed_Lower_leg | *ARHGAP9* | 964 | 0.000 | 0.001 | 0.000 | 0.999 | 0.000 | rs10877013 | 0.070 |
| chr12:57,665,085-58,665,085 | All | gtex.Skin_Sun_Exposed_Lower_leg | *METTL21B* | 964 | 0.000 | 0.000 | 0.000 | 0.047 | **0.953** | rs8181644 | 0.364 |
| chr12:57,665,085-58,665,085 | All | gtex.Skin_Sun_Exposed_Lower_leg | *AGAP2* | 964 | 0.000 | 0.333 | 0.000 | 0.119 | 0.549 | rs10877013 | 0.110 |
| chr12:57,665,085-58,665,085 | All | gtex.Skin_Sun_Exposed_Lower_leg | *CDK4* | 964 | 0.000 | 0.887 | 0.000 | 0.067 | 0.046 | rs10877013 | 0.073 |
| chr12:57,665,085-58,665,085 | All | gtex.Skin_Sun_Exposed_Lower_leg | *TSPAN31* | 964 | 0.000 | 0.842 | 0.000 | 0.068 | 0.090 | rs1021469 | 0.054 |
| chr12:57,665,085-58,665,085 | All | gtex.Skin_Sun_Exposed_Lower_leg | *B4GALNT1* | 964 | 0.000 | 0.796 | 0.000 | 0.155 | 0.049 | rs10877013 | 0.083 |
| chr12:57,665,085-58,665,085 | All | gtex.Skin_Sun_Exposed_Lower_leg | *SLC26A10* | 964 | 0.000 | 0.674 | 0.000 | 0.112 | 0.214 | rs10877013 | 0.070 |
| chr12:57,665,085-58,665,085 | All | gtex.Skin_Sun_Exposed_Lower_leg | *OS9* | 964 | 0.000 | 0.689 | 0.000 | 0.275 | 0.036 | rs10877013 | 0.073 |
| chr12:57,665,085-58,665,085 | All | gtex.Skin_Sun_Exposed_Lower_leg | *INHBE* | 964 | 0.000 | 0.861 | 0.000 | 0.094 | 0.045 | rs10877013 | 0.074 |
| chr12:57,665,085-58,665,085 | All | gtex.Skin_Sun_Exposed_Lower_leg | *KIF5A* | 964 | 0.000 | 0.790 | 0.000 | 0.074 | 0.136 | rs10877013 | 0.109 |
| chr12:57,665,085-58,665,085 | All | gtex.Skin_Sun_Exposed_Lower_leg | *ATP23* | 964 | 0.000 | 0.000 | 0.000 | 1.000 | 0.000 | rs7965287 | 0.000 |
| chr12:57,665,085-58,665,085 | All | gtex.Skin_Sun_Exposed_Lower_leg | *PIP4K2C* | 964 | 0.000 | 0.824 | 0.000 | 0.078 | 0.098 | rs10877013 | 0.062 |
| chr12:57,665,085-58,665,085 | All | gtex.Skin_Sun_Exposed_Lower_leg | *MBD6* | 964 | 0.000 | 0.597 | 0.000 | 0.341 | 0.062 | rs10877013 | 0.076 |
| chr12:57,665,085-58,665,085 | All | gtex.Skin_Sun_Exposed_Lower_leg | *INHBC* | 964 | 0.000 | 0.859 | 0.000 | 0.084 | 0.057 | rs10877013 | 0.075 |
| chr12:57,665,085-58,665,085 | All | gtex.Skin_Sun_Exposed_Lower_leg | *DDIT3* | 964 | 0.000 | 0.804 | 0.000 | 0.102 | 0.094 | rs10877013 | 0.072 |
| chr12:57,665,085-58,665,085 | All | gtex.Skin_Sun_Exposed_Lower_leg | *DCTN2* | 964 | 0.000 | 0.890 | 0.000 | 0.065 | 0.045 | rs10877013 | 0.075 |
| chr12:57,665,085-58,665,085 | All | gtex.Skin_Sun_Exposed_Lower_leg | *CTDSP2* | 964 | 0.000 | 0.866 | 0.000 | 0.057 | 0.076 | rs10877013 | 0.071 |
| chr12:57,665,085-58,665,085 | All | gtex.Skin_Sun_Exposed_Lower_leg | *DTX3* | 964 | 0.000 | 0.877 | 0.000 | 0.074 | 0.050 | rs10877013 | 0.077 |
| chr12:57,665,085-58,665,085 | All | gtex.Skin_Sun_Exposed_Lower_leg | *R3HDM2* | 964 | 0.000 | 0.847 | 0.000 | 0.073 | 0.080 | rs10877013 | 0.075 |
| chr12:57,665,085-58,665,085 | All | gtex.Skin_Sun_Exposed_Lower_leg | *ARHGEF25* | 964 | 0.000 | 0.018 | 0.000 | 0.957 | 0.025 | rs703842 | 0.014 |
| chr12:57,665,085-58,665,085 | All | gtex.Skin_Sun_Exposed_Lower_leg | *AGAP2-AS1* | 964 | 0.000 | 0.879 | 0.000 | 0.077 | 0.044 | rs10877013 | 0.076 |
| chr12:57,665,085-58,665,085 | All | gtex.Skin_Sun_Exposed_Lower_leg | *GIHCG* | 964 | 0.000 | 0.826 | 0.000 | 0.091 | 0.083 | rs10877013 | 0.099 |
| chr12:57,665,085-58,665,085 | All | gtex.Skin_Not_Sun_Exposed_Suprapubic | *METTL1* | 964 | 0.000 | 0.890 | 0.000 | 0.065 | 0.045 | rs10877013 | 0.074 |
| chr12:57,665,085-58,665,085 | All | gtex.Skin_Not_Sun_Exposed_Suprapubic | *CYP27B1* | 964 | 0.000 | 0.845 | 0.000 | 0.070 | 0.085 | rs10877013 | 0.113 |
| chr12:57,665,085-58,665,085 | All | gtex.Skin_Not_Sun_Exposed_Suprapubic | *GLI1* | 964 | 0.000 | 0.881 | 0.000 | 0.064 | 0.055 | rs10877013 | 0.084 |
| chr12:57,665,085-58,665,085 | All | gtex.Skin_Not_Sun_Exposed_Suprapubic | *TSFM* | 964 | 0.000 | 0.199 | 0.000 | 0.133 | 0.668 | rs10877012 | 0.076 |
| chr12:57,665,085-58,665,085 | All | gtex.Skin_Not_Sun_Exposed_Suprapubic | *ARHGAP9* | 964 | 0.000 | 0.005 | 0.000 | 0.995 | 0.001 | rs10877013 | 0.111 |
| chr12:57,665,085-58,665,085 | All | gtex.Skin_Not_Sun_Exposed_Suprapubic | *METTL21B* | 964 | 0.000 | 0.000 | 0.000 | 0.052 | **0.948** | rs923829 | 0.349 |
| chr12:57,665,085-58,665,085 | All | gtex.Skin_Not_Sun_Exposed_Suprapubic | *AGAP2* | 964 | 0.000 | 0.219 | 0.000 | 0.727 | 0.055 | rs2291617 | 0.063 |
| chr12:57,665,085-58,665,085 | All | gtex.Skin_Not_Sun_Exposed_Suprapubic | *CDK4* | 964 | 0.000 | 0.803 | 0.000 | 0.101 | 0.096 | rs10877015 | 0.062 |
| chr12:57,665,085-58,665,085 | All | gtex.Skin_Not_Sun_Exposed_Suprapubic | *TSPAN31* | 964 | 0.000 | 0.760 | 0.000 | 0.106 | 0.133 | rs2291617 | 0.106 |
| chr12:57,665,085-58,665,085 | All | gtex.Skin_Not_Sun_Exposed_Suprapubic | *B4GALNT1* | 964 | 0.000 | 0.765 | 0.000 | 0.189 | 0.046 | rs10877013 | 0.063 |
| chr12:57,665,085-58,665,085 | All | gtex.Skin_Not_Sun_Exposed_Suprapubic | *SLC26A10* | 964 | 0.000 | 0.849 | 0.000 | 0.101 | 0.050 | rs10877013 | 0.080 |
| chr12:57,665,085-58,665,085 | All | gtex.Skin_Not_Sun_Exposed_Suprapubic | *OS9* | 964 | 0.000 | 0.842 | 0.000 | 0.112 | 0.045 | rs10877013 | 0.071 |
| chr12:57,665,085-58,665,085 | All | gtex.Skin_Not_Sun_Exposed_Suprapubic | *INHBE* | 964 | 0.000 | 0.881 | 0.000 | 0.071 | 0.047 | rs10877013 | 0.076 |
| chr12:57,665,085-58,665,085 | All | gtex.Skin_Not_Sun_Exposed_Suprapubic | *KIF5A* | 964 | 0.000 | 0.813 | 0.000 | 0.106 | 0.080 | rs10877013 | 0.106 |
| chr12:57,665,085-58,665,085 | All | gtex.Skin_Not_Sun_Exposed_Suprapubic | *ATP23* | 964 | 0.000 | 0.000 | 0.000 | 1.000 | 0.000 | rs1021469 | 0.000 |
| chr12:57,665,085-58,665,085 | All | gtex.Skin_Not_Sun_Exposed_Suprapubic | *PIP4K2C* | 964 | 0.000 | 0.711 | 0.000 | 0.104 | 0.185 | rs10877013 | 0.078 |
| chr12:57,665,085-58,665,085 | All | gtex.Skin_Not_Sun_Exposed_Suprapubic | *MBD6* | 964 | 0.000 | 0.560 | 0.000 | 0.107 | 0.333 | rs2291617 | 0.109 |
| chr12:57,665,085-58,665,085 | All | gtex.Skin_Not_Sun_Exposed_Suprapubic | *INHBC* | 964 | 0.000 | 0.850 | 0.000 | 0.077 | 0.073 | rs10877013 | 0.114 |
| chr12:57,665,085-58,665,085 | All | gtex.Skin_Not_Sun_Exposed_Suprapubic | *DDIT3* | 964 | 0.000 | 0.866 | 0.000 | 0.080 | 0.054 | rs10877013 | 0.083 |
| chr12:57,665,085-58,665,085 | All | gtex.Skin_Not_Sun_Exposed_Suprapubic | *DCTN2* | 964 | 0.000 | 0.868 | 0.000 | 0.077 | 0.055 | rs10877013 | 0.070 |
| chr12:57,665,085-58,665,085 | All | gtex.Skin_Not_Sun_Exposed_Suprapubic | *CTDSP2* | 964 | 0.000 | 0.865 | 0.000 | 0.091 | 0.044 | rs10877013 | 0.074 |
| chr12:57,665,085-58,665,085 | All | gtex.Skin_Not_Sun_Exposed_Suprapubic | *DTX3* | 964 | 0.000 | 0.881 | 0.000 | 0.068 | 0.051 | rs10877013 | 0.079 |
| chr12:57,665,085-58,665,085 | All | gtex.Skin_Not_Sun_Exposed_Suprapubic | *R3HDM2* | 964 | 0.000 | 0.829 | 0.000 | 0.090 | 0.081 | rs4646536 | 0.076 |
| chr12:57,665,085-58,665,085 | All | gtex.Skin_Not_Sun_Exposed_Suprapubic | *ARHGEF25* | 964 | 0.000 | 0.340 | 0.000 | 0.637 | 0.023 | rs10877013 | 0.057 |
| chr12:57,665,085-58,665,085 | All | gtex.Skin_Not_Sun_Exposed_Suprapubic | *AGAP2-AS1* | 964 | 0.000 | 0.893 | 0.000 | 0.063 | 0.045 | rs10877013 | 0.075 |
| chr12:57,665,085-58,665,085 | All | gtex.Skin_Not_Sun_Exposed_Suprapubic | *GIHCG* | 964 | 0.000 | 0.850 | 0.000 | 0.085 | 0.065 | rs10877013 | 0.083 |
| chr15:74,587,571-75,587,571 | All | gtex.Whole_Blood | *CSK* | 1354 | 0.000 | 0.793 | 0.000 | 0.162 | 0.045 | rs117618569 | 0.170 |
| chr15:74,587,571-75,587,571 | All | gtex.Whole_Blood | *PPCDC* | 1354 | 0.000 | 0.729 | 0.000 | 0.209 | 0.062 | rs117618569 | 0.178 |
| chr15:74,587,571-75,587,571 | All | gtex.Whole_Blood | *SEMA7A* | 1354 | 0.000 | 0.803 | 0.000 | 0.143 | 0.054 | rs117618569 | 0.221 |
| chr15:74,587,571-75,587,571 | All | gtex.Whole_Blood | *CYP11A1* | 1354 | 0.000 | 0.818 | 0.000 | 0.113 | 0.069 | rs16972628 | 0.170 |
| chr15:74,587,571-75,587,571 | All | gtex.Whole_Blood | *CYP1A1* | 1354 | 0.000 | 0.867 | 0.000 | 0.081 | 0.052 | rs117618569 | 0.183 |
| chr15:74,587,571-75,587,571 | All | gtex.Whole_Blood | *ULK3* | 1354 | 0.000 | 0.000 | 0.000 | 1.000 | 0.000 | rs59567621 | 0.000 |
| chr15:74,587,571-75,587,571 | All | gtex.Whole_Blood | *SCAMP2* | 1354 | 0.000 | 0.000 | 0.000 | 1.000 | 0.000 | rs16972486 | 0.000 |
| chr15:74,587,571-75,587,571 | All | gtex.Whole_Blood | *LMAN1L* | 1354 | 0.000 | 0.502 | 0.000 | 0.143 | 0.355 | rs4488423 | 0.468 |
| chr15:74,587,571-75,587,571 | All | gtex.Whole_Blood | *C15orf39* | 1354 | 0.000 | 0.828 | 0.000 | 0.117 | 0.055 | rs117618569 | 0.157 |
| chr15:74,587,571-75,587,571 | All | gtex.Whole_Blood | *RPP25* | 1354 | 0.000 | 0.825 | 0.000 | 0.108 | 0.067 | rs4488423 | 0.191 |
| chr15:74,587,571-75,587,571 | All | gtex.Whole_Blood | *COX5A* | 1354 | 0.000 | 0.836 | 0.000 | 0.115 | 0.049 | rs117618569 | 0.169 |
| chr15:74,587,571-75,587,571 | All | gtex.Whole_Blood | *FAM219B* | 1354 | 0.000 | 0.798 | 0.000 | 0.134 | 0.067 | rs16972628 | 0.209 |
| chr15:74,587,571-75,587,571 | All | gtex.Whole_Blood | *MPI* | 1354 | 0.000 | 0.000 | 0.000 | 1.000 | 0.000 | rs16972486 | 0.003 |
| chr15:74,587,571-75,587,571 | All | gtex.Whole_Blood | *EDC3* | 1354 | 0.000 | 0.797 | 0.000 | 0.156 | 0.047 | rs117618569 | 0.156 |
| chr15:74,587,571-75,587,571 | All | gtex.Whole_Blood | *CLK3* | 1354 | 0.000 | 0.853 | 0.000 | 0.091 | 0.056 | rs117618569 | 0.209 |
| chr15:74,587,571-75,587,571 | All | gtex.Whole_Blood | *ARID3B* | 1354 | 0.000 | 0.793 | 0.000 | 0.136 | 0.071 | rs4488423 | 0.252 |
| chr15:74,587,571-75,587,571 | All | gtex.Whole_Blood | *SCAMP5* | 1354 | 0.000 | 0.771 | 0.000 | 0.165 | 0.064 | rs56992651 | 0.201 |
| chr15:74,587,571-75,587,571 | All | gtex.Whole_Blood | *CPLX3* | 1354 | 0.000 | 0.388 | 0.000 | 0.141 | 0.471 | rs4488423 | 0.325 |
| chr15:74,587,571-75,587,571 | All | gtex.Whole_Blood | *UBL7-DT* | 1354 | 0.000 | 0.838 | 0.000 | 0.105 | 0.057 | rs117618569 | 0.146 |
| chr15:74,587,571-75,587,571 | All | gtex.Nerve_Tibial | *CSK* | 1354 | 0.000 | 0.847 | 0.000 | 0.097 | 0.056 | rs117618569 | 0.143 |
| chr15:74,587,571-75,587,571 | All | gtex.Nerve_Tibial | *PPCDC* | 1354 | 0.000 | 0.000 | 0.000 | 1.000 | 0.000 | rs117618569 | 0.000 |
| chr15:74,587,571-75,587,571 | All | gtex.Nerve_Tibial | *SEMA7A* | 1354 | 0.000 | 0.011 | 0.000 | 0.988 | 0.001 | rs117618569 | 0.137 |
| chr15:74,587,571-75,587,571 | All | gtex.Nerve_Tibial | *CYP11A1* | 1354 | 0.000 | 0.238 | 0.000 | 0.743 | 0.019 | rs117618569 | 0.162 |
| chr15:74,587,571-75,587,571 | All | gtex.Nerve_Tibial | *CYP1A1* | 1354 | 0.000 | 0.790 | 0.000 | 0.158 | 0.052 | rs117618569 | 0.169 |
| chr15:74,587,571-75,587,571 | All | gtex.Nerve_Tibial | *ULK3* | 1354 | 0.000 | 0.654 | 0.000 | 0.303 | 0.043 | rs117618569 | 0.142 |
| chr15:74,587,571-75,587,571 | All | gtex.Nerve_Tibial | *SCAMP2* | 1354 | 0.000 | 0.578 | 0.000 | 0.276 | 0.146 | rs16972628 | 0.323 |
| chr15:74,587,571-75,587,571 | All | gtex.Nerve_Tibial | *C15orf39* | 1354 | 0.000 | 0.642 | 0.000 | 0.181 | 0.177 | rs117618569 | 0.131 |
| chr15:74,587,571-75,587,571 | All | gtex.Nerve_Tibial | *RPP25* | 1354 | 0.000 | 0.000 | 0.000 | 1.000 | 0.000 | rs16972628 | 0.000 |
| chr15:74,587,571-75,587,571 | All | gtex.Nerve_Tibial | *COX5A* | 1354 | 0.000 | 0.796 | 0.000 | 0.121 | 0.082 | rs4488423 | 0.125 |
| chr15:74,587,571-75,587,571 | All | gtex.Nerve_Tibial | *FAM219B* | 1354 | 0.000 | 0.838 | 0.000 | 0.112 | 0.050 | rs117618569 | 0.185 |
| chr15:74,587,571-75,587,571 | All | gtex.Nerve_Tibial | *MPI* | 1354 | 0.000 | 0.000 | 0.000 | 1.000 | 0.000 | rs16972628 | 0.000 |
| chr15:74,587,571-75,587,571 | All | gtex.Nerve_Tibial | *EDC3* | 1354 | 0.000 | 0.830 | 0.000 | 0.103 | 0.067 | rs117618569 | 0.142 |
| chr15:74,587,571-75,587,571 | All | gtex.Nerve_Tibial | *CLK3* | 1354 | 0.000 | 0.638 | 0.000 | 0.326 | 0.036 | rs117618569 | 0.171 |
| chr15:74,587,571-75,587,571 | All | gtex.Nerve_Tibial | *ARID3B* | 1354 | 0.000 | 0.019 | 0.000 | 0.979 | 0.003 | rs4488423 | 0.234 |
| chr15:74,587,571-75,587,571 | All | gtex.Nerve_Tibial | *SCAMP5* | 1354 | 0.000 | 0.000 | 0.000 | 1.000 | 0.000 | rs117618569 | 0.066 |
| chr15:74,587,571-75,587,571 | All | gtex.Nerve_Tibial | *CPLX3* | 1354 | 0.000 | 0.765 | 0.000 | 0.102 | 0.133 | rs4488423 | 0.362 |
| chr15:74,587,571-75,587,571 | All | gtex.Nerve_Tibial | *UBL7-DT* | 1354 | 0.000 | 0.824 | 0.000 | 0.092 | 0.084 | rs4488423 | 0.186 |
| chr15:74,587,571-75,587,571 | All | gtex.Nerve_Tibial | *PPIAP46* | 1354 | 0.000 | 0.748 | 0.000 | 0.166 | 0.086 | rs16972628 | 0.315 |
| chr15:74,587,571-75,587,571 | All | gtex.Skin_Sun_Exposed_Lower_leg | *CSK* | 1354 | 0.000 | 0.617 | 0.000 | 0.338 | 0.045 | rs117618569 | 0.189 |
| chr15:74,587,571-75,587,571 | All | gtex.Skin_Sun_Exposed_Lower_leg | *PPCDC* | 1354 | 0.000 | 0.000 | 0.000 | 1.000 | 0.000 | rs12437562 | 0.000 |
| chr15:74,587,571-75,587,571 | All | gtex.Skin_Sun_Exposed_Lower_leg | *SEMA7A* | 1354 | 0.000 | 0.764 | 0.000 | 0.182 | 0.054 | rs117618569 | 0.156 |
| chr15:74,587,571-75,587,571 | All | gtex.Skin_Sun_Exposed_Lower_leg | *CYP11A1* | 1354 | 0.000 | 0.800 | 0.000 | 0.117 | 0.082 | rs117429208 | 0.177 |
| chr15:74,587,571-75,587,571 | All | gtex.Skin_Sun_Exposed_Lower_leg | *CYP1A1* | 1354 | 0.000 | 0.858 | 0.000 | 0.093 | 0.049 | rs117618569 | 0.167 |
| chr15:74,587,571-75,587,571 | All | gtex.Skin_Sun_Exposed_Lower_leg | *ULK3* | 1354 | 0.000 | 0.457 | 0.000 | 0.507 | 0.036 | rs117618569 | 0.230 |
| chr15:74,587,571-75,587,571 | All | gtex.Skin_Sun_Exposed_Lower_leg | *SCAMP2* | 1354 | 0.000 | 0.002 | 0.000 | 0.998 | 0.000 | rs117618569 | 0.172 |
| chr15:74,587,571-75,587,571 | All | gtex.Skin_Sun_Exposed_Lower_leg | *CYP1A2* | 1354 | 0.000 | 0.849 | 0.000 | 0.092 | 0.058 | rs117618569 | 0.224 |
| chr15:74,587,571-75,587,571 | All | gtex.Skin_Sun_Exposed_Lower_leg | *LMAN1L* | 1354 | 0.000 | 0.840 | 0.000 | 0.085 | 0.075 | rs16972628 | 0.298 |
| chr15:74,587,571-75,587,571 | All | gtex.Skin_Sun_Exposed_Lower_leg | *C15orf39* | 1354 | 0.000 | 0.803 | 0.000 | 0.139 | 0.057 | rs117618569 | 0.132 |
| chr15:74,587,571-75,587,571 | All | gtex.Skin_Sun_Exposed_Lower_leg | *RPP25* | 1354 | 0.000 | 0.000 | 0.000 | 1.000 | 0.000 | rs117618569 | 0.160 |
| chr15:74,587,571-75,587,571 | All | gtex.Skin_Sun_Exposed_Lower_leg | *COX5A* | 1354 | 0.000 | 0.643 | 0.000 | 0.311 | 0.045 | rs117429208 | 0.145 |
| chr15:74,587,571-75,587,571 | All | gtex.Skin_Sun_Exposed_Lower_leg | *FAM219B* | 1354 | 0.000 | 0.508 | 0.000 | 0.311 | 0.180 | rs16972628 | 0.362 |
| chr15:74,587,571-75,587,571 | All | gtex.Skin_Sun_Exposed_Lower_leg | *MPI* | 1354 | 0.000 | 0.000 | 0.000 | 1.000 | 0.000 | rs117618569 | 0.131 |
| chr15:74,587,571-75,587,571 | All | gtex.Skin_Sun_Exposed_Lower_leg | *EDC3* | 1354 | 0.000 | 0.835 | 0.000 | 0.105 | 0.060 | rs16972628 | 0.136 |
| chr15:74,587,571-75,587,571 | All | gtex.Skin_Sun_Exposed_Lower_leg | *CLK3* | 1354 | 0.000 | 0.800 | 0.000 | 0.145 | 0.055 | rs117618569 | 0.147 |
| chr15:74,587,571-75,587,571 | All | gtex.Skin_Sun_Exposed_Lower_leg | *ARID3B* | 1354 | 0.000 | 0.575 | 0.000 | 0.390 | 0.035 | rs117618569 | 0.156 |
| chr15:74,587,571-75,587,571 | All | gtex.Skin_Sun_Exposed_Lower_leg | *SCAMP5* | 1354 | 0.000 | 0.000 | 0.000 | 1.000 | 0.000 | rs12437562 | 0.000 |
| chr15:74,587,571-75,587,571 | All | gtex.Skin_Sun_Exposed_Lower_leg | *CPLX3* | 1354 | 0.000 | 0.761 | 0.000 | 0.126 | 0.113 | rs16972486 | 0.296 |
| chr15:74,587,571-75,587,571 | All | gtex.Skin_Sun_Exposed_Lower_leg | *UBL7-DT* | 1354 | 0.000 | 0.857 | 0.000 | 0.091 | 0.052 | rs117618569 | 0.156 |
| chr15:74,587,571-75,587,571 | All | gtex.Skin_Not_Sun_Exposed_Suprapubic | *CSK* | 1354 | 0.000 | 0.828 | 0.000 | 0.112 | 0.060 | rs4488423 | 0.136 |
| chr15:74,587,571-75,587,571 | All | gtex.Skin_Not_Sun_Exposed_Suprapubic | *PPCDC* | 1354 | 0.000 | 0.000 | 0.000 | 1.000 | 0.000 | rs12437562 | 0.000 |
| chr15:74,587,571-75,587,571 | All | gtex.Skin_Not_Sun_Exposed_Suprapubic | *SEMA7A* | 1354 | 0.000 | 0.829 | 0.000 | 0.115 | 0.056 | rs117618569 | 0.152 |
| chr15:74,587,571-75,587,571 | All | gtex.Skin_Not_Sun_Exposed_Suprapubic | *CYP11A1* | 1354 | 0.000 | 0.804 | 0.000 | 0.083 | 0.113 | rs117618569 | 0.333 |
| chr15:74,587,571-75,587,571 | All | gtex.Skin_Not_Sun_Exposed_Suprapubic | *CYP1A1* | 1354 | 0.000 | 0.832 | 0.000 | 0.089 | 0.080 | rs117618569 | 0.234 |
| chr15:74,587,571-75,587,571 | All | gtex.Skin_Not_Sun_Exposed_Suprapubic | *ULK3* | 1354 | 0.000 | 0.069 | 0.000 | 0.915 | 0.016 | rs12437562 | 0.232 |
| chr15:74,587,571-75,587,571 | All | gtex.Skin_Not_Sun_Exposed_Suprapubic | *SCAMP2* | 1354 | 0.000 | 0.272 | 0.000 | 0.703 | 0.025 | rs16972628 | 0.181 |
| chr15:74,587,571-75,587,571 | All | gtex.Skin_Not_Sun_Exposed_Suprapubic | *CYP1A2* | 1354 | 0.000 | 0.863 | 0.000 | 0.088 | 0.049 | rs117618569 | 0.165 |
| chr15:74,587,571-75,587,571 | All | gtex.Skin_Not_Sun_Exposed_Suprapubic | *LMAN1L* | 1354 | 0.000 | 0.763 | 0.000 | 0.188 | 0.050 | rs117618569 | 0.184 |
| chr15:74,587,571-75,587,571 | All | gtex.Skin_Not_Sun_Exposed_Suprapubic | *C15orf39* | 1354 | 0.000 | 0.820 | 0.000 | 0.126 | 0.054 | rs16972628 | 0.218 |
| chr15:74,587,571-75,587,571 | All | gtex.Skin_Not_Sun_Exposed_Suprapubic | *RPP25* | 1354 | 0.000 | 0.257 | 0.000 | 0.721 | 0.022 | rs117618569 | 0.220 |
| chr15:74,587,571-75,587,571 | All | gtex.Skin_Not_Sun_Exposed_Suprapubic | *COX5A* | 1354 | 0.000 | 0.746 | 0.000 | 0.211 | 0.043 | rs117618569 | 0.160 |
| chr15:74,587,571-75,587,571 | All | gtex.Skin_Not_Sun_Exposed_Suprapubic | *FAM219B* | 1354 | 0.000 | 0.727 | 0.000 | 0.202 | 0.071 | rs16972628 | 0.157 |
| chr15:74,587,571-75,587,571 | All | gtex.Skin_Not_Sun_Exposed_Suprapubic | *MPI* | 1354 | 0.000 | 0.063 | 0.000 | 0.931 | 0.006 | rs16972628 | 0.348 |
| chr15:74,587,571-75,587,571 | All | gtex.Skin_Not_Sun_Exposed_Suprapubic | *EDC3* | 1354 | 0.000 | 0.831 | 0.000 | 0.097 | 0.071 | rs117618569 | 0.175 |
| chr15:74,587,571-75,587,571 | All | gtex.Skin_Not_Sun_Exposed_Suprapubic | *CLK3* | 1354 | 0.000 | 0.809 | 0.000 | 0.131 | 0.060 | rs117618569 | 0.199 |
| chr15:74,587,571-75,587,571 | All | gtex.Skin_Not_Sun_Exposed_Suprapubic | *ARID3B* | 1354 | 0.000 | 0.776 | 0.000 | 0.140 | 0.084 | rs16972628 | 0.339 |
| chr15:74,587,571-75,587,571 | All | gtex.Skin_Not_Sun_Exposed_Suprapubic | *SCAMP5* | 1354 | 0.000 | 0.000 | 0.000 | 1.000 | 0.000 | rs117429208 | 0.001 |
| chr15:74,587,571-75,587,571 | All | gtex.Skin_Not_Sun_Exposed_Suprapubic | *CPLX3* | 1354 | 0.000 | 0.639 | 0.000 | 0.140 | 0.221 | rs16972486 | 0.373 |
| chr15:74,587,571-75,587,571 | All | gtex.Skin_Not_Sun_Exposed_Suprapubic | *UBL7-DT* | 1354 | 0.000 | 0.808 | 0.000 | 0.141 | 0.051 | rs117618569 | 0.172 |
| chr12:110,914,461-111,914,461 | All | gtex.Whole_Blood | *VPS29* | 664 | 0.090 | 0.775 | 0.008 | 0.070 | 0.057 | . | . |
| chr12:110,914,461-111,914,461 | All | gtex.Whole_Blood | *MYL2* | 664 | 0.091 | 0.790 | 0.004 | 0.038 | 0.076 | . | . |
| chr12:110,914,461-111,914,461 | All | gtex.Whole_Blood | *SH2B3* | 664 | 0.087 | 0.757 | 0.008 | 0.072 | 0.076 | . | . |
| chr12:110,914,461-111,914,461 | All | gtex.Whole_Blood | *HVCN1* | 664 | 0.089 | 0.772 | 0.007 | 0.061 | 0.071 | . | . |
| chr12:110,914,461-111,914,461 | All | gtex.Whole_Blood | *RAD9B* | 664 | 0.092 | 0.797 | 0.006 | 0.048 | 0.057 | . | . |
| chr12:110,914,461-111,914,461 | All | gtex.Whole_Blood | *PPP1CC* | 664 | 0.090 | 0.783 | 0.005 | 0.040 | 0.082 | . | . |
| chr12:110,914,461-111,914,461 | All | gtex.Whole_Blood | *PPTC7* | 664 | 0.092 | 0.795 | 0.006 | 0.048 | 0.059 | . | . |
| chr12:110,914,461-111,914,461 | All | gtex.Whole_Blood | *PHETA1* | 664 | 0.057 | 0.496 | 0.028 | 0.245 | 0.173 | . | . |
| chr12:110,914,461-111,914,461 | All | gtex.Whole_Blood | *ATXN2* | 640 | 0.090 | 0.778 | 0.007 | 0.056 | 0.070 | . | . |
| chr12:110,914,461-111,914,461 | All | gtex.Whole_Blood | *TCTN1* | 664 | 0.083 | 0.716 | 0.005 | 0.044 | 0.152 | . | . |
| chr12:110,914,461-111,914,461 | All | gtex.Whole_Blood | *FAM216A* | 659 | 0.024 | 0.209 | 0.077 | 0.664 | 0.026 | . | . |
| chr12:110,914,461-111,914,461 | All | gtex.Nerve_Tibial | *VPS29* | 664 | 0.012 | 0.102 | 0.088 | 0.759 | 0.039 | . | . |
| chr12:110,914,461-111,914,461 | All | gtex.Nerve_Tibial | *MYL2* | 664 | 0.094 | 0.811 | 0.005 | 0.042 | 0.049 | . | . |
| chr12:110,914,461-111,914,461 | All | gtex.Nerve_Tibial | *SH2B3* | 664 | 0.092 | 0.798 | 0.005 | 0.043 | 0.062 | . | . |
| chr12:110,914,461-111,914,461 | All | gtex.Nerve_Tibial | *HVCN1* | 664 | 0.087 | 0.754 | 0.007 | 0.060 | 0.091 | . | . |
| chr12:110,914,461-111,914,461 | All | gtex.Nerve_Tibial | *RAD9B* | 664 | 0.092 | 0.798 | 0.005 | 0.046 | 0.058 | . | . |
| chr12:110,914,461-111,914,461 | All | gtex.Nerve_Tibial | *PPP1CC* | 664 | 0.087 | 0.756 | 0.008 | 0.066 | 0.083 | . | . |
| chr12:110,914,461-111,914,461 | All | gtex.Nerve_Tibial | *PPTC7* | 664 | 0.091 | 0.786 | 0.006 | 0.051 | 0.067 | . | . |
| chr12:110,914,461-111,914,461 | All | gtex.Nerve_Tibial | *PHETA1* | 664 | 0.002 | 0.016 | 0.101 | 0.877 | 0.004 | . | . |
| chr12:110,914,461-111,914,461 | All | gtex.Nerve_Tibial | *ATXN2* | 640 | 0.094 | 0.810 | 0.004 | 0.038 | 0.054 | . | . |
| chr12:110,914,461-111,914,461 | All | gtex.Nerve_Tibial | *TCTN1* | 664 | 0.083 | 0.714 | 0.013 | 0.109 | 0.082 | . | . |
| chr12:110,914,461-111,914,461 | All | gtex.Nerve_Tibial | *FAM216A* | 659 | 0.079 | 0.687 | 0.011 | 0.092 | 0.131 | . | . |
| chr12:110,914,461-111,914,461 | All | gtex.Skin_Sun_Exposed_Lower_leg | *VPS29* | 664 | 0.000 | 0.000 | 0.102 | 0.887 | 0.011 | . | . |
| chr12:110,914,461-111,914,461 | All | gtex.Skin_Sun_Exposed_Lower_leg | *MYL2* | 664 | 0.090 | 0.779 | 0.007 | 0.056 | 0.068 | . | . |
| chr12:110,914,461-111,914,461 | All | gtex.Skin_Sun_Exposed_Lower_leg | *CUX2* | 664 | 0.091 | 0.786 | 0.005 | 0.045 | 0.073 | . | . |
| chr12:110,914,461-111,914,461 | All | gtex.Skin_Sun_Exposed_Lower_leg | *SH2B3* | 664 | 0.092 | 0.795 | 0.006 | 0.052 | 0.056 | . | . |
| chr12:110,914,461-111,914,461 | All | gtex.Skin_Sun_Exposed_Lower_leg | *HVCN1* | 664 | 0.091 | 0.788 | 0.006 | 0.050 | 0.066 | . | . |
| chr12:110,914,461-111,914,461 | All | gtex.Skin_Sun_Exposed_Lower_leg | *RAD9B* | 664 | 0.093 | 0.805 | 0.005 | 0.043 | 0.055 | . | . |
| chr12:110,914,461-111,914,461 | All | gtex.Skin_Sun_Exposed_Lower_leg | *CCDC63* | 664 | 0.046 | 0.394 | 0.043 | 0.372 | 0.145 | . | . |
| chr12:110,914,461-111,914,461 | All | gtex.Skin_Sun_Exposed_Lower_leg | *PPP1CC* | 664 | 0.087 | 0.754 | 0.009 | 0.074 | 0.076 | . | . |
| chr12:110,914,461-111,914,461 | All | gtex.Skin_Sun_Exposed_Lower_leg | *PPTC7* | 664 | 0.087 | 0.753 | 0.008 | 0.068 | 0.084 | . | . |
| chr12:110,914,461-111,914,461 | All | gtex.Skin_Sun_Exposed_Lower_leg | *PHETA1* | 664 | 0.091 | 0.790 | 0.007 | 0.059 | 0.053 | . | . |
| chr12:110,914,461-111,914,461 | All | gtex.Skin_Sun_Exposed_Lower_leg | *ATXN2* | 640 | 0.093 | 0.806 | 0.005 | 0.041 | 0.055 | . | . |
| chr12:110,914,461-111,914,461 | All | gtex.Skin_Sun_Exposed_Lower_leg | *TCTN1* | 664 | 0.092 | 0.795 | 0.005 | 0.046 | 0.061 | . | . |
| chr12:110,914,461-111,914,461 | All | gtex.Skin_Sun_Exposed_Lower_leg | *FAM216A* | 659 | 0.075 | 0.646 | 0.014 | 0.120 | 0.145 | . | . |
| chr12:110,914,461-111,914,461 | All | gtex.Skin_Not_Sun_Exposed_Suprapubic | *VPS29* | 664 | 0.056 | 0.488 | 0.038 | 0.326 | 0.091 | . | . |
| chr12:110,914,461-111,914,461 | All | gtex.Skin_Not_Sun_Exposed_Suprapubic | *MYL2* | 664 | 0.089 | 0.774 | 0.008 | 0.065 | 0.064 | . | . |
| chr12:110,914,461-111,914,461 | All | gtex.Skin_Not_Sun_Exposed_Suprapubic | *CUX2* | 664 | 0.090 | 0.777 | 0.008 | 0.066 | 0.060 | . | . |
| chr12:110,914,461-111,914,461 | All | gtex.Skin_Not_Sun_Exposed_Suprapubic | *SH2B3* | 664 | 0.091 | 0.787 | 0.006 | 0.048 | 0.069 | . | . |
| chr12:110,914,461-111,914,461 | All | gtex.Skin_Not_Sun_Exposed_Suprapubic | *HVCN1* | 664 | 0.090 | 0.775 | 0.007 | 0.057 | 0.072 | . | . |
| chr12:110,914,461-111,914,461 | All | gtex.Skin_Not_Sun_Exposed_Suprapubic | *RAD9B* | 664 | 0.087 | 0.757 | 0.008 | 0.069 | 0.079 | . | . |
| chr12:110,914,461-111,914,461 | All | gtex.Skin_Not_Sun_Exposed_Suprapubic | *CCDC63* | 664 | 0.091 | 0.787 | 0.006 | 0.055 | 0.061 | . | . |
| chr12:110,914,461-111,914,461 | All | gtex.Skin_Not_Sun_Exposed_Suprapubic | *PPP1CC* | 664 | 0.090 | 0.777 | 0.006 | 0.054 | 0.073 | . | . |
| chr12:110,914,461-111,914,461 | All | gtex.Skin_Not_Sun_Exposed_Suprapubic | *PPTC7* | 664 | 0.086 | 0.740 | 0.006 | 0.052 | 0.116 | . | . |
| chr12:110,914,461-111,914,461 | All | gtex.Skin_Not_Sun_Exposed_Suprapubic | *PHETA1* | 664 | 0.084 | 0.724 | 0.010 | 0.087 | 0.096 | . | . |
| chr12:110,914,461-111,914,461 | All | gtex.Skin_Not_Sun_Exposed_Suprapubic | *ATXN2* | 640 | 0.089 | 0.771 | 0.008 | 0.073 | 0.058 | . | . |
| chr12:110,914,461-111,914,461 | All | gtex.Skin_Not_Sun_Exposed_Suprapubic | *TCTN1* | 664 | 0.091 | 0.791 | 0.006 | 0.052 | 0.059 | . | . |
| chr12:110,914,461-111,914,461 | All | gtex.Skin_Not_Sun_Exposed_Suprapubic | *FAM216A* | 659 | 0.084 | 0.726 | 0.013 | 0.113 | 0.063 | . | . |
| chr16:10,960,480-11,960,480 | All | gtex.Whole_Blood | *CLEC16A* | 2544 | 0.000 | 0.737 | 0.000 | 0.222 | 0.041 | rs4781072 | 0.716 |
| chr16:10,960,480-11,960,480 | All | gtex.Whole_Blood | *ZC3H7A* | 2544 | 0.000 | 0.133 | 0.000 | 0.861 | 0.007 | rs4781072 | 0.661 |
| chr16:10,960,480-11,960,480 | All | gtex.Whole_Blood | *PRM2* | 2544 | 0.000 | 0.726 | 0.000 | 0.179 | 0.095 | rs4781072 | 0.774 |
| chr16:10,960,480-11,960,480 | All | gtex.Whole_Blood | *TXNDC11* | 2544 | 0.000 | 0.652 | 0.000 | 0.311 | 0.037 | rs4781072 | 0.674 |
| chr16:10,960,480-11,960,480 | All | gtex.Whole_Blood | *RSL1D1* | 2544 | 0.000 | 0.659 | 0.000 | 0.305 | 0.036 | rs4781072 | 0.624 |
| chr16:10,960,480-11,960,480 | All | gtex.Whole_Blood | *RMI2* | 2544 | 0.000 | 0.486 | 0.000 | 0.181 | 0.333 | rs4781072 | 0.720 |
| chr16:10,960,480-11,960,480 | All | gtex.Whole_Blood | *PRM1* | 2544 | 0.000 | 0.743 | 0.000 | 0.193 | 0.064 | rs4781072 | 0.711 |
| chr16:10,960,480-11,960,480 | All | gtex.Whole_Blood | *CIITA* | 2544 | 0.000 | 0.788 | 0.000 | 0.162 | 0.050 | rs4781072 | 0.648 |
| chr16:10,960,480-11,960,480 | All | gtex.Whole_Blood | *DEXI* | 2544 | 0.000 | 0.798 | 0.000 | 0.163 | 0.039 | rs4781072 | 0.681 |
| chr16:10,960,480-11,960,480 | All | gtex.Whole_Blood | *SNN* | 2544 | 0.000 | 0.687 | 0.000 | 0.261 | 0.051 | rs4781072 | 0.648 |
| chr16:10,960,480-11,960,480 | All | gtex.Whole_Blood | *SOCS1* | 2544 | 0.000 | 0.730 | 0.000 | 0.167 | 0.102 | rs4781072 | 0.705 |
| chr16:10,960,480-11,960,480 | All | gtex.Whole_Blood | *LITAF* | 2544 | 0.000 | 0.677 | 0.000 | 0.242 | 0.081 | rs4781072 | 0.646 |
| chr16:10,960,480-11,960,480 | All | gtex.Whole_Blood | *LOC101927131* | 2544 | 0.000 | 0.738 | 0.000 | 0.222 | 0.039 | rs4781072 | 0.656 |
| chr16:10,960,480-11,960,480 | All | gtex.Nerve_Tibial | *CLEC16A* | 2544 | 0.000 | 0.213 | 0.000 | 0.776 | 0.011 | rs4781072 | 0.688 |
| chr16:10,960,480-11,960,480 | All | gtex.Nerve_Tibial | *ZC3H7A* | 2544 | 0.000 | 0.690 | 0.000 | 0.230 | 0.079 | rs4781072 | 0.707 |
| chr16:10,960,480-11,960,480 | All | gtex.Nerve_Tibial | *PRM2* | 2544 | 0.000 | 0.789 | 0.000 | 0.163 | 0.048 | rs4781072 | 0.660 |
| chr16:10,960,480-11,960,480 | All | gtex.Nerve_Tibial | *TXNDC11* | 2544 | 0.000 | 0.570 | 0.000 | 0.366 | 0.064 | rs4781072 | 0.789 |
| chr16:10,960,480-11,960,480 | All | gtex.Nerve_Tibial | *RSL1D1* | 2544 | 0.000 | 0.766 | 0.000 | 0.195 | 0.039 | rs4781072 | 0.665 |
| chr16:10,960,480-11,960,480 | All | gtex.Nerve_Tibial | *RMI2* | 2544 | 0.000 | 0.000 | 0.000 | 1.000 | 0.000 | rs4781072 | 0.000 |
| chr16:10,960,480-11,960,480 | All | gtex.Nerve_Tibial | *PRM1* | 2544 | 0.000 | 0.773 | 0.000 | 0.167 | 0.060 | rs4781072 | 0.612 |
| chr16:10,960,480-11,960,480 | All | gtex.Nerve_Tibial | *CIITA* | 2544 | 0.000 | 0.659 | 0.000 | 0.300 | 0.041 | rs4781072 | 0.740 |
| chr16:10,960,480-11,960,480 | All | gtex.Nerve_Tibial | *DEXI* | 2544 | 0.000 | 0.705 | 0.000 | 0.231 | 0.064 | rs4781072 | 0.757 |
| chr16:10,960,480-11,960,480 | All | gtex.Nerve_Tibial | *SNN* | 2544 | 0.000 | 0.081 | 0.000 | 0.915 | 0.004 | rs4781072 | 0.707 |
| chr16:10,960,480-11,960,480 | All | gtex.Nerve_Tibial | *SOCS1* | 2544 | 0.000 | 0.702 | 0.000 | 0.198 | 0.100 | **rs4781072** | 0.803 |
| chr16:10,960,480-11,960,480 | All | gtex.Nerve_Tibial | *LITAF* | 2544 | 0.000 | 0.756 | 0.000 | 0.207 | 0.037 | rs4781072 | 0.679 |
| chr16:10,960,480-11,960,480 | All | gtex.Skin_Sun_Exposed_Lower_leg | *CLEC16A* | 2544 | 0.000 | 0.580 | 0.000 | 0.169 | 0.251 | rs4781072 | 0.720 |
| chr16:10,960,480-11,960,480 | All | gtex.Skin_Sun_Exposed_Lower_leg | *ZC3H7A* | 2544 | 0.000 | 0.743 | 0.000 | 0.184 | 0.073 | rs4781072 | 0.522 |
| chr16:10,960,480-11,960,480 | All | gtex.Skin_Sun_Exposed_Lower_leg | *PRM2* | 2544 | 0.000 | 0.734 | 0.000 | 0.174 | 0.092 | rs4781072 | 0.708 |
| chr16:10,960,480-11,960,480 | All | gtex.Skin_Sun_Exposed_Lower_leg | *TXNDC11* | 2544 | 0.000 | 0.663 | 0.000 | 0.301 | 0.035 | rs4781072 | 0.652 |
| chr16:10,960,480-11,960,480 | All | gtex.Skin_Sun_Exposed_Lower_leg | *RSL1D1* | 2544 | 0.000 | 0.006 | 0.000 | 0.994 | 0.000 | rs4781072 | 0.693 |
| chr16:10,960,480-11,960,480 | All | gtex.Skin_Sun_Exposed_Lower_leg | *RMI2* | 2544 | 0.000 | 0.000 | 0.000 | 1.000 | 0.000 | rs4781072 | 0.000 |
| chr16:10,960,480-11,960,480 | All | gtex.Skin_Sun_Exposed_Lower_leg | *PRM1* | 2544 | 0.000 | 0.732 | 0.000 | 0.177 | 0.091 | rs4781072 | 0.753 |
| chr16:10,960,480-11,960,480 | All | gtex.Skin_Sun_Exposed_Lower_leg | *CIITA* | 2544 | 0.000 | 0.720 | 0.000 | 0.239 | 0.041 | rs4781072 | 0.616 |
| chr16:10,960,480-11,960,480 | All | gtex.Skin_Sun_Exposed_Lower_leg | *DEXI* | 2544 | 0.000 | 0.739 | 0.000 | 0.225 | 0.037 | rs4781072 | 0.679 |
| chr16:10,960,480-11,960,480 | All | gtex.Skin_Sun_Exposed_Lower_leg | *SNN* | 2544 | 0.000 | 0.071 | 0.000 | 0.833 | 0.097 | **rs4781072** | 0.915 |
| chr16:10,960,480-11,960,480 | All | gtex.Skin_Sun_Exposed_Lower_leg | *SOCS1* | 2544 | 0.000 | 0.622 | 0.000 | 0.338 | 0.040 | rs4781072 | 0.718 |
| chr16:10,960,480-11,960,480 | All | gtex.Skin_Sun_Exposed_Lower_leg | *LITAF* | 2544 | 0.000 | 0.012 | 0.000 | 0.988 | 0.001 | rs4781072 | 0.670 |
| chr16:10,960,480-11,960,480 | All | gtex.Skin_Not_Sun_Exposed_Suprapubic | *CLEC16A* | 2544 | 0.000 | 0.341 | 0.000 | 0.566 | 0.093 | rs4781072 | 0.602 |
| chr16:10,960,480-11,960,480 | All | gtex.Skin_Not_Sun_Exposed_Suprapubic | *ZC3H7A* | 2544 | 0.000 | 0.772 | 0.000 | 0.182 | 0.046 | rs4781072 | 0.737 |
| chr16:10,960,480-11,960,480 | All | gtex.Skin_Not_Sun_Exposed_Suprapubic | *PRM2* | 2544 | 0.000 | 0.724 | 0.000 | 0.206 | 0.069 | rs4781072 | 0.643 |
| chr16:10,960,480-11,960,480 | All | gtex.Skin_Not_Sun_Exposed_Suprapubic | *TXNDC11* | 2544 | 0.000 | 0.681 | 0.000 | 0.285 | 0.034 | rs4781072 | 0.678 |
| chr16:10,960,480-11,960,480 | All | gtex.Skin_Not_Sun_Exposed_Suprapubic | *RSL1D1* | 2544 | 0.000 | 0.720 | 0.000 | 0.236 | 0.044 | rs4781072 | 0.735 |
| chr16:10,960,480-11,960,480 | All | gtex.Skin_Not_Sun_Exposed_Suprapubic | *RMI2* | 2544 | 0.000 | 0.000 | 0.000 | 1.000 | 0.000 | rs4781072 | 0.000 |
| chr16:10,960,480-11,960,480 | All | gtex.Skin_Not_Sun_Exposed_Suprapubic | *PRM1* | 2544 | 0.000 | 0.673 | 0.000 | 0.248 | 0.079 | rs4781072 | 0.687 |
| chr16:10,960,480-11,960,480 | All | gtex.Skin_Not_Sun_Exposed_Suprapubic | *CIITA* | 2544 | 0.000 | 0.748 | 0.000 | 0.187 | 0.065 | rs4781072 | 0.732 |
| chr16:10,960,480-11,960,480 | All | gtex.Skin_Not_Sun_Exposed_Suprapubic | *DEXI* | 2544 | 0.000 | 0.760 | 0.000 | 0.191 | 0.050 | rs4781072 | 0.716 |
| chr16:10,960,480-11,960,480 | All | gtex.Skin_Not_Sun_Exposed_Suprapubic | *SNN* | 2544 | 0.000 | 0.020 | 0.000 | 0.979 | 0.002 | rs4781072 | 0.663 |
| chr16:10,960,480-11,960,480 | All | gtex.Skin_Not_Sun_Exposed_Suprapubic | *SOCS1* | 2544 | 0.000 | 0.717 | 0.000 | 0.205 | 0.078 | rs4781072 | 0.719 |
| chr16:10,960,480-11,960,480 | All | gtex.Skin_Not_Sun_Exposed_Suprapubic | *LITAF* | 2544 | 0.000 | 0.689 | 0.000 | 0.263 | 0.047 | rs4781072 | 0.690 |
| chr10:63,991,638-64,994,157 | First | gtex.Whole_Blood | *EGR2* | 1771 | 0.000 | 0.822 | 0.000 | 0.116 | 0.062 | rs10822054 | 0.099 |
| chr10:63,991,638-64,994,157 | First | gtex.Whole_Blood | *ZNF365* | 1771 | 0.000 | 0.753 | 0.000 | 0.164 | 0.083 | rs7084016 | 0.084 |
| chr10:63,991,638-64,994,157 | First | gtex.Whole_Blood | *NRBF2* | 1771 | 0.000 | 0.697 | 0.000 | 0.125 | 0.177 | rs10822054 | 0.144 |
| chr10:63,991,638-64,994,157 | First | gtex.Whole_Blood | *JMJD1C* | 1668 | 0.000 | 0.821 | 0.000 | 0.119 | 0.060 | rs7084016 | 0.094 |
| chr10:63,991,638-64,994,157 | First | gtex.Whole_Blood | *ADO* | 1771 | 0.000 | 0.131 | 0.000 | 0.700 | 0.169 | rs10822054 | 0.135 |
| chr10:63,991,638-64,994,157 | First | gtex.Whole_Blood | *RTKN2* | 1771 | 0.000 | 0.817 | 0.000 | 0.126 | 0.057 | rs7084016 | 0.091 |
| chr10:63,991,638-64,994,157 | First | gtex.Nerve_Tibial | *EGR2* | 1771 | 0.000 | 0.781 | 0.000 | 0.144 | 0.075 | rs10995311 | 0.098 |
| chr10:63,991,638-64,994,157 | First | gtex.Nerve_Tibial | *ZNF365* | 1771 | 0.000 | 0.774 | 0.000 | 0.162 | 0.065 | rs7084016 | 0.092 |
| chr10:63,991,638-64,994,157 | First | gtex.Nerve_Tibial | *NRBF2* | 1771 | 0.000 | 0.816 | 0.000 | 0.127 | 0.057 | rs7084016 | 0.093 |
| chr10:63,991,638-64,994,157 | First | gtex.Nerve_Tibial | *JMJD1C* | 1668 | 0.000 | 0.792 | 0.000 | 0.147 | 0.061 | rs7084016 | 0.094 |
| chr10:63,991,638-64,994,157 | First | gtex.Nerve_Tibial | *ADO* | 1771 | 0.000 | 0.768 | 0.000 | 0.175 | 0.057 | rs10822054 | 0.099 |
| chr10:63,991,638-64,994,157 | First | gtex.Nerve_Tibial | *RTKN2* | 1771 | 0.000 | 0.639 | 0.000 | 0.128 | 0.233 | rs10822054 | 0.125 |
| chr10:63,991,638-64,994,157 | First | gtex.Skin_Sun_Exposed_Lower_leg | *EGR2* | 1771 | 0.000 | 0.285 | 0.000 | 0.684 | 0.030 | rs7084016 | 0.099 |
| chr10:63,991,638-64,994,157 | First | gtex.Skin_Sun_Exposed_Lower_leg | *ZNF365* | 1771 | 0.000 | 0.000 | 0.000 | 1.000 | 0.000 | rs7084016 | 0.000 |
| chr10:63,991,638-64,994,157 | First | gtex.Skin_Sun_Exposed_Lower_leg | *NRBF2* | 1771 | 0.000 | 0.588 | 0.000 | 0.352 | 0.061 | rs10995311 | 0.127 |
| chr10:63,991,638-64,994,157 | First | gtex.Skin_Sun_Exposed_Lower_leg | *JMJD1C* | 1668 | 0.000 | 0.793 | 0.000 | 0.153 | 0.054 | rs7084016 | 0.092 |
| chr10:63,991,638-64,994,157 | First | gtex.Skin_Sun_Exposed_Lower_leg | *ADO* | 1771 | 0.000 | 0.608 | 0.000 | 0.324 | 0.067 | rs10995282 | 0.098 |
| chr10:63,991,638-64,994,157 | First | gtex.Skin_Sun_Exposed_Lower_leg | *RTKN2* | 1771 | 0.000 | 0.000 | 0.000 | 1.000 | 0.000 | rs10995282 | 0.092 |
| chr10:63,991,638-64,994,157 | First | gtex.Skin_Not_Sun_Exposed_Suprapubic | *EGR2* | 1771 | 0.000 | 0.805 | 0.000 | 0.140 | 0.055 | rs7084016 | 0.091 |
| chr10:63,991,638-64,994,157 | First | gtex.Skin_Not_Sun_Exposed_Suprapubic | *ZNF365* | 1771 | 0.000 | 0.000 | 0.000 | 1.000 | 0.000 | rs10822054 | 0.000 |
| chr10:63,991,638-64,994,157 | First | gtex.Skin_Not_Sun_Exposed_Suprapubic | *NRBF2* | 1771 | 0.000 | 0.586 | 0.000 | 0.358 | 0.056 | rs7084016 | 0.104 |
| chr10:63,991,638-64,994,157 | First | gtex.Skin_Not_Sun_Exposed_Suprapubic | *JMJD1C* | 1668 | 0.000 | 0.823 | 0.000 | 0.122 | 0.055 | rs7084016 | 0.093 |
| chr10:63,991,638-64,994,157 | First | gtex.Skin_Not_Sun_Exposed_Suprapubic | *ADO* | 1771 | 0.000 | 0.738 | 0.000 | 0.212 | 0.050 | rs10822054 | 0.092 |
| chr10:63,991,638-64,994,157 | First | gtex.Skin_Not_Sun_Exposed_Suprapubic | *RTKN2* | 1771 | 0.000 | 0.000 | 0.000 | 1.000 | 0.000 | rs10822054 | 0.102 |
| chr10:63,991,638-64,994,157 | Second | gtex.Whole_Blood | *EGR2* | 1786 | 0.000 | 0.827 | 0.000 | 0.118 | 0.055 | rs57565226 | 0.069 |
| chr10:63,991,638-64,994,157 | Second | gtex.Whole_Blood | *ZNF365* | 1786 | 0.000 | 0.776 | 0.000 | 0.171 | 0.053 | rs58600253 | 0.055 |
| chr10:63,991,638-64,994,157 | Second | gtex.Whole_Blood | *NRBF2* | 1786 | 0.000 | 0.787 | 0.000 | 0.139 | 0.073 | rs58600253 | 0.074 |
| chr10:63,991,638-64,994,157 | Second | gtex.Whole_Blood | *JMJD1C* | 1665 | 0.000 | 0.827 | 0.000 | 0.119 | 0.054 | rs57565226 | 0.055 |
| chr10:63,991,638-64,994,157 | Second | gtex.Whole_Blood | *ADO* | 1786 | 0.000 | 0.163 | 0.000 | 0.826 | 0.011 | rs57565226 | 0.059 |
| chr10:63,991,638-64,994,157 | Second | gtex.Whole_Blood | *RTKN2* | 1786 | 0.000 | 0.814 | 0.000 | 0.129 | 0.057 | rs58600253 | 0.053 |
| chr10:63,991,638-64,994,157 | Second | gtex.Nerve_Tibial | *EGR2* | 1786 | 0.000 | 0.778 | 0.000 | 0.152 | 0.070 | rs79754022 | 0.056 |
| chr10:63,991,638-64,994,157 | Second | gtex.Nerve_Tibial | *ZNF365* | 1786 | 0.000 | 0.724 | 0.000 | 0.162 | 0.114 | rs79754022 | 0.074 |
| chr10:63,991,638-64,994,157 | Second | gtex.Nerve_Tibial | *NRBF2* | 1786 | 0.000 | 0.815 | 0.000 | 0.130 | 0.055 | rs58600253 | 0.058 |
| chr10:63,991,638-64,994,157 | Second | gtex.Nerve_Tibial | *JMJD1C* | 1665 | 0.000 | 0.796 | 0.000 | 0.153 | 0.051 | rs57565226 | 0.057 |
| chr10:63,991,638-64,994,157 | Second | gtex.Nerve_Tibial | *ADO* | 1786 | 0.000 | 0.683 | 0.000 | 0.157 | 0.160 | rs113168114 | 0.075 |
| chr10:63,991,638-64,994,157 | Second | gtex.Nerve_Tibial | *RTKN2* | 1786 | 0.000 | 0.785 | 0.000 | 0.146 | 0.068 | rs57565226 | 0.073 |
| chr10:63,991,638-64,994,157 | Second | gtex.Skin_Sun_Exposed_Lower_leg | *EGR2* | 1786 | 0.000 | 0.262 | 0.000 | 0.636 | 0.102 | rs11818600 | 0.524 |
| chr10:63,991,638-64,994,157 | Second | gtex.Skin_Sun_Exposed_Lower_leg | *ZNF365* | 1786 | 0.000 | 0.000 | 0.000 | 1.000 | 0.000 | rs57352524 | 0.000 |
| chr10:63,991,638-64,994,157 | Second | gtex.Skin_Sun_Exposed_Lower_leg | *NRBF2* | 1786 | 0.000 | 0.559 | 0.000 | 0.358 | 0.082 | rs57565226 | 0.063 |
| chr10:63,991,638-64,994,157 | Second | gtex.Skin_Sun_Exposed_Lower_leg | *JMJD1C* | 1665 | 0.000 | 0.790 | 0.000 | 0.156 | 0.054 | rs57565226 | 0.073 |
| chr10:63,991,638-64,994,157 | Second | gtex.Skin_Sun_Exposed_Lower_leg | *ADO* | 1786 | 0.000 | 0.357 | 0.000 | 0.184 | 0.459 | rs76510618 | 0.007 |
| chr10:63,991,638-64,994,157 | Second | gtex.Skin_Sun_Exposed_Lower_leg | *RTKN2* | 1786 | 0.000 | 0.000 | 0.000 | 0.999 | 0.000 | rs58600253 | 0.050 |
| chr10:63,991,638-64,994,157 | Second | gtex.Skin_Not_Sun_Exposed_Suprapubic | *EGR2* | 1786 | 0.000 | 0.799 | 0.000 | 0.140 | 0.060 | rs57565226 | 0.065 |
| chr10:63,991,638-64,994,157 | Second | gtex.Skin_Not_Sun_Exposed_Suprapubic | *ZNF365* | 1786 | 0.000 | 0.000 | 0.000 | 1.000 | 0.000 | rs58600253 | 0.000 |
| chr10:63,991,638-64,994,157 | Second | gtex.Skin_Not_Sun_Exposed_Suprapubic | *NRBF2* | 1786 | 0.000 | 0.565 | 0.000 | 0.370 | 0.065 | rs79754022 | 0.068 |
| chr10:63,991,638-64,994,157 | Second | gtex.Skin_Not_Sun_Exposed_Suprapubic | *JMJD1C* | 1665 | 0.000 | 0.824 | 0.000 | 0.123 | 0.052 | rs58600253 | 0.057 |
| chr10:63,991,638-64,994,157 | Second | gtex.Skin_Not_Sun_Exposed_Suprapubic | *ADO* | 1786 | 0.000 | 0.717 | 0.000 | 0.203 | 0.080 | rs57565226 | 0.049 |
| chr10:63,991,638-64,994,157 | Second | gtex.Skin_Not_Sun_Exposed_Suprapubic | *RTKN2* | 1786 | 0.000 | 0.000 | 0.000 | 1.000 | 0.000 | rs58600253 | 0.022 |
| chr1:151,289,647-152,289,647 | All | gtex.Whole_Blood | *FLG* | 1223 | 0.011 | 0.766 | 0.002 | 0.126 | 0.095 | rs12144914 | 0.345 |
| chr1:151,289,647-152,289,647 | All | gtex.Nerve_Tibial | *FLG* | 1223 | 0.000 | 0.000 | 0.014 | 0.986 | 0.000 | rs949969 | 0.000 |
| chr1:151,289,647-152,289,647 | All | gtex.Skin_Sun_Exposed_Lower_leg | *FLG* | 1223 | 0.000 | 0.000 | 0.014 | 0.985 | 0.000 | rs61815119 | 0.074 |
| chr1:151,289,647-152,289,647 | All | gtex.Skin_Not_Sun_Exposed_Suprapubic | *FLG* | 1223 | 0.007 | 0.461 | 0.007 | 0.493 | 0.033 | rs12144914 | 0.360 |
| chr5:158,327,769-159,327,769 | All | gtex.Whole_Blood | *RNF145* | 1702 | 0.000 | 0.837 | 0.000 | 0.117 | 0.046 | rs56167332 | 0.391 |
| chr5:158,327,769-159,327,769 | All | gtex.Whole_Blood | *EBF1* | 1702 | 0.000 | 0.830 | 0.000 | 0.107 | 0.063 | rs56167332 | 0.406 |
| chr5:158,327,769-159,327,769 | All | gtex.Whole_Blood | *UBLCP1* | 1702 | 0.000 | 0.563 | 0.000 | 0.404 | 0.032 | rs56167332 | 0.437 |
| chr5:158,327,769-159,327,769 | All | gtex.Whole_Blood | *LINC02202* | 1702 | 0.000 | 0.739 | 0.000 | 0.137 | 0.124 | rs56167332 | 0.392 |
| chr5:158,327,769-159,327,769 | All | gtex.Nerve_Tibial | *RNF145* | 1702 | 0.000 | 0.804 | 0.000 | 0.153 | 0.043 | rs56167332 | 0.397 |
| chr5:158,327,769-159,327,769 | All | gtex.Nerve_Tibial | *EBF1* | 1702 | 0.000 | 0.840 | 0.000 | 0.116 | 0.044 | rs56167332 | 0.412 |
| chr5:158,327,769-159,327,769 | All | gtex.Nerve_Tibial | *UBLCP1* | 1702 | 0.000 | 0.309 | 0.000 | 0.669 | 0.022 | rs56167332 | 0.385 |
| chr5:158,327,769-159,327,769 | All | gtex.Nerve_Tibial | *LINC02202* | 1702 | 0.000 | 0.028 | 0.000 | 0.969 | 0.002 | rs56167332 | 0.417 |
| chr5:158,327,769-159,327,769 | All | gtex.Skin_Sun_Exposed_Lower_leg | *RNF145* | 1702 | 0.000 | 0.802 | 0.000 | 0.137 | 0.062 | rs56167332 | 0.433 |
| chr5:158,327,769-159,327,769 | All | gtex.Skin_Sun_Exposed_Lower_leg | *EBF1* | 1702 | 0.000 | 0.826 | 0.000 | 0.124 | 0.049 | rs56167332 | 0.404 |
| chr5:158,327,769-159,327,769 | All | gtex.Skin_Sun_Exposed_Lower_leg | *UBLCP1* | 1702 | 0.000 | 0.761 | 0.000 | 0.196 | 0.043 | rs56167332 | 0.417 |
| chr5:158,327,769-159,327,769 | All | gtex.Skin_Sun_Exposed_Lower_leg | *LINC02202* | 1702 | 0.000 | 0.359 | 0.000 | 0.621 | 0.020 | rs56167332 | 0.399 |
| chr5:158,327,769-159,327,769 | All | gtex.Skin_Not_Sun_Exposed_Suprapubic | *RNF145* | 1702 | 0.000 | 0.763 | 0.000 | 0.108 | 0.129 | rs755374 | 0.424 |
| chr5:158,327,769-159,327,769 | All | gtex.Skin_Not_Sun_Exposed_Suprapubic | *EBF1* | 1702 | 0.000 | 0.804 | 0.000 | 0.154 | 0.042 | rs56167332 | 0.415 |
| chr5:158,327,769-159,327,769 | All | gtex.Skin_Not_Sun_Exposed_Suprapubic | *UBLCP1* | 1702 | 0.000 | 0.782 | 0.000 | 0.175 | 0.043 | rs56167332 | 0.401 |
| chr5:158,327,769-159,327,769 | All | gtex.Skin_Not_Sun_Exposed_Suprapubic | *LINC02202* | 1702 | 0.000 | 0.807 | 0.000 | 0.145 | 0.047 | rs56167332 | 0.431 |
| chr2:102,591,540-103,591,540 | All | gtex.Whole_Blood | *IL1R2* | 2156 | 0.000 | 0.820 | 0.000 | 0.136 | 0.045 | rs17027258 | 0.085 |
| chr2:102,591,540-103,591,540 | All | gtex.Whole_Blood | *IL1R1* | 2156 | 0.000 | 0.722 | 0.000 | 0.137 | 0.141 | rs17027258 | 0.150 |
| chr2:102,591,540-103,591,540 | All | gtex.Whole_Blood | *IL1RL2* | 2156 | 0.000 | 0.793 | 0.000 | 0.153 | 0.054 | rs17027258 | 0.087 |
| chr2:102,591,540-103,591,540 | All | gtex.Whole_Blood | *IL1RL1* | 2156 | 0.000 | 0.787 | 0.000 | 0.164 | 0.048 | rs17027258 | 0.108 |
| chr2:102,591,540-103,591,540 | All | gtex.Whole_Blood | *IL18R1* | 2156 | 0.000 | 0.240 | 0.000 | 0.743 | 0.017 | rs17027258 | 0.095 |
| chr2:102,591,540-103,591,540 | All | gtex.Whole_Blood | *IL18RAP* | 2156 | 0.000 | 0.000 | 0.000 | 1.000 | 0.000 | rs887971 | 0.000 |
| chr2:102,591,540-103,591,540 | All | gtex.Whole_Blood | *MFSD9* | 2156 | 0.000 | 0.000 | 0.000 | 1.000 | 0.000 | rs17027258 | 0.000 |
| chr2:102,591,540-103,591,540 | All | gtex.Whole_Blood | *TMEM182* | 2156 | 0.000 | 0.767 | 0.000 | 0.188 | 0.045 | rs17027258 | 0.084 |
| chr2:102,591,540-103,591,540 | All | gtex.Whole_Blood | *SLC9A4* | 2156 | 0.000 | 0.792 | 0.000 | 0.141 | 0.067 | rs17027258 | 0.115 |
| chr2:102,591,540-103,591,540 | All | gtex.Nerve_Tibial | *IL1R2* | 2156 | 0.000 | 0.717 | 0.000 | 0.244 | 0.039 | rs17027258 | 0.079 |
| chr2:102,591,540-103,591,540 | All | gtex.Nerve_Tibial | *IL1R1* | 2156 | 0.000 | 0.759 | 0.000 | 0.187 | 0.054 | rs17027258 | 0.078 |
| chr2:102,591,540-103,591,540 | All | gtex.Nerve_Tibial | *IL1RL2* | 2156 | 0.000 | 0.782 | 0.000 | 0.174 | 0.043 | rs17027258 | 0.094 |
| chr2:102,591,540-103,591,540 | All | gtex.Nerve_Tibial | *IL1RL1* | 2156 | 0.000 | 0.773 | 0.000 | 0.145 | 0.082 | rs17027258 | 0.085 |
| chr2:102,591,540-103,591,540 | All | gtex.Nerve_Tibial | *IL18R1* | 2156 | 0.000 | 0.002 | 0.000 | 0.970 | 0.028 | rs887971 | 0.280 |
| chr2:102,591,540-103,591,540 | All | gtex.Nerve_Tibial | *IL18RAP* | 2156 | 0.000 | 0.690 | 0.000 | 0.233 | 0.077 | rs17027258 | 0.072 |
| chr2:102,591,540-103,591,540 | All | gtex.Nerve_Tibial | *MFSD9* | 2156 | 0.000 | 0.721 | 0.000 | 0.232 | 0.047 | rs17027258 | 0.084 |
| chr2:102,591,540-103,591,540 | All | gtex.Nerve_Tibial | *TMEM182* | 2156 | 0.000 | 0.785 | 0.000 | 0.157 | 0.058 | rs17027258 | 0.089 |
| chr2:102,591,540-103,591,540 | All | gtex.Skin_Sun_Exposed_Lower_leg | *IL1R2* | 2156 | 0.000 | 0.000 | 0.000 | 1.000 | 0.000 | rs17027258 | 0.000 |
| chr2:102,591,540-103,591,540 | All | gtex.Skin_Sun_Exposed_Lower_leg | *IL1R1* | 2156 | 0.000 | 0.541 | 0.000 | 0.160 | 0.299 | rs17027255 | 0.073 |
| chr2:102,591,540-103,591,540 | All | gtex.Skin_Sun_Exposed_Lower_leg | *IL1RL2* | 2156 | 0.000 | 0.000 | 0.000 | 1.000 | 0.000 | rs17027255 | 0.087 |
| chr2:102,591,540-103,591,540 | All | gtex.Skin_Sun_Exposed_Lower_leg | *IL1RL1* | 2156 | 0.000 | 0.754 | 0.000 | 0.171 | 0.075 | rs17027258 | 0.072 |
| chr2:102,591,540-103,591,540 | All | gtex.Skin_Sun_Exposed_Lower_leg | *IL18R1* | 2156 | 0.000 | 0.000 | 0.000 | 1.000 | 0.000 | rs17027258 | 0.063 |
| chr2:102,591,540-103,591,540 | All | gtex.Skin_Sun_Exposed_Lower_leg | *IL18RAP* | 2156 | 0.000 | 0.001 | 0.000 | 0.166 | **0.833** | rs11678975 | 0.065 |
| chr2:102,591,540-103,591,540 | All | gtex.Skin_Sun_Exposed_Lower_leg | *SLC9A2* | 2156 | 0.000 | 0.674 | 0.000 | 0.291 | 0.036 | rs17027258 | 0.084 |
| chr2:102,591,540-103,591,540 | All | gtex.Skin_Sun_Exposed_Lower_leg | *MFSD9* | 2156 | 0.000 | 0.732 | 0.000 | 0.231 | 0.037 | rs17027258 | 0.087 |
| chr2:102,591,540-103,591,540 | All | gtex.Skin_Sun_Exposed_Lower_leg | *TMEM182* | 2156 | 0.000 | 0.775 | 0.000 | 0.183 | 0.042 | rs17027258 | 0.082 |
| chr2:102,591,540-103,591,540 | All | gtex.Skin_Sun_Exposed_Lower_leg | *SLC9A4* | 2156 | 0.000 | 0.643 | 0.000 | 0.163 | 0.193 | rs17027258 | 0.111 |
| chr2:102,591,540-103,591,540 | All | gtex.Skin_Not_Sun_Exposed_Suprapubic | *IL1R2* | 2156 | 0.000 | 0.000 | 0.000 | 1.000 | 0.000 | rs17027258 | 0.000 |
| chr2:102,591,540-103,591,540 | All | gtex.Skin_Not_Sun_Exposed_Suprapubic | *IL1R1* | 2156 | 0.000 | 0.650 | 0.000 | 0.312 | 0.038 | rs17027258 | 0.075 |
| chr2:102,591,540-103,591,540 | All | gtex.Skin_Not_Sun_Exposed_Suprapubic | *IL1RL2* | 2156 | 0.000 | 0.000 | 0.000 | 1.000 | 0.000 | rs17027258 | 0.154 |
| chr2:102,591,540-103,591,540 | All | gtex.Skin_Not_Sun_Exposed_Suprapubic | *IL1RL1* | 2156 | 0.000 | 0.811 | 0.000 | 0.144 | 0.045 | rs17027258 | 0.088 |
| chr2:102,591,540-103,591,540 | All | gtex.Skin_Not_Sun_Exposed_Suprapubic | *IL18R1* | 2156 | 0.000 | 0.004 | 0.000 | 0.994 | 0.002 | rs17027258 | 0.088 |
| chr2:102,591,540-103,591,540 | All | gtex.Skin_Not_Sun_Exposed_Suprapubic | *IL18RAP* | 2156 | 0.000 | 0.039 | 0.000 | 0.532 | 0.429 | rs66566526 | 0.048 |
| chr2:102,591,540-103,591,540 | All | gtex.Skin_Not_Sun_Exposed_Suprapubic | *SLC9A2* | 2156 | 0.000 | 0.804 | 0.000 | 0.152 | 0.044 | rs17027258 | 0.098 |
| chr2:102,591,540-103,591,540 | All | gtex.Skin_Not_Sun_Exposed_Suprapubic | *MFSD9* | 2156 | 0.000 | 0.754 | 0.000 | 0.207 | 0.039 | rs17027258 | 0.084 |
| chr2:102,591,540-103,591,540 | All | gtex.Skin_Not_Sun_Exposed_Suprapubic | *TMEM182* | 2156 | 0.000 | 0.823 | 0.000 | 0.133 | 0.044 | rs17027258 | 0.085 |
| chr2:102,591,540-103,591,540 | All | gtex.Skin_Not_Sun_Exposed_Suprapubic | *SLC9A4* | 2156 | 0.000 | 0.804 | 0.000 | 0.154 | 0.042 | rs17027258 | 0.082 |
| chr1:67,097,119-68,097,119 | All | gtex.Whole_Blood | *IL12RB2* | 1670 | 0.000 | 0.000 | 0.000 | 1.000 | 0.000 | rs3762318 | 0.300 |
| chr1:67,097,119-68,097,119 | All | gtex.Whole_Blood | *SLC35D1* | 1670 | 0.000 | 0.068 | 0.000 | 0.926 | 0.006 | rs3762318 | 0.286 |
| chr1:67,097,119-68,097,119 | All | gtex.Whole_Blood | *SGIP1* | 1422 | 0.000 | 0.000 | 0.000 | 1.000 | 0.000 | rs3762318 | 0.310 |
| chr1:67,097,119-68,097,119 | All | gtex.Whole_Blood | *SERBP1* | 1670 | 0.000 | 0.813 | 0.000 | 0.119 | 0.068 | rs3762318 | 0.316 |
| chr1:67,097,119-68,097,119 | All | gtex.Whole_Blood | *MIER1* | 1670 | 0.000 | 0.002 | 0.000 | 0.998 | 0.000 | rs3762318 | 0.461 |
| chr1:67,097,119-68,097,119 | All | gtex.Nerve_Tibial | *IL12RB2* | 1670 | 0.000 | 0.242 | 0.000 | 0.737 | 0.021 | rs3762318 | 0.364 |
| chr1:67,097,119-68,097,119 | All | gtex.Nerve_Tibial | *SLC35D1* | 1670 | 0.000 | 0.673 | 0.000 | 0.271 | 0.056 | rs3762318 | 0.356 |
| chr1:67,097,119-68,097,119 | All | gtex.Nerve_Tibial | *SGIP1* | 1422 | 0.000 | 0.005 | 0.000 | 0.995 | 0.000 | rs3762318 | 0.274 |
| chr1:67,097,119-68,097,119 | All | gtex.Nerve_Tibial | *SERBP1* | 1670 | 0.000 | 0.000 | 0.000 | 1.000 | 0.000 | rs3762318 | 0.189 |
| chr1:67,097,119-68,097,119 | All | gtex.Nerve_Tibial | *DNAI4* | 1670 | 0.000 | 0.002 | 0.000 | 0.998 | 0.000 | rs3762318 | 0.480 |
| chr1:67,097,119-68,097,119 | All | gtex.Nerve_Tibial | *MIER1* | 1670 | 0.000 | 0.054 | 0.000 | 0.936 | 0.010 | rs78377598 | 0.239 |
| chr1:67,097,119-68,097,119 | All | gtex.Skin_Sun_Exposed_Lower_leg | *IL12RB2* | 1670 | 0.000 | 0.727 | 0.000 | 0.213 | 0.059 | rs3762318 | 0.342 |
| chr1:67,097,119-68,097,119 | All | gtex.Skin_Sun_Exposed_Lower_leg | *SLC35D1* | 1670 | 0.000 | 0.018 | 0.000 | 0.979 | 0.004 | rs3762318 | 0.458 |
| chr1:67,097,119-68,097,119 | All | gtex.Skin_Sun_Exposed_Lower_leg | *SGIP1* | 1422 | 0.000 | 0.125 | 0.000 | 0.862 | 0.013 | rs3762318 | 0.339 |
| chr1:67,097,119-68,097,119 | All | gtex.Skin_Sun_Exposed_Lower_leg | *SERBP1* | 1670 | 0.000 | 0.000 | 0.000 | 1.000 | 0.000 | rs3762318 | 0.318 |
| chr1:67,097,119-68,097,119 | All | gtex.Skin_Sun_Exposed_Lower_leg | *DNAI4* | 1670 | 0.000 | 0.760 | 0.000 | 0.181 | 0.058 | rs3762318 | 0.308 |
| chr1:67,097,119-68,097,119 | All | gtex.Skin_Sun_Exposed_Lower_leg | *MIER1* | 1670 | 0.000 | 0.746 | 0.000 | 0.141 | 0.114 | rs3762318 | 0.514 |
| chr1:67,097,119-68,097,119 | All | gtex.Skin_Not_Sun_Exposed_Suprapubic | *IL12RB2* | 1670 | 0.000 | 0.808 | 0.000 | 0.125 | 0.068 | rs3762318 | 0.351 |
| chr1:67,097,119-68,097,119 | All | gtex.Skin_Not_Sun_Exposed_Suprapubic | *SLC35D1* | 1670 | 0.000 | 0.352 | 0.000 | 0.545 | 0.103 | rs3762318 | 0.637 |
| chr1:67,097,119-68,097,119 | All | gtex.Skin_Not_Sun_Exposed_Suprapubic | *SGIP1* | 1422 | 0.000 | 0.741 | 0.000 | 0.195 | 0.064 | rs3762318 | 0.260 |
| chr1:67,097,119-68,097,119 | All | gtex.Skin_Not_Sun_Exposed_Suprapubic | *SERBP1* | 1670 | 0.000 | 0.001 | 0.000 | 0.999 | 0.000 | rs3762318 | 0.287 |
| chr1:67,097,119-68,097,119 | All | gtex.Skin_Not_Sun_Exposed_Suprapubic | *DNAI4* | 1670 | 0.000 | 0.722 | 0.000 | 0.215 | 0.063 | rs3762318 | 0.344 |
| chr1:67,097,119-68,097,119 | All | gtex.Skin_Not_Sun_Exposed_Suprapubic | *MIER1* | 1670 | 0.000 | 0.779 | 0.000 | 0.146 | 0.075 | rs3762318 | 0.423 |
| chr16:28,008,048-29,008,048 | All | gtex.Whole_Blood | *ATXN2L* | 861 | 0.000 | 0.352 | 0.000 | 0.326 | 0.321 | rs181209 | 0.082 |
| chr16:28,008,048-29,008,048 | All | gtex.Whole_Blood | *XPO6* | 861 | 0.000 | 0.002 | 0.000 | 0.998 | 0.000 | rs180744 | 0.102 |
| chr16:28,008,048-29,008,048 | All | gtex.Whole_Blood | *GSG1L* | 861 | 0.000 | 0.800 | 0.000 | 0.113 | 0.086 | rs180744 | 0.092 |
| chr16:28,008,048-29,008,048 | All | gtex.Whole_Blood | *SPNS1* | 861 | 0.000 | 0.000 | 0.000 | 0.999 | 0.001 | rs180744 | 0.001 |
| chr16:28,008,048-29,008,048 | All | gtex.Whole_Blood | *NUPR1* | 861 | 0.000 | 0.814 | 0.000 | 0.087 | 0.099 | rs180744 | 0.192 |
| chr16:28,008,048-29,008,048 | All | gtex.Whole_Blood | *NFATC2IP* | 861 | 0.000 | 0.862 | 0.000 | 0.070 | 0.069 | rs180744 | 0.114 |
| chr16:28,008,048-29,008,048 | All | gtex.Whole_Blood | *CD19* | 861 | 0.000 | 0.881 | 0.000 | 0.057 | 0.062 | rs180744 | 0.109 |
| chr16:28,008,048-29,008,048 | All | gtex.Whole_Blood | *RABEP2* | 861 | 0.000 | 0.878 | 0.000 | 0.062 | 0.059 | rs180744 | 0.105 |
| chr16:28,008,048-29,008,048 | All | gtex.Whole_Blood | *SH2B1* | 861 | 0.000 | 0.063 | 0.000 | 0.495 | 0.441 | rs151174 | 0.115 |
| chr16:28,008,048-29,008,048 | All | gtex.Whole_Blood | *TUFM* | 861 | 0.000 | 0.000 | 0.000 | 0.723 | 0.277 | rs180744 | 0.000 |
| chr16:28,008,048-29,008,048 | All | gtex.Whole_Blood | *EIF3C* | 861 | 0.000 | 0.358 | 0.000 | 0.367 | 0.275 | rs180743 | 0.155 |
| chr16:28,008,048-29,008,048 | All | gtex.Whole_Blood | *APOBR* | 861 | 0.000 | 0.741 | 0.000 | 0.095 | 0.164 | rs180743 | 0.163 |
| chr16:28,008,048-29,008,048 | All | gtex.Whole_Blood | *SBK1* | 861 | 0.000 | 0.800 | 0.000 | 0.087 | 0.112 | rs180744 | 0.099 |
| chr16:28,008,048-29,008,048 | All | gtex.Whole_Blood | *CLN3* | 861 | 0.000 | 0.106 | 0.000 | 0.836 | 0.058 | rs180743 | 0.069 |
| chr16:28,008,048-29,008,048 | All | gtex.Whole_Blood | *ATP2A1* | 861 | 0.000 | 0.756 | 0.000 | 0.113 | 0.131 | rs180744 | 0.120 |
| chr16:28,008,048-29,008,048 | All | gtex.Whole_Blood | *SULT1A1* | 861 | 0.000 | 0.000 | 0.000 | 0.880 | 0.120 | rs75539558 | 0.077 |
| chr16:28,008,048-29,008,048 | All | gtex.Whole_Blood | *SULT1A2* | 861 | 0.000 | 0.000 | 0.000 | 0.987 | 0.013 | rs180744 | 0.000 |
| chr16:28,008,048-29,008,048 | All | gtex.Whole_Blood | *IL27* | 861 | 0.000 | 0.386 | 0.000 | 0.145 | 0.469 | rs180744 | 0.181 |
| chr16:28,008,048-29,008,048 | All | gtex.Whole_Blood | *NPIPB6* | 861 | 0.000 | 0.548 | 0.000 | 0.214 | 0.238 | rs180744 | 0.100 |
| chr16:28,008,048-29,008,048 | All | gtex.Whole_Blood | *EIF3CL* | 861 | 0.000 | 0.821 | 0.000 | 0.117 | 0.062 | rs180744 | 0.099 |
| chr16:28,008,048-29,008,048 | All | gtex.Whole_Blood | *LAT* | 861 | 0.000 | 0.848 | 0.000 | 0.080 | 0.072 | rs180744 | 0.138 |
| chr16:28,008,048-29,008,048 | All | gtex.Nerve_Tibial | *ATXN2L* | 861 | 0.000 | 0.850 | 0.000 | 0.071 | 0.078 | rs180743 | 0.099 |
| chr16:28,008,048-29,008,048 | All | gtex.Nerve_Tibial | *XPO6* | 861 | 0.000 | 0.000 | 0.000 | 1.000 | 0.000 | rs180744 | 0.073 |
| chr16:28,008,048-29,008,048 | All | gtex.Nerve_Tibial | *GSG1L* | 861 | 0.000 | 0.810 | 0.000 | 0.072 | 0.117 | rs180744 | 0.131 |
| chr16:28,008,048-29,008,048 | All | gtex.Nerve_Tibial | *SPNS1* | 861 | 0.000 | 0.731 | 0.000 | 0.218 | 0.051 | rs180744 | 0.130 |
| chr16:28,008,048-29,008,048 | All | gtex.Nerve_Tibial | *NUPR1* | 861 | 0.000 | 0.704 | 0.000 | 0.081 | 0.215 | rs11074911 | 0.043 |
| chr16:28,008,048-29,008,048 | All | gtex.Nerve_Tibial | *NFATC2IP* | 861 | 0.000 | 0.729 | 0.000 | 0.097 | 0.174 | rs181207 | 0.178 |
| chr16:28,008,048-29,008,048 | All | gtex.Nerve_Tibial | *CD19* | 861 | 0.000 | 0.877 | 0.000 | 0.055 | 0.067 | rs180744 | 0.096 |
| chr16:28,008,048-29,008,048 | All | gtex.Nerve_Tibial | *RABEP2* | 861 | 0.000 | 0.876 | 0.000 | 0.060 | 0.064 | rs180744 | 0.098 |
| chr16:28,008,048-29,008,048 | All | gtex.Nerve_Tibial | *SH2B1* | 861 | 0.000 | 0.017 | 0.000 | 0.525 | 0.458 | rs11074911 | 0.050 |
| chr16:28,008,048-29,008,048 | All | gtex.Nerve_Tibial | *TUFM* | 861 | 0.000 | 0.000 | 0.000 | 0.858 | 0.142 | rs180744 | 0.000 |
| chr16:28,008,048-29,008,048 | All | gtex.Nerve_Tibial | *EIF3C* | 861 | 0.000 | 0.591 | 0.000 | 0.172 | 0.237 | rs181209 | 0.097 |
| chr16:28,008,048-29,008,048 | All | gtex.Nerve_Tibial | *APOBR* | 861 | 0.000 | 0.697 | 0.000 | 0.087 | 0.216 | rs149271 | 0.177 |
| chr16:28,008,048-29,008,048 | All | gtex.Nerve_Tibial | *SBK1* | 861 | 0.000 | 0.793 | 0.000 | 0.081 | 0.126 | rs180744 | 0.154 |
| chr16:28,008,048-29,008,048 | All | gtex.Nerve_Tibial | *CLN3* | 861 | 0.000 | 0.694 | 0.000 | 0.108 | 0.198 | rs180743 | 0.144 |
| chr16:28,008,048-29,008,048 | All | gtex.Nerve_Tibial | *ATP2A1* | 861 | 0.000 | 0.140 | 0.000 | 0.384 | 0.477 | rs180744 | 0.071 |
| chr16:28,008,048-29,008,048 | All | gtex.Nerve_Tibial | *SULT1A1* | 861 | 0.000 | 0.000 | 0.000 | 0.975 | 0.025 | rs149271 | 0.000 |
| chr16:28,008,048-29,008,048 | All | gtex.Nerve_Tibial | *NPIPB9* | 861 | 0.000 | 0.021 | 0.000 | 0.795 | 0.184 | rs149271 | 0.093 |
| chr16:28,008,048-29,008,048 | All | gtex.Nerve_Tibial | *SULT1A2* | 861 | 0.000 | 0.000 | 0.000 | 0.995 | 0.005 | rs180743 | 0.000 |
| chr16:28,008,048-29,008,048 | All | gtex.Nerve_Tibial | *IL27* | 861 | 0.000 | 0.857 | 0.000 | 0.073 | 0.070 | rs180744 | 0.117 |
| chr16:28,008,048-29,008,048 | All | gtex.Nerve_Tibial | *NPIPB6* | 861 | 0.000 | 0.886 | 0.000 | 0.052 | 0.062 | rs180744 | 0.105 |
| chr16:28,008,048-29,008,048 | All | gtex.Nerve_Tibial | *EIF3CL* | 861 | 0.000 | 0.686 | 0.000 | 0.237 | 0.077 | rs180744 | 0.125 |
| chr16:28,008,048-29,008,048 | All | gtex.Nerve_Tibial | *LAT* | 861 | 0.000 | 0.858 | 0.000 | 0.072 | 0.070 | rs180744 | 0.116 |
| chr16:28,008,048-29,008,048 | All | gtex.Nerve_Tibial | *ATP2A1-AS1* | 861 | 0.000 | 0.877 | 0.000 | 0.062 | 0.062 | rs180744 | 0.116 |
| chr16:28,008,048-29,008,048 | All | gtex.Skin_Sun_Exposed_Lower_leg | *ATXN2L* | 861 | 0.000 | 0.854 | 0.000 | 0.086 | 0.061 | rs180744 | 0.114 |
| chr16:28,008,048-29,008,048 | All | gtex.Skin_Sun_Exposed_Lower_leg | *XPO6* | 861 | 0.000 | 0.000 | 0.000 | 1.000 | 0.000 | rs180743 | 0.000 |
| chr16:28,008,048-29,008,048 | All | gtex.Skin_Sun_Exposed_Lower_leg | *GSG1L* | 861 | 0.000 | 0.733 | 0.000 | 0.218 | 0.050 | rs180744 | 0.104 |
| chr16:28,008,048-29,008,048 | All | gtex.Skin_Sun_Exposed_Lower_leg | *SPNS1* | 861 | 0.000 | 0.837 | 0.000 | 0.094 | 0.069 | rs180744 | 0.110 |
| chr16:28,008,048-29,008,048 | All | gtex.Skin_Sun_Exposed_Lower_leg | *NUPR1* | 861 | 0.000 | 0.722 | 0.000 | 0.084 | 0.194 | rs181206 | 0.053 |
| chr16:28,008,048-29,008,048 | All | gtex.Skin_Sun_Exposed_Lower_leg | *NFATC2IP* | 861 | 0.000 | 0.368 | 0.000 | 0.526 | 0.106 | rs180743 | 0.093 |
| chr16:28,008,048-29,008,048 | All | gtex.Skin_Sun_Exposed_Lower_leg | *CD19* | 861 | 0.000 | 0.866 | 0.000 | 0.070 | 0.064 | rs180744 | 0.103 |
| chr16:28,008,048-29,008,048 | All | gtex.Skin_Sun_Exposed_Lower_leg | *RABEP2* | 861 | 0.000 | 0.653 | 0.000 | 0.226 | 0.121 | rs180743 | 0.081 |
| chr16:28,008,048-29,008,048 | All | gtex.Skin_Sun_Exposed_Lower_leg | *SH2B1* | 861 | 0.000 | 0.785 | 0.000 | 0.116 | 0.099 | rs180744 | 0.175 |
| chr16:28,008,048-29,008,048 | All | gtex.Skin_Sun_Exposed_Lower_leg | *TUFM* | 861 | 0.000 | 0.000 | 0.000 | 0.772 | 0.227 | rs180743 | 0.000 |
| chr16:28,008,048-29,008,048 | All | gtex.Skin_Sun_Exposed_Lower_leg | *EIF3C* | 861 | 0.000 | 0.027 | 0.000 | 0.697 | 0.276 | rs180744 | 0.097 |
| chr16:28,008,048-29,008,048 | All | gtex.Skin_Sun_Exposed_Lower_leg | *APOBR* | 861 | 0.000 | 0.866 | 0.000 | 0.054 | 0.080 | rs180744 | 0.110 |
| chr16:28,008,048-29,008,048 | All | gtex.Skin_Sun_Exposed_Lower_leg | *SBK1* | 861 | 0.000 | 0.002 | 0.000 | 0.871 | 0.127 | rs180743 | 0.161 |
| chr16:28,008,048-29,008,048 | All | gtex.Skin_Sun_Exposed_Lower_leg | *CLN3* | 861 | 0.000 | 0.496 | 0.000 | 0.128 | 0.377 | rs180744 | 0.104 |
| chr16:28,008,048-29,008,048 | All | gtex.Skin_Sun_Exposed_Lower_leg | *ATP2A1* | 861 | 0.000 | 0.189 | 0.000 | 0.341 | 0.470 | rs180744 | 0.147 |
| chr16:28,008,048-29,008,048 | All | gtex.Skin_Sun_Exposed_Lower_leg | *SULT1A1* | 861 | 0.000 | 0.002 | 0.000 | 0.300 | 0.698 | rs12447461 | 0.047 |
| chr16:28,008,048-29,008,048 | All | gtex.Skin_Sun_Exposed_Lower_leg | *NPIPB9* | 861 | 0.000 | 0.762 | 0.000 | 0.121 | 0.117 | rs180743 | 0.086 |
| chr16:28,008,048-29,008,048 | All | gtex.Skin_Sun_Exposed_Lower_leg | *SULT1A2* | 861 | 0.000 | 0.000 | 0.000 | 0.859 | 0.141 | rs180743 | 0.195 |
| chr16:28,008,048-29,008,048 | All | gtex.Skin_Sun_Exposed_Lower_leg | *IL27* | 861 | 0.000 | 0.851 | 0.000 | 0.071 | 0.078 | rs180744 | 0.077 |
| chr16:28,008,048-29,008,048 | All | gtex.Skin_Sun_Exposed_Lower_leg | *NPIPB6* | 861 | 0.000 | 0.003 | 0.000 | 0.291 | 0.706 | rs180744 | 0.191 |
| chr16:28,008,048-29,008,048 | All | gtex.Skin_Sun_Exposed_Lower_leg | *EIF3CL* | 861 | 0.000 | 0.605 | 0.000 | 0.350 | 0.045 | rs180744 | 0.099 |
| chr16:28,008,048-29,008,048 | All | gtex.Skin_Sun_Exposed_Lower_leg | *LAT* | 861 | 0.000 | 0.856 | 0.000 | 0.058 | 0.086 | rs180744 | 0.111 |
| chr16:28,008,048-29,008,048 | All | gtex.Skin_Sun_Exposed_Lower_leg | *ATP2A1-AS1* | 861 | 0.000 | 0.843 | 0.000 | 0.062 | 0.096 | rs180744 | 0.083 |
| chr16:28,008,048-29,008,048 | All | gtex.Skin_Not_Sun_Exposed_Suprapubic | *ATXN2L* | 861 | 0.000 | 0.824 | 0.000 | 0.078 | 0.098 | rs180743 | 0.114 |
| chr16:28,008,048-29,008,048 | All | gtex.Skin_Not_Sun_Exposed_Suprapubic | *XPO6* | 861 | 0.000 | 0.000 | 0.000 | 1.000 | 0.000 | rs180743 | 0.104 |
| chr16:28,008,048-29,008,048 | All | gtex.Skin_Not_Sun_Exposed_Suprapubic | *GSG1L* | 861 | 0.000 | 0.862 | 0.000 | 0.073 | 0.065 | rs180744 | 0.112 |
| chr16:28,008,048-29,008,048 | All | gtex.Skin_Not_Sun_Exposed_Suprapubic | *SPNS1* | 861 | 0.000 | 0.875 | 0.000 | 0.061 | 0.063 | rs180744 | 0.132 |
| chr16:28,008,048-29,008,048 | All | gtex.Skin_Not_Sun_Exposed_Suprapubic | *NUPR1* | 861 | 0.000 | 0.581 | 0.000 | 0.142 | 0.277 | rs180744 | 0.060 |
| chr16:28,008,048-29,008,048 | All | gtex.Skin_Not_Sun_Exposed_Suprapubic | *NFATC2IP* | 861 | 0.000 | 0.144 | 0.000 | 0.476 | 0.381 | rs180744 | 0.376 |
| chr16:28,008,048-29,008,048 | All | gtex.Skin_Not_Sun_Exposed_Suprapubic | *CD19* | 861 | 0.000 | 0.836 | 0.000 | 0.106 | 0.058 | rs180744 | 0.100 |
| chr16:28,008,048-29,008,048 | All | gtex.Skin_Not_Sun_Exposed_Suprapubic | *RABEP2* | 861 | 0.000 | 0.587 | 0.000 | 0.114 | 0.299 | rs180743 | 0.074 |
| chr16:28,008,048-29,008,048 | All | gtex.Skin_Not_Sun_Exposed_Suprapubic | *SH2B1* | 861 | 0.000 | 0.415 | 0.000 | 0.274 | 0.311 | rs180743 | 0.105 |
| chr16:28,008,048-29,008,048 | All | gtex.Skin_Not_Sun_Exposed_Suprapubic | *TUFM* | 861 | 0.000 | 0.000 | 0.000 | 0.858 | 0.142 | rs180744 | 0.000 |
| chr16:28,008,048-29,008,048 | All | gtex.Skin_Not_Sun_Exposed_Suprapubic | *EIF3C* | 861 | 0.000 | 0.509 | 0.000 | 0.157 | 0.334 | rs181207 | 0.093 |
| chr16:28,008,048-29,008,048 | All | gtex.Skin_Not_Sun_Exposed_Suprapubic | *APOBR* | 861 | 0.000 | 0.087 | 0.000 | 0.416 | 0.497 | rs180743 | 0.212 |
| chr16:28,008,048-29,008,048 | All | gtex.Skin_Not_Sun_Exposed_Suprapubic | *SBK1* | 861 | 0.000 | 0.004 | 0.000 | 0.565 | 0.432 | rs180743 | 0.166 |
| chr16:28,008,048-29,008,048 | All | gtex.Skin_Not_Sun_Exposed_Suprapubic | *CLN3* | 861 | 0.000 | 0.882 | 0.000 | 0.053 | 0.065 | rs180744 | 0.125 |
| chr16:28,008,048-29,008,048 | All | gtex.Skin_Not_Sun_Exposed_Suprapubic | *ATP2A1* | 861 | 0.000 | 0.397 | 0.000 | 0.200 | 0.402 | rs181207 | 0.105 |
| chr16:28,008,048-29,008,048 | All | gtex.Skin_Not_Sun_Exposed_Suprapubic | *SULT1A1* | 861 | 0.000 | 0.002 | 0.000 | 0.906 | 0.092 | rs181207 | 0.158 |
| chr16:28,008,048-29,008,048 | All | gtex.Skin_Not_Sun_Exposed_Suprapubic | *NPIPB9* | 861 | 0.000 | 0.802 | 0.000 | 0.131 | 0.067 | rs180744 | 0.107 |
| chr16:28,008,048-29,008,048 | All | gtex.Skin_Not_Sun_Exposed_Suprapubic | *SULT1A2* | 861 | 0.000 | 0.001 | 0.000 | 0.157 | **0.842** | rs180744 | 0.132 |
| chr16:28,008,048-29,008,048 | All | gtex.Skin_Not_Sun_Exposed_Suprapubic | *NPIPB6* | 861 | 0.000 | 0.880 | 0.000 | 0.060 | 0.060 | rs180744 | 0.107 |
| chr16:28,008,048-29,008,048 | All | gtex.Skin_Not_Sun_Exposed_Suprapubic | *EIF3CL* | 861 | 0.000 | 0.880 | 0.000 | 0.058 | 0.063 | rs180744 | 0.097 |
| chr16:28,008,048-29,008,048 | All | gtex.Skin_Not_Sun_Exposed_Suprapubic | *LAT* | 861 | 0.000 | 0.874 | 0.000 | 0.063 | 0.063 | rs180744 | 0.113 |
| chr16:28,008,048-29,008,048 | All | gtex.Skin_Not_Sun_Exposed_Suprapubic | *ATP2A1-AS1* | 861 | 0.000 | 0.874 | 0.000 | 0.057 | 0.069 | rs180744 | 0.090 |
| chr13:43,959,499-45,012,908 | First | gtex.Whole_Blood | *CCDC122* | 2013 | 0.000 | 0.181 | 0.000 | 0.667 | 0.152 | rs3764147 | 0.481 |
| chr13:43,959,499-45,012,908 | First | gtex.Whole_Blood | *SERP2* | 2013 | 0.000 | 0.772 | 0.000 | 0.182 | 0.046 | rs3764147 | 0.489 |
| chr13:43,959,499-45,012,908 | First | gtex.Whole_Blood | *LACC1* | 2013 | 0.000 | 0.787 | 0.000 | 0.174 | 0.039 | rs3764147 | 0.481 |
| chr13:43,959,499-45,012,908 | First | gtex.Nerve_Tibial | *ENOX1* | 2013 | 0.000 | 0.158 | 0.000 | 0.833 | 0.009 | rs3764147 | 0.481 |
| chr13:43,959,499-45,012,908 | First | gtex.Nerve_Tibial | *CCDC122* | 2013 | 0.000 | 0.000 | 0.000 | 1.000 | 0.000 | rs3764147 | 0.233 |
| chr13:43,959,499-45,012,908 | First | gtex.Nerve_Tibial | *SERP2* | 2013 | 0.000 | 0.776 | 0.000 | 0.182 | 0.041 | rs3764147 | 0.481 |
| chr13:43,959,499-45,012,908 | First | gtex.Nerve_Tibial | *LACC1* | 2013 | 0.000 | 0.024 | 0.000 | 0.807 | 0.168 | rs3764147 | 0.530 |
| chr13:43,959,499-45,012,908 | First | gtex.Nerve_Tibial | *SMIM2-AS1* | 2013 | 0.000 | 0.041 | 0.000 | 0.956 | 0.002 | rs3764147 | 0.483 |
| chr13:43,959,499-45,012,908 | First | gtex.Nerve_Tibial | *NRAD1* | 2013 | 0.000 | 0.792 | 0.000 | 0.134 | 0.075 | rs3764147 | 0.506 |
| chr13:43,959,499-45,012,908 | First | gtex.Skin_Sun_Exposed_Lower_leg | *ENOX1* | 2013 | 0.000 | 0.791 | 0.000 | 0.163 | 0.046 | rs3764147 | 0.494 |
| chr13:43,959,499-45,012,908 | First | gtex.Skin_Sun_Exposed_Lower_leg | *CCDC122* | 2013 | 0.000 | 0.003 | 0.000 | 0.996 | 0.000 | rs3764147 | 0.477 |
| chr13:43,959,499-45,012,908 | First | gtex.Skin_Sun_Exposed_Lower_leg | *SERP2* | 2013 | 0.000 | 0.703 | 0.000 | 0.154 | 0.143 | rs3764147 | 0.500 |
| chr13:43,959,499-45,012,908 | First | gtex.Skin_Sun_Exposed_Lower_leg | *LACC1* | 2013 | 0.000 | 0.154 | 0.000 | 0.388 | 0.458 | rs3764147 | 0.485 |
| chr13:43,959,499-45,012,908 | First | gtex.Skin_Sun_Exposed_Lower_leg | *SMIM2-AS1* | 2013 | 0.000 | 0.000 | 0.000 | 1.000 | 0.000 | rs3764147 | 0.493 |
| chr13:43,959,499-45,012,908 | First | gtex.Skin_Sun_Exposed_Lower_leg | *TUSC8* | 1976 | 0.000 | 0.676 | 0.000 | 0.134 | 0.190 | rs3764147 | 0.534 |
| chr13:43,959,499-45,012,908 | First | gtex.Skin_Not_Sun_Exposed_Suprapubic | *ENOX1* | 2013 | 0.000 | 0.690 | 0.000 | 0.212 | 0.098 | rs3764147 | 0.414 |
| chr13:43,959,499-45,012,908 | First | gtex.Skin_Not_Sun_Exposed_Suprapubic | *CCDC122* | 2013 | 0.000 | 0.003 | 0.000 | 0.997 | 0.001 | rs3764147 | 0.526 |
| chr13:43,959,499-45,012,908 | First | gtex.Skin_Not_Sun_Exposed_Suprapubic | *SERP2* | 2013 | 0.000 | 0.769 | 0.000 | 0.161 | 0.070 | rs3764147 | 0.525 |
| chr13:43,959,499-45,012,908 | First | gtex.Skin_Not_Sun_Exposed_Suprapubic | *LACC1* | 2013 | 0.000 | 0.532 | 0.000 | 0.157 | 0.311 | rs3764147 | 0.493 |
| chr13:43,959,499-45,012,908 | First | gtex.Skin_Not_Sun_Exposed_Suprapubic | *SMIM2-AS1* | 2013 | 0.000 | 0.000 | 0.000 | 1.000 | 0.000 | rs3764147 | 0.479 |
| chr13:43,959,499-45,012,908 | First | gtex.Skin_Not_Sun_Exposed_Suprapubic | *TUSC8* | 1976 | 0.000 | 0.794 | 0.000 | 0.167 | 0.039 | rs3764147 | 0.483 |
| chr13:43,959,499-45,012,908 | Second | gtex.Whole_Blood | *CCDC122* | 1799 | 0.000 | 0.442 | 0.000 | 0.534 | 0.024 | . | . |
| chr13:43,959,499-45,012,908 | Second | gtex.Whole_Blood | *SERP2* | 1799 | 0.000 | 0.791 | 0.000 | 0.141 | 0.068 | . | . |
| chr13:43,959,499-45,012,908 | Second | gtex.Whole_Blood | *LACC1* | 1799 | 0.000 | 0.628 | 0.000 | 0.116 | 0.256 | . | . |
| chr13:43,959,499-45,012,908 | Second | gtex.Nerve_Tibial | *ENOX1* | 1799 | 0.000 | 0.159 | 0.000 | 0.829 | 0.012 | . | . |
| chr13:43,959,499-45,012,908 | Second | gtex.Nerve_Tibial | *CCDC122* | 1799 | 0.000 | 0.000 | 0.000 | 1.000 | 0.000 | . | . |
| chr13:43,959,499-45,012,908 | Second | gtex.Nerve_Tibial | *SERP2* | 1799 | 0.000 | 0.766 | 0.000 | 0.171 | 0.063 | . | . |
| chr13:43,959,499-45,012,908 | Second | gtex.Nerve_Tibial | *LACC1* | 1799 | 0.000 | 0.228 | 0.000 | 0.320 | 0.451 | . | . |
| chr13:43,959,499-45,012,908 | Second | gtex.Nerve_Tibial | *SMIM2-AS1* | 1799 | 0.000 | 0.039 | 0.000 | 0.905 | 0.056 | . | . |
| chr13:43,959,499-45,012,908 | Second | gtex.Nerve_Tibial | *NRAD1* | 1799 | 0.000 | 0.814 | 0.000 | 0.125 | 0.061 | . | . |
| chr13:43,959,499-45,012,908 | Second | gtex.Skin_Sun_Exposed_Lower_leg | *ENOX1* | 1799 | 0.000 | 0.802 | 0.000 | 0.155 | 0.043 | . | . |
| chr13:43,959,499-45,012,908 | Second | gtex.Skin_Sun_Exposed_Lower_leg | *CCDC122* | 1799 | 0.000 | 0.003 | 0.000 | 0.996 | 0.001 | . | . |
| chr13:43,959,499-45,012,908 | Second | gtex.Skin_Sun_Exposed_Lower_leg | *SERP2* | 1799 | 0.000 | 0.789 | 0.000 | 0.156 | 0.055 | . | . |
| chr13:43,959,499-45,012,908 | Second | gtex.Skin_Sun_Exposed_Lower_leg | *LACC1* | 1799 | 0.000 | 0.643 | 0.000 | 0.304 | 0.053 | . | . |
| chr13:43,959,499-45,012,908 | Second | gtex.Skin_Sun_Exposed_Lower_leg | *SMIM2-AS1* | 1799 | 0.000 | 0.000 | 0.000 | 1.000 | 0.000 | . | . |
| chr13:43,959,499-45,012,908 | Second | gtex.Skin_Sun_Exposed_Lower_leg | *TUSC8* | 1799 | 0.000 | 0.794 | 0.000 | 0.148 | 0.058 | . | . |
| chr13:43,959,499-45,012,908 | Second | gtex.Skin_Not_Sun_Exposed_Suprapubic | *ENOX1* | 1799 | 0.000 | 0.755 | 0.000 | 0.189 | 0.056 | . | . |
| chr13:43,959,499-45,012,908 | Second | gtex.Skin_Not_Sun_Exposed_Suprapubic | *CCDC122* | 1799 | 0.000 | 0.003 | 0.000 | 0.996 | 0.000 | . | . |
| chr13:43,959,499-45,012,908 | Second | gtex.Skin_Not_Sun_Exposed_Suprapubic | *SERP2* | 1799 | 0.000 | 0.800 | 0.000 | 0.157 | 0.043 | . | . |
| chr13:43,959,499-45,012,908 | Second | gtex.Skin_Not_Sun_Exposed_Suprapubic | *LACC1* | 1799 | 0.000 | 0.767 | 0.000 | 0.192 | 0.041 | . | . |
| chr13:43,959,499-45,012,908 | Second | gtex.Skin_Not_Sun_Exposed_Suprapubic | *SMIM2-AS1* | 1799 | 0.000 | 0.000 | 0.000 | 1.000 | 0.000 | . | . |
| chr13:43,959,499-45,012,908 | Second | gtex.Skin_Not_Sun_Exposed_Suprapubic | *TUSC8* | 1799 | 0.000 | 0.788 | 0.000 | 0.168 | 0.044 | . | . |
| chr12:40,190,061-41,190,061 | All | gtex.Whole_Blood | *SLC2A13* | 2443 | 0.000 | 0.756 | 0.000 | 0.200 | 0.045 | rs17443815 | 0.081 |
| chr12:40,190,061-41,190,061 | All | gtex.Whole_Blood | *LRRK2* | 2443 | 0.000 | 0.000 | 0.000 | 0.864 | 0.136 | rs10878249 | 0.000 |
| chr12:40,190,061-41,190,061 | All | gtex.Nerve_Tibial | *CNTN1* | 2443 | 0.000 | 0.045 | 0.000 | 0.953 | 0.003 | rs17443815 | 0.081 |
| chr12:40,190,061-41,190,061 | All | gtex.Nerve_Tibial | *SLC2A13* | 2443 | 0.000 | 0.628 | 0.000 | 0.307 | 0.065 | rs7973479 | 0.108 |
| chr12:40,190,061-41,190,061 | All | gtex.Nerve_Tibial | *LRRK2* | 2443 | 0.000 | 0.000 | 0.000 | 0.992 | 0.008 | rs4272849 | 0.067 |
| chr12:40,190,061-41,190,061 | All | gtex.Skin_Sun_Exposed_Lower_leg | *CNTN1* | 2443 | 0.000 | 0.000 | 0.000 | 1.000 | 0.000 | rs17443815 | 0.000 |
| chr12:40,190,061-41,190,061 | All | gtex.Skin_Sun_Exposed_Lower_leg | *SLC2A13* | 2443 | 0.000 | 0.709 | 0.000 | 0.241 | 0.050 | rs17443815 | 0.072 |
| chr12:40,190,061-41,190,061 | All | gtex.Skin_Sun_Exposed_Lower_leg | *LRRK2* | 2443 | 0.000 | 0.000 | 0.000 | 0.889 | 0.111 | rs12146857 | 0.017 |
| chr12:40,190,061-41,190,061 | All | gtex.Skin_Not_Sun_Exposed_Suprapubic | *CNTN1* | 2443 | 0.000 | 0.000 | 0.000 | 1.000 | 0.000 | rs12146857 | 0.000 |
| chr12:40,190,061-41,190,061 | All | gtex.Skin_Not_Sun_Exposed_Suprapubic | *SLC2A13* | 2443 | 0.000 | 0.758 | 0.000 | 0.197 | 0.045 | rs12820920 | 0.078 |
| chr12:40,190,061-41,190,061 | All | gtex.Skin_Not_Sun_Exposed_Suprapubic | *LRRK2* | 2443 | 0.000 | 0.000 | 0.000 | 0.999 | 0.000 | rs10878249 | 0.072 |
| chr16:50,223,371-51,223,371 | All | gtex.Whole_Blood | *CYLD* | 1407 | 0.000 | 0.805 | 0.000 | 0.134 | 0.061 | rs8063362 | 0.203 |
| chr16:50,223,371-51,223,371 | All | gtex.Whole_Blood | *TENT4B* | 1360 | 0.000 | 0.722 | 0.000 | 0.098 | 0.181 | rs9939349 | 0.202 |
| chr16:50,223,371-51,223,371 | All | gtex.Whole_Blood | *ADCY7* | 1407 | 0.000 | 0.000 | 0.000 | 1.000 | 0.000 | rs8063362 | 0.173 |
| chr16:50,223,371-51,223,371 | All | gtex.Whole_Blood | *NKD1* | 1407 | 0.000 | 0.829 | 0.000 | 0.113 | 0.058 | rs8063362 | 0.204 |
| chr16:50,223,371-51,223,371 | All | gtex.Whole_Blood | *BRD7* | 1407 | 0.000 | 0.000 | 0.000 | 1.000 | 0.000 | rs8063362 | 0.189 |
| chr16:50,223,371-51,223,371 | All | gtex.Whole_Blood | *NOD2* | 1407 | 0.000 | 0.000 | 0.000 | 0.009 | **0.991** | rs1981760 | 0.395 |
| chr16:50,223,371-51,223,371 | All | gtex.Whole_Blood | *SNX20* | 1407 | 0.000 | 0.721 | 0.000 | 0.154 | 0.125 | rs8063362 | 0.240 |
| chr16:50,223,371-51,223,371 | All | gtex.Nerve_Tibial | *CYLD* | 1407 | 0.000 | 0.141 | 0.000 | 0.768 | 0.091 | rs8063362 | 0.270 |
| chr16:50,223,371-51,223,371 | All | gtex.Nerve_Tibial | *SALL1* | 1407 | 0.000 | 0.803 | 0.000 | 0.100 | 0.097 | rs9939349 | 0.203 |
| chr16:50,223,371-51,223,371 | All | gtex.Nerve_Tibial | *TENT4B* | 1360 | 0.000 | 0.844 | 0.000 | 0.108 | 0.048 | rs8063362 | 0.183 |
| chr16:50,223,371-51,223,371 | All | gtex.Nerve_Tibial | *ADCY7* | 1407 | 0.000 | 0.812 | 0.000 | 0.144 | 0.044 | rs8063362 | 0.191 |
| chr16:50,223,371-51,223,371 | All | gtex.Nerve_Tibial | *NKD1* | 1407 | 0.000 | 0.755 | 0.000 | 0.196 | 0.049 | rs2287195 | 0.210 |
| chr16:50,223,371-51,223,371 | All | gtex.Nerve_Tibial | *BRD7* | 1407 | 0.000 | 0.825 | 0.000 | 0.131 | 0.044 | rs8063362 | 0.189 |
| chr16:50,223,371-51,223,371 | All | gtex.Nerve_Tibial | *NOD2* | 1407 | 0.000 | 0.001 | 0.000 | 0.025 | **0.975** | rs9939349 | 0.291 |
| chr16:50,223,371-51,223,371 | All | gtex.Nerve_Tibial | *SNX20* | 1407 | 0.000 | 0.797 | 0.000 | 0.127 | 0.075 | rs8063362 | 0.190 |
| chr16:50,223,371-51,223,371 | All | gtex.Skin_Sun_Exposed_Lower_leg | *CYLD* | 1407 | 0.000 | 0.017 | 0.000 | 0.976 | 0.007 | rs8063362 | 0.221 |
| chr16:50,223,371-51,223,371 | All | gtex.Skin_Sun_Exposed_Lower_leg | *SALL1* | 1407 | 0.000 | 0.843 | 0.000 | 0.106 | 0.051 | rs8063362 | 0.199 |
| chr16:50,223,371-51,223,371 | All | gtex.Skin_Sun_Exposed_Lower_leg | *TENT4B* | 1360 | 0.000 | 0.852 | 0.000 | 0.102 | 0.045 | rs8063362 | 0.190 |
| chr16:50,223,371-51,223,371 | All | gtex.Skin_Sun_Exposed_Lower_leg | *ADCY7* | 1407 | 0.000 | 0.709 | 0.000 | 0.138 | 0.153 | rs2287195 | 0.221 |
| chr16:50,223,371-51,223,371 | All | gtex.Skin_Sun_Exposed_Lower_leg | *NKD1* | 1407 | 0.000 | 0.804 | 0.000 | 0.143 | 0.053 | rs8063362 | 0.199 |
| chr16:50,223,371-51,223,371 | All | gtex.Skin_Sun_Exposed_Lower_leg | *BRD7* | 1407 | 0.000 | 0.846 | 0.000 | 0.105 | 0.049 | rs2287195 | 0.189 |
| chr16:50,223,371-51,223,371 | All | gtex.Skin_Sun_Exposed_Lower_leg | *NOD2* | 1407 | 0.000 | 0.000 | 0.000 | 1.000 | 0.000 | rs8063362 | 0.290 |
| chr16:50,223,371-51,223,371 | All | gtex.Skin_Sun_Exposed_Lower_leg | *SNX20* | 1407 | 0.000 | 0.718 | 0.000 | 0.105 | 0.177 | rs8063362 | 0.245 |
| chr16:50,223,371-51,223,371 | All | gtex.Skin_Not_Sun_Exposed_Suprapubic | *CYLD* | 1407 | 0.000 | 0.119 | 0.000 | 0.873 | 0.008 | rs8063362 | 0.201 |
| chr16:50,223,371-51,223,371 | All | gtex.Skin_Not_Sun_Exposed_Suprapubic | *SALL1* | 1407 | 0.000 | 0.858 | 0.000 | 0.096 | 0.046 | rs8063362 | 0.188 |
| chr16:50,223,371-51,223,371 | All | gtex.Skin_Not_Sun_Exposed_Suprapubic | *TENT4B* | 1360 | 0.000 | 0.820 | 0.000 | 0.129 | 0.051 | rs2287195 | 0.185 |
| chr16:50,223,371-51,223,371 | All | gtex.Skin_Not_Sun_Exposed_Suprapubic | *ADCY7* | 1407 | 0.000 | 0.767 | 0.000 | 0.181 | 0.051 | rs8063362 | 0.197 |
| chr16:50,223,371-51,223,371 | All | gtex.Skin_Not_Sun_Exposed_Suprapubic | *NKD1* | 1407 | 0.000 | 0.821 | 0.000 | 0.108 | 0.071 | rs8063362 | 0.191 |
| chr16:50,223,371-51,223,371 | All | gtex.Skin_Not_Sun_Exposed_Suprapubic | *BRD7* | 1407 | 0.000 | 0.633 | 0.000 | 0.088 | 0.279 | rs8063362 | 0.217 |
| chr16:50,223,371-51,223,371 | All | gtex.Skin_Not_Sun_Exposed_Suprapubic | *NOD2* | 1407 | 0.000 | 0.000 | 0.000 | 1.000 | 0.000 | rs8063362 | 0.193 |
| chr16:50,223,371-51,223,371 | All | gtex.Skin_Not_Sun_Exposed_Suprapubic | *SNX20* | 1407 | 0.000 | 0.814 | 0.000 | 0.129 | 0.057 | rs8063362 | 0.201 |
| chr6:146,420,271-147,420,271 | All | gtex.Whole_Blood | *RAB32* | 1522 | 0.000 | 0.807 | 0.000 | 0.148 | 0.044 | rs74468352 | 0.199 |
| chr6:146,420,271-147,420,271 | All | gtex.Whole_Blood | *STXBP5-AS1* | 1420 | 0.000 | 0.660 | 0.000 | 0.303 | 0.037 | rs75395649 | 0.211 |
| chr6:146,420,271-147,420,271 | All | gtex.Nerve_Tibial | *RAB32* | 1522 | 0.000 | 0.657 | 0.000 | 0.179 | 0.164 | rs74468352 | 0.237 |
| chr6:146,420,271-147,420,271 | All | gtex.Nerve_Tibial | *GRM1* | 1390 | 0.000 | 0.840 | 0.000 | 0.101 | 0.060 | rs74468352 | 0.204 |
| chr6:146,420,271-147,420,271 | All | gtex.Nerve_Tibial | *STXBP5-AS1* | 1420 | 0.000 | 0.817 | 0.000 | 0.117 | 0.066 | rs75395649 | 0.201 |
| chr6:146,420,271-147,420,271 | All | gtex.Skin_Sun_Exposed_Lower_leg | *ADGB* | 1522 | 0.000 | 0.808 | 0.000 | 0.117 | 0.075 | rs75395649 | 0.225 |
| chr6:146,420,271-147,420,271 | All | gtex.Skin_Sun_Exposed_Lower_leg | *RAB32* | 1522 | 0.000 | 0.758 | 0.000 | 0.095 | 0.146 | rs75395649 | 0.252 |
| chr6:146,420,271-147,420,271 | All | gtex.Skin_Sun_Exposed_Lower_leg | *STXBP5-AS1* | 1420 | 0.000 | 0.787 | 0.000 | 0.166 | 0.047 | rs74468352 | 0.193 |
| chr6:146,420,271-147,420,271 | All | gtex.Skin_Not_Sun_Exposed_Suprapubic | *ADGB* | 1522 | 0.000 | 0.803 | 0.000 | 0.107 | 0.090 | rs74468352 | 0.211 |
| chr6:146,420,271-147,420,271 | All | gtex.Skin_Not_Sun_Exposed_Suprapubic | *RAB32* | 1522 | 0.000 | 0.839 | 0.000 | 0.103 | 0.059 | rs74468352 | 0.198 |
| chr6:146,420,271-147,420,271 | All | gtex.Skin_Not_Sun_Exposed_Suprapubic | *STXBP5-AS1* | 1420 | 0.000 | 0.796 | 0.000 | 0.159 | 0.046 | rs75395649 | 0.209 |
| chr8:90,315,235-91,315,235 | All | gtex.Whole_Blood | *RIPK2* | 1492 | 0.000 | 0.830 | 0.000 | 0.111 | 0.059 | rs416324 | 0.079 |
| chr8:90,315,235-91,315,235 | All | gtex.Whole_Blood | *NBN* | 1492 | 0.000 | 0.025 | 0.000 | 0.961 | 0.013 | rs39504 | 0.083 |
| chr8:90,315,235-91,315,235 | All | gtex.Whole_Blood | *DECR1* | 1492 | 0.000 | 0.854 | 0.000 | 0.104 | 0.042 | rs416324 | 0.086 |
| chr8:90,315,235-91,315,235 | All | gtex.Whole_Blood | *CALB1* | 1492 | 0.000 | 0.855 | 0.000 | 0.101 | 0.044 | rs416324 | 0.089 |
| chr8:90,315,235-91,315,235 | All | gtex.Whole_Blood | *OSGIN2* | 1492 | 0.000 | 0.000 | 0.000 | 1.000 | 0.000 | rs416324 | 0.098 |
| chr8:90,315,235-91,315,235 | All | gtex.Whole_Blood | *LINC00534* | 1492 | 0.000 | 0.734 | 0.000 | 0.229 | 0.037 | rs416324 | 0.100 |
| chr8:90,315,235-91,315,235 | All | gtex.Nerve_Tibial | *RIPK2* | 1492 | 0.000 | 0.782 | 0.000 | 0.100 | 0.119 | rs416324 | 0.119 |
| chr8:90,315,235-91,315,235 | All | gtex.Nerve_Tibial | *NBN* | 1492 | 0.000 | 0.404 | 0.000 | 0.563 | 0.033 | rs39504 | 0.155 |
| chr8:90,315,235-91,315,235 | All | gtex.Nerve_Tibial | *DECR1* | 1492 | 0.000 | 0.671 | 0.000 | 0.296 | 0.033 | rs416324 | 0.087 |
| chr8:90,315,235-91,315,235 | All | gtex.Nerve_Tibial | *CALB1* | 1492 | 0.000 | 0.200 | 0.000 | 0.790 | 0.010 | rs416324 | 0.095 |
| chr8:90,315,235-91,315,235 | All | gtex.Nerve_Tibial | *OSGIN2* | 1492 | 0.000 | 0.826 | 0.000 | 0.132 | 0.042 | rs416324 | 0.088 |
| chr8:90,315,235-91,315,235 | All | gtex.Skin_Sun_Exposed_Lower_leg | *RIPK2* | 1492 | 0.000 | 0.061 | 0.000 | 0.191 | 0.748 | rs39504 | 0.179 |
| chr8:90,315,235-91,315,235 | All | gtex.Skin_Sun_Exposed_Lower_leg | *NBN* | 1492 | 0.000 | 0.840 | 0.000 | 0.117 | 0.043 | rs416324 | 0.096 |
| chr8:90,315,235-91,315,235 | All | gtex.Skin_Sun_Exposed_Lower_leg | *DECR1* | 1492 | 0.000 | 0.685 | 0.000 | 0.137 | 0.178 | rs39504 | 0.095 |
| chr8:90,315,235-91,315,235 | All | gtex.Skin_Sun_Exposed_Lower_leg | *CALB1* | 1492 | 0.000 | 0.710 | 0.000 | 0.256 | 0.035 | rs416324 | 0.087 |
| chr8:90,315,235-91,315,235 | All | gtex.Skin_Sun_Exposed_Lower_leg | *OSGIN2* | 1492 | 0.000 | 0.838 | 0.000 | 0.118 | 0.043 | rs416324 | 0.082 |
| chr8:90,315,235-91,315,235 | All | gtex.Skin_Not_Sun_Exposed_Suprapubic | *RIPK2* | 1492 | 0.000 | 0.858 | 0.000 | 0.093 | 0.049 | rs39504 | 0.095 |
| chr8:90,315,235-91,315,235 | All | gtex.Skin_Not_Sun_Exposed_Suprapubic | *NBN* | 1492 | 0.000 | 0.846 | 0.000 | 0.101 | 0.053 | rs416324 | 0.116 |
| chr8:90,315,235-91,315,235 | All | gtex.Skin_Not_Sun_Exposed_Suprapubic | *DECR1* | 1492 | 0.000 | 0.812 | 0.000 | 0.121 | 0.067 | rs416324 | 0.143 |
| chr8:90,315,235-91,315,235 | All | gtex.Skin_Not_Sun_Exposed_Suprapubic | *CALB1* | 1492 | 0.000 | 0.825 | 0.000 | 0.097 | 0.079 | rs416324 | 0.124 |
| chr8:90,315,235-91,315,235 | All | gtex.Skin_Not_Sun_Exposed_Suprapubic | *OSGIN2* | 1492 | 0.000 | 0.849 | 0.000 | 0.110 | 0.041 | rs416324 | 0.088 |
| chr10:72,610,977-73,610,977 | All | gtex.Whole_Blood | *UNC5B* | 2496 | 0.000 | 0.000 | 0.003 | 0.997 | 0.000 | rs780679 | 0.000 |
| chr10:72,610,977-73,610,977 | All | gtex.Whole_Blood | *CDH23* | 2496 | 0.000 | 0.012 | 0.003 | 0.985 | 0.001 | rs2487066 | 0.068 |
| chr10:72,610,977-73,610,977 | All | gtex.Whole_Blood | *SGPL1* | 2406 | 0.002 | 0.770 | 0.000 | 0.171 | 0.057 | rs809691 | 0.084 |
| chr10:72,610,977-73,610,977 | All | gtex.Whole_Blood | *SGPL1* | 2406 | 0.002 | 0.770 | 0.000 | 0.171 | 0.057 | rs703256 | 0.084 |
| chr10:72,610,977-73,610,977 | All | gtex.Whole_Blood | *PCBD1* | 2496 | 0.000 | 0.094 | 0.003 | 0.896 | 0.007 | rs780666 | 0.100 |
| chr10:72,610,977-73,610,977 | All | gtex.Whole_Blood | *PSAP* | 2496 | 0.000 | 0.056 | 0.003 | 0.938 | 0.003 | rs780666 | 0.089 |
| chr10:72,610,977-73,610,977 | All | gtex.Whole_Blood | *SLC29A3* | 2496 | 0.002 | 0.595 | 0.001 | 0.219 | 0.184 | rs2487066 | 0.109 |
| chr10:72,610,977-73,610,977 | All | gtex.Whole_Blood | *C10orf105* | 2496 | 0.002 | 0.787 | 0.000 | 0.170 | 0.041 | rs780666 | 0.080 |
| chr10:72,610,977-73,610,977 | All | gtex.Nerve_Tibial | *UNC5B* | 2496 | 0.002 | 0.767 | 0.001 | 0.191 | 0.039 | rs780666 | 0.074 |
| chr10:72,610,977-73,610,977 | All | gtex.Nerve_Tibial | *CDH23* | 2496 | 0.000 | 0.008 | 0.003 | 0.988 | 0.001 | rs780667 | 0.078 |
| chr10:72,610,977-73,610,977 | All | gtex.Nerve_Tibial | *SGPL1* | 2406 | 0.002 | 0.725 | 0.001 | 0.206 | 0.067 | rs2243485 | 0.084 |
| chr10:72,610,977-73,610,977 | All | gtex.Nerve_Tibial | *PCBD1* | 2496 | 0.000 | 0.000 | 0.003 | 0.997 | 0.000 | rs780666 | 0.000 |
| chr10:72,610,977-73,610,977 | All | gtex.Nerve_Tibial | *PSAP* | 2496 | 0.000 | 0.005 | 0.003 | 0.991 | 0.000 | rs2243485 | 0.071 |
| chr10:72,610,977-73,610,977 | All | gtex.Nerve_Tibial | *SLC29A3* | 2496 | 0.000 | 0.000 | 0.003 | 0.997 | 0.000 | rs2487066 | 0.000 |
| chr10:72,610,977-73,610,977 | All | gtex.Nerve_Tibial | *C10orf105* | 2496 | 0.002 | 0.757 | 0.001 | 0.202 | 0.039 | rs780666 | 0.073 |
| chr10:72,610,977-73,610,977 | All | gtex.Nerve_Tibial | *UNC5B-AS1* | 2496 | 0.002 | 0.769 | 0.000 | 0.173 | 0.056 | rs780666 | 0.094 |
| chr10:72,610,977-73,610,977 | All | gtex.Skin_Sun_Exposed_Lower_leg | *UNC5B* | 2496 | 0.002 | 0.775 | 0.001 | 0.181 | 0.041 | rs2487066 | 0.080 |
| chr10:72,610,977-73,610,977 | All | gtex.Skin_Sun_Exposed_Lower_leg | *CDH23* | 2496 | 0.001 | 0.515 | 0.001 | 0.456 | 0.026 | rs780666 | 0.073 |
| chr10:72,610,977-73,610,977 | All | gtex.Skin_Sun_Exposed_Lower_leg | *SGPL1* | 2406 | 0.001 | 0.494 | 0.001 | 0.449 | 0.054 | rs2487066 | 0.152 |
| chr10:72,610,977-73,610,977 | All | gtex.Skin_Sun_Exposed_Lower_leg | *PCBD1* | 2496 | 0.002 | 0.538 | 0.001 | 0.430 | 0.030 | rs2243485 | 0.078 |
| chr10:72,610,977-73,610,977 | All | gtex.Skin_Sun_Exposed_Lower_leg | *PSAP* | 2496 | 0.002 | 0.786 | 0.000 | 0.169 | 0.043 | rs780666 | 0.074 |
| chr10:72,610,977-73,610,977 | All | gtex.Skin_Sun_Exposed_Lower_leg | *SLC29A3* | 2496 | 0.000 | 0.003 | 0.003 | 0.994 | 0.000 | rs2487066 | 0.079 |
| chr10:72,610,977-73,610,977 | All | gtex.Skin_Sun_Exposed_Lower_leg | *C10orf105* | 2496 | 0.002 | 0.788 | 0.000 | 0.170 | 0.040 | rs780666 | 0.080 |
| chr10:72,610,977-73,610,977 | All | gtex.Skin_Sun_Exposed_Lower_leg | *UNC5B-AS1* | 2496 | 0.002 | 0.759 | 0.001 | 0.183 | 0.056 | rs780666 | 0.093 |
| chr10:72,610,977-73,610,977 | All | gtex.Skin_Not_Sun_Exposed_Suprapubic | *UNC5B* | 2496 | 0.002 | 0.671 | 0.000 | 0.172 | 0.154 | rs780666 | 0.105 |
| chr10:72,610,977-73,610,977 | All | gtex.Skin_Not_Sun_Exposed_Suprapubic | *CDH23* | 2496 | 0.002 | 0.641 | 0.001 | 0.315 | 0.042 | rs2249989 | 0.108 |
| chr10:72,610,977-73,610,977 | All | gtex.Skin_Not_Sun_Exposed_Suprapubic | *SGPL1* | 2406 | 0.001 | 0.315 | 0.002 | 0.665 | 0.018 | rs780666 | 0.090 |
| chr10:72,610,977-73,610,977 | All | gtex.Skin_Not_Sun_Exposed_Suprapubic | *PCBD1* | 2496 | 0.002 | 0.697 | 0.001 | 0.262 | 0.039 | rs2487066 | 0.107 |
| chr10:72,610,977-73,610,977 | All | gtex.Skin_Not_Sun_Exposed_Suprapubic | *PSAP* | 2496 | 0.002 | 0.706 | 0.000 | 0.164 | 0.128 | rs809691 | 0.173 |
| chr10:72,610,977-73,610,977 | All | gtex.Skin_Not_Sun_Exposed_Suprapubic | *PSAP* | 2496 | 0.002 | 0.706 | 0.000 | 0.164 | 0.128 | rs703256 | 0.173 |
| chr10:72,610,977-73,610,977 | All | gtex.Skin_Not_Sun_Exposed_Suprapubic | *SLC29A3* | 2496 | 0.000 | 0.006 | 0.003 | 0.990 | 0.000 | rs2243485 | 0.082 |
| chr10:72,610,977-73,610,977 | All | gtex.Skin_Not_Sun_Exposed_Suprapubic | *C10orf105* | 2496 | 0.002 | 0.784 | 0.000 | 0.166 | 0.048 | rs2487066 | 0.080 |
| chr10:72,610,977-73,610,977 | All | gtex.Skin_Not_Sun_Exposed_Suprapubic | *UNC5B-AS1* | 2496 | 0.002 | 0.786 | 0.000 | 0.171 | 0.039 | rs780666 | 0.074 |
| chr9:117,079,504-118,140,404 | First | gtex.Whole_Blood | *ORM2* | 1819 | 0.000 | 0.012 | 0.000 | 0.987 | 0.001 | rs10817678 | 0.613 |
| chr9:117,079,504-118,140,404 | First | gtex.Whole_Blood | *ORM1* | 1819 | 0.000 | 0.141 | 0.000 | 0.851 | 0.008 | rs10817678 | 0.612 |
| chr9:117,079,504-118,140,404 | First | gtex.Whole_Blood | *TNC* | 1819 | 0.000 | 0.746 | 0.000 | 0.158 | 0.096 | rs10817678 | 0.511 |
| chr9:117,079,504-118,140,404 | First | gtex.Whole_Blood | *WHRN* | 1819 | 0.000 | 0.000 | 0.000 | 1.000 | 0.000 | rs10817678 | 0.604 |
| chr9:117,079,504-118,140,404 | First | gtex.Whole_Blood | *AKNA* | 1819 | 0.000 | 0.609 | 0.000 | 0.361 | 0.030 | rs10817678 | 0.595 |
| chr9:117,079,504-118,140,404 | First | gtex.Whole_Blood | *TNFSF8* | 1819 | 0.000 | 0.316 | 0.000 | 0.335 | 0.349 | rs10817678 | 0.542 |
| chr9:117,079,504-118,140,404 | First | gtex.Whole_Blood | *ATP6V1G1* | 1819 | 0.000 | 0.699 | 0.000 | 0.251 | 0.051 | rs10817678 | 0.573 |
| chr9:117,079,504-118,140,404 | First | gtex.Whole_Blood | *TNFSF15* | 1819 | 0.000 | 0.000 | 0.000 | 0.006 | **0.994** | rs10817678 | 0.865 |
| chr9:117,079,504-118,140,404 | First | gtex.Nerve_Tibial | *ORM2* | 1819 | 0.000 | 0.791 | 0.000 | 0.170 | 0.039 | rs10817678 | 0.604 |
| chr9:117,079,504-118,140,404 | First | gtex.Nerve_Tibial | *ORM1* | 1819 | 0.000 | 0.809 | 0.000 | 0.138 | 0.053 | rs10817678 | 0.630 |
| chr9:117,079,504-118,140,404 | First | gtex.Nerve_Tibial | *TNC* | 1819 | 0.000 | 0.236 | 0.000 | 0.752 | 0.012 | rs10817678 | 0.610 |
| chr9:117,079,504-118,140,404 | First | gtex.Nerve_Tibial | *WHRN* | 1819 | 0.000 | 0.103 | 0.000 | 0.892 | 0.005 | rs10817678 | 0.605 |
| chr9:117,079,504-118,140,404 | First | gtex.Nerve_Tibial | *AKNA* | 1819 | 0.000 | 0.798 | 0.000 | 0.148 | 0.055 | rs10817678 | 0.610 |
| chr9:117,079,504-118,140,404 | First | gtex.Nerve_Tibial | *TNFSF8* | 1819 | 0.000 | 0.717 | 0.000 | 0.123 | 0.161 | rs10817678 | 0.569 |
| chr9:117,079,504-118,140,404 | First | gtex.Nerve_Tibial | *ATP6V1G1* | 1819 | 0.000 | 0.806 | 0.000 | 0.134 | 0.059 | rs10817678 | 0.587 |
| chr9:117,079,504-118,140,404 | First | gtex.Nerve_Tibial | *DELEC1* | 1819 | 0.000 | 0.000 | 0.000 | 1.000 | 0.000 | rs10817678 | 0.661 |
| chr9:117,079,504-118,140,404 | First | gtex.Nerve_Tibial | *TNFSF15* | 1819 | 0.000 | 0.815 | 0.000 | 0.127 | 0.058 | rs10817678 | 0.578 |
| chr9:117,079,504-118,140,404 | First | gtex.Skin_Sun_Exposed_Lower_leg | *ORM2* | 1819 | 0.000 | 0.844 | 0.000 | 0.115 | 0.041 | rs10817678 | 0.601 |
| chr9:117,079,504-118,140,404 | First | gtex.Skin_Sun_Exposed_Lower_leg | *ORM1* | 1819 | 0.000 | 0.811 | 0.000 | 0.128 | 0.061 | rs10817678 | 0.641 |
| chr9:117,079,504-118,140,404 | First | gtex.Skin_Sun_Exposed_Lower_leg | *TNC* | 1819 | 0.000 | 0.823 | 0.000 | 0.131 | 0.046 | rs10817678 | 0.608 |
| chr9:117,079,504-118,140,404 | First | gtex.Skin_Sun_Exposed_Lower_leg | *WHRN* | 1819 | 0.000 | 0.002 | 0.000 | 0.998 | 0.000 | rs10817678 | 0.606 |
| chr9:117,079,504-118,140,404 | First | gtex.Skin_Sun_Exposed_Lower_leg | *AKNA* | 1819 | 0.000 | 0.195 | 0.000 | 0.795 | 0.009 | rs10817678 | 0.596 |
| chr9:117,079,504-118,140,404 | First | gtex.Skin_Sun_Exposed_Lower_leg | *TNFSF8* | 1819 | 0.000 | 0.827 | 0.000 | 0.130 | 0.044 | rs10817678 | 0.585 |
| chr9:117,079,504-118,140,404 | First | gtex.Skin_Sun_Exposed_Lower_leg | *ATP6V1G1* | 1819 | 0.000 | 0.729 | 0.000 | 0.220 | 0.050 | rs10817678 | 0.643 |
| chr9:117,079,504-118,140,404 | First | gtex.Skin_Sun_Exposed_Lower_leg | *TNFSF15* | 1819 | 0.000 | 0.768 | 0.000 | 0.127 | 0.105 | rs10817678 | 0.603 |
| chr9:117,079,504-118,140,404 | First | gtex.Skin_Not_Sun_Exposed_Suprapubic | *ORM2* | 1819 | 0.000 | 0.791 | 0.000 | 0.169 | 0.040 | rs10817678 | 0.612 |
| chr9:117,079,504-118,140,404 | First | gtex.Skin_Not_Sun_Exposed_Suprapubic | *ORM1* | 1819 | 0.000 | 0.798 | 0.000 | 0.130 | 0.072 | rs10817678 | 0.607 |
| chr9:117,079,504-118,140,404 | First | gtex.Skin_Not_Sun_Exposed_Suprapubic | *TNC* | 1819 | 0.000 | 0.788 | 0.000 | 0.173 | 0.038 | rs10817678 | 0.603 |
| chr9:117,079,504-118,140,404 | First | gtex.Skin_Not_Sun_Exposed_Suprapubic | *WHRN* | 1819 | 0.000 | 0.006 | 0.000 | 0.994 | 0.000 | rs10817678 | 0.607 |
| chr9:117,079,504-118,140,404 | First | gtex.Skin_Not_Sun_Exposed_Suprapubic | *AKNA* | 1819 | 0.000 | 0.278 | 0.000 | 0.673 | 0.049 | rs10817678 | 0.585 |
| chr9:117,079,504-118,140,404 | First | gtex.Skin_Not_Sun_Exposed_Suprapubic | *TNFSF8* | 1819 | 0.000 | 0.833 | 0.000 | 0.126 | 0.040 | rs10817678 | 0.599 |
| chr9:117,079,504-118,140,404 | First | gtex.Skin_Not_Sun_Exposed_Suprapubic | *ATP6V1G1* | 1819 | 0.000 | 0.797 | 0.000 | 0.149 | 0.054 | rs10817678 | 0.588 |
| chr9:117,079,504-118,140,404 | First | gtex.Skin_Not_Sun_Exposed_Suprapubic | *DELEC1* | 1819 | 0.000 | 0.789 | 0.000 | 0.152 | 0.059 | rs10817678 | 0.600 |
| chr9:117,079,504-118,140,404 | First | gtex.Skin_Not_Sun_Exposed_Suprapubic | *TNFSF15* | 1819 | 0.000 | 0.808 | 0.000 | 0.137 | 0.055 | rs10817678 | 0.557 |
| chr9:117,079,504-118,140,404 | Second | gtex.Whole_Blood | *TNC* | 1904 | 0.000 | 0.765 | 0.000 | 0.176 | 0.058 | . | . |
| chr9:117,079,504-118,140,404 | Second | gtex.Whole_Blood | *WHRN* | 1904 | 0.000 | 0.000 | 0.000 | 1.000 | 0.000 | . | . |
| chr9:117,079,504-118,140,404 | Second | gtex.Whole_Blood | *AKNA* | 1904 | 0.000 | 0.737 | 0.000 | 0.180 | 0.083 | . | . |
| chr9:117,079,504-118,140,404 | Second | gtex.Whole_Blood | *TNFSF8* | 1904 | 0.000 | 0.487 | 0.000 | 0.485 | 0.029 | . | . |
| chr9:117,079,504-118,140,404 | Second | gtex.Whole_Blood | *ATP6V1G1* | 1904 | 0.000 | 0.686 | 0.000 | 0.249 | 0.065 | . | . |
| chr9:117,079,504-118,140,404 | Second | gtex.Whole_Blood | *TNFSF15* | 1904 | 0.000 | 0.396 | 0.000 | 0.189 | 0.415 | . | . |
| chr9:117,079,504-118,140,404 | Second | gtex.Whole_Blood | *ORM2* | 1715 | 0.000 | 0.647 | 0.000 | 0.317 | 0.036 | . | . |
| chr9:117,079,504-118,140,404 | Second | gtex.Whole_Blood | *ORM1* | 1701 | 0.000 | 0.825 | 0.000 | 0.133 | 0.042 | . | . |
| chr9:117,079,504-118,140,404 | Second | gtex.Nerve_Tibial | *ORM2* | 1715 | 0.000 | 0.622 | 0.000 | 0.119 | 0.258 | . | . |
| chr9:117,079,504-118,140,404 | Second | gtex.Nerve_Tibial | *ORM1* | 1701 | 0.000 | 0.677 | 0.000 | 0.104 | 0.219 | . | . |
| chr9:117,079,504-118,140,404 | Second | gtex.Nerve_Tibial | *TNC* | 1904 | 0.000 | 0.139 | 0.000 | 0.853 | 0.008 | . | . |
| chr9:117,079,504-118,140,404 | Second | gtex.Nerve_Tibial | *WHRN* | 1904 | 0.000 | 0.103 | 0.000 | 0.891 | 0.006 | . | . |
| chr9:117,079,504-118,140,404 | Second | gtex.Nerve_Tibial | *AKNA* | 1904 | 0.000 | 0.811 | 0.000 | 0.147 | 0.042 | . | . |
| chr9:117,079,504-118,140,404 | Second | gtex.Nerve_Tibial | *TNFSF8* | 1904 | 0.000 | 0.802 | 0.000 | 0.145 | 0.053 | . | . |
| chr9:117,079,504-118,140,404 | Second | gtex.Nerve_Tibial | *ATP6V1G1* | 1904 | 0.000 | 0.808 | 0.000 | 0.141 | 0.050 | . | . |
| chr9:117,079,504-118,140,404 | Second | gtex.Nerve_Tibial | *DELEC1* | 1904 | 0.000 | 0.000 | 0.000 | 1.000 | 0.000 | . | . |
| chr9:117,079,504-118,140,404 | Second | gtex.Nerve_Tibial | *TNFSF15* | 1904 | 0.000 | 0.829 | 0.000 | 0.129 | 0.043 | . | . |
| chr9:117,079,504-118,140,404 | Second | gtex.Skin_Sun_Exposed_Lower_leg | *TNC* | 1904 | 0.000 | 0.786 | 0.000 | 0.174 | 0.040 | . | . |
| chr9:117,079,504-118,140,404 | Second | gtex.Skin_Sun_Exposed_Lower_leg | *WHRN* | 1904 | 0.000 | 0.002 | 0.000 | 0.998 | 0.000 | . | . |
| chr9:117,079,504-118,140,404 | Second | gtex.Skin_Sun_Exposed_Lower_leg | *AKNA* | 1904 | 0.000 | 0.195 | 0.000 | 0.794 | 0.010 | . | . |
| chr9:117,079,504-118,140,404 | Second | gtex.Skin_Sun_Exposed_Lower_leg | *TNFSF8* | 1904 | 0.000 | 0.800 | 0.000 | 0.130 | 0.070 | . | . |
| chr9:117,079,504-118,140,404 | Second | gtex.Skin_Sun_Exposed_Lower_leg | *ATP6V1G1* | 1904 | 0.000 | 0.726 | 0.000 | 0.223 | 0.051 | . | . |
| chr9:117,079,504-118,140,404 | Second | gtex.Skin_Sun_Exposed_Lower_leg | *TNFSF15* | 1904 | 0.000 | 0.822 | 0.000 | 0.135 | 0.044 | . | . |
| chr9:117,079,504-118,140,404 | Second | gtex.Skin_Sun_Exposed_Lower_leg | *ORM2* | 1715 | 0.000 | 0.844 | 0.000 | 0.108 | 0.048 | . | . |
| chr9:117,079,504-118,140,404 | Second | gtex.Skin_Sun_Exposed_Lower_leg | *ORM1* | 1701 | 0.000 | 0.792 | 0.000 | 0.114 | 0.094 | . | . |
| chr9:117,079,504-118,140,404 | Second | gtex.Skin_Not_Sun_Exposed_Suprapubic | *ORM2* | 1715 | 0.000 | 0.794 | 0.000 | 0.165 | 0.041 | . | . |
| chr9:117,079,504-118,140,404 | Second | gtex.Skin_Not_Sun_Exposed_Suprapubic | *ORM1* | 1701 | 0.000 | 0.827 | 0.000 | 0.128 | 0.044 | . | . |
| chr9:117,079,504-118,140,404 | Second | gtex.Skin_Not_Sun_Exposed_Suprapubic | *TNC* | 1904 | 0.000 | 0.658 | 0.000 | 0.150 | 0.192 | . | . |
| chr9:117,079,504-118,140,404 | Second | gtex.Skin_Not_Sun_Exposed_Suprapubic | *WHRN* | 1904 | 0.000 | 0.006 | 0.000 | 0.994 | 0.000 | . | . |
| chr9:117,079,504-118,140,404 | Second | gtex.Skin_Not_Sun_Exposed_Suprapubic | *AKNA* | 1904 | 0.000 | 0.287 | 0.000 | 0.696 | 0.017 | . | . |
| chr9:117,079,504-118,140,404 | Second | gtex.Skin_Not_Sun_Exposed_Suprapubic | *TNFSF8* | 1904 | 0.000 | 0.832 | 0.000 | 0.123 | 0.044 | . | . |
| chr9:117,079,504-118,140,404 | Second | gtex.Skin_Not_Sun_Exposed_Suprapubic | *ATP6V1G1* | 1904 | 0.000 | 0.770 | 0.000 | 0.155 | 0.074 | . | . |
| chr9:117,079,504-118,140,404 | Second | gtex.Skin_Not_Sun_Exposed_Suprapubic | *DELEC1* | 1904 | 0.000 | 0.794 | 0.000 | 0.162 | 0.044 | . | . |
| chr9:117,079,504-118,140,404 | Second | gtex.Skin_Not_Sun_Exposed_Suprapubic | *TNFSF15* | 1904 | 0.000 | 0.781 | 0.000 | 0.137 | 0.082 | . | . |

Signal, Which GWAS signal was tested;

First, the signal before conditional;Second, the conditional signal;

PP.H0.abf, H0 probability from coloc; PP.H1.abf, H1 probability from coloc;

PP.H2.abf, H2 probability from coloc; PP.H3.abf, H3 probability from coloc;

PP.H4.abf, H4 probability from coloc;

Top Coloc SNP, the most likely SNP which GWAS and eQTL signals colocalization.;

PP of SNP, the posterior probability (PP) of the GWAS and eQTL signals to have the same underlying this SNP.

., not applicable.

Table S7. Protein altering variants in the 95% credible sets of genome-wide significant loci.

| **Locus** | **SNP** | **CHR:BP** | **MA** | **P** | **OR** | **r^2^** | **gene** | **Function** | **PIP** | **CADD** | **SpliceAI_max** |
| --- | --- | --- | --- | --- | --- | --- | --- | --- | --- | --- | --- |
| chr10:63,991,638 - 64,994,157 | rs11599754 | 10:64466651 | T | 1.57E-10 | 0.77 | 0.99 | 35kb 3' of ZNF365 | . | 0.04 | **15.8** | . |
| chr10:63,991,638 - 64,994,157 | rs76510618 | 10:64528725 | C | 3.67E-08 | 1.21 | 0.33 | 36kb 5' of ADO | . | 0.04 | **15.4** | . |
| chr10:63,991,638 - 64,994,157 | rs2236295 | 10:64564892 | G | 4.93E-10 | 0.78 | 0.53 | ADO | missense | 0.02 | **23.6** | 0 |
| chr10:63,991,638 - 64,994,157 | rs10995311 | 10:64564934 | C | 1.07E-10 | 0.78 | 0.56 | ADO | missense | 0.06 | 14.3 | 0 |
| chr10:72,610,977 - 73,610,977 | rs780668 | 10:73111408 | C | 7.57E-08 | 1.14 | 1.00 | SLC29A3 | missense | 0.05 | **23.5** | 0 |
| chr10:72,610,977 - 73,610,977 | rs780675 | 10:73113790 | C | 1.58E-07 | 1.14 | 0.98 | SLC29A3 | intronic | 0.03 | **15.5** | 0 |
| chr12:57,665,085 - 58,665,085 | rs2014886 | 12:58177437 | T | 1.59E-08 | 0.86 | 0.91 | TSFM | intronic | 0.01 | 9.5 | **0.82** |
| chr13:43,959,499 - 45,012,908 | rs3764147 | 13:44457925 | A | 4.12E-81 | 1.63 | 1.00 | LACC1 | missense | 0.49 | 12.5 | 0 |
| chr16:28,008,048 - 29,008,048 | rs180743 | 16:28507644 | C | 8.24E-11 | 0.78 | 1.00 | APOBR | missense | 0.10 | 9.2 | 0.07 |
| chr16:28,008,048 - 29,008,048 | rs181206 | 16:28513403 | A | 3.57E-10 | 0.78 | 0.94 | IL27 | missense | 0.02 | **22.4** | 0 |
| chr16:28,008,048 - 29,008,048 | rs1059491 | 16:28603655 | T | 1.56E-09 | 0.78 | 0.77 | SULT1A2 | missense | 0.01 | **24.7** | 0 |

CHR:BP, Chromosome and position, position based on hg19 coordinates;

MA, minor allele;

r^2^, r^2^ with the lead SNP in all GWAS samples;

Function, SNP funcition annotation from HaploReg v4.1(<https://pubs.broadinstitute.org/mammals/haploreg/haploreg.php>);

PIP, the posterior inclusion probability (PIP) that each SNP was causal by SusieR R package;

CADD, the score value from CADD tool (CADD score >15 was defined as deleterious;

SpliceAI_max, the max score value from SpliceAI tool (score > 0.8 indicated that this variant has a high accuracy affecting the splicing).

., not applicable.

**Table S8. Summary of top10 pathways in pathway analysis as implemented in MAGMA.**

| A) The 10 most significant GO terms. |  |  |  |  |  |
| --- | --- | --- | --- | --- | --- |
| Pathway name | Ngenes | BETA | BETA_STD | SE | P |
| **GOMF_MHC_CLASS_II_RECEPTOR_ACTIVITY** | **9** | **2.00** | **0.05** | **0.36** | **2.16E-08** |
| **GOBP_PEPTIDE_ANTIGEN_ASSEMBLY_WITH_MHC_CLASS_II_PROTEIN_COMPLEX** | **12** | **1.49** | **0.04** | **0.31** | **7.77E-07** |
| **GOCC_MHC_CLASS_II_PROTEIN_COMPLEX** | **13** | **1.34** | **0.04** | **0.29** | **2.21E-06** |
| GOBP_POSITIVE_REGULATION_OF_CELL_CELL_ADHESION | 265 | 0.26 | 0.03 | 0.06 | 8.00E-06 |
| GOBP_REGULATION_OF_ADIPONECTIN_SECRETION | 6 | 1.34 | 0.03 | 0.32 | 1.08E-05 |
| GOBP_POSITIVE_REGULATION_OF_LEUKOCYTE_CELL_CELL_ADHESION | 222 | 0.27 | 0.03 | 0.07 | 1.64E-05 |
| **GOMF_IMMUNE_RECEPTOR_ACTIVITY** | **122** | **0.37** | **0.03** | **0.09** | **1.65E-05** |
| GOBP_CD4_POSITIVE_ALPHA_BETA_T_CELL_DIFFERENTIATION | 78 | 0.44 | 0.03 | 0.11 | 3.06E-05 |
| GOCC_MHC_PROTEIN_COMPLEX | 21 | 0.83 | 0.03 | 0.21 | 3.98E-05 |
| GOBP_ALPHA_BETA_T_CELL_DIFFERENTIATION | 106 | 0.37 | 0.03 | 0.09 | 5.04E-05 |
|  |  |  |  |  |  |
| B) The 10 most significant KEGG terms. |  |  |  |  |  |
| Pathway name | Ngenes | BETA | BETA_STD | SE | P |
| **KEGG_LEISHMANIA_INFECTION** | **62** | **0.55** | **0.03** | **0.12** | **2.52E-06** |
| **KEGG_GRAFT_VERSUS_HOST_DISEASE** | **33** | **0.75** | **0.03** | **0.17** | **3.96E-06** |
| **KEGG_ALLOGRAFT_REJECTION** | **33** | **0.65** | **0.03** | **0.17** | **7.07E-05** |
| **KEGG_AUTOIMMUNE_THYROID_DISEASE** | **45** | **0.55** | **0.03** | **0.16** | **2.27E-04** |
| KEGG_TYPE_I_DIABETES_MELLITUS | 40 | 0.51 | 0.02 | 0.16 | 5.50E-04 |
| KEGG_SYSTEMIC_LUPUS_ERYTHEMATOSUS | 93 | 0.33 | 0.02 | 0.11 | 8.77E-04 |
| KEGG_ASTHMA | 26 | 0.58 | 0.02 | 0.19 | 1.00E-03 |
| KEGG_CELL_ADHESION_MOLECULES_CAMS | 119 | 0.24 | 0.02 | 0.09 | 3.01E-03 |
| KEGG_CELL_CYCLE | 114 | 0.20 | 0.02 | 0.08 | 5.92E-03 |
| KEGG_CYTOKINE_CYTOKINE_RECEPTOR_INTERACTION | 232 | 0.16 | 0.02 | 0.07 | 6.64E-03 |
|  |  |  |  |  |  |
| C) The 10 most significant REACTOME terms. |  |  |  |  |  |
| Pathway name | Ngenes | BETA | BETA_STD | SE | P |
| **REACTOME_PD_1_SIGNALING** | **19** | **0.96** | **0.03** | **0.23** | **1.35E-05** |
| REACTOME_OVARIAN_TUMOR_DOMAIN_PROTEASES | 35 | 0.62 | 0.03 | 0.15 | 2.34E-05 |
| REACTOME_RNA_POLYMERASE_II_TRANSCRIPTION | 1233 | 0.10 | 0.03 | 0.03 | 7.30E-05 |
| REACTOME_INTERFERON_ALPHA_BETA_SIGNALING | 61 | 0.44 | 0.03 | 0.12 | 9.12E-05 |
| REACTOME_P75NTR_RECRUITS_SIGNALLING_COMPLEXES | 12 | 1.08 | 0.03 | 0.32 | 3.15E-04 |
| **REACTOME_INTERFERON_GAMMA_SIGNALING** | **84** | **0.35** | **0.02** | **0.11** | **4.00E-04** |
| REACTOME_NEGATIVE_REGULATION_OF_FLT3 | 15 | 0.87 | 0.03 | 0.26 | 4.14E-04 |
| REACTOME_RESOLUTION_OF_D_LOOP_STRUCTURES | 32 | 0.52 | 0.02 | 0.15 | 4.23E-04 |
| REACTOME_SIGNALING_BY_NOTCH1_T_7_9_NOTCH1_M1580_K2555_TRANSLOCATION_MUTANT | 6 | 1.04 | 0.02 | 0.31 | 4.63E-04 |
| REACTOME_HOMOLOGY_DIRECTED_REPAIR | 122 | 0.24 | 0.02 | 0.07 | 5.15E-04 |

Table S9. Tissues and cell types enrichment analysis (P < 0.05) based on specifically expressed genes.

| Name | Coefficient_P_value | Tissue category for display | -log10Pvalue |
| --- | --- | --- | --- |
| **A02.835.583.443.800.800.Synovial.Fluid** | **1.36E-03** | **Blood/Immune** | **2.87** |
| **A15.145.Blood** | **1.67E-03** | **Blood/Immune** | **2.78** |
| A15.378.316.Bone.Marrow.Cells | 2.03E-03 | Blood/Immune | 2.69 |
| A15.382.680.Phagocytes | 2.20E-03 | Blood/Immune | 2.66 |
| A15.382.812.260.Dendritic.Cells | 3.19E-03 | Blood/Immune | 2.50 |
| A15.145.229.Blood.Cells | 4.12E-03 | Blood/Immune | 2.39 |
| A15.378.316.580.Monocytes | 7.49E-03 | Blood/Immune | 2.13 |
| A02.165.Cartilage | 8.11E-03 | Musculoskeletal/Connective | 2.09 |
| A11.118.637.Leukocytes | 8.24E-03 | Blood/Immune | 2.08 |
| A15.382.490.315.583.Neutrophils | 1.05E-02 | Blood/Immune | 1.98 |
| A06.407.900.Thyroid.Gland | 1.31E-02 | Endocrine | 1.88 |
| A15.382.812.522.Macrophages | 1.38E-02 | Blood/Immune | 1.86 |
| A15.382.812.Mononuclear.Phagocyte.System | 1.45E-02 | Blood/Immune | 1.84 |
| A02.835.583.443.800.Synovial.Membrane | 1.95E-02 | Musculoskeletal/Connective | 1.71 |
| A05.360.319.679.690.Myometrium | 2.30E-02 | Musculoskeletal/Connective | 1.64 |
| A11.443.Erythroid.Cells | 3.67E-02 | Blood/Immune | 1.44 |
| A15.145.300.Fetal.Blood | 4.23E-02 | Blood/Immune | 1.37 |
| A11.872.378.590.635.Granulocyte.Macrophage.Progenitor.Cells | 4.36E-02 | Blood/Immune | 1.36 |
| A15.382.Immune.System | 4.57E-02 | Blood/Immune | 1.34 |
| A11.872.653.Neural.Stem.Cells | 4.63E-02 | CNS | 1.33 |

Table S10. Tissues and cell types enrichment analysis results (P < 0.05) based on the peaks of chromatin mark.

| Tissue/cell type | Mark | Coefficient_P_value | Tissue category for figures | -log10Pvalue |
| --- | --- | --- | --- | --- |
| **Primary_monocytes_from_peripheral_blood** | **DNase** | **1.74E-04** | **Blood/Immune** | **3.76** |
| **Primary_T_helper_17_cells_PMA-I_stimulated** | **H3K4me1** | **6.11E-04** | **Blood/Immune** | **3.21** |
| **Primary_hematopoietic_stem_cells_short_term_culture** | **H3K4me1** | **6.67E-04** | **Blood/Immune** | **3.18** |
| **Primary_T_helper_17_cells_PMA-I_stimulated** | **H3K4me3** | **7.75E-04** | **Blood/Immune** | **3.11** |
| **Primary_Natural_Killer_cells_from_peripheral_blood** | **DNase** | **1.23E-03** | **Blood/Immune** | **2.91** |
| Primary_T_helper_17_cells_PMA-I_stimulated | H3K27ac | 1.93E-03 | Blood/Immune | 2.71 |
| Primary_T_helper_cells_from_peripheral_blood | H3K4me1 | 2.13E-03 | Blood/Immune | 2.67 |
| Primary_T_helper_cells_PMA-I_stimulated | H3K4me1 | 2.14E-03 | Blood/Immune | 2.67 |
| Primary_T_cells_from_cord_blood | DNase | 2.19E-03 | Blood/Immune | 2.66 |
| Primary_T_helper_cells_PMA-I_stimulated | H3K4me3 | 2.73E-03 | Blood/Immune | 2.56 |
| Primary_T_helper_memory_cells_from_peripheral_blood_2 | H3K4me1 | 3.27E-03 | Blood/Immune | 2.49 |
| Primary_neutrophils_from_peripheral_blood | H3K4me3 | 3.37E-03 | Blood/Immune | 2.47 |
| Colonic_Mucosa | H3K9ac | 3.47E-03 | Digestive | 2.46 |
| Spleen | H3K4me3 | 3.51E-03 | Blood/Immune | 2.45 |
| Primary_monocytes_from_peripheral_blood | H3K4me1 | 4.94E-03 | Blood/Immune | 2.31 |
| Primary_T_helper_memory_cells_from_peripheral_blood_1 | H3K4me1 | 6.31E-03 | Blood/Immune | 2.20 |
| Primary_mononuclear_cells_from_peripheral_blood | H3K4me3 | 6.75E-03 | Blood/Immune | 2.17 |
| Primary_hematopoietic_stem_cells_short_term_culture | H3K4me3 | 7.42E-03 | Blood/Immune | 2.13 |
| Colon_Smooth_Muscle | H3K36me3 | 7.52E-03 | Musculoskeletal/Connective | 2.12 |
| Primary_T_helper_cells_PMA-I_stimulated | H3K27ac | 7.57E-03 | Blood/Immune | 2.12 |
| Primary_T_killer_memory_cells_from_peripheral_blood | H3K4me1 | 7.85E-03 | Blood/Immune | 2.10 |
| Primary_T_helper_naive_cells_from_peripheral_blood_1 | H3K4me1 | 9.89E-03 | Blood/Immune | 2.00 |
| Primary_T_cells_effector_memory_enriched_from_peripheral_blood | H3K4me1 | 1.01E-02 | Blood/Immune | 1.99 |
| Colon-TV | H3K4me3 | 1.05E-02 | Digestive | 1.98 |
| Spleen | H3K4me1 | 1.06E-02 | Blood/Immune | 1.97 |
| Primary_B_cells_from_peripheral_blood | DNase | 1.15E-02 | Blood/Immune | 1.94 |
| Colonic_Mucosa | H3K4me3 | 1.16E-02 | Digestive | 1.93 |
| Primary_Natural_Killer_cells_from_peripheral_blood | H3K4me3 | 1.17E-02 | Blood/Immune | 1.93 |
| Primary_T_cells_from_peripheral_blood | DNase | 1.22E-02 | Blood/Immune | 1.91 |
| Primary_T_cells_from_peripheral_blood | H3K4me3 | 1.26E-02 | Blood/Immune | 1.90 |
| Lung | H3K36me3 | 1.45E-02 | Other | 1.84 |
| Primary_T_regulatory_cells_from_peripheral_blood | H3K4me1 | 1.61E-02 | Blood/Immune | 1.79 |
| Spleen | H3K27ac | 1.75E-02 | Blood/Immune | 1.76 |
| Primary_monocytes_from_peripheral_blood | H3K36me3 | 1.88E-02 | Blood/Immune | 1.73 |
| Primary_hematopoietic_stem_cells_G-CSF-mobilized_Female | DNase | 1.95E-02 | Blood/Immune | 1.71 |
| Primary_T_cells_from_peripheral_blood | H3K27ac | 1.99E-02 | Blood/Immune | 1.70 |
| Primary_neutrophils_from_peripheral_blood | H3K4me1 | 2.04E-02 | Blood/Immune | 1.69 |
| Primary_monocytes_from_peripheral_blood | H3K4me3 | 2.05E-02 | Blood/Immune | 1.69 |
| Stomach | H3K4me3 | 2.10E-02 | Digestive | 1.68 |
| Primary_T_cells_from_cord_blood | H3K4me3 | 2.19E-02 | Blood/Immune | 1.66 |
| Stomach_Smooth_Muscle | H3K36me3 | 2.53E-02 | Musculoskeletal/Connective | 1.60 |
| Primary_T_cells_effector_memory_enriched_from_peripheral_blood | H3K4me3 | 2.56E-02 | Blood/Immune | 1.59 |
| Primary_Natural_Killer_cells_from_peripheral_blood | H3K4me1 | 2.56E-02 | Blood/Immune | 1.59 |
| Rectal_Mucosa_Donor_29 | H3K9ac | 2.75E-02 | Digestive | 1.56 |
| Primary_T_helper_naive_cells_from_peripheral_blood_2 | H3K4me1 | 2.78E-02 | Blood/Immune | 1.56 |
| Primary_monocytes_from_peripheral_blood | H3K27ac | 3.02E-02 | Blood/Immune | 1.52 |
| Primary_T_helper_memory_cells_from_peripheral_blood_1 | H3K4me3 | 3.17E-02 | Blood/Immune | 1.50 |
| Primary_T_helper_cells_from_peripheral_blood | H3K4me3 | 3.28E-02 | Blood/Immune | 1.48 |
| Primary_neutrophils_from_peripheral_blood | H3K36me3 | 3.35E-02 | Blood/Immune | 1.47 |
| Primary_T_cells_from_cord_blood | H3K4me1 | 3.62E-02 | Blood/Immune | 1.44 |
| Stomach_Mucosa | H3K9ac | 3.66E-02 | Digestive | 1.44 |
| Rectal_Smooth_Muscle | H3K36me3 | 3.72E-02 | Musculoskeletal/Connective | 1.43 |
| Primary_B_cells_from_cord_blood | H3K4me1 | 3.99E-02 | Blood/Immune | 1.40 |
| Primary_Natural_Killer_cells_from_peripheral_blood | H3K27ac | 4.41E-02 | Blood/Immune | 1.36 |
| Spleen | H3K27ac | 4.48E-02 | Blood/Immune | 1.35 |
| Esoph-GJ | H3K4me1 | 4.63E-02 | Digestive | 1.33 |
| Primary_T_helper_memory_cells_from_peripheral_blood_1 | H3K27ac | 4.64E-02 | Blood/Immune | 1.33 |
| Primary_T_helper_memory_cells_from_peripheral_blood_2 | H3K4me3 | 4.95E-02 | Blood/Immune | 1.31 |

Table S11. Phenotypes associated with lead SNPs in each locus.

| Locus name | Lead SNP | RA_lep | OA_lep | Category | Results from PheWAS | | | | | | | | | |  |
| --- | --- | --- | --- | --- | --- | --- | --- | --- | --- | --- | --- | --- | --- | --- | --- |
|  |  |  |  |  | atlas ID | PMID | Relevant domain | Trait | Population | Ncase | Ncontrol | P-value | EA | NEA |  |
| **chr12:57,665,085 - 58,665,085** | **rs10877013** | **T** | **C** | **D*** | **1203** | **24390342** | **Connective Tissue** | **Rheumatoid Arthritis** | **EUR** | **14361** | **43923** | **7.20E-06** | **C** | **T** | |
|  |  |  |  |  | **3819** | **21833088** | **Neurological** | **Multiple Sclerosis** | **EUR** | **9772** | **17376** | **7.77E-06** | **G** | **A** |  |
| chr5:158,327,769 - 159,327,769 (*IL12B*) | rs56167332 | C | A | D* | 2031 | 28067908 | Gastrointestinal | Inflammatory Bowel Disease | EUR | 25042 | 34915 | 2.52E-38 | A | C |  |
|  |  |  |  |  | 2029 | 28067908 | Gastrointestinal | Crohn's Disease | EUR | 12194 | 28072 | 1.19E-27 | A | C |  |
|  |  |  |  |  | 2030 | 28067908 | Gastrointestinal | Ulcerative colitis | EUR | 12366 | 33609 | 1.14E-23 | A | C |  |
|  |  |  |  |  | 67 | 26192919 | Gastrointestinal | Inflammatory Bowel Disease | EUR | 12882 | 21770 | 3.26E-17 | A | C |  |
|  |  |  |  |  | 68 | 26192919 | Gastrointestinal | Crohn's Disease | EUR | 5956 | 14927 | 2.28E-11 | A | C |  |
|  |  |  |  |  | 69 | 26192919 | Gastrointestinal | Ulcerative Colitis | EUR | 6968 | 20464 | 5.30E-11 | A | C |  |
| **chr2:102,591,540 - 103,591,540** **(*IL18RAP*)** | **rs17027258** | **G** | **A** | **C*** | **3966** | **29273806** | **Respiratory** | **Asthma (fixed effect model)** | **EUR+AFR+EAS+AMR** | **23948** | **118538** | **7.54E-13** | **G** | **A** |  |
|  |  |  |  |  | **3968** | **29273806** | **Respiratory** | **Asthma (fixed effect model)** | **EUR** | **19954** | **107715** | **1.60E-12** | **G** | **A** |  |
|  |  |  |  |  | **3969** | **29273806** | **Respiratory** | **Asthma (random effect model)** | **EUR** | **19954** | **107715** | **7.72E-12** | **G** | **A** |  |
|  |  |  |  |  | **3967** | **29273806** | **Respiratory** | **Asthma (random effect model)** | **EUR+AFR+EAS+AMR** | **23948** | **118538** | **3.29E-11** | **G** | **A** |  |
|  |  |  |  |  | **4334** | **30552067** | **Respiratory** | **Asthma** | **EUR** | **5135** | **25675** | **1.92E-09** | **G** | **A** |  |
|  |  |  |  | D* | 2031 | 28067908 | Gastrointestinal | Inflammatory Bowel Disease | EUR | 25042 | 34915 | 1.72E-10 | A | G |  |
|  |  |  |  |  | 2029 | 28067908 | Gastrointestinal | Crohn's Disease | EUR | 12194 | 28072 | 4.51E-09 | A | G |  |
|  |  |  |  |  | 67 | 26192919 | Gastrointestinal | Inflammatory Bowel Disease | EUR | 12882 | 21770 | 2.91E-07 | A | G |  |
| chr1:67,097,119 - 68,097,119 (*IL23R*) | rs3762318 | A | G | D* | 2029 | 28067908 | Gastrointestinal | Crohn's Disease | EUR | 12194 | 28072 | 3.11E-12 | G | A |  |
|  |  |  |  |  | 2031 | 28067908 | Gastrointestinal | Inflammatory Bowel Disease | EUR | 25042 | 34915 | 7.24E-10 | G | A |  |
|  |  |  |  |  | 68 | 26192919 | Gastrointestinal | Crohn's Disease | EUR | 5956 | 14927 | 2.78E-08 | G | A |  |
|  |  |  |  |  | 67 | 26192919 | Gastrointestinal | Inflammatory Bowel Disease | EUR | 12882 | 21770 | 5.38E-06 | G | A |  |
| **chr16:28,008,048 - 29,008,048**  **(*IL27*)** | **rs180744** | **A** | **G** | **D*** | **2031** | **28067908** | **Gastrointestinal** | **Inflammatory Bowel Disease** | **EUR** | **25042** | **34915** | **1.62E-14** | **G** | **A** |  |
|  |  |  |  |  | **2029** | **28067908** | **Gastrointestinal** | **Crohn's Disease** | **EUR** | **12194** | **28072** | **4.50E-12** | **G** | **A** |  |
|  |  |  |  |  | **67** | **26192919** | **Gastrointestinal** | **Inflammatory Bowel Disease** | **EUR** | **12882** | **21770** | **5.62E-10** | **G** | **A** |  |
|  |  |  |  |  | **68** | **26192919** | **Gastrointestinal** | **Crohn's Disease** | **EUR** | **5956** | **14927** | **2.43E-09** | **G** | **A** |  |
|  |  |  |  |  | **2030** | **28067908** | **Gastrointestinal** | **Ulcerative colitis** | **EUR** | **12366** | **33609** | **8.74E-07** | **G** | **A** |  |
| **chr13:43,959,499 - 45,012,908** **(*LACC1*)** | **rs3764147** | **G** | **A** | C* | 2029 | 28067908 | Gastrointestinal | Crohn's Disease | EUR | 12194 | 28072 | 1.38E-13 | G | A |  |
|  |  |  |  |  | 68 | 26192919 | Gastrointestinal | Crohn's Disease | EUR | 5956 | 14927 | 7.31E-09 | G | A |  |
|  |  |  |  |  | 2031 | 28067908 | Gastrointestinal | Inflammatory Bowel Disease | EUR | 25042 | 34915 | 2.74E-08 | G | A |  |
|  |  |  |  | **D*** | **1085** | **20622878** | **Connective Tissue** | **Behcet disease** | **EUR** | **1215** | **1278** | **7.36E-06** | **A** | **G** |  |
| chr9:117,079,504 - 118,140,404 (*TNFSF15*) | rs10817678 | G | A | D* | 2031 | 28067908 | Gastrointestinal | Inflammatory Bowel Disease | EUR | 25042 | 34915 | 7.67E-27 | A | G |  |
|  |  |  |  |  | 2029 | 28067908 | Gastrointestinal | Crohn's Disease | EUR | 12194 | 28072 | 2.18E-19 | A | G |  |
|  |  |  |  |  | 2030 | 28067908 | Gastrointestinal | Ulcerative colitis | EUR | 12366 | 33609 | 4.42E-15 | A | G |  |
|  |  |  |  |  | 67 | 26192919 | Gastrointestinal | Inflammatory Bowel Disease | EUR | 12882 | 21770 | 1.17E-13 | A | G |  |
|  |  |  |  |  | 68 | 26192919 | Gastrointestinal | Crohn's Disease | EUR | 5956 | 14927 | 4.68E-12 | A | G |  |
|  |  |  |  |  | 69 | 26192919 | Gastrointestinal | Ulcerative Colitis | EUR | 6968 | 20464 | 1.26E-06 | A | G |  |

RA_lep, Risk allele in leprosy; OA_lep, Other alleles in leprosy; D*, Discordant Effect; C*, Concordant effect; atlas ID, ID in GWASATLAS database (<https://atlas.ctglab.nl/>); EA, allele increases the risk of the phenotype (for binary phenotype) or the phenotype value (for quantitative phenotype). NEA, other alleles.

Reference

1. Zhang F, Liu H, Chen S, et al. Identification of two new loci at IL23R and RAB32 that influence susceptibility to leprosy. *Nat Genet.* 2011;43(12):1247-1251.

2. Liu H, Wang Z, Li Y, et al. Genome-wide analysis of protein-coding variants in leprosy. *J Invest Dermatol.* 2017;137(12):2544-2551.

3. Liu H, Bao F, Irwanto A, et al. An association study of TOLL and CARD with leprosy susceptibility in Chinese population. *Hum Mol Genet.* 2013;22(21):4430-4437.

4. Liu H, Irwanto A, Fu X, et al. Discovery of six new susceptibility loci and analysis of pleiotropic effects in leprosy. *Nat Genet.* 2015;47(3):267-271.

5. Liu H, Irwanto A, Tian H, et al. Identification of IL18RAP/IL18R1 and IL12B as leprosy risk genes demonstrates shared pathogenesis between inflammation and infectious diseases. *Am J Hum Genet.* 2012;91(5):935-941.

6. Wang Z, Sun Y, Fu X, et al. A large-scale genome-wide association and meta-analysis identified four novel susceptibility loci for leprosy. *Nat Commun.* 2016;7:13760.

7. Marques Cde S, Brito-de-Souza VN, Guerreiro LT, et al. Toll-like receptor 1 N248S single-nucleotide polymorphism is associated with leprosy risk and regulates immune activation during mycobacterial infection. *J Infect Dis.* 2013;208(1):120-129.

8. Wong SH, Gochhait S, Malhotra D, et al. Leprosy and the adaptation of human toll-like receptor 1. *PLoS Pathog.* 2010;6:e1000979.

9. Fava VM, Dallmann-Sauer M, Orlova M, et al. Deep resequencing identifies candidate functional genes in leprosy GWAS loci. *PLoS Negl Trop Dis.* 2021;15(12):e0010029.

10. Zhang FR, Huang W, Chen SM, et al. Genomewide association study of leprosy. *N Engl J Med.* 2009;361(27):2609-2618.

11. Wang Z, Mi Z, Wang H, et al. Discovery of 4 exonic and 1 intergenic novel susceptibility loci for leprosy. *Clin Genet.* 2018;94(2):259-263.

12. Wang D, Fan Y, Malhi M, et al. Missense variants in HIF1A and LACC1 contribute to leprosy risk in Han Chinese. *Am J Hum Genet.* 2018;102(5):794-805.
